# Deuteration *versus* ethylation – strategies to improve the metabolic fate of an ^18^F-labeled celecoxib derivative†

**DOI:** 10.1039/d0ra04494f

**Published:** 2020-10-20

**Authors:** Markus Laube, Cemena Gassner, Christin Neuber, Robert Wodtke, Martin Ullrich, Cathleen Haase-Kohn, Reik Löser, Martin Köckerling, Klaus Kopka, Torsten Kniess, Evamarie Hey-Hawkins, Jens Pietzsch

**Affiliations:** Helmholtz-Zentrum Dresden-Rossendorf, Institute of Radiopharmaceutical Cancer Research Bautzner Landstrasse 400 01328 Dresden Germany m.laube@hzdr.de j.pietzsch@hzdr.de; Faculty of Chemistry and Food Chemistry, School of Science, Technische Universität Dresden Mommsenstrasse 4 D-01062 Dresden Germany; University of Rostock, Institute of Chemistry, Department of Inorganic Solid State Chemistry Albert-Einstein-Str. 3a D-18059 Rostock Germany; Leipzig University, Faculty of Chemistry and Mineralogy, Institute of Inorganic Chemistry Johannisallee 29 D-04103 Leipzig Germany

## Abstract

The inducible isoenzyme cyclooxygenase-2 (COX-2) is closely associated with chemo-/radioresistance and poor prognosis of solid tumors. Therefore, COX-2 represents an attractive target for functional characterization of tumors by positron emission tomography (PET). In this study, the celecoxib derivative 3-([^18^F]fluoromethyl)-1-[4-(methylsulfonyl)phenyl]-5-(*p*-tolyl)-1*H*-pyrazole ([^18^F]5a) was chosen as a lead compound having a reported high COX-2 inhibitory potency and a potentially low carbonic anhydrase binding tendency. The respective deuterated analog [D_2_,^18^F]5a and the fluoroethyl-substituted derivative [^18^F]5b were selected to study the influence of these modifications with respect to COX inhibition potency *in vitro* and metabolic stability of the radiolabeled tracers *in vivo*. COX-2 inhibitory potency was found to be influenced by elongation of the side chain but, as expected, not by deuteration. An automated radiosynthesis comprising ^18^F-fluorination and purification under comparable conditions provided the radiotracers [^18^F]5a,b and [D_2_,^18^F]5a in good radiochemical yields (RCY) and high radiochemical purity (RCP). Biodistribution and PET studies comparing all three compounds revealed bone accumulation of ^18^F-activity to be lowest for the ethyl derivative [^18^F]5b. However, the deuterated analog [D_2_,^18^F]5a turned out to be the most stable compound of the three derivatives studied here. Time-dependent degradation of [^18^F]5a,b and [D_2_,^18^F]5a after incubation in murine liver microsomes was in accordance with the data on metabolism *in vivo*. Furthermore, metabolites were identified based on UPLC-MS/MS.

## Abstract 2

The aim of this study is to investigate the influence of deuteration and elongation on an ^18^F-labeled COX-2 inhibitor with focus on metabolic stability to develop suitable COX-2 targeting radiotracers.

## Graphical abstract

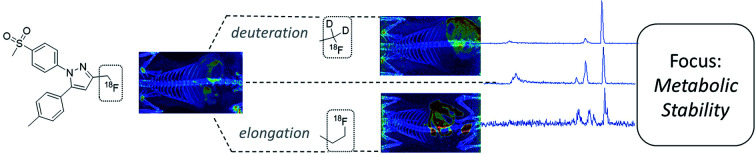

## 1 Introduction

Celecoxib (tradename Celebrex®) is an FDA-approved anti-inflammatory, analgesic, and antipyretic drug which belongs to the class of selective cyclooxygenase-2 (COX-2) inhibitors and can be prescribed for the treatment of rheumatoid arthritis, osteoarthritis, and acute pain. The development of this drug in the late 1990s represented a breakthrough in the development of anti-inflammatory drugs taking into account that the long-term gastrointestinal toxicity of common non-steroidal anti-inflammatory drugs (NSAIDs) like ibuprofen could be circumvented with this new class of COX-2-selective inhibitors (Coxibs). Today however, only a few Coxibs such as celecoxib, etoricoxib, and the prodrug parecoxib are in clinical use because of general concerns about the cardiovascular safety in long-term applications of this inhibitor class.^1,2^ The mode of action is based on the inhibition pattern for cyclooxygenase (COX), an enzyme which converts arachidonic acid to prostaglandin H_2_. Thereby, COX catalyzes a key step in the synthesis of autocrine and paracrine acting prostaglandins and thromboxane A_2_ that are involved in the regulation of a multitude of physiological and pathophysiological processes. Two isoforms of cyclooxygenases are known: the constitutively and throughout the whole body expressed isoform COX-1 which regulates mainly homeostatic processes and its counterpart COX-2 that is nearly absent in the body with exception of some organs, such as kidney and brain. However, COX-2 is induced in inflammatory conditions and neoplasia. The COX-2 pathway hence provides prostaglandins at inflamed sites and, furthermore, in tumors which makes it a potential target for diagnosis and treatment of a variety of disorders. COX-2 is overexpressed in chronic inflammatory diseases such as rheumatoid arthritis or in neurodegenerative diseases such as Parkinson's and Alzheimer's. In addition, as a key player in tumor-associated inflammation, COX-2 modulates radiation sensitivity, progression, and metastasis in a variety of cancers and leads to a reduction in overall survival.^1–8^

**Fig. 1:**
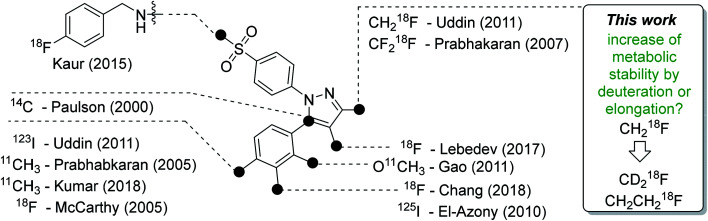
Schematic presentation of selected radiolabeled celecoxib derivatives reported in the literature^12–31^ and the aim of this work. The generalized structure shows the vicinal diphenyl-substituted pyrazole as the core structure of celecoxib derivatives. The black dots indicate only the position of the radiolabel in the respective study. Beside the radiolabel, author and year are given as a reference.

A high ‘molecular contrast’ between healthy tissue and pathological lesions also forms the basis for successful radionuclide-based approaches. From the perspective of the oncologically oriented radiochemist/radiopharmacologist, COX-2 is a highly promising drugable target because (i) under physiological conditions it is nearly absent in tissues except kidneys, heart, and brain, (ii) its expression is inducible and COX-2 is overexpressed at inflammatory sites and in (inflamed) tumor tissue, and (iii) the availability of clinically approved drugs would allow the clinician to intervene in a personalized manner in, *e.g.*, radioresistant tumors with high COX-2 expression. Among a multitude of labeled COX-2 inhibitors,^9–11^ a wide variety of celecoxib-based radiotracer approaches have been described in the literature (Fig. 1) for studies on absorption, distribution, metabolism, and excretion (carbon-14 (ref. 12)) as well as for positron emission tomography (PET; carbon-11,^13–18^ fluorine-18 (ref. 19–24)) or single photon emission computed tomography (SPECT; iodine-123/125,^25–29^, technetium-99m^30,31^). However, up to now no radiotracer could be transferred to clinical application because of high non-specific binding, metabolic instability, inability to demonstrate COX-2-specific binding *in vivo*, and/or lack of data from primates or first-in-patient studies. As one example, the [^18^F]fluoromethyl-substituted celecoxib derivative was synthesized by Uddin *et al.*^21^ and evaluated in an inflammation model as well as in tumor xenografts of COX-2-negative human colorectal carcinoma (HCT116) and COX-2-expressing human head and neck squamous cell carcinoma (1483 HNSCC) providing evidence for COX-2-specific uptake of this radiotracer *in vivo*. Unfortunately, no further studies on this radiotracer have been presented up to now. One reason might be, as shown by Uddin *et al.*,^21^ that the [^18^F]fluoromethyl-substituted radiotracer is prone to ^18^F-defluorination *in vivo* and, presumably, binds to carbonic anhydrase in the erythrocytes as it is known for celecoxib and other sulfonamide-substituted Coxibs.^28,32,33^

Based on our interest in the development of radiolabeled COX-2 inhibitors^34–37^ and to minimize non-specific binding, we aimed to synthesize the respective methylsulfonyl-substituted celecoxib derivatives and thus we herein describe two attempts to increase their metabolic stability. In general, possibilities to increase the metabolic stability of a radiotracer containing ^18^F bonded to an sp^3^-hybridized carbon atom include (i) the deuteration vicinal to the fluorine substituent^38–41^ or at other parts of the molecule,^41–43^ (ii) the replacement of an alkyl chain by a cycloalkyl ring,^44^ (iii) the use of CF_2_^18^F groups,^20^ and (iv) the application of fluorophenyl-instead of fluoroalkyl-substituted analogs wherever possible.^45–47^ As an early example, Zhang *et al.* demonstrated that utilization of a [^18^F]fluoromethoxy-*d*_2_ group in a peripheral benzodiazepine receptor ligand decreased its metabolization and, hence, ^18^F-defluorination in the brain of rats but not in primates.^39^ Accordingly, the use of [^18^F]fluoroethoxy-*d*_4_ group was found to increase the metabolic stability of verapamil analogs.^41^ In the present study, we focus on the development of a deuterated and a fluoroethyl-substituted celecoxib analog (Fig. 1). We present the synthesis, COX inhibition studies, radiolabeling with fluorine-18 as well as radiopharmacological evaluation *in vitro* and *in vivo* for three methylsulfonyl-substituted celecoxib derivatives to identify potent and metabolically stable radiotracers.

## 2 Results and discussion

### 2.1. Synthesis

**Scheme 1:**
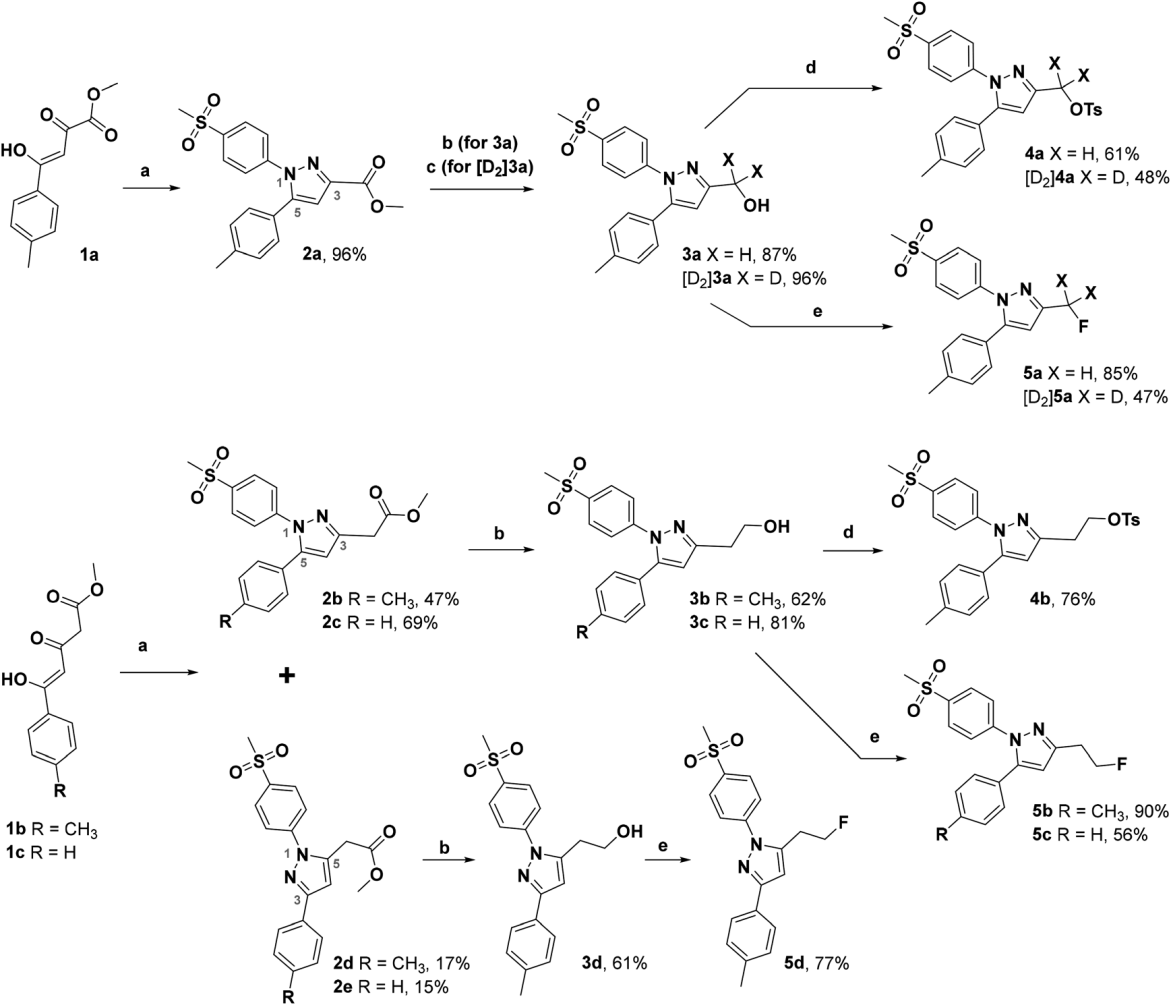
Synthesis of labeling precursors and reference compounds. Reagents and conditions: (a) 4-methylsulfonylphenylhydrazine hydrochloride, MeOH, 70 °C, 21 h; (b) LiAlH_4_, THF, 70 °C, 1.5 h or r.t., 0.5 h; (c) LiAlD_4_, THF, 70 °C, 1.5 h; (d) (Ts)_2_O, DMAP, pyridine, DCM, −10 °C → r.t., 1–2 d; (e) DAST, DCM, r.t., 2 d.

The synthetic route for the labeling precursors 4a,b and [D_2_]4a as well as the fluorine-19-substituted reference compounds 5a–d and [D_2_]5a comprises a three-step synthesis starting from the diketoacid esters 1a–c (Scheme 1).

**Fig. 2:**
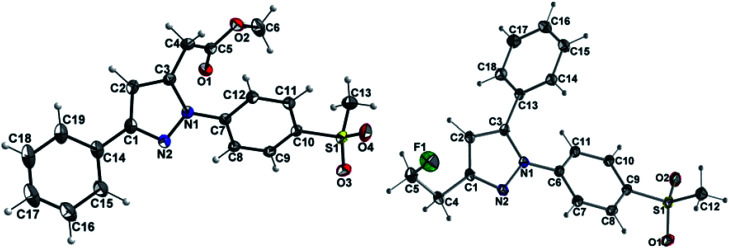
Molecular structures of compounds 2e (left) and 5c (right) in the crystal (ORTEP plot with atom labeling scheme, displacement thermal ellipsoids are drawn at 50% probability level. Only one of the two independent molecules of the asymmetric unit of 5c is shown).

At first, Knorr pyrazole synthesis by cyclocondensation of the β-dicarbonyl compounds 1a–c with methylsulfonylphenylhydrazine hydrochloride in methanol furnished the acid ester-substituted pyrazoles 2a–e in 15–96% yield. As commonly utilized for the synthesis of celecoxib, the reaction of the α,γ-diketoacid ester 1a with the respective phenylhydrazine leads predetermined by the −I and −M effect of the neighboring ester group to the initial condensation at the α-keto group and, hence, regioselectively to the 1,5-diphenyl-substituted pyrazole 2a. In comparison, in the β,δ-diketoacid ester 1b the corresponding keto group is adjacent to a methylene group exerting a +I-effect instead which disfavors the initial condensation at this site and therefore results in the non-regioselective reaction towards the 1,5-diphenyl- (2b,c: 47–69% yield) and 1,3-diphenyl-substituted (2d,e: 15–17% yield) products. The structural identity of the regioisomers was confirmed by NMR spectroscopy, and X-ray crystal structure analysis in case of 2e (Fig. 2, left). Exemplarily, 2e showed coupling in NOESY and ROESY spectra for the signal of the methylene group (*δ* = 3.91 parts per million (ppm)) to the pyrazole proton (6.85 ppm) and to the aromatic protons of the *para*-methylsulfonyl-substituted phenyl ring (7.79 ppm). In contrast, in NOESY and ROESY spectra of 2c only the coupling of the methylene group (3.76 ppm) to the pyrazole proton (6.57 ppm) was observed (for selected expansions of NOESY spectra of 2c and 2e see ESI, Fig. S1 and S2†). The asymmetric unit of crystals of 2e contains only the regioisomer 2e, not 2c (see ESI, Fig. S3†). Although the pyrazoles 2d,e do not exhibit the vicinal diaryl heterocyclic structure of Coxibs we selected 2d and further processed this compound to generate 5d as a negative control for our COX inhibition assay. In the next step, the four pyrazole derivatives 2a–d were reduced using lithium aluminum hydride in THF to form the alcohols 3a–d, and in case of 2a also with lithium aluminum deuteride in THF to form the deuterated alcohol [D_2_]3a, in 61–96% yield. The radiolabeling precursors 4a,b and [D_2_]4a, bearing a tosyl leaving group, were synthesized in 48–76% yield by conversion of the respective alcohols with tosyl anhydride in DCM utilizing 4-(dimethylamino)pyridine (DMAP) and pyridine as bases. The fluoro-substituted reference compounds 5a–d and [D_2_]5a were obtained by fluorination of the alcohols 3a–d and [D_2_]3a, respectively, with (diethylamino)sulfur trifluoride (DAST) in DCM in 47–90% yield. The X-ray crystal structure obtained for the 1,5-diphenyl-3-fluoroethyl-substituted reference compound 5c (Fig. 2, right) confirmed the structural identity of this regioisomer. Crystals of 5c contained two symmetry-independent molecules in the asymmetric unit (ESI, Fig. S4†). Both have the same group connectivity, such that they are the same regioisomers. They only differ in dihedral angles around single bonds (*i.e.* C1–C4, C3–C13, C9–S1) as a result of crystal packing effects.

### 2.2. COX inhibition

COX inhibition potency was determined *in vitro* for the reference compounds 5a–d and [D_2_]5a (Table 1) using purified ovine COX-1 and recombinant human COX-2 enzyme in the commercially available ‘COX Fluorescent Inhibitor Screening Assay KIT’ (Cayman Chemical, Ann Arbor, MI). The data obtained for the COX-2-selective inhibitor 5a are in accordance with the inhibition pattern previously determined by Uddin *et al.* with a ^14^C-based COX assay.^21^ As expected, deuteration did not markedly change COX-2 inhibitory potency of [D_2_]5a, while both prolongation to the fluoroethyl chain resulting in the tolyl derivative 5b and the lack of a methyl substituent in derivative 5c weakened the inhibitory potency and selectivity for COX-2. The regioisomer 5d, which does not contain the typical vicinal diarylheterocyclic structure of Coxibs, showed neither COX-2 nor COX-1 inhibition as anticipated.

**Table 1:** COX inhibition data for compounds 5a–d and [D_2_]5a

		R^1^	R^2^	IC_50_ (COX-1) [μM]	IC_50_ (COX-2) [μM]	Selectivity index^c^
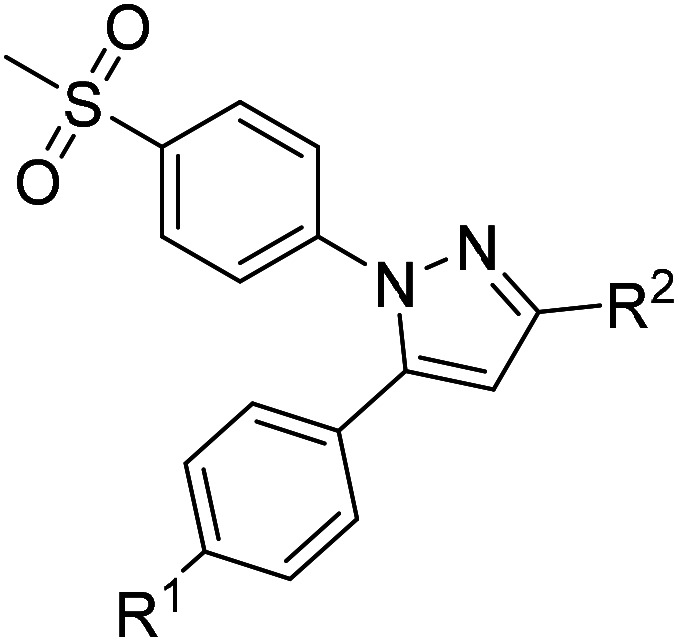	5a^a^	CH_3_	CH_2_F	>4	0.60	>7
	5a	CH_3_	CH_2_F	>100	0.51	>196
	[D_2_]5a	CH_3_	CD_2_F	>100	0.97	>103
	5b	CH_3_	CH_2_CH_2_F	38.51	2.53	15
	5c	H	CH_2_CH_2_F	>100	4.97	>20
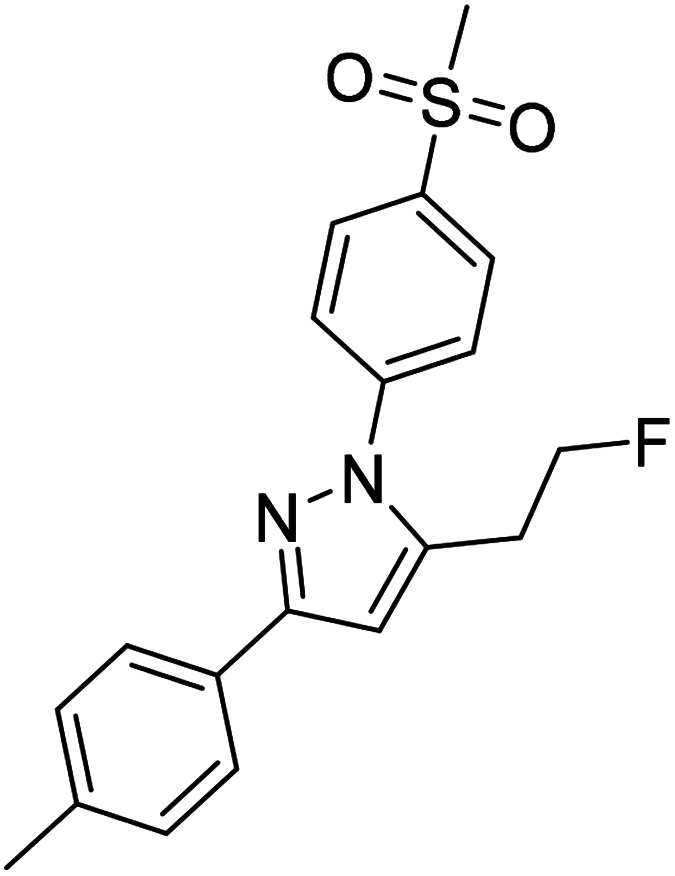	5d	—	—	>100	>100	—
Celecoxib				>100	0.04 ± 0.02^b^	

a Previously reported by Uddin *et al.*^21^

b Mean ± SD of six independent experiments.

c Selectivity index = IC_50_ (COX-1)/IC_50_ (COX-2).

### 2.3. Radiosynthesis

**Scheme 2:**
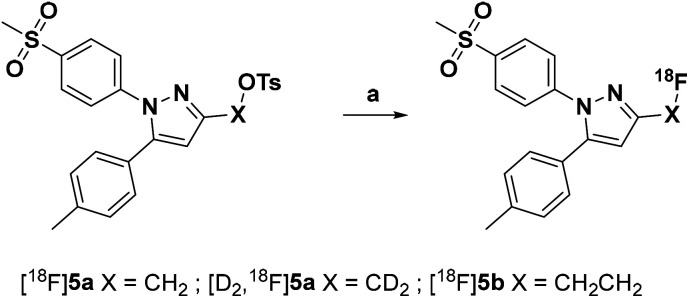
Radiosynthesis of the ^18^F-labeled celecoxib derivatives [^18^F]5a,b and [D_2_,^18^F]5a. Reagents and conditions: (a) [^18^F]fluoride/K_2_CO_3_/Kryptofix® 222 (K_222_), MeCN, 80 °C, 15 min.

Radiolabeling with [^18^F]fluoride was achieved starting from the tosylated precursors 4a,b and [D_2_]4a under standard conditions (Scheme 2 and Table 2) in a synthesis sequence using an automated radiosynthesizer (TRACERlab FX-N). After azeotropic drying of [^18^F]fluoride, labeling was performed at 80 °C in acetonitrile for 15 min followed by dilution of the mixture with eluent, filtration, and purification by semi-preparative HPLC. In order to obtain the radiotracer in high chemical purity, we adjusted the eluent to 40% MeCN/60% water with 0.1% trifluoroacetic acid (TFA) and allowed the tracers to be eluted from the HPLC column after a separation time of approximately 50 min. After a C_18_-based solid phase extraction to remove the eluent, the final tracers [^18^F]5a,b and [D_2_,^18^F]5a were isolated in radiochemical yields (RCY) between 33 and 38% and high radiochemical purity (RCP > 99%) as well as chemical purity (CP > 92%) as an ethanolic solution with high activity concentration suitable for further *in vitro* and *in vivo* applications (for radio-HPLC chromatograms and radio-TLC data of final radiotracers, see ESI, Fig. S5†). The log *D* value (pH 7.4) was determined to be 2.72 for [^18^F]5a and [D_2_,^18^F]5a, and 2.65 for [^18^F]5b by an HPLC-based method.^35,48^

**Table 2:** Results of radiosyntheses for the ^18^F-labeled celecoxib derivatives [^18^F]5a,b and [D_2_,^18^F]5a

	[^18^F]5a	[D_2_,^18^F]5a	[^18^F]5b
Isolated RCY^a^	35 ± 9% (*n* = 8)	33 ± 6% (*n* = 4)	38 ± 8% (*n* = 6)
*A*_m_ [GBq μmol^−1^]	7–54	13–44	4–40
RCP	>99%	>99%	>99%
CP	>94%	>92%	>94%
Synthesis time^b^	116 min (*n* = 8)	129 min (*n* = 4)	121 min (*n* = 6)
log *D*_7.4,HPLC_	2.72	2.72	2.65

a Mean ± SD of *n* independent experiments.

b Mean of *n* independent experiments.

### 2.4. Cellular uptake

**Fig. 3:**
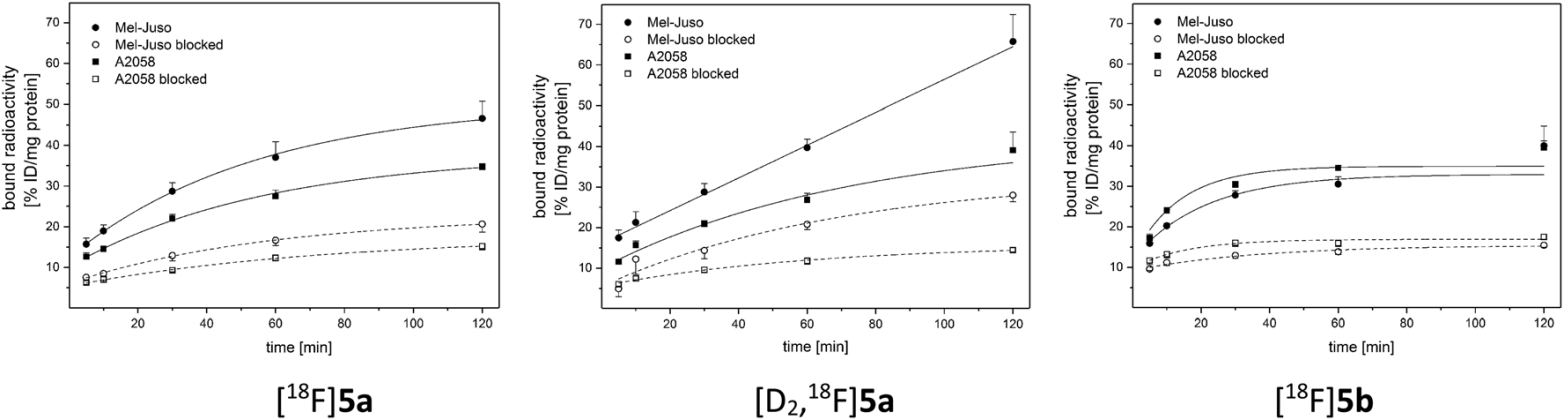
Representative cellular binding and uptake of [^18^F]5a,b and [D_2_,^18^F]5a in COX-2-negative Mel-Juso and COX-2-positive A2058 cells at 37 °C. Blocking was performed by preincubation with 100 μM celecoxib. Results are shown as mean ± SD of one representative experiment performed in quadruplicate.

For evaluation of COX-2 selectivity *in vitro*, cell uptake was studied in two melanoma cell lines with known COX expression pattern:^35^ the COX-2-negative Mel-Juso and COX-2-positive A2058 cell line, both expressing COX-1 at low but constitutive levels. Cellular binding and uptake of [^18^F]5a,b and [D_2_,^18^F]5a was found to be independent of the COX-2 expression since cellular uptake by COX-2-negative Mel-Juso cells was slightly higher compared to uptake by COX-2-positive A2058 cells (Fig. 3). Furthermore, inhibition with the known COX-2-selective inhibitor celecoxib resulted in a reduction of cell uptake, however, in both cell lines in a comparable manner pointing to a saturation of other binding sites instead of COX-2. For celecoxib it is known that it can also bind and interact with other targets which contributes to its anticarcinogenic effects.^49–51^ As examples, interactions with moderate affinity like the inhibition of 3-phosphoinositide-dependent kinase 1 (PDK1, IC_50_ = 3.5 μM,^52^ IC_50_ = 48 μM (ref. 53)), protein kinase B (PKB, IC_50_ = 28 μM (ref. 50 and 53)), phosphodiesterase-5 (PDE51A, IC_50_ = 16 μM (ref. 54)), or sarcoplasmic/ER Ca^2+^ ATPase (SERCA, IC_50_ = 35 μM (ref. 49 and 55)) and the high affinity interaction with carbonic anhydrases (CA, hCA-IX IC_50_ = 16 nM, hCA-XII IC_50_ = 18 nM (ref. 49 and 56)) have been described. Compounds 5a,b and [D_2_]5a lack the sulfonamide group which is a major determinant of substrate and CA inhibitor specificity^57^ and hence are not likely potent CA inhibitors. However, it is possible that other interactions play a role in cellular binding effects observed in this investigation because in both cell lines a COX-independent blocking effect was observed at a concentration of 100 μM celecoxib which is higher than the IC_50_ value of the interactions with moderate affinity described above. Because of that and insufficient COX inhibition potencies, compounds 5a,b and [D_2_]5a did not meet the requirements for *in vivo* studies in tumor xenograft-bearing mice. Instead, we focused on biodistribution studies in healthy rats to evaluate the influence of deuteration or elongation on metabolic stability for the celecoxib-based radiotracers.

### 2.5. Metabolic stability *in vivo*

To assess the *in vivo* behavior of [^18^F]5a,b and [D_2_,^18^F]5a in healthy rats with focus on metabolic stability, (i) *ex vivo* biodistribution at 5 and 60 min post injection (p.i.) was analyzed, (ii) small animal PET imaging was performed up to 90 min p.i., and (iii) blood samples were analyzed for metabolites at 20 and 60 min p.i.

**Fig. 4:**
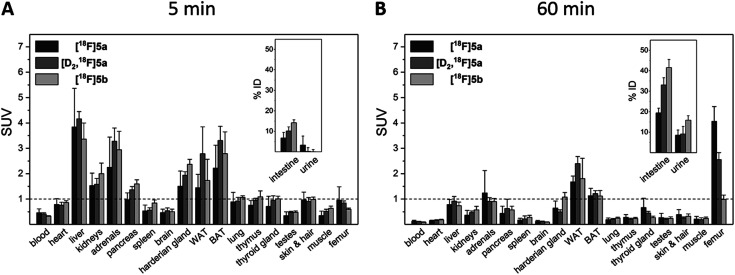
Biodistribution of [^18^F]5a,b and [D_2_,^18^F]5a in healthy rats at 5 min (A) and 60 min p.i. (B). Results are shown as mean ± SD of two independent experiments each performed in quadruplicate (8 animals for each time point and tracer).

Biodistribution experiments (Fig. 4, see ESI, Tables S5 and S6†) showed a similar pharmacokinetic pattern for [^18^F]5a,b and [D_2_,^18^F]5a. At 5 min p.i., the highest initial radioactivity concentration was found in liver, adrenals, and brown as well as white adipose tissue (BAT/WAT). Whereas radioactivity concentration considerably decreased in almost all organs from 5 to 60 min p.i., radioactivity concentration in intestine, urine, and femur increased over time. At 60 min p.i., the hepatobiliary and renal excretion was found to increase within the row [^18^F]5a < [D_2_,^18^F]5a < [^18^F]5b resulting in liver-to-bladder ratios of 2.3, 3.2, and 2.6. By contrast to excretion, ^18^F-defluorination tendency clearly decreased in the row [^18^F]5a > [D_2_,^18^F]5a > [^18^F]5b resulting in femur uptakes (standardized uptake value, SUV) of 4.1 ± 0.6, 2.6 ± 0.3, and 1.0 ± 0.2, respectively.

**Fig. 5:**
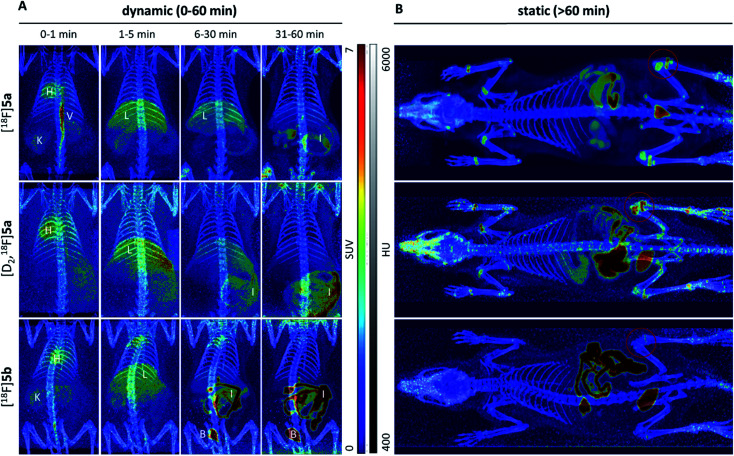
Maximum intensity projections of small animal PET imaging after i.v. injection of [^18^F]5a, [D_2_,^18^F]5a, and [^18^F]5b in healthy rats. Tracer distribution was investigated for 60 min p.i. in dynamic mode (A) and, afterwards, the whole animal was scanned in static mode (B). Different organs are marked as H-heart, V-vein, K-kidney, L-liver, I-intestine, and B-bladder. For better visualization, PET images (scaled to SUV_max_ = 7.0) are overlayed with CT images (scaled between 400 and 6000 Houndsfield Units (HU)).

Small animal PET imaging was in accordance with the biodistribution experiments showing a rapid clearance of [^18^F]5a, [D_2_,^18^F]5a, and [^18^F]5b from the blood and a fast excretion of radioactivity into the intestine and the bladder (Fig. 5). In line with the lipophilicity of the compounds, hepatobiliary excretion predominated renal excretion for all three tracers. From the dynamic PET study it appears that [^18^F]5b gets eliminated even faster than [^18^F]5a or [D_2_,^18^F]5a. However, with regard to ^18^F-defluorination, [^18^F]5b was clearly superior to [^18^F]5a or [D_2_,^18^F]5a, which means that prolongation of the alkyl chain is beneficial resulting in significantly decreased radioactivity concentration in epiphyses (Fig. 5 red circle) and skeleton. These results are in accordance with previous reports showing that deuteration^38–41^ and prolongation of fluoro-substituted alkyl chains^58^ decreased the ^18^F-defluorination rate *in vivo*. Of note, prolongation of the alkyl chain does not necessarily influence the stability in blood plasma as shown for *O*-[^18^F]fluoromethyl-, *O*-[^18^F]fluoroethyl, and *O*-[^18^F]fluoropropyl tyrosine derivatives^58^ or even increased ^18^F-defluorination as shown for 5-[^18^F]fluoroalkyl pyrimidine nucleosides^59^ in a comparison between propyl-, butyl-, and pentyl-substituted derivatives.

**Fig. 6:**
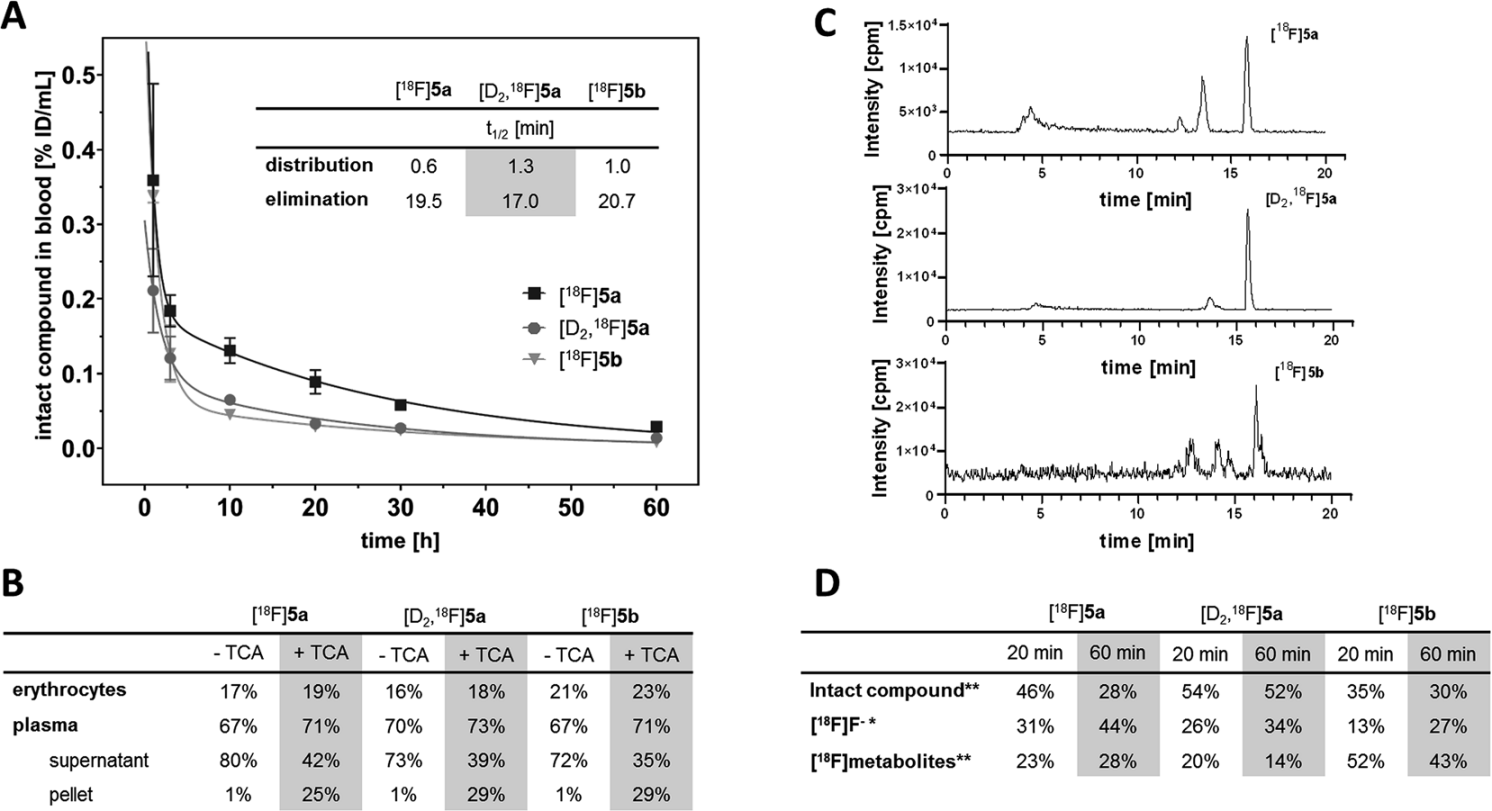
(A) Blood clearance rate (% ID per mL of intact compound) after i.v. injection of [^18^F]5a,b and [D_2_,^18^F]5a. (B) Radioactivity distribution in blood components with and without TCA precipitation. (C) Analytical radio-HPLC chromatograms of rat blood plasma at 60 min p.i. (D) Ratio of [^18^F]fluoride, intact compound, and radiometabolites at 20 and 60 min p.i. determined by radio-TLC (*) and radio-HPLC (**).

With the aim to elucidate the differences in the metabolic fate of [^18^F]5a, [D_2_,^18^F]5a, and [^18^F]5b, metabolites of the three radiotracers were analyzed in samples of blood as well as of liver, urine, and intestinal content by radio-HPLC (Fig. 6, see ESI, Fig. S6–S8†) and radio-TLC (see ESI, Fig. S9†) after i.v. injection in healthy rats. Analysis of blood samples (Fig. 6A) confirmed the rapid blood clearance of [^18^F]5a, [D_2_,^18^F]5a, and [^18^F]5b resulting from very fast tracer distribution (*t*_1/2_ = 0.6, 1.3, and 1.0 min, respectively) followed by a considerably slower tracer elimination (*t*_1/2_ = 19.5, 17.0, and 20.7 min). At 60 min p.i., for the three radiotracers 16–23% of activity in blood was found in erythrocytes, whereas about 70% of activity in blood was retrieved in blood plasma (Fig. 6B). Precipitation with trichloroacetic acid (TCA) revealed that about 25–29% of activity in blood was bound to plasma proteins. Low binding of [^18^F]5a,b and [D_2_,^18^F]5a to erythrocytes is in line with the absence of a sulfonamide group typically interacting with carbonic anhydrase. With regard to metabolization, the formation of free [^18^F]fluoride and more hydrophilic metabolites was observed for all three radiotracers (Fig. 6C). However, radiotracers showed a different number of metabolites and different amounts of intact compound. For all three radiotracers, no intact parent compound was excreted into the urinary bladder or the intestine so that only metabolites and [^18^F]fluoride could be observed there.

At 60 min p.i., 28% of intact [^18^F]5a was found in blood (Fig. 6D) together with two more hydrophilic metabolites (*Σ* = 28%). Both metabolites were found to be excreted into the urine (ESI, Fig. S6 and S9†). In comparison, 52% of the deuterated analog [D_2_,^18^F]5a was intact at 60 min p.i. and only one radioactive metabolite (14%) was present (Fig. 6D). However, two metabolites were observed in intestinal content and urine (ESI, Fig. S7 and S9†).

For the [^18^F]fluoroethyl derivative [^18^F]5b, 30% of the parent compound was intact at 60 min p.i (Fig. 6D). showing the presence of three metabolites in blood (*Σ* = 43%). For this tracer, excretion into the intestine *via* four metabolites and into the urinary bladder *via* two metabolites was observed (ESI, Fig. S8 and S9†). Hence, excretion into the urinary bladder and the intestine are dominant factors determining the metabolic fate of [^18^F]5a,b and [D_2_,^18^F]5a which is in accordance with other results from the literature.

The lead celecoxib is metabolized by CYP2C9 and to lower extent (<25%) by CYP3A4 to the hydroxyl form, oxidized to the carboxyl derivative by cytosolic alcohol dehydrogenases ADH1 and ADH2, and finally converted to a minor extent to the *O*-glucuronide by UDP glucuronosyltransferases.^12,51^ Also Takashima-Hirano *et al.*^13,14^ showed that isotopically labeled [^11^C]celecoxib is oxidized *via* the hydroxymethyl form to the carboxylic acid derivative. While in blood and liver [^11^C]celecoxib was found to be mostly intact, the carboxylic acid was the major radioactive component in the bile suggesting dominant excretion of this radiometabolite into the intestine. Similarly, for [^18^F]5a,b and [D_2_,^18^F]5a only more polar radiometabolites were found to be excreted into the urinary bladder and the intestine while both the intact compound and radiometabolites were present in blood plasma. The deuterated analog [D_2_,^18^F]5a showed faster blood clearance compared to [^18^F]5a (Fig. 6A) presumably caused by both tissue tracer distribution of intact compound, *e.g.*, to white adipose tissue (Fig. 4) as well as a metabolic shift towards the more extensive oxidation to the carboxylic acid and excretion in this form. Of note, a fast metabolization of [^11^C]celecoxib was also observed in male baboon, *i.e.* only 17% of intact radiotracer was found at 90 min p.i. in blood.^18^

### 2.6. Metabolic stability *in vitro*

Murine liver microsome assay was performed to investigate the time-dependent formation of ^18^F-bearing metabolites and their structure in more detail. Different liver subcellular fractions such as liver microsomes, liver S9 fractions, and liver cytosol are principally available differing in their capability to catalyze phase I and phase II reactions. We decided to use liver microsomes to follow cytochrome P450 (CYP)-mediated phase I oxidation to the hydroxylated metabolites as rate limiting steps in radiotracer metabolization. Moreover, liver microsomes contain flavin monooxygenases and uridine glucuronide transferase, which allow further phase I and II transformations. Enzymes like aldehyde oxidase, glutathione transferase, sulfotransferase, or other cytosolic cofactors are not present in this subcellular fraction.^60^

**Fig. 7:**
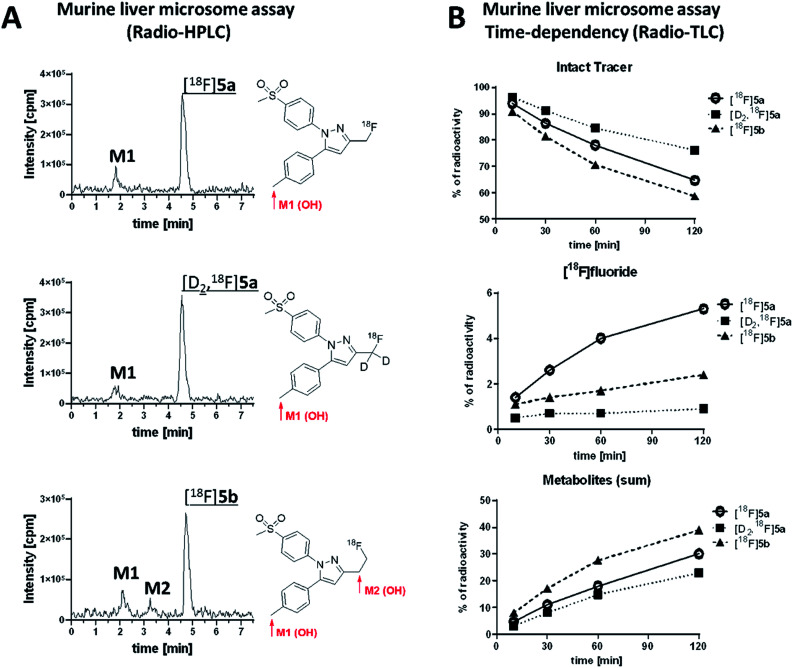
(A) Analytical radio-HPLC chromatograms of [^18^F]5a, [D_2_,^18^F]5a, and [^18^F]5b incubated for 60 min with murine liver microsomes and structure of metabolites determined by UPLC-MS/MS; (B) time dependency of metabolism of intact tracer and formation of [^18^F]fluoride and ^18^F-metabolites based on radio-TLC.

For the experiment, [^18^F]5a, [D_2_,^18^F]5a, and [^18^F]5b were prepared at the same day to ensure comparable activity of the liver microsomes. All three radiotracers were subjected to oxidative conditions in the murine liver microsome assay for 10, 30, 60, and 120 min and samples were analyzed by radio-HPLC and radio-TLC (Fig. 7). Furthermore, carrier-added samples were subjected to the same conditions and analyzed after radioactive decay by UPLC-MS/MS to identify the structure of the metabolites. While for [^18^F]5a and [D_2_,^18^F]5a the formation of one major metabolite was observed, two metabolites were formed from [^18^F]5b (Fig. 7A and ESI, Fig. S13–S24†). Of note, glucuronidation was exemplarily investigated for [^18^F]5b by applying oxidative conditions as described in the experimental section with addition of MgCl_2_ (5 mM), alamethicin (50 μg mL^−1^), and uridine diphosphate glucuronic acid (UDPGA, 5 mM) but glucuronidation was not observed in this experimental set-up (data not shown). UPLC-MS/MS experiments verified the formation of the respective hydroxylated ^18^F-bearing metabolites for all three compounds as well as the formation of a defluorinated and hydroxyl-substituted metabolite in case of [^18^F]5a and [D_2_,^18^F]5a. The time-dependent formation of [^18^F]fluoride and other ^18^F-bearing metabolites was found to follow different trends. While ^18^F-defluorination decreased in the order [^18^F]5a > [^18^F]5b > [D_2_,^18^F]5a, the amount of metabolites bearing covalently bound ^18^F increased in the order [D_2_,^18^F]5a < [^18^F]5a < [^18^F]5b (Fig. 7B and ESI, Fig. S10–S12†). Hence, deuteration effectively enhanced the metabolic stability as observed *in vitro* and *in vivo*.

Our results showed that oxidative metabolism represents a dominant factor determining the metabolic fate of [^18^F]5a,b and [D_2_,^18^F]5a which is in accordance with previous reports. For example, after administration of a non-radioactive fluoromethyl-substituted celecoxib derivative Uddin *et al.*^21^ also identified the oxidatively defluorinated metabolite bearing a hydroxymethyl group in the inflamed footpad. In accordance, [^11^C]celecoxib is oxidized to the carboxylic acid derivative,^13,14^ a fact that also causes fast metabolization of [^11^C]celecoxib in male baboon^18^ which shows the need for metabolic stabilization also in primates. Deuteration as shown for [D_2_,^18^F]5a in this study effectively suppressed metabolization *in vitro* and *in vivo* suggesting that this might be a useful strategy for further developments. Interestingly, other studies indicated that the metabolic degradation at the 5-phenyl ring can be lowered as well. For example, a ^18^F-labeled celecoxib derivative having a 4-[^18^F]fluoro-5-phenyl-pyrazole^22^ as structural key feature but no oxidizable methyl group has been reported to be metabolically stable and was found to be intact in brain, liver, intestine, and blood plasma of healthy mice. Also, only a small amount of metabolites has been observed for the *ortho*-^18^F-celecoxib derivative^23^ which has a fluorine in *ortho*-position to the methyl group indicating that steric hindrance or substitution nearby can also lower the oxidative metabolism at this site. For other celecoxib derivatives, only non-specific^17,19,24,26,28^ or no^16,20,25,27,29–31^ information about the detailed metabolism is currently available.

## 3 Conclusion

**Fig. 8:**
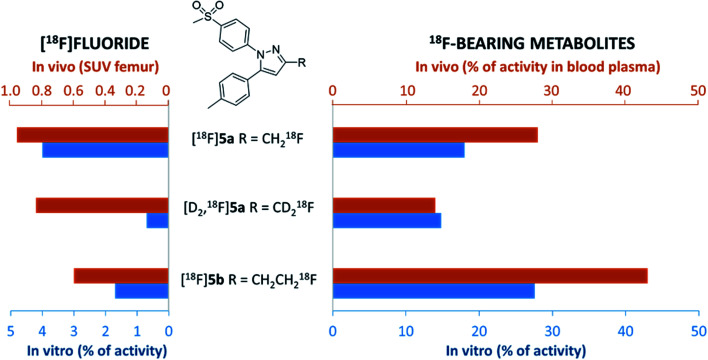
Comparison of ^18^F-defluorination and ^18^F-bearing metabolite formation tendency obtained for [^18^F]5a, [D_2_,^18^F]5a, and [^18^F]5b*in vitro* and *in vivo* based on selected results of this study. Formation of [^18^F]fluoride *in vivo* (left, orange bars) refers to SUV values for femur obtained in biodistribution studies 60 min p.i. Formation of ^18^F-bearing metabolites *in vivo* (right, orange bars) refers to % of activity in blood plasma samples as analyzed by radio-TLC and radio-HPLC 60 min p.i. Formation of [^18^F]fluoride and ^18^F-bearing metabolites *in vitro* (blue bars) refers to % of activity as analyzed by radio-TLC after 60 min incubation with murine liver microsomes.

In this study, alterations in the substitution pattern of a celecoxib derivative were studied with respect to deuteration and elongation of the side chain in position 3 of the pyrazole ring. The radiotracers [^18^F]5a,b and [D_2_,^18^F]5a were examined within detailed *in vitro* and *in vivo* studies. COX inhibitory potency of [D_2_]5a was found to be comparable to the potent and selective COX-2 inhibitor 5a, while the ethyl derivative 5b turned out to be slightly less potent. However, all three radiotracers [^18^F]5a,b and [D_2_,^18^F]5a did not show COX-2 specific binding *in vitro* so that radiopharmacological evaluation *in vivo* focused on metabolic stability in healthy rats. Our results demonstrate marked effects of both deuteration and elongation on ^18^F-defluorination and formation of ^18^F-bearing metabolites *in vitro* and *in vivo* (Fig. 8).

In principle, oxidative metabolism and excretion to urinary bladder and intestine were found to be dominant factors determining the metabolic fate of all three investigated radiotracers which is in accordance with other results from the literature. As a main result, deuteration was shown to be most beneficial as it effectively decelerates metabolic transformation without compromising the biological activity of the molecule. In this sense, it is part of ongoing research to envisage the additional exchange of the CH_3_ with a CD_3_ group at the tolyl ring to result in a radiotracer candidate with highest possible metabolic stability for this kind of substance class. Other COX-2-targeting radiotracers, *e.g.* the [^18^F]fluoromethyl-substituted valdecoxib derivative^61^ for which the evaluation in monkey was substantially hampered by ^18^F-defluorination, might accordingly benefit from similar modifications.

## Ethical statement

All animal experiments were carried out according to the guidelines of the German Regulations for Animal Welfare. The protocols were approved by the local Ethical Committee for Animal Experiments (Landesdirektion Sachsen; AZ 24-9168.21-4/2004-1).

## Author contributions

Conceptualization and design: M. L., T. K., J. P.; methodology and validation: M. L., J. P.; investigation (acquisition, analysis and interpretation of data): M. L., C. G., C. N., R. W., R. L., M. K., E. H.-H.; writing – original draft: M. L., C. G., C. N., R. W., R. L.; writing – review and editing: M. L., M. U., C. H.-K., K. K., E. H.-H., J. P.; administrative, technical, or material support: K. K., J. P.; study supervision: J. P.

## Conflicts of interest

There are no conflicts to declare.

## Supplementary Material

RA-010-D0RA04494F-s001

RA-010-D0RA04494F-s002
