# Supplementary material for: Deuteration versus ethylation – strategies to improve the metabolic fate of an 18F-labeled celecoxib derivative
Source: RSC Adv. 2020 Oct 20;10(63):38601–11. doi: 10.1039/d0ra04494f (PMC9057277; doi:10.1039/d0ra04494f)
Supplement: RA-010-D0RA04494F-s001 [file RA-010-D0RA04494F-s001.pdf]

Electronic Supplementary Material (ESI) for RSC Advances.
This journal is © The Royal Society of Chemistry 2020

Supporting Information

Deuteration versus ethylation – strategies to improve the metabolic fate of a ^{18}F-labeled celecoxib derivative

Markus Laube[a,,#], Cemena Gassner[a,b,*], Christin Neuber[a,*], Robert Wodtke[a], Martin Ullrich[a], Cathleen Haase-Kohn[a], Reik Löser[a], Martin Köckerling[c], Klaus Kopka[a], Torsten Kniess[a], Evamarie Hey-Hawkins[d], and Jens Pietzsch[a,b,#]*

Affiliations

[a]Helmholtz-Zentrum Dresden-Rossendorf, Institute of Radiopharmaceutical Cancer Research, Bautzner Landstrasse 400, 01328 Dresden, Germany

[b]Faculty of Chemistry and Food Chemistry, School of Science, Technische Universität Dresden, Mommsenstrasse 4, D-01062 Dresden, Germany

[c]University of Rostock, Institute of Chemistry, Department of Inorganic Solid State Chemistry, Albert-Einstein-Str. 3a, D-18059 Rostock, Germany.

[d]Leipzig University, Faculty of Chemistry and Mineralogy, Institute of Inorganic Chemistry, Johannisallee 29, D-04103 Leipzig, Germany.

* M.L., C.G., and C.N. contributed equally to this manuscript.

[#]corresponding authors: Markus Laube, E-mail: m.laube@hzdr.de; Jens Pietzsch, j.pietzsch@hzdr.de

Table of contents

1. Expansion of NOESY spectrum of compound 2c and 2e 3
2. Crystal structure data of 2e and 5c 4
3. HPLC and TLC of final radiotracers [^{18}F]5a,b, and [D_2,^{18}F]5a 8
4. Biodistribution data of [^{18}F]5a,b, and [D_2,^{18}F]5a 9
5. Copies of radio-HPLC/TLC chromatograms of metabolite studies...11
6. Structural elucidation of MLM-metabolites via UPLC-MS/MS 16
7. Experimental Section 22
8. Copies of ^{1}H NMR and ^{13}C NMR spectra of new compounds 38
9. References 79

1. Expansion of NOESY spectrum of compound 2c and 2e

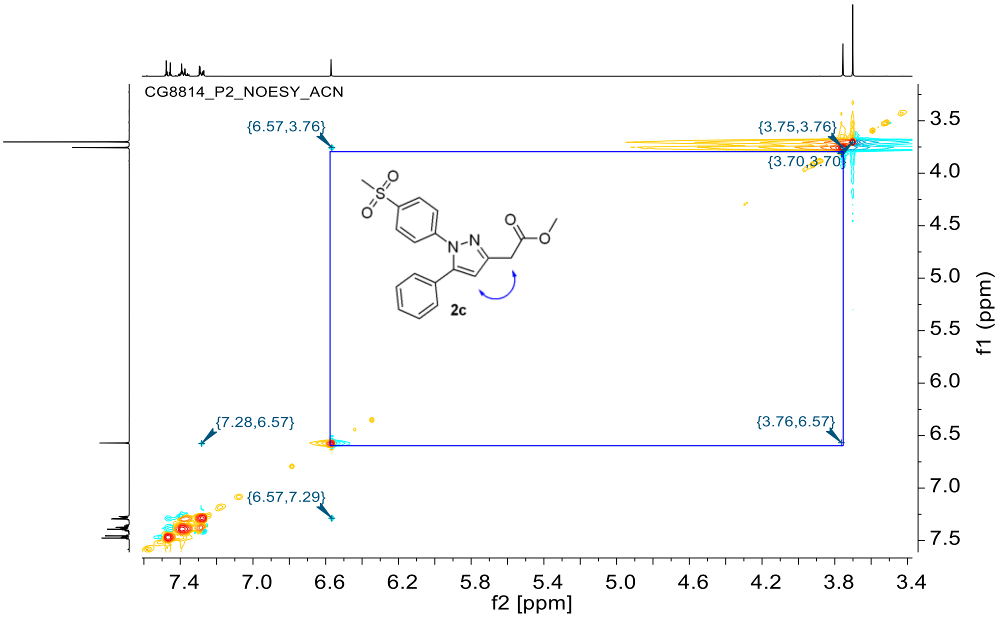

Figure S1. Expansion of NOESY spectrum of compound **2c** in CD_3CN. Significant correlations for the determination of regioisomer identity are highlighted as dashed line in the spectrum and arrow in the structure.

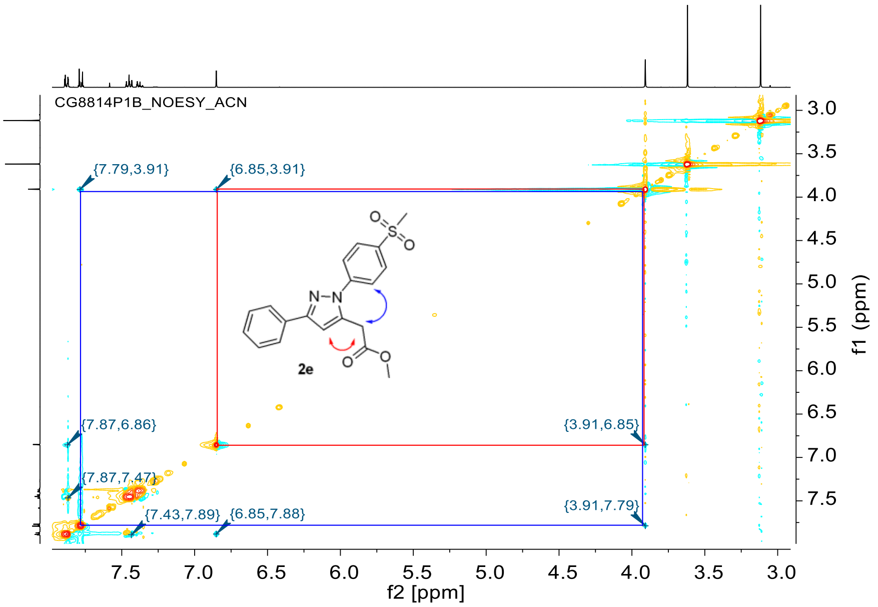

Figure S2. Expansion of NOESY spectrum of **2e** in MeCN. Significant correlations for the determination of regioisomer identity are highlighted as dashed line in the spectrum and arrow in the structure.

2. Crystal structure data of 2e and 5c

Crystal Structure data of 2e

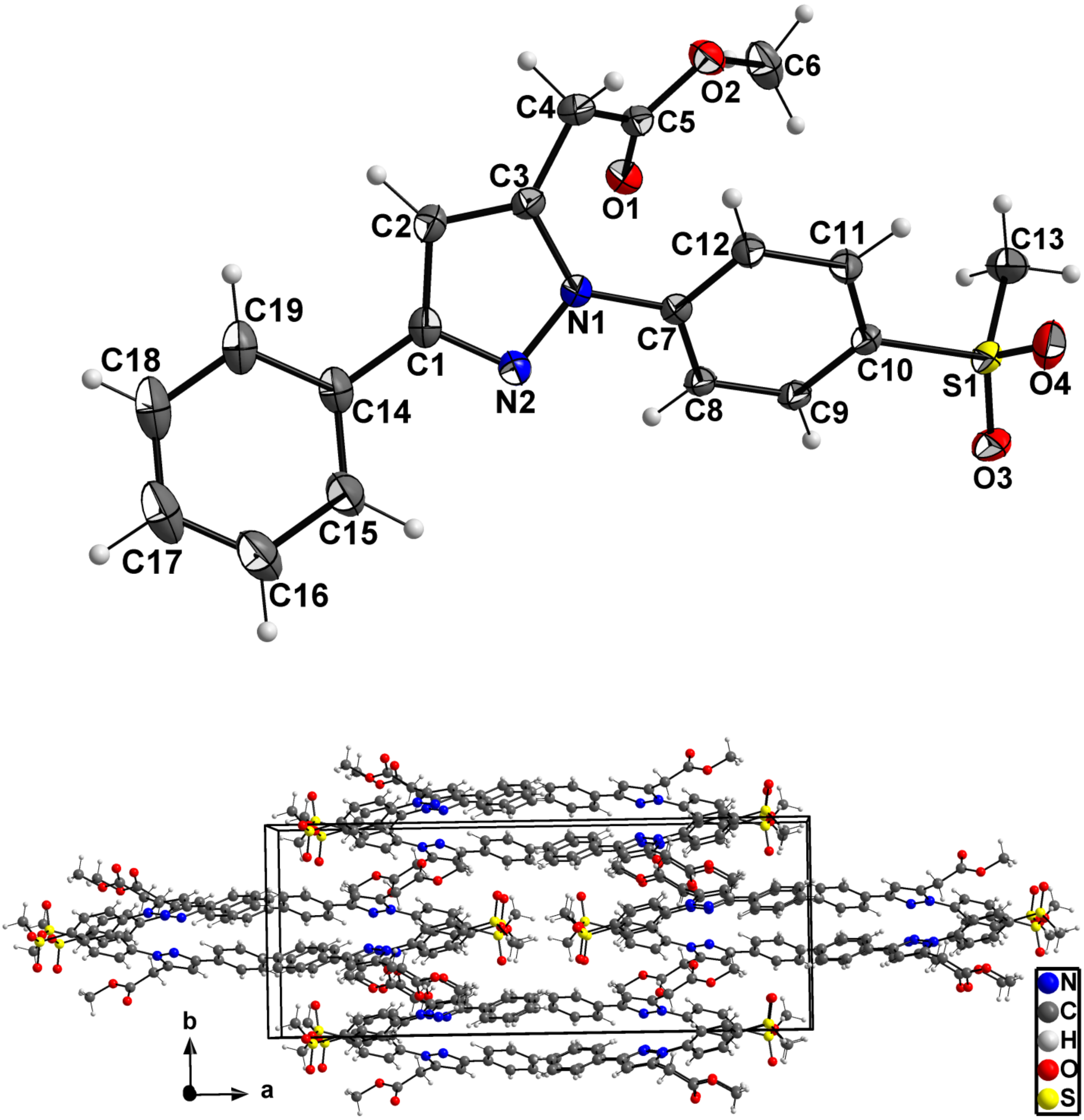

Figure S3. Molecular structure of compound **2e** (top, ORTEP plot with atom labeling, displacement thermal ellipsoids are drawn at the 50% probability level) and crystal packing (bottom).

Table S1. Crystal, structure solution and refinement data of **2e**

Chemical formula	$C_{19}H_{18}N_2O_4S$
*M*r	370.41
Crystal system, space group	Monoclinic, *C*2/*c*
Temperature (K)	123
a, *b*, *c* (Å)	28.442(1), 11.1139(4), 11.4467(4)
β (°)	90.780(2)
V (Å^3)	3618.0(2)
Z	8
Radiation type	Mo*Kα*
μ (mm^{-1})	0.20
Crystal size (mm^3)	0.35 × 0.28 × 0.12
Diffractometer	Bruker *APEX*-II CCD
Absorption correction	Multi-scan, Brunker-Nonius, SADABS
No. of measured, indep. refl.	52794, 8655
observed [$I > 2\sigma(I)$] refl.	7227
*R*int	0.035
(sin θ/λ)max (Å^{-1})	0.830
$R[F^2 > 2\sigma(F^2)]$, $wR(F^2)$, S	0.036, 0.112, 1.03
No. of reflections	8655
No. of parameters	307
H-atom treatment	All H-atom parameters refined
Δ*ρ*max, Δ*ρ*min (e Å^{-3})	0.53, −0.46

Computer programs: Bruker *APEX2*, Bruker *SAINT*, *SHELXS2014*/1 (Sheldrick, 2014), *SHELXL14* (Sheldrick, 2014).

Table S2. Hydrogen-bond geometry (Å, º)

D—H···*A*	*D*—H	H···*A*	*D*···*A*	*D*—H···*A*
C4—H4A···O2^{i}	0.99(1)	2.51(1)	3.408(1)	151(1)
C6—H6A···O4^{ii}	0.95(2)	2.59(2)	3.501(1)	161(1)
C12—H12···O1^{iii}	0.97(1)	2.51(1)	3.123(1)	121(1)
C13—H13A···O3^{ii}	1.00(2)	2.47(2)	3.474(1)	178(1)
C13—H13C···O3^{iv}	0.97 (2)	2.53(2)	3.493(1)	174(1)

Symmetry codes: (i) −*x*+1/2, −*y*+3/2, −*z*+2; (ii) *x*, −*y*+1, *z*+1/2; (iii) −*x*+1/2, *y*−1/2, −*z*+3/2; (iv) −*x*+1, −*y*+1, −*z*+1.

Crystal structure data of 5c

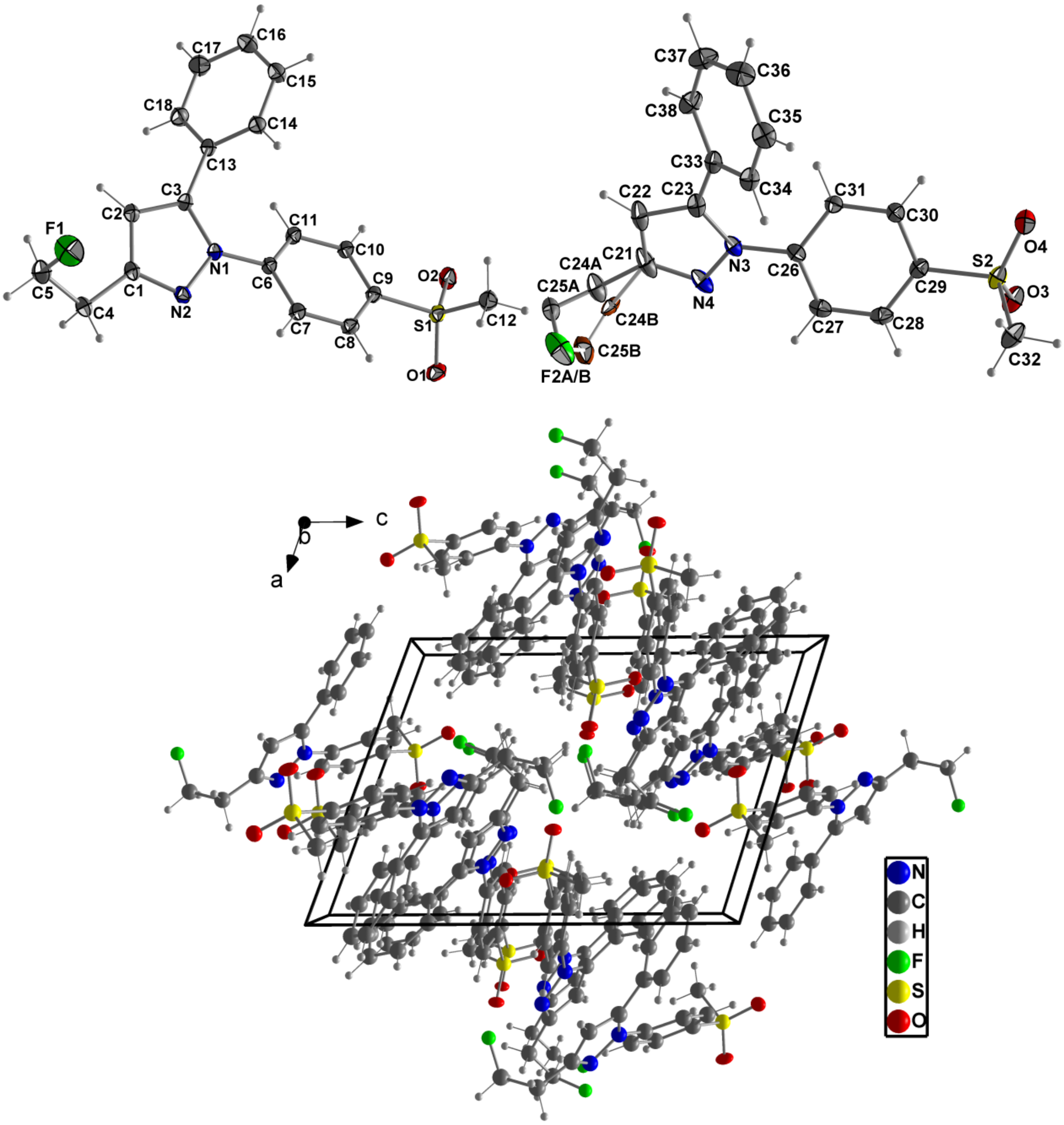

Figure S4. Molecular structures of the two symmetry independent molecules of compound **5c** (top, ORTEP plot with atom labelling scheme, displacement thermal ellipsoids are drawn at the 50% probability level) and crystal packing (bottom) in a view along b.

Table S3. Crystal, structure solution, and refinement data of **5c**

Chemical formula	$C_{18}H_{17}FN_2O_2S$
*M*r	344.39
Crystal system, space group	Triclinic, *P-1*
Temperature (K)	123
a, *b*, *c* (Å)	11.2831(3), 11.9926(3), 14.2709(4)
α, *β*, *γ* (°)	92.663(1), 105.904(1), 115.409(1)
V ($Å^3$)	1647.31(8)
Z	4
Radiation type	Mo*Kα*
μ (mm^{-1})	0.22
Crystal size (mm^3)	0.35 × 0.12 × 0.10
Diffractometer	Bruker-Nonius *APEX*-II CCD
Absorption correction	Multi-scan, Bruker-Nonius *SADABS*
No. of measured, indep. refl.	59598, 16365
observed [$I > 2\sigma(I)$] refl.	13574
*R*int	0.029
$(\sin \theta/\lambda)_{max}$ ($Å^{-1}$)	0.845
$R[F^2 > 2\sigma(F^2)]$, $wR(F^2)$, S	0.039, 0.115, 1.03
No. of reflections	16365
No. of parameters	452
H-atom treatment	H-atom parameters constrained
$\Delta\rho_{max}$, $\Delta\rho_{min}$ (e $Å^{-3}$)	0.63, −0.51

Computer programs: Bruker *APEX2*, Bruker *SAINT*, *SHELXS2014*/1 (Sheldrick, 2014), *SHELXL14* (Sheldrick, 2014).

Table S4. Hydrogen-bond geometry (Å, º)

D—H⋯*A*	*D*—H	H⋯*A*	*D*⋯*A*	*D*—H⋯*A*
C4—H4B⋯O2^{i}	0.99	2.51	3.220(1)	128
C12—H12A⋯F2A^{ii}	0.98	2.57	3.483(1)	155
C12—H12B⋯O1^{ii}	0.98	2.33	3.219(1)	151
C22—H22A⋯O1^{ii}	0.95	2.57	3.330(1)	138
C25A—H25A⋯F2A^{iii}	0.99	2.63	3.341(2)	129
C32—H32A⋯F1^{iv}	0.98	2.54	3.315(1)	136
C32—H32B⋯O3^{iv}	0.98	2.36	3.258(1)	151

Symmetry codes: (i) −*x*+2, −*y*+1, −*z*+1; (ii) −*x*+2, −*y*+2, −*z*+1; (iii) −*x*+1, −*y*+1, −*z*+1; (iv) −*x*+1, −*y*, −*z*.

3. HPLC and TLC of final radiotracers [^{18}F]5a,b, and [D_2,^{18}F]5a

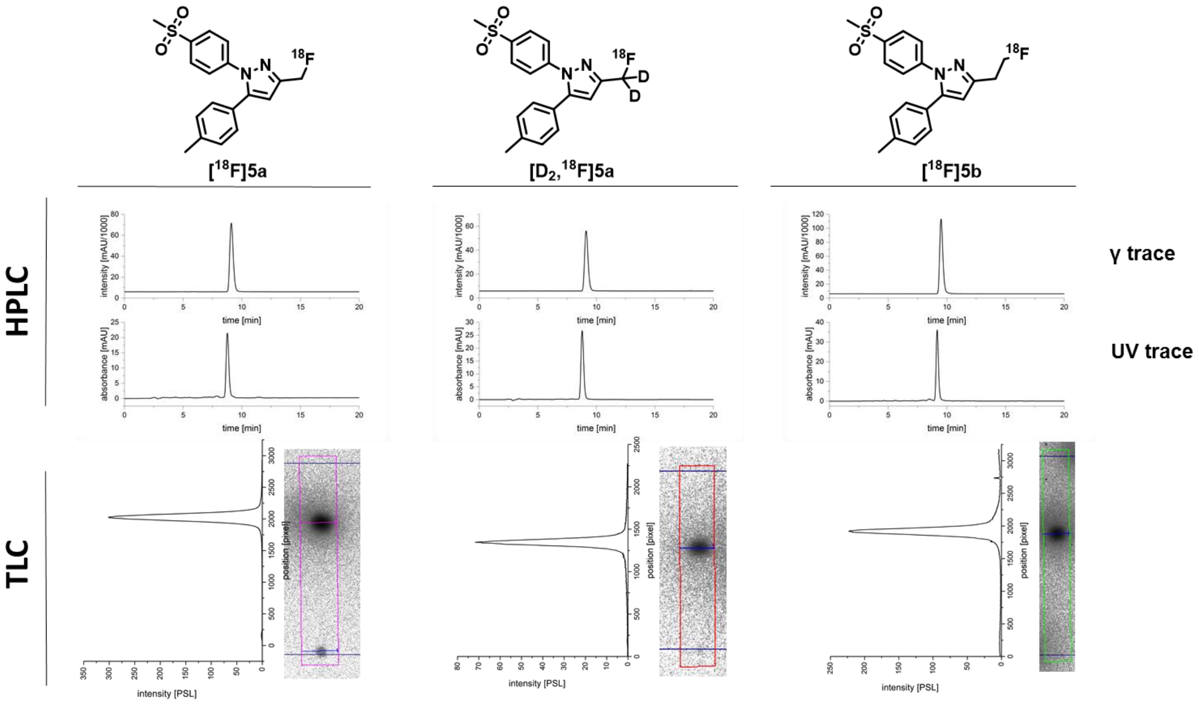

Figure S5. Analytical Radio-HPLC and Radio-TLC chromatograms of the final radiotracers [^{18}F]**5a,b**, and [D_2,^{18}F]**5a**. HPLC conditions: column Luna C18, eluent MeCN/H_2O + 0.1% TFA, 60/40; Radio-TLC, eluent petroleum ether/ethyl acetate, 1/2.

4. Biodistribution data of [^{18}F]5a,b, and [D_2,^{18}F]5a

Table S5. Biodistribution of [^{18}F]**5a**, [D_2,^{18}F]**5a**, and [^{18}F]**5b** in healthy rats at 5 min p.i.; n = 8 for each tracer. Data are given as SUV or %ID as indicated.

5 minutes	[^{18}F]**5a**	[D_2.^{18}F]**5a**	[^{18}F]**5b**
SUV			
blood	0.46 ± 0.15	0.42 ± 0.04	0.32 ± 0.02
heart	0.79 ± 0.23	0.76 ± 0.12	0.86 ± 0.07
liver	3.84 ± 1.52	4.16 ± 0.29	3.37 ± 0.63
kidneys	1.53 ± 0.49	1.58 ± 0.24	2 ± 0.42
adrenals	2.25 ± 1.19	3.28 ± 0.52	2.95 ± 0.73
pancreas	0.97 ± 0.27	1.37 ± 0.13	1.6 ± 0.16
spleen	0.53 ± 0.24	0.56 ± 0.1	0.84 ± 0.12
brain	0.46 ± 0.14	0.54 ± 0.12	0.5 ± 0.1
harderian gland	1.5 ± 0.6	1.94 ± 0.14	2.37 ± 0.19
WAT	1.45 ± 0.53	2.79 ± 1.06	1.73 ± 0.84
BAT	2.21 ± 0.91	3.31 ± 0.56	2.79 ± 0.85
lung	0.89 ± 0.38	0.92 ± 0.14	1.06 ± 0.06
thymus	0.76 ± 0.18	0.95 ± 0.08	1.09 ± 0.23
thyroid gland	0.71 ± 0.4	0.95 ± 0.18	1.01 ± 0.1
testes	0.34 ± 0.16	0.48 ± 0.04	0.47 ± 0.05
skin & hair	0.97 ± 0.31	0.9 ± 0.15	0.98 ± 0.09
muscle	0.35 ± 0.18	0.52 ± 0.08	0.63 ± 0.09
femur	0.96 ± 0.53	0.84 ± 0.09	0.6 ± 0.05
%ID			
intestine	6.78 ± 2.69	10.14 ± 1.96	14.17 ± 1.43
stomach	0.87 ± 0.65	1.14 ± 0.57	1.35 ± 0.6
urine (calc.)	3.32 ± 4.35	0.15 ± 1.91	0.02 ± 1.08

Table S6. Biodistribution of [^{18}F]**5a**, [D$_2$,^{18}F]**5a**, and [^{18}F]**5b** in healthy rats at 60 min p.i.; n = 8 for each tracer. Data are given as SUV or %ID as indicated.

60 minutes	[^{18}F]**5a**	[D$_2$.^{18}F]5**a**	[^{18}F]**5b**
SUV			
blood	0.12 ± 0.04	0.11 ± 0.01	0.08 ± 0.02
heart	0.15 ± 0.02	0.16 ± 0.03	0.18 ± 0.02
liver	0.78 ± 0.2	0.92 ± 0.19	0.74 ± 0.14
kidneys	0.36 ± 0.19	0.47 ± 0.06	0.57 ± 0.14
adrenals	1.85 ± 1.91	0.91 ± 0.15	0.9 ± 0.13
pancreas	0.44 ± 0.28	0.62 ± 0.37	0.57 ± 0.14
spleen	0.18 ± 0.07	0.22 ± 0.12	0.28 ± 0.08
brain	0.12 ± 0.04	0.11 ± 0.02	0.09 ± 0.01
harderian gland	0.64 ± 0.28	0.5 ± 0.08	1.08 ± 0.18
WAT	1.67 ± 0.23	2.4 ± 0.28	1.81 ± 0.8
BAT	1.13 ± 0.29	1.21 ± 0.11	1.12 ± 0.22
lung	0.19 ± 0.06	0.21 ± 0.03	0.25 ± 0.04
thymus	0.28 ± 0.11	0.21 ± 0.04	0.23 ± 0.04
thyroid gland	0.67 ± 0.37	0.45 ± 0.06	0.27 ± 0.06
testes	0.27 ± 0.17	0.21 ± 0.04	0.24 ± 0.06
skin & hair	0.39 ± 0.2	0.25 ± 0.04	0.32 ± 0.09
muscle	0.21 ± 0.1	0.19 ± 0.07	0.23 ± 0.05
femur	4.06 ± 0.58	2.56 ± 0.28	0.99 ± 0.17
%ID			
intestine	19.43 ± 2.27	33.02 ± 3.53	41.52 ± 3.98
stomach	1.06 ± 0.42	1.19 ± 0.49	0.94 ± 0.58
urine (calc.)	8.55 ± 2.53	9.12 ± 3.68	15.85 ± 2.16

5. Copies of radio-HPLC/TLC chromatograms of metabolite studies

[^{18}F]**5a**

A
Blood
0 min p.i.

B
Blood
60 min p.i.

C
Liver
60 min p.i.

D
Urine
60 min p.i.

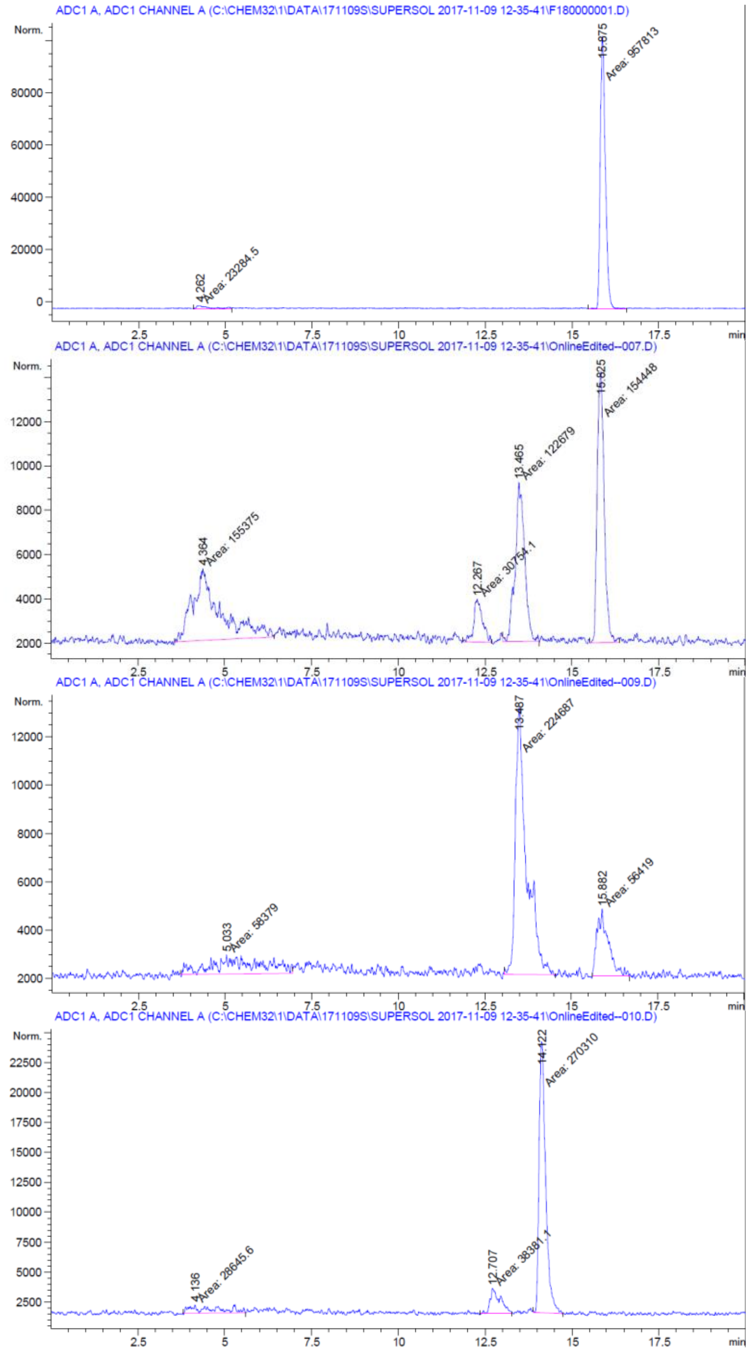

Figure S6. Metabolite analysis of [^{18}F]**5a**. Radio-HPLC chromatograms of blood, liver, and urine at the indicated time p.i. (A-D).

$[D_2,^{18}F]$**5a**

A
Blood
0 min p.i.

B
Blood
60 min p.i.

C
Liver
60 min p.i.

D
Urine
60 min p.i.

E
Intestinal content
60 min p.i.

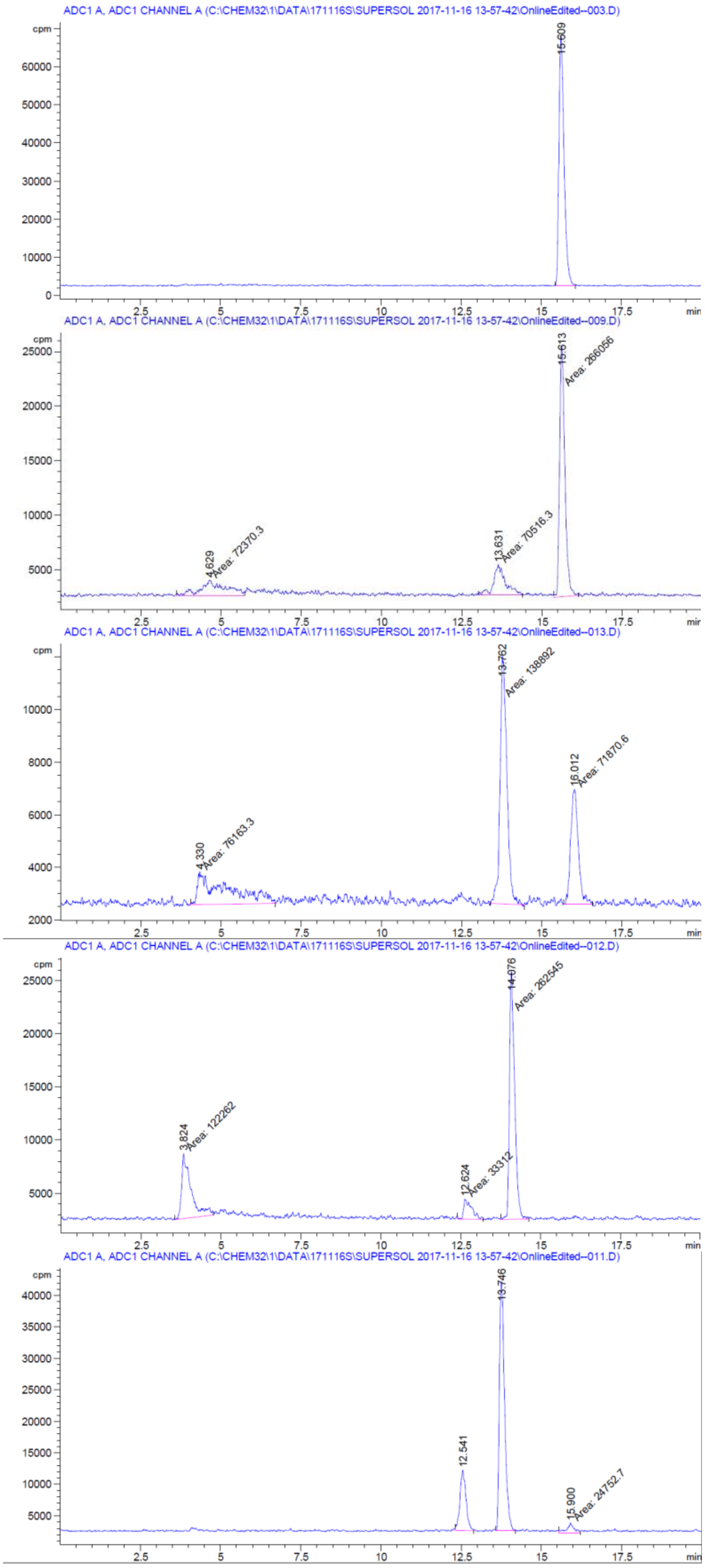

Figure S7. Metabolite analysis of $[D_2,^{18}F]$**5a**. Radio-HPLC chromatograms of blood, liver, urine, and intestinal content at the indicated time p.i. (A-E).

[^{18}F]**5b**

A
Blood
0 min p.i.

B
Blood
60 min p.i.

C
Liver
60 min p.i.

D
Urine
60 min p.i.

E
Intestinal content
60 min p.i.

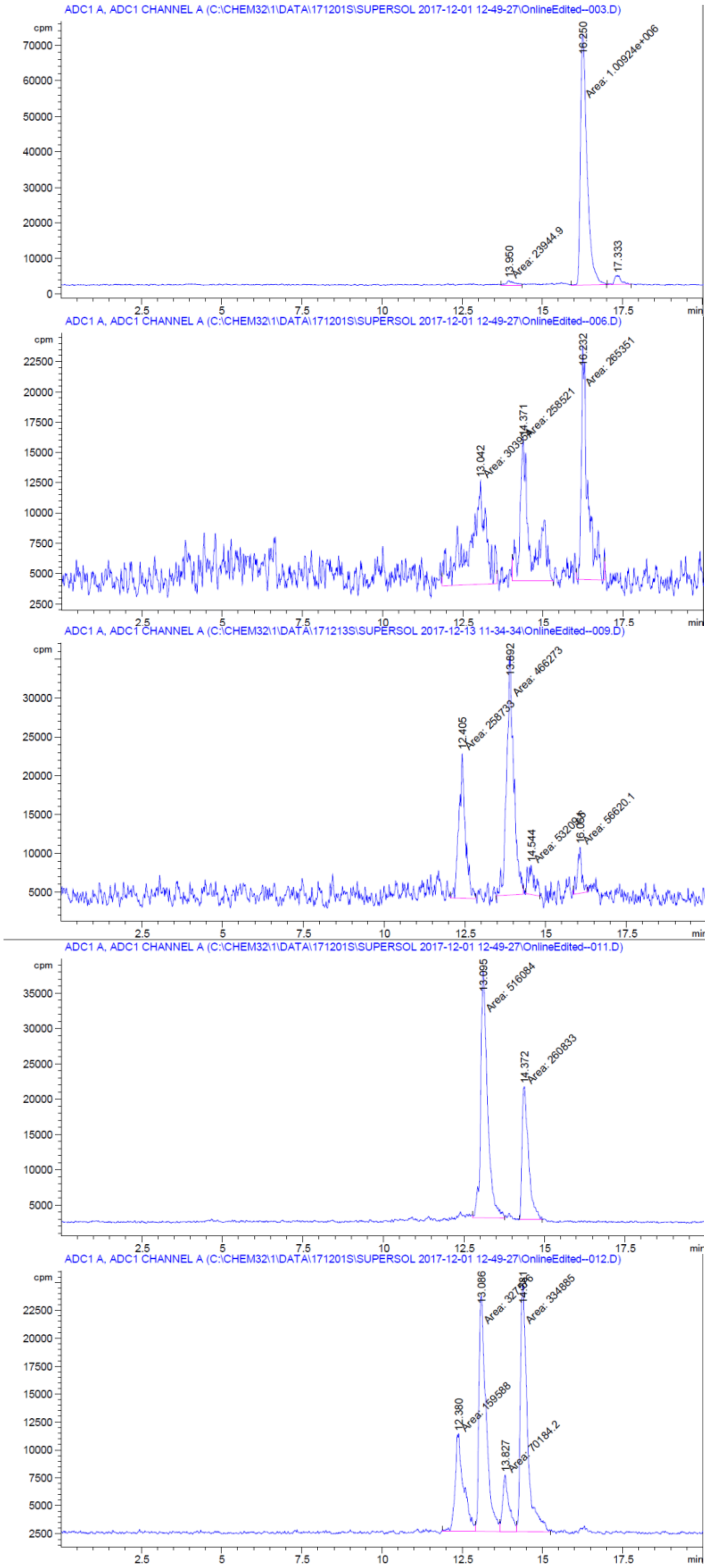

Figure S8. Metabolite analysis of [^{18}F]**5b**. Radio-HPLC chromatograms of blood, liver, urine, and intestinal content at the indicated time p.i. (A-E).

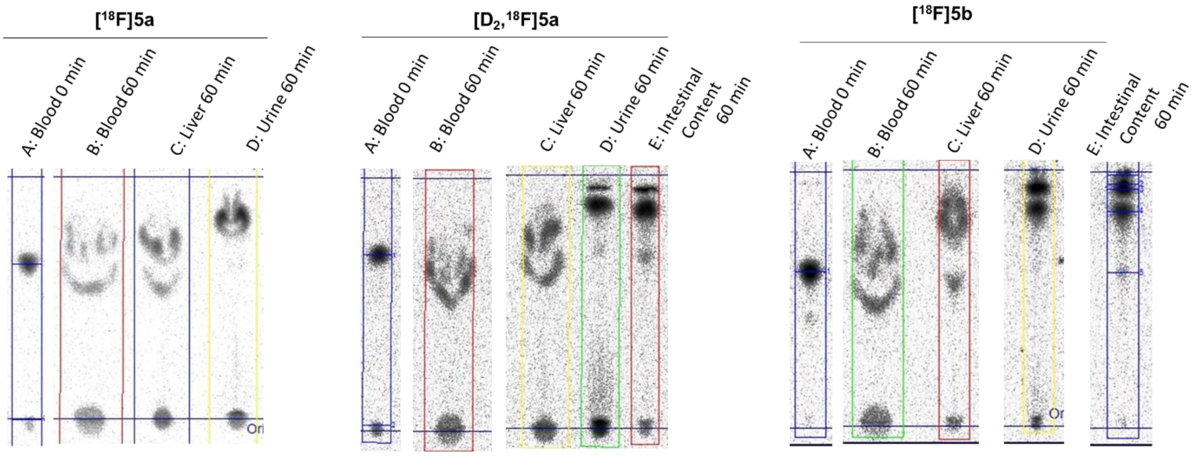

Figure S9. Metabolite analysis of [^{18}F]**5a,b**, and [D_2,^{18}F]**5a**. Radio-TLC chromatograms of blood, liver, urine, and intestinal content at the indicated time p.i. (A-E).

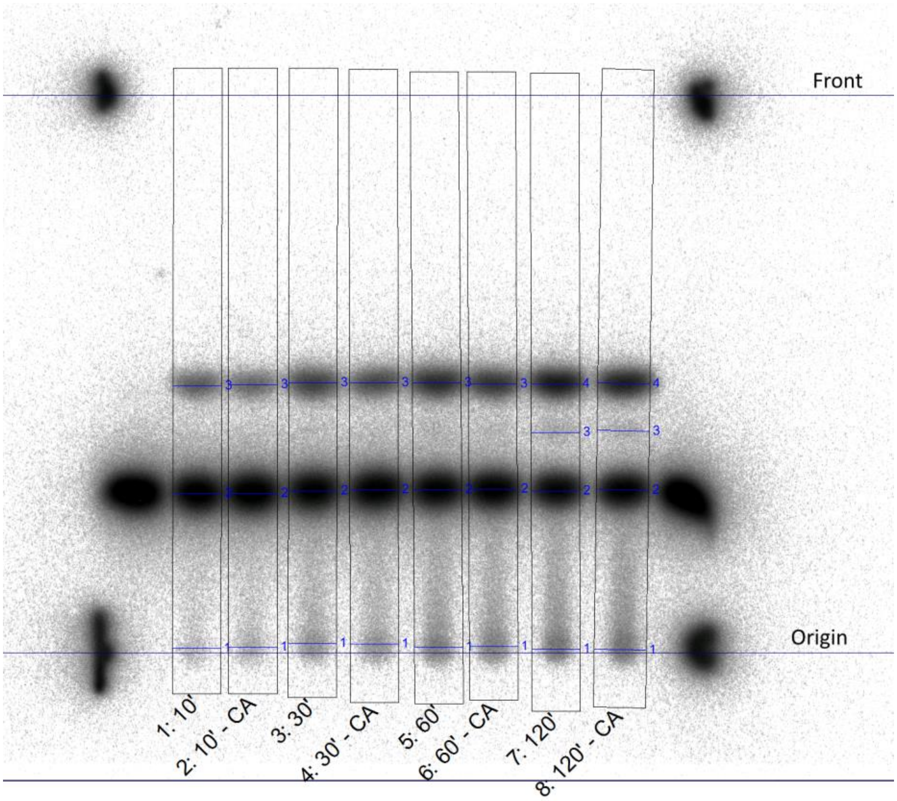

Figure S10. Radio-TLC of [^{18}F]**5a** after incubation with murine liver microsomes for the indicated time (10 – 120 min) with or without carrier addition (CA) using **5a**. The intact radiotracer [^{18}F]**5a** in EtOH was spotted at the outer left and right lane as a reference for the intact radiotracer. Origin and front was spotted with ^{18}F-activity after development of the radio-TLC.

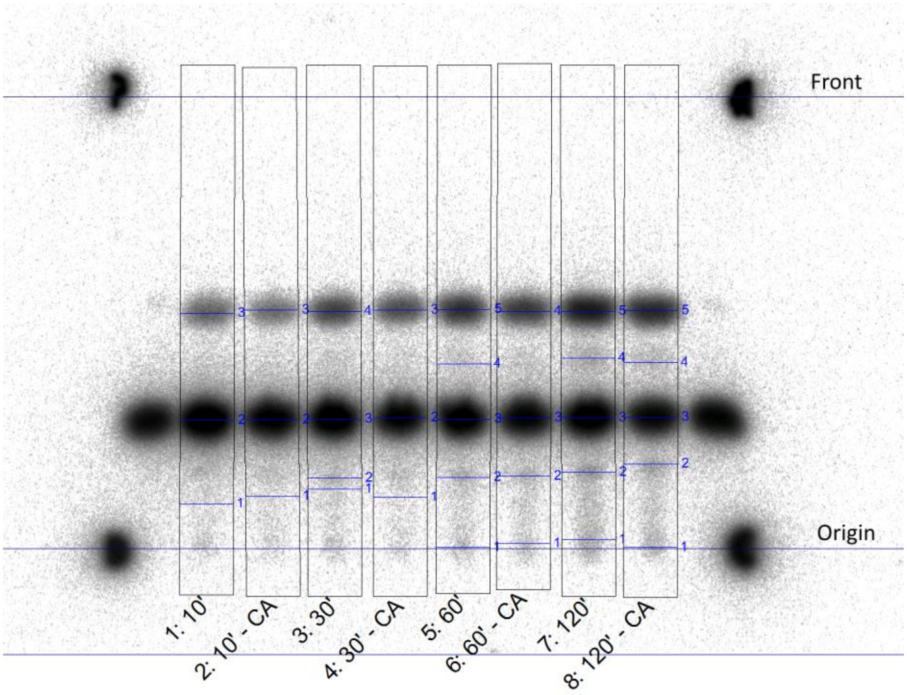

Figure S11. Radio-TLC of [D_2,[18]F]**5a** after incubation with murine liver microsomes for the indicated time (10 – 120 min) with or without carrier addition (CA) using [D_2]**5a**. The intact radiotracer [D_2,[18]F]**5a** in EtOH was spotted at the outer left and right lane as a reference for the intact radiotracer. Origin and front was spotted with ^{18}F-activity after development of the radio-TLC.

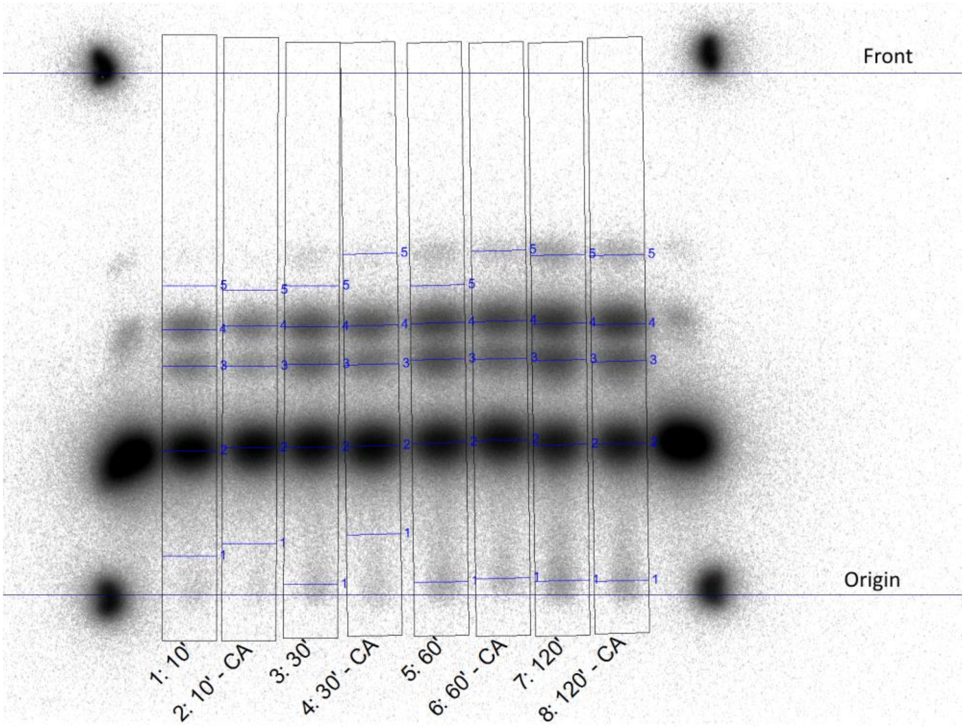

Figure S12. Radio-TLC of [^{18}F]**5b** after incubation with murine liver microsomes for the indicated time (10 – 120 min) with or without carrier addition (CA) using **5b**. The intact radiotracer [^{18}F]**5b** in EtOH was spotted at the outer left and right lane as a reference for the intact radiotracer. Origin and front was spotted with ^{18}F-activity after development of the radio-TLC.

6. Structural elucidation of MLM-metabolites via UPLC-MS/MS

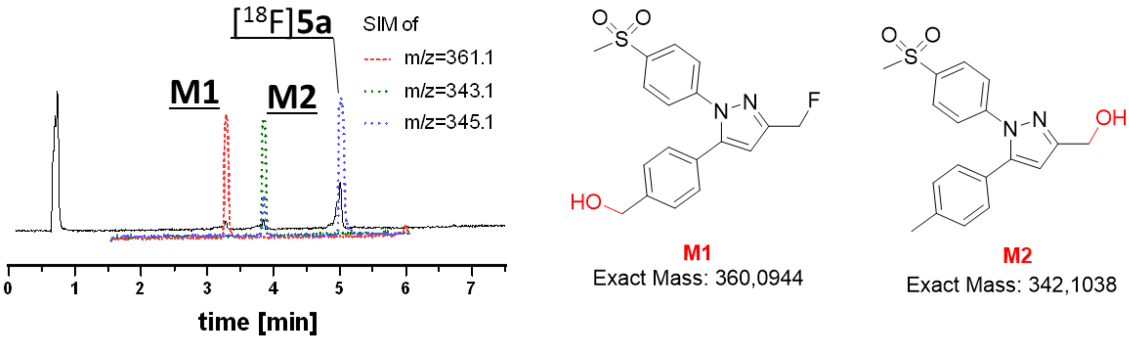

Figure S13. UPLC (λ = 254 nm, black) and SIM (dotted) chromatogram of carrier-added [^{18}F]**5a** after incubation with murine liver microsomes for 60 min and structures of metabolites identified by UPLC-MS/MS. Samples were stored at -20°C between MLM assay and measurement by UPLC-MS/MS.

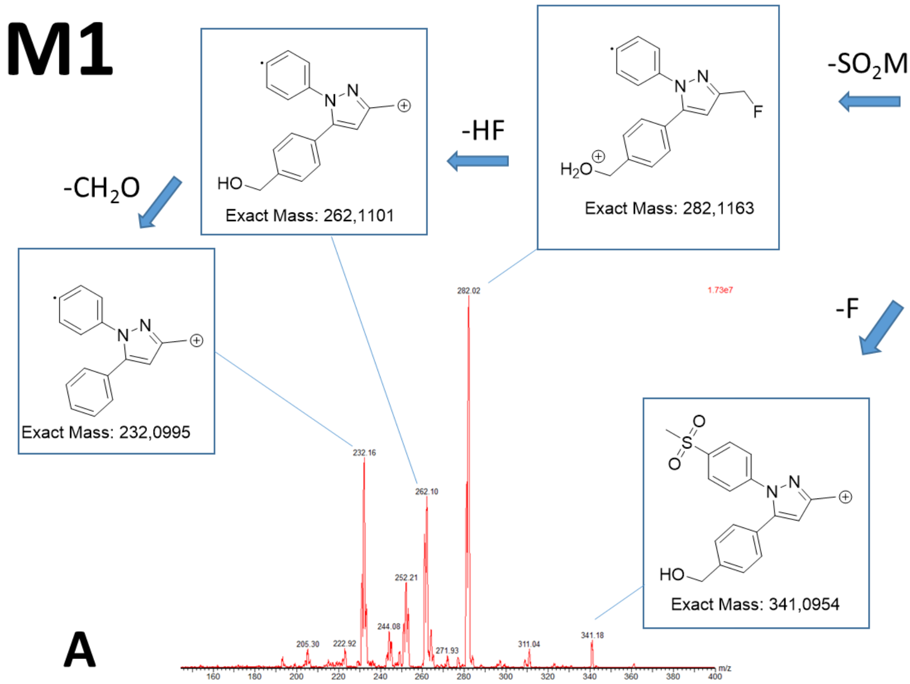

Figure S14. UPLC-MS/MS spectra (daughter scan of m/z = 361 at t_R = 3.245 min, ramp 5-45, representative m/z range between 160 and 400 is shown) and schematic fragmentation pattern of metabolite **M1** originating from carrier-added [^{18}F]**5a** incubation with murine liver microsomes.

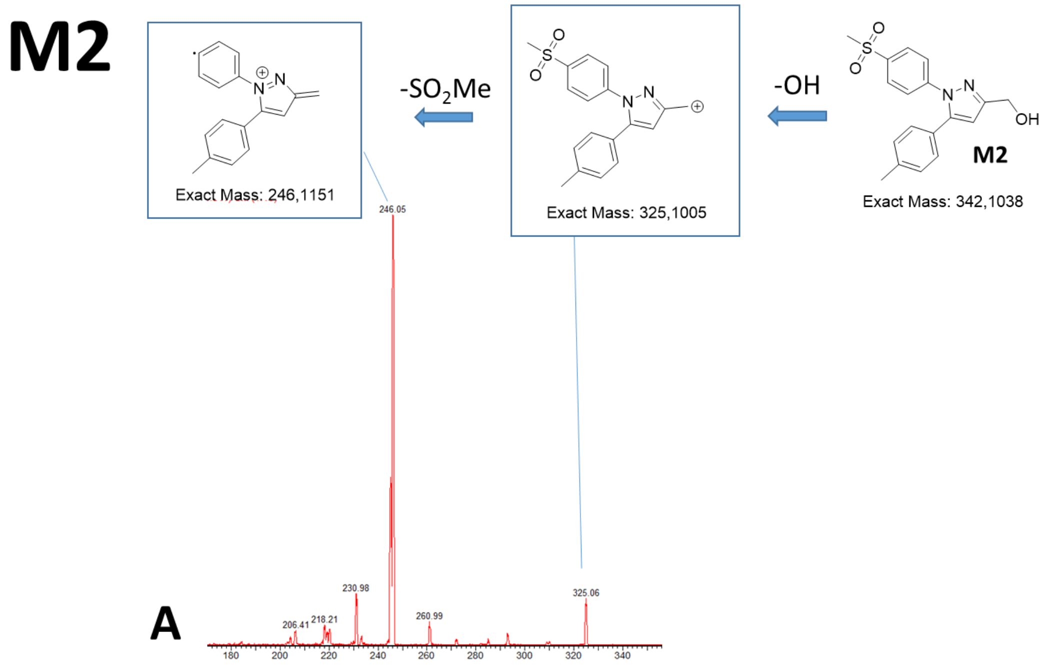

Figure S15. UPLC-MS/MS spectra (daughter scan of m/z = 343 at t_R = 3.811 min, ramp 5-45, representative m/z range between 180 and 340 is shown) and schematic fragmentation pattern of metabolite **M2** originating from carrier-added [^{18}F]**5a** incubation with murine liver microsomes.

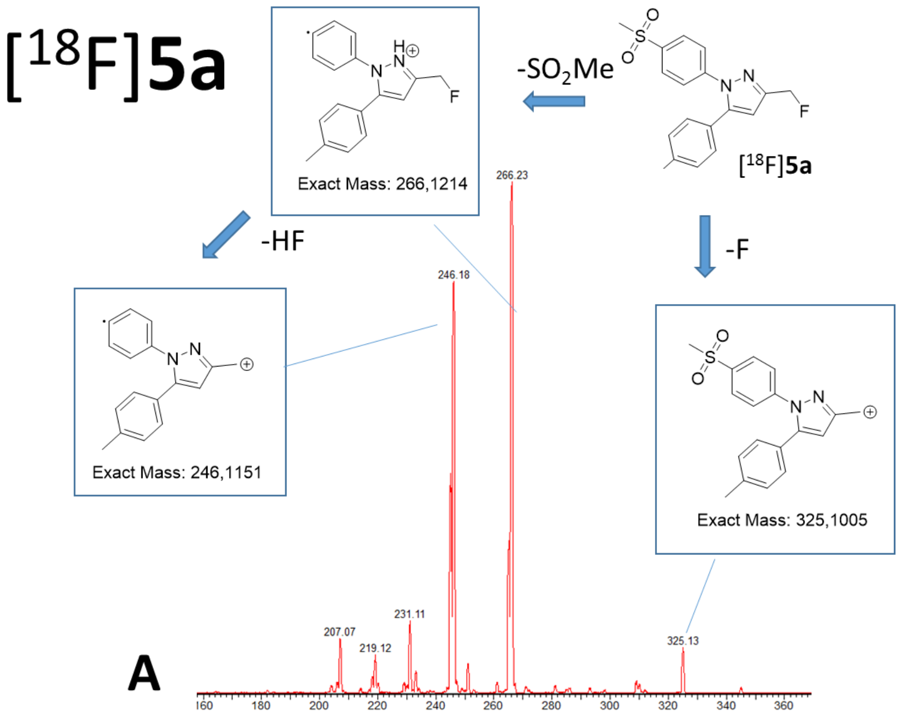

Figure S16. UPLC-MS/MS spectra (daughter scan of m/z = 345 at t_R = 4.982 min, ramp 5-45, representative m/z range between 160 and 360 is shown) and schematic fragmentation pattern of intact carrier-added [^{18}F]**5a** resulting from incubation with murine liver microsomes.

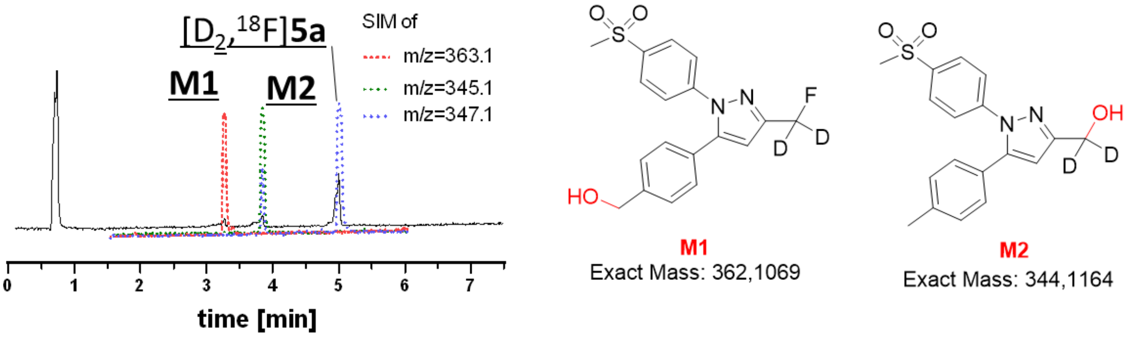

Figure S17. UPLC (λ = 254 nm, black) and SIM (dotted) chromatogram of carrier-added [D_2,^{18}F]**5a** after incubation with murine liver microsomes for 60 min and structures of metabolites identified by UPLC-MS/MS. Samples were stored at -20°C between MLM assay and measurement by UPLC-MS/MS.

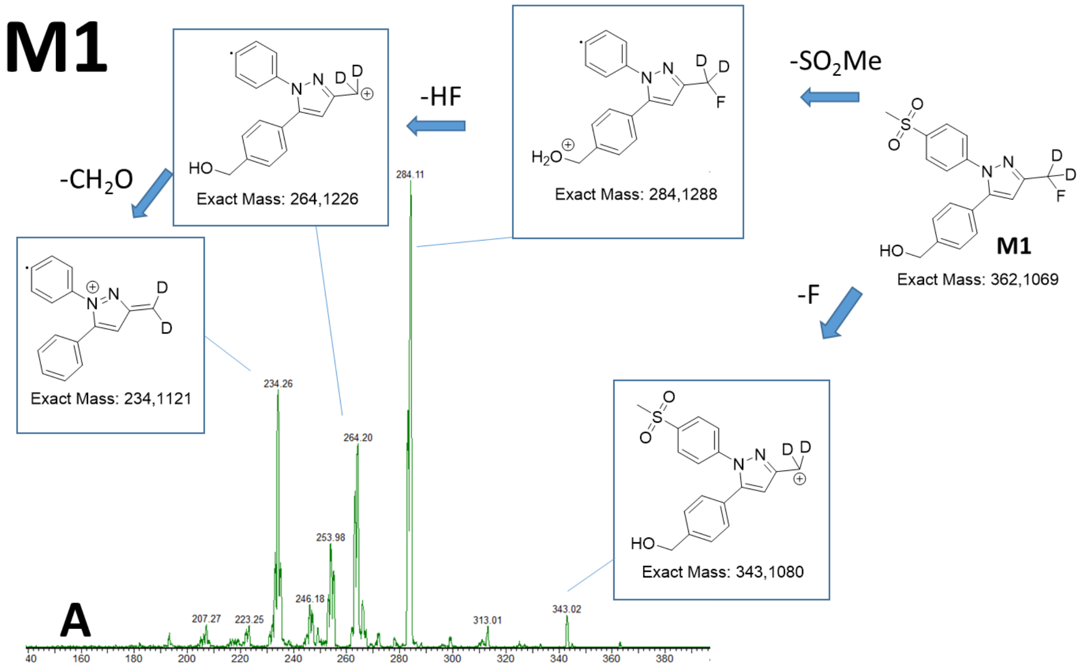

Figure S18. UPLC-MS/MS spectra (daughter scan of m/z = 363 at t_R = 3.228 min, ramp 5-45, representative m/z range between 40 and 380 is shown) and schematic fragmentation pattern of metabolite **M1** originating from carrier-added [D_2,^{18}F]**5a** incubation with murine liver microsomes.

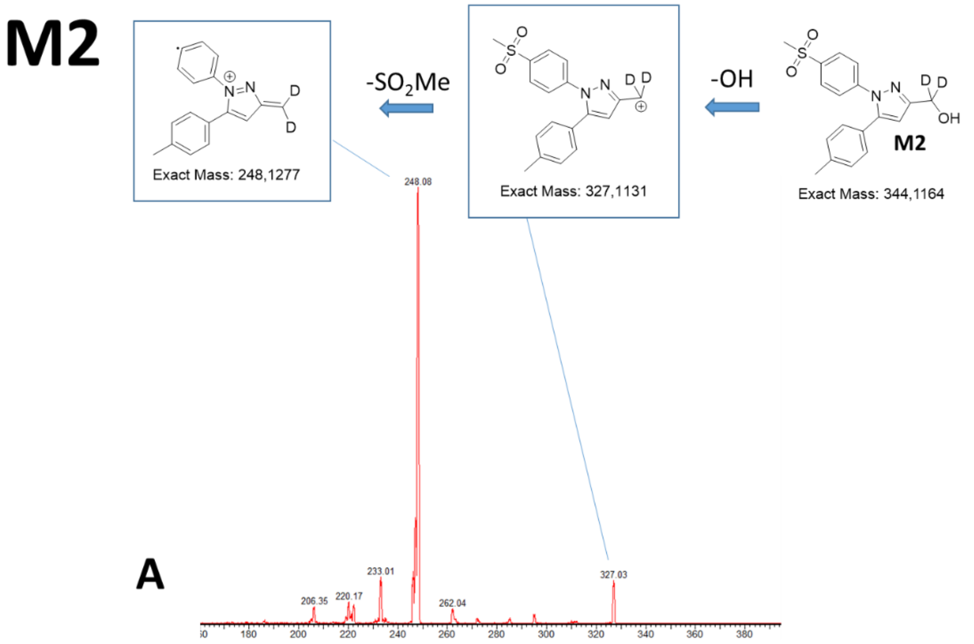

Figure S19. UPLC-MS/MS spectra (daughter scan of m/z = 345 at t_R = 3.804 min, ramp 5-45, representative m/z range between 180 and 380 is shown) and schematic fragmentation pattern of metabolite **M2** originating from carrier-added [D_2,^{18}F]**5a** incubation with murine liver microsomes.

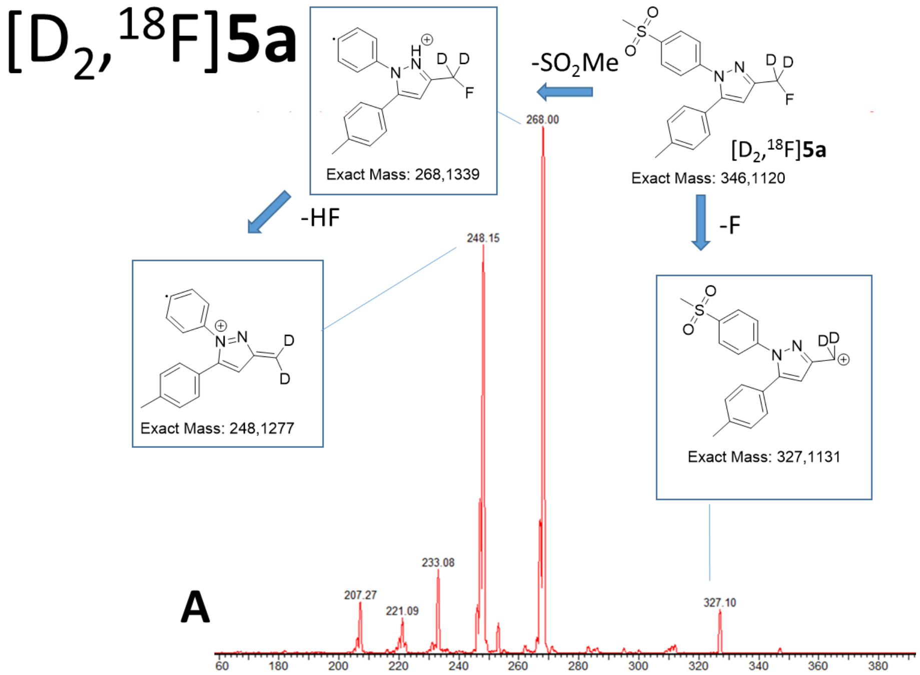

Figure S20. UPLC-MS/MS spectra (daughter scan of m/z = 347 at t_R = 4.964 min, ramp 5-45, representative m/z range between 180 and 380 is shown) and schematic fragmentation pattern of intact carrier-added [D_2,^{18}F]**5a** resulting from incubation with murine liver microsomes.

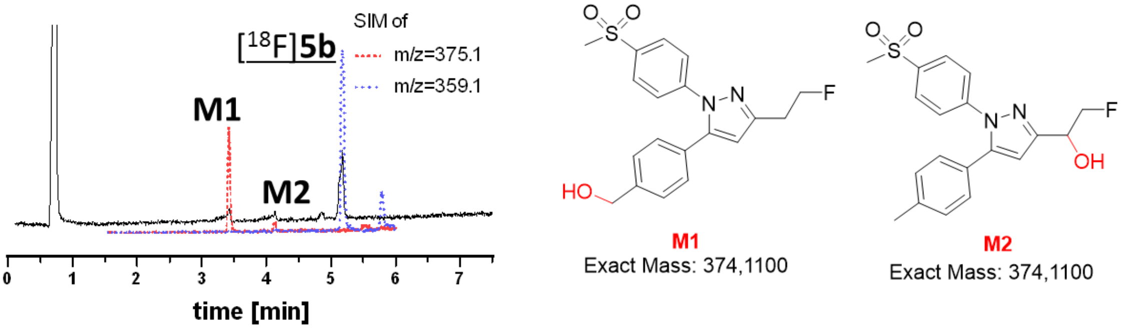

Figure S21. UPLC (λ = 254 nm, black) and SIM (dotted) chromatogram of carrier-added [18F]**5b** incubation after incubation with murine liver microsomes for 60 min and structures of metabolites identified by UPLC-MS/MS. Samples were stored at -20°C between MLM assay and measurement by UPLC-MS/MS.

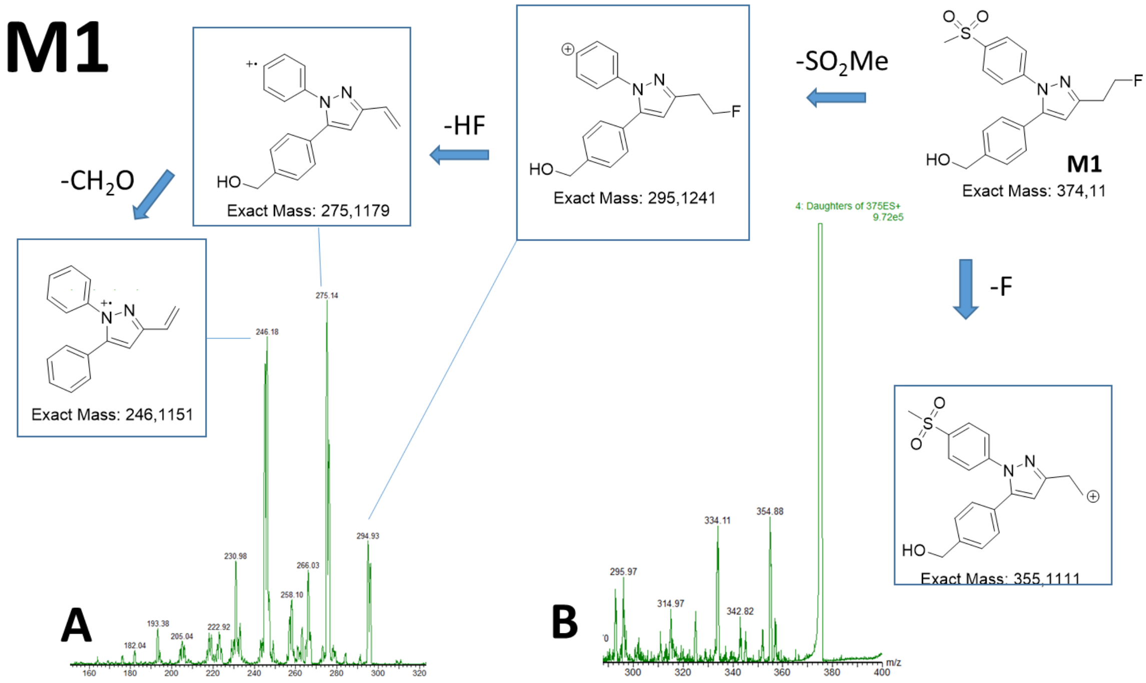

Figure S22. UPLC-MS/MS spectra (daughter scan of m/z = 375 at t_R = 3.338 min, (A) ramp 5-45, representative m/z range between 160 and 320 is shown, (B) constant cone voltage, representative m/z range between 300 and 400 is shown) and schematic fragmentation pattern of metabolite **M1** originating from carrier-added [18F]**5b** incubation with murine liver microsomes.

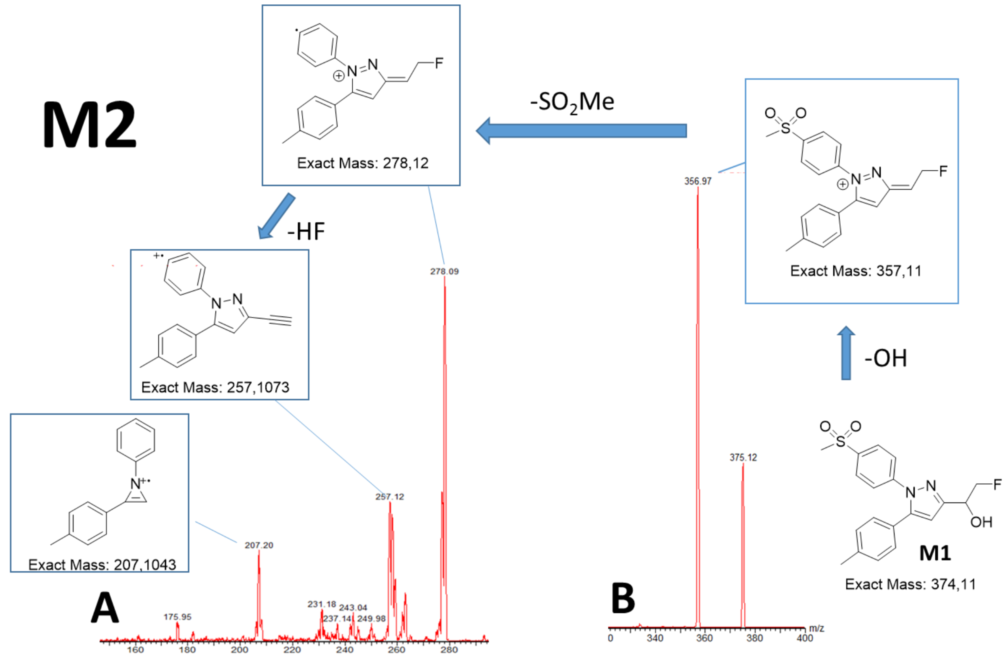

Figure S23. UPLC-MS/MS spectra (daughter scan of m/z = 375 at t_R = 4.094 min, (A) ramp 5-45, representative m/z range between 160 and 280 is shown, (B) constant cone voltage, representative m/z range between 340 and 400 is shown)) and schematic fragmentation pattern of metabolite **M2** originating from carrier-added [^{18}F]**5b** incubation with murine liver microsomes.

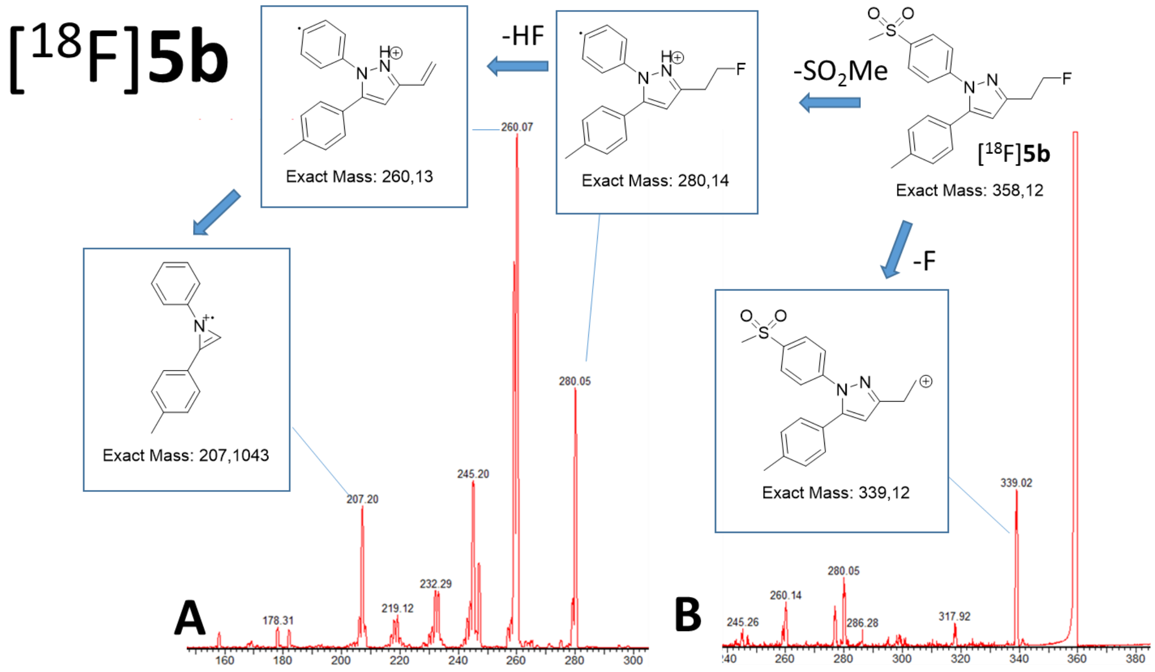

Figure S24. UPLC-MS/MS spectra (daughter scan of m/z = 359 at t_R = 5.150 min, (A) ramp 5-45, representative m/z range between 160 and 300 is shown, (B) constant cone voltage, representative m/z range between 260 and 380 is shown)) and schematic fragmentation pattern of intact carrier-added [^{18}F]**5b** resulting from incubation with murine liver microsomes.

7. Experimental Section

7.1. General

All commercial reagents and solvents were used without further purification. General procedures A-E followed the synthetic strategy published by Uddin *et al.*[1] with minor modifications. Synthesis of starting materials **1a**[1] and **1b**[2] were performed as described in the literature and analytical data were in accordance with literature findings. Flash chromatography was conducted using silica gel (mesh size 40−63 µm). DCVC indicates the use of "dry column vacuum chromatography" as reported by Pedersen and Rosenbohm.[3] Thin-layer chromatography (TLC) was performed on silica gel F-254 aluminum plates (Merck TLC silica gel 60 F_{254}, 1.05554.0001). Visualization was carried out using UV (254 nm/366 nm). Analytical HPLC was performed with the following systems: (*System 1*) C_{18} column (Luna, Phenomenex, 5 µm, 250 x 4.6 mm) using Agilent 1200 HPLC: pump G1311A, auto sampler G1329A, column oven G1316A, degasser G1322A, UV detector G1315D, γ detector Gabi Star®; flow rate = 1 mL/min, isocratic eluent: MeCN/H_2O + 0.1% TFA 80/20 (Iso 1) or 70/30 (Iso 2) or 60/40 (Iso 3) or 50/50 (Iso 4) (*v/v*)); (*System 2*) column Kinetex C-18 (Phenomenex 50 x 2.1 mm, 1.7 µm, 100 Å), Shimadzu Nexera X2 UHPLC system (Kyoto, Japan; degasser DGU-$20A_{3R}$ and DGU-$20A_{5R}$, pump LC-30AD, autosampler SIL-30AC, column oven CTO-20AC with two column switching valves FCV-14AH, diode array detector SPD-M30A, γ detector Gabi Star (Raytest, Straubenhardt, Germany), communication bus module CBM-20A), eluent: (A): MeCN, (B): 0.1% trifluoroacetic acid in H_2O; flow rate 0.5 mL/min), gradient: $t_{0\ min}$ 25/75 - $t_{0.3\ min}$ 25/75 - $t_{4.0\ min}$ 50/50 - $t_{4.5\ min}$ 95/5 - $t_{5.5\ min}$ 95/5 - $t_{6.0\ min}$ 25/75 – $t_{7.5\ min}$ 25/75; (*System 3*) waters UPLC *I*-Class (Milford, Massachusetts, USA; binary gradient pump BSM, autosampler FTN, column manager CM, and diode array detector PDAeλ coupled to Waters Xevo TQ-S), column Aquity UPLC® BEH C_{18} column (waters, 100 x 2.1 mm, 1.7 µm, 130 Å), eluent: (A): 0.1% acetic acid in MeCN/MeOH 1/1/(B): 0.1% acetic acid in H_2O; flow rate 0.4 mL/min, gradient: $t_{0\ min}$ 25/75 - $t_{0.5\ min}$ 25/75 - $t_{5.5\ min}$ 75/25 – $t_{6.0\ min}$ 95/5 - $t_{7.0\ min}$ 95/5 - $t_{8.0\ min}$ 25/75 – $t_{8.5\ min}$ 25/75). The products were monitored at λ = 254 nm and in case of radio-HPLC using additionally the γ detector unless otherwise specified. Purity of all compounds exceeded 95% as determined by analytical HPLC analysis unless otherwise stated. Low resolution mass spectra were obtained using electrospray and ASAP (atmospheric solids analysis probe) ionization on a Waters Xevo TQ-S coupled to a UPLC I-Class (*system 3*). High resolution mass spectra were obtained on a Bruker Daltonics MicrOTOF ESI-TOF-MS using electrospray ionization and analyzed using Bruker Compass DataAnalysis software (ver. 4.2). Elemental (C, H, N, S) analyses were carried out on an elemental analyzer (EuroEA3000, Eurovector). Melting points are uncorrected and were determined on a melting points apparatus (Cambridge Instruments, GalenTM III, Testotherm testo 700; heater: Leica) or, if indicated, on an automated system (Stanford Research Systems, MPA100

OptiMelt, heating rate 1 °C/min). Nuclear magnetic resonance spectra were recorded on a 400 MHz (Varian, Unity INOVA 400 MHz) or 600 MHz (Varian VNMRS 600 MHz) spectrometer. NMR spectra were referenced to the residual solvent shifts for ^{1}H and ^{13}C, and to $CFCl_3$ for ^{19}F spectra as internal standard and HSQC, HMBC, NOESY, ROESY spectra were recorded for assignment. ^{13}C NMR chemical shifts were obtained from broadband proton decoupled spectra. Chemical shifts (δ) are reported in parts per million (ppm). *J* values are given in Hz and, unless otherwise stated, are given for the coupling between two hydrogen atoms. For assignment, the numbering scheme follows the nomenclature of the compounds IUPAC name. In ^{1}H NMR for example $H_{SO2\text{-}Ph}$ refers to the protons of the methylsulfonyl-substituted phenyl ring, $H_{tolyl\ H2/H6}$ refers to the protons at position 2 and 6 of the tolyl ring.

No-carrier-added aqueous [^{18}F]fluoride was produced in a CYCLONE 18/9® cyclotron (IBA, Belgium) or TR-FLEX 18-30 MeV (ACSI, Richmond/Vancouver, Canada) by irradiation of [^{18}O]H_2O via the $^{18}O(p,n)^{18}F$ nuclear reaction. Radio-thin layer chromatography was performed as given above, visualized using a Fuji BAS 2000® scanner system and analyzed using advanced image data analyzer (AIDA) software (Version 5.1 SP4, Raytest, Straubenhardt, Germany). Radiochemical yields (RCY) are decay-corrected. For SPE-based purification the following cartridges were used; Sep-Pak light Accell Plus QMA® (130 mg, Part. Nr. WAT023525), Chromafix C_{18} ec (s) (Macherey-Nagel Part Nr. 731804). Radiosyntheses were performed using two identical automated radiosynthesizers TRACERlab FX-N only differing in their integrated semi-preparative HPLC systems (*System 4 & 5*): (*System 4*) Jasco pump PU-1580, degasser DG-2080-53, UV detector UV-1575 (detection at 254 nm), (*System 5*) S1122 Solvent Delivery System (Synkam), degasser DG-1310 (DEGASYS), UV detector K-2001 (Knauer); γ detector of both systems integrated in the synthesizer module (GE). For both systems, semi-preparative HPLC was performed on a C_{18} column (Discovery® HS C_{18}, 250 x 10 mm, 5 µm, Supelco; flow rate = 4 mL/min, isocratic eluent: MeCN/H2O + 0.1% TFA 40/60 (*v/v*)). Analytical radio-HPLC was carried out on the analytical Agilent 1200 HPLC (*system 1*).

X-ray Crystallography. The crystallographic data were collected using CCD detector based X-ray diffractometers, with MoK*α* radiation (λ = 0.71073 Å). The structures were solved using SHELXS-14 and refined against F^2 on all data by full-matrix least squares with SHELXL-14.[4,5] All non-hydrogen atoms were refined anisotropically. The hydrogen atoms bonded to carbon atoms were placed on geometrically calculated positions and refined isotrocially (**2e**) or using a riding model (**5c**). The asymmetric unit of **5c** consists of two independent molecules, which are identical besides some different dihedral angles along single bonds. One of these two molecules has a disordered C_2H_5F group which was refined using a split model with the sum of the occupational factors of the two parts being fixed to total occupancy. Full crystallographic data for compounds **2e** and **5c** were deposited with the

Cambridge Crystallographic Data Center: CCDC-1428720 (compound **2e**), CCDC 1433314 (compound **5c**). Copies can be obtained, free of charge, on application to CCDC, 12 Union Road, Cambridge CB2 1EZ, UK, (fax: +44(0)1223 336033 or e-mail: deposit@ccdc.cam.ac.uk).

7.2. Chemical syntheses

Methyl (*Z*)-5-hydroxy-3-oxo-5-(*p*-tolyl)pent-4-enoate (1c):The synthesis was performed in analogy to the procedure described by Weber *et al.*[2] with minor modifications and analytical data were in accordance with data published by Rahn *et al.*[6] Under nitrogen atmosphere, *n*-butyllithium (2.4 M in hexane, 10.4 mL, 25 mmol, 2.5 equiv) was added to a solution of diisopropylamine (3523 µL, 25 mmol, 2.5 equiv) in THF (10 mL) at 0 °C followed by stirring for 1 h at this temperature. Then, *N,N,N′,N′*-tetramethylethylenediamine (1.5 mL, 10 mmol, 1.0 equiv) and methyl acetoacetate (1080 µL, 10 mmol, 1.0 equiv) in THF (10 mL) was added. After stirring of the mixture 15 min at 0 °C, methyl 4-methylbenzoate (1878 mg, 12.5 mmol, 1.25 equiv) in THF (15 mL) was added and the mixture was stirred at 4 °C in a cold room. After stirring for 3 days, the reaction was allowed to warm up to room temperature, quenched with HCl (9.5 M, 25 mL), and the mixture was extracted with ethyl acetate (3 x 25 mL). The combined organic phase was dried over Na_2SO_4, filtered, and adsorbed on silica gel. After column chromatographic purification (petroleum ether/EtOAc, 90/10 → 80/20), compound **1c** was isolated as an orange oil (673 mg, 29%): R_f (petroleum ether/ EtOAc 80/20) = 0.23; ^{1}H NMR (400 MHz, $CDCl_3$): δ 2.41 (s, 3H, CH_3), 3.48 (s, 2H, CH_2), 3.77 (s, 3H, $COOCH_3$), 6.26 (s, 1H, CH), 7.26 (d, ^3J 8.0, 2H, H_{tolyl}), 7.78 (d, ^3J 8.3, 2H, H_{tolyl}), OH Signal is not resolved in this spectra; ^{13}C NMR (101 MHz, $CDCl_3$): δ 21.8 (CH_3), 45.7 (CH_2), 52.6 (COO$\underline{C}H_3$), 96.5 (CH), 127.3 (2CH, C_{tolyl}), 129.6 (2CH_{tolyl}), 131.5 (C), 143.7 (C), 168.2 (C), 183.2 (C), 188.5 (CO); MS (ASAP$^+$): *m/z* (%) = 234 (19) [*M*]$^+$, 174 (100) [*M*-$COHOCH_3$]$^+$, 161 (57) [*M*-CH_2COOCH_3]$^+$; HPLC: t_R = 6.03 min (broad, 98.4%, Iso 2).

General procedure A

4-Methylsulfonylphenylhydrazine hydrochloride (1079 mg, 4.85 mmol, 1.1 equiv) was added to a solution of β-dicarbonyl compounds **1a-c** (4.41 mmol, 1.0 equiv) in methanol (30 mL). The mixture was heated at 70 °C for 17-21 h. After cooling to room temperature, the crude product was adsorbed on silica gel and purified by column chromatography yielding the pyrazoles **2a-e** as given below.

Methyl 1-[4-(methylsulfonyl)phenyl]-5-(4-tolyl)-1*H*-pyrazole-3-carboxylate (2a): Starting from methyl (*Z*)-4-hydroxy-2-oxo-4-(*p*-tolyl)but-3-enoate (**1a**, 4.50 g, 18.0 mmol, 1.00 equiv) and 4-(methylsulfonyl)phenylhydrazine hydrochloride (4.25 g, 18.0 mmol, 1.00 equiv) following general

procedure A, **2a** was obtained as a pale pink solid (crude product, 6.41 g, 96%): mp: 178-180 °C; R_f (petroleum ether/ EtOAc 60/40) = 0.21; ^{1}H NMR (400 MHz, DMSO-d_6): *δ* 2.31 (s, 3 H, $CH_{3\ tolyl}$), 3.28 (s, 3 H, SO_2CH_3), 3.87 (s, 3 H, $COOCH_3$), 7.13 (s, 1 H, $CH_{pyrazole}$), 7.19 (d, ^3J 8.4, 2 H, CH_{tolyl}), 7.22 (d, ^3J 8.3, 2 H, CH_{tolyl}), 7.59 (d, ^3J 8.6, 2 H, $CH_{SO2\text{-}phenyl}$), 8.00 (d, ^3J 8.6, 2 H, $CH_{SO2\text{-}phenyl}$), aromatic impurity detected at 7.06 and 7.80 (17 mol% relative to aromatic CH_{tolyl}); ^{13}C NMR (101 MHz, DMSO-d_6): *δ* 20.8, 43.3, 51.9, 110.1, 125.7, 126.0, 128.2, 128.7, 129.4, 138.9, 140.3, 142.9, 144.0, 144.8, 161.8; MS (ESI$^+$, M calc. for $C_{19}H_{18}N_2O_4S$ = 370.10) *m/z* (%): 339.1 (100) [*M*-OH]$^+$, 371.1 (72) [*M*+H]$^+$; HPLC: t_R = 5.56 min (94%, *system 1*); HRMS (ESI/QTOF) m/z: [M+H]$^+$ Calc. for $C_{19}H_{19}N_2O_4S$ 371.1060, Found 371.1063.

Methyl 2-{1-[4-(methylsulfonyl)phenyl]-5-(*p*-tolyl)-1*H*-pyrazol-3-yl}acetate (2b) and methyl 2-{1-[4-(methylsulfonyl)phenyl]-3-(*p*-tolyl)-1*H*-pyrazol-5-yl}acetate (2d): Starting from methyl (*Z*)-5-hydroxy-3-oxo-5-(*p*-tolyl)pent-4-enoate (**1b**, 820 mg, 3.5 mmol, 1.0 equiv) and 4-methylsulfonylphenylhydrazin hydrochloride (860 mg, 3.86 mmol, 1.1 equiv) following general procedure A and column chromatographic purification (chloroform/methanol, 500/1 → 100/1), the 1,5-diaryl-substituted main product **2b** was isolated as a colorless solid (705 mg, 47%): mp: 146.4-148.3 °C (Optimelt)*;* R_f (chloroform/methanol, 100/1) = 0.30; ^{1}H NMR (400 MHz, $CDCl_3$): *δ* 2.37 (s, 3H, $CH_{3\ tolyl}$), 3.04 (s, 3H, SO_2CH_3), 3.76 (s, 3H, $COOCH_3$), 3.79 (s, 2H, CH_2), 6.51 (s, 1H, $CH_{pyrazol\ H4}$), 7.12 (d, ^3J 8.3, 2H, $H_{tolyl\ H2/H6}$), 7.16 (d, ^3J 8.4, 2H, $H_{tolyl\ H3/H5}$), 7.49 (d, ^3J 8.6, 2H, $H_{SO2\text{-}Ph\ H2/H6}$), 7.87 (d, ^3J 8.7, 2H, $H_{SO2\text{-}Ph\ H3/H5}$); ^{13}C NMR (101 MHz, $CDCl_3$): *δ* 21.4 ($CH_{3\ tolyl}$), 34.3 (CH_2), 44.7 (SO_2CH_3), 52.4 (COO<u>C</u>H$_3$), 109.3 ($CH_{pyrazol\ H4}$), 125.1 ($2CH_{SO2\text{-}Ph\ C2/C6}$), 127.1 ($C_{tolyl\ C1}$), 128.4 ($2CH_{SO2\text{-}Ph\ C3/C5}$), 128.8 ($2CH_{tolyl\ C2/C6}$), 129.7 ($2CH_{tolyl\ C3/C5}$), 138.5 ($C_{SO2\text{-}Ph\ C4}$), 139.2 ($C_{tolyl\ C4}$), 144.3 ($C_{SO2\text{-}Ph\ C1}$), 144.8 ($C_{pyrazol\ C5}$), 147.7 ($C_{pyrazol\ C3}$), 171.0 (<u>C</u>OOCH$_3$); MS (ASAP$^+$): *m/z* (%) = 385 (100) [*M*+H]$^+$, 384 (25) [*M*]$^+$; t_R (HPLC; λ_{ref} 360 nm): 5.25 min (100%, Iso 2, *system 1*); HRMS (ESI/micrOTOF) m/z: [M + Na]$^+$ Calc. for $C_{20}H_{20}N_2O_4SNa$ 407.1036; Found 407.1043. The 1,3-diaryl-substituted side product **2d** was isolated as a beige solid (234 mg, 17%): mp: 119-122 °C; R_f (chloroform/methanol, 100/1) = 0.46; ^{1}H NMR (400 MHz, $CDCl_3$): *δ* 2.39 (s, 3H, $CH_{3\ tolyl}$), 3.10 (s, 3H, SO_2CH_3), 3.73 (s, 3H, $COOCH_3$), 3.82 (s, 2H, CH_2), 6.75 (s, 1H, $CH_{pyrazol\ H4}$), 7.23 (d, ^3J 8.2, 2H, $H_{tolyl\ H3/H5}$), 7.74 (d, ^3J 8.1, 2H, $H_{tolyl\ H2/H6}$), 7.79 (d, ^3J 8.8, 2H, $H_{SO2\text{-}Ph\ H2/H6}$), 8.08 (d, ^3J 8.8, 2H, $H_{SO2\text{-}Ph\ H3/H5}$); ^{13}C NMR (101 MHz, $CDCl_3$): *δ* 21.5 ($CH_{3\ tolyl}$), 32.4 (CH_2), 44.8 (SO_2CH_3), 52.8 (COO<u>C</u>H$_3$), 106.8 ($CH_{pyrazol\ C4}$), 125.6 ($2CH_{SO2\text{-}Ph\ C2/C6}$), 125.9 ($2CH_{tolyl\ C2/C6}$), 129.0 ($2CH_{SO2\text{-}Ph\ C3/C5}$), 129.5 ($2CH_{tolyl\ C3/C5}$), 129.6 ($C_{tolyl\ C1}$), 136.9 ($C_{pyrazol\ C5}$), 138.5 ($C_{tolyl\ C4}$), 139.6 ($C_{SO2\text{-}Ph\ C4}$), 144.0 ($C_{SO2\text{-}Ph\ C1}$), 153.2 ($C_{pyrazol\ C3}$), 169.5 (<u>C</u>OOCH$_3$); MS (ASAP$^+$): *m/z* (%) = 385 (100) [*M*+H]$^+$, 384 (42) [*M*]$^+$; elemental analysis calc. for $C_{20}H_{20}N_2O_4S$ (384.45): C: 62.5, H: 5.2, N: 7.3, S: 8.3%, Found: C: 62.5, H: 5.3, N: 7.0, S: 8.2%; t_R (HPLC, λref 360 nm)**:** 5.25 min (100%, Iso 2, *system 1*).

Methyl 2-{1-[4-(methylsulfonyl)phenyl]-5-phenyl-1*H*-pyrazol-3-yl}acetate (2c) and methyl 2-{1-[4-(methylsulfonyl)phenyl]-3-phenyl-1*H*-pyrazol-5-yl}acetate (2e): Starting from methyl (*Z*)-5-hydroxy-3-oxo-5-phenyl-pent-4-enoate (**1c**, 970 mg, 4.41 mmol, 1.0 equiv) and 4-methylsulfonylphenylhydrazin hydrochloride (1079 mg, 4.85 mmol, 1.1 equiv) following general procedure A and column chromatographic purification (chloroform/methanol, 300/1 → 100/1), the 1,5-diaryl-substituted main product **2c** was isolated as a colorless solid (1131 mg, 69%): mp: 81.4-84.3 °C (Optimelt); R_f (chloroform/methanol, 100/1) = 0.29, R_f (petroleum ether /EtOAc, 1/1) = 0.50; ^{1}H NMR (400 MHz, CD_3CN): *δ* 3.06 (s, 3H, SO_2CH_3), 3.70 (s, 3H, $COOCH_3$), 3.76 (s, 2H, CH_2), 6.57 (s, 1H, $H_{pyrazol\ H4}$), 7.26 – 7.31 (m, 2H, $H_{phenyl\ H2/H6}$), 7.34 – 7.42 (m, 3H, $H_{phenyl\ H3/H4/H5}$), 7.47 (d, ^3J 8.9, 2H, $H_{SO2\text{-}Ph\ H2/H6}$), 7.86 (d, ^3J 8.9, 2H, $H_{SO2\text{-}Ph\ H3/H5}$); ^{13}C NMR (101 MHz, CD_3CN): *δ* 34.7 (CH_2), 44.5 (SO_2CH_3), 52.6 (COO<u>C</u>H_3), 110.1 ($CH_{pyrazol\ C4}$), 126.0 ($2CH_{SO2\text{-}Ph\ C2/C6}$), 129.2 ($2CH_{SO2\text{-}Ph\ C3/C5}$), 129.7 ($2CH_{phenyl\ C3/C5}$ + $CH_{phenyl\ C4}$)*, 129.8 ($2CH_{phenyl\ C2/C6}$), 131.0 ($C_{phenyl\ C1}$), 140.1 ($C_{SO2\text{-}Ph\ C4}$), 144.9 ($C_{SO2\text{-}Ph\ C1}$), 145.3 ($C_{pyrazol\ C5}$), 148.8 ($C_{pyrazol\ C3}$), 171.7 (<u>C</u>$OOCH_3$), *two carbon atoms with identical chemical shift; MS (ASAP$^+$): *m/z* (%) = 371 (100) [*M*+H]$^+$, 370 (18) [*M*]$^+$; elemental analysis calc. for $C_{19}H_{18}N_2O_4S$ (370.42): C: 61.61, H: 4.90, N: 7.56, S: 8.65; Found: C: 61.43, H: 4.93, N: 7.43, S: 8.75; t_R (HPLC; λ_{ref} 360 nm): 8.53 min (100%, Iso 4, *system 1*). The 1,3-diaryl-substituted side product **2e** was isolated as a beige solid (248 mg, 15%): mp: 89-92 °C; R_f (chloroform/methanol, 100/1) = 0.40; ^{1}H NMR (400 MHz, CD_3CN): *δ* 3.12 (s, 3H, SO_2CH_3), 3.62 (s, 3H, $COOCH_3$), 3.91 (s, 2H, CH_2), 6.85 (s, 1H, $H_{pyrazol\ H4}$), 7.35 – 7.40 (m, 1H, $H_{phenyl\ H4}$), 7.45 (t, ^3J 7.7, 2H, $H_{phenyl\ H3/H5}$), 7.78 (d, ^3J 8.9, 2H, $H_{SO2\text{-}Ph\ H2/H6}$), 7.88 (dd, ^3J 8.4, $^4J_{2,4}$ 1.3, 2H, $H_{phenyl\ H2/H6}$), 8.06 (d, ^3J 8.9, 2H, $H_{SO2\text{-}Ph\ H3/H5}$); ^{13}C NMR (101 MHz, CD_3CN): *δ* 32.9 (CH_2), 44.6 (SO_2CH_3), 53.0 (COO<u>C</u>H_3), 107.6 ($CH_{Pyrazol\ C4}$), 126.1 ($2CH_{SO2\text{-}Ph\ C2/C6}$), 126.6 ($2CH_{phenyl\ C2/C6}$), 129.3 ($CH_{phenyl\ C4}$), 129.7 ($2CH_{SO2\text{-}Ph\ C3/C5}$), 129.8 ($2CH_{phenyl\ C3/C5}$), 133.7 ($C_{phenyl\ C1}$), 139.2 ($C_{pyrazol\ C5}$), 140.9 ($C_{SO2\text{-}Ph\ C4}$), 144.7 ($C_{SO2\text{-}Ph\ C1}$), 153.2 ($C_{pyrazol\ C3}$), 170.6 (<u>C</u>$OOCH_3$); MS (ASAP$^+$): *m/z* (%) = 371 (100) [*M*+H]$^+$, 370 (38) [*M*]$^+$; elemental analysis calc. for $C_{19}H_{18}N_2O_4S$ (370.42): C: 61.6, H: 4.9, N: 7.6, S: 8.65%, Found: C: 61.3, H: 5.0, N: 7.3, S: 8.5%; t_R (HPLC, λref 360 nm): 11.21 min (100%, Iso 4, *system 1*); Crystals suitable for X-ray analysis were obtained by slow evaporation of a solution of **2e** in chloroform layered with petroleum ether. The obtained crystals were washed with a small amount of petroleum ether and then chloroform.

General procedure B

Lithium aluminum hydride (31 mg, 0.82 mmol, 1.52 equiv) or lithium aluminium deuterid (34 mg, 0.82 mmol, 1.52 equiv) was added portionwise to a solution of the esters **2a-d** (0.54 mmol, 1 equiv) in THF (15 mL) at room temperature. The mixture was heated to 70 °C for 1.5 h, cooled to room temperature, and quenched with water (6 mL). After addition of ethyl acetate and water (15 mL) the mixture was transferred to a separation funnel and the layers were allowed to separate. The organic phase was

collected and the aqueous phase was extracted with ethyl acetate (3 x 30 mL). The combined organic phases were dried over sodium sulfate, filtered, and the solvent was evaporated under reduced pressure. Further purification was carried out as given below to give the pyrazole derivatives **3a-d** and [D_2]**3a**.

{1-[4-(Methylsulfonyl)phenyl]-5-(*p*-tolyl)-1*H*-pyrazol-3-yl}methanol (3a): Starting from **2a** (1.30 g, 3.51 mmol, 1.00 equiv) and 2.4 M $LiAlH_4$ in THF (1.95 mL, 177 mg $LiAlH_4$, 4.68 mmol, 1.33 equiv) following general procedure C but performing the reaction only at room temperature for 30 min furnished **3a** as crude product (1.10 g, 87%, purity determined by HPLC 91%). An analytical sample was obtained by dry column vacuum chromatography ($CHCl_3$/ MeOH 97.5/2.5): pale yellow solid, mp: 127-130 °C); R_f ($CHCl_3$/ MeOH 97.5/2.5) = 0.23; ^1H NMR (400 MHz, DMSO-d_6): δ 2.32 (s, 3 H, CH_3), 3.24 (s, 3 H, CH_3), 4.52 (d, ^3J 5.8, 2 H, CH_2OH), 5.25 (td, ^3J 5.8, ^5J 0.6, 1 H, CH_2OH), 6.60 (s, 1 H, $CH_{pyrazole}$), 7.16 (d, ^3J 8.1, 2 H, CH_{phenyl}), 7.22 (d, ^3J 8.6, 2 H, CH_{phenyl}), 7.48 (d, ^3J 8.4, 1 H, $CH_{SO2\text{-}phenyl}$), 7.93 (d, ^3J 8.4, 1 H, $CH_{SO2\text{-}phenyl}$); ^{13}C NMR (101 MHz, DMSO-d_6): δ 20.8, 43.4, 57.2, 107.8, 124.8, 127.0, 128.0, 128.4, 129.5, 138.3, 138.9, 143.5, 143.7, 155.0; MS (ESI$^+$, M calc. for $C_{18}H_{18}N_2O_3S$ = 342,10) *m/z* (%): 325.2 (100) [*M*-OH]$^+$, 343.2 (37) [*M*+H]$^+$; HRMS (ESI/QTOF) m/z: [*M*+H]$^+$ Calc. for $C_{18}H_{19}N_2O_3S$ 343.1111, Found 343.1119; HPLC: t_R = 3.66 min (91.4%; gradient 70-90%, *system 1*).

{1-[4-(Methylsulfonyl)phenyl]-5-(*p*-tolyl)-1*H*-pyrazol-3-yl}methan-*d₂*-ol ([D_2]3a): Starting from **2a** (210 mg, 0.57 mmol, 1.0 equiv) and lithium aluminium deuterid (36 mg, 0.85 mmol, 1.49 equiv) following general procedure B, the title compound [D_2]**3a** was isolated as pale yellow solid (189 mg, 96%): mp: 138-142 °C; R_f (chloroform/methanol, 95/5) = 0.25; ^1H NMR (400 MHz, $CDCl_3$): δ 1.93 (br. s, 1H, OH), 2.38 (s, 3H, $CH_{3\ tolyl}$), 3.05 (s, 3H, SO_2CH_3), 6.52 (s, 1H, $H_{pyrazol\ H4}$), 7.11 (d, ^3J 8.1, 2H, $H_{tolyl\ H2/H6}$), 7.16 (d, ^3J 7.8, 2H, $H_{tolyl\ H3/H5}$), 7.49 (d, ^3J 8.5, 2H, $H_{SO2\text{-}Ph\ H2/H6}$), 7.88 (d, ^3J 8.6, 2H, $H_{SO2\text{-}Ph\ H3/H5}$); ^{13}C NMR (101 MHz, $CDCl_3$): δ 21.4 (CH_3), 44.7 (SO_2CH_3), 58.6 (dt, $^2J_{D,C}$ 22.1, CD_2OH), 107.7 ($CH_{Pyrazol\ C4}$), 125.1 ($2CH_{SO2\text{-}Ph\ C2/C6}$), 127.0 ($C_{tolyl\ C1}$), 128.5 ($2CH_{SO2\text{-}Ph\ C3/C5}$), 128.8 ($2CH_{tolyl\ C2/C6}$), 129.8 ($2CH_{tolyl\ C3/C5}$), 138.7, ($C_{SO2\text{-}Ph\ C4}$), 139.3 ($C_{tolyl\ C4}$), 144.3 ($C_{SO2\text{-}Ph\ C1}$), 145.0 ($C_{pyrazol\ C5}$), 154.1 ($C_{pyrazol\ C3}$); MS (ASAP$^+$): *m/z* (%) = 344 (100) [*M*]$^+$; t_R (HPLC, λref 360 nm): 3.80 min (88.3%, Iso 2, *system 1*); HRMS (ESI/micrOTOF) m/z: [*M* + Na]$^+$ calc. for $C_{18}H_{16}D_2N_2O_3SNa$ 367.1056; Found 367.1072.

2-{1-[4-(Methylsulfonyl)phenyl]-5-(*p*-tolyl)-1*H*-pyrazol-3-yl}ethan-1-ol (3b): Starting from **2b** (185 mg, 0.48 mmol, 1.0 equiv) and lithium aluminium hydrid (27 mg, 0.71 mmol, 1.48 equiv) following general procedure B resulted in a crude product which was adsorbed on silica gel and purified by column chromatography (chloroform/methanol, 100/1 → 98/2). The title compound **3b** was isolated

as colorless solid (107 mg, 62%): mp: 123.0-126.0 °C (Optimelt); R_f (chloroform/methanol, 95/5) = 0.40; ^{1}H NMR (400 MHz, $CDCl_3$): δ 2.37 (s, 3H, $CH_{3\ tolyl}$), 2.96 (t, ^3J 5.9, 2H, $\underline{CH_2}CH_2OH$), 3.05 (s, 3H, SO_2CH_3), 4.00 (t, ^3J 5.9, 2H, $CH_2\underline{CH_2}OH$), 6.37 (s, 1H, $H_{pyrazol\ H4}$), 7.11 (d, ^3J 8.1, 2H, $H_{tolyl\ H2/H6}$), 7.16 (d, ^3J 8.0, 2H, $H_{tolyl\ H3/H5}$), 7.47 (d, ^3J 8.6, 2H, $H_{SO2\text{-}Ph\ H2/H6}$), 7.86 (d, ^3J 8.7, 2H, $H_{SO2\text{-}Ph\ H3/H5}$); ^{13}C NMR (101 MHz, $CDCl_3$): δ 21.4 ($CH_{3\ tolyl}$), 31.3 ($\underline{C}H_2CH_2OH$), 44.7 (SO_2CH_3), 61.7 ($CH_2\underline{C}H_2OH$), 108.9 ($CH_{pyrazol\ C4}$), 124.9 ($2CH_{SO2\text{-}Ph\ C2/C6}$), 127.2 ($C_{tolyl\ C1}$), 128.4 ($2CH_{SO2\text{-}Ph\ H3/H5}$), 128.7 ($2CH_{tolyl\ C2/C6}$), 129.7 ($2CH_{tolyl\ C3/C5}$), 138.4 ($C_{SO2\text{-}Ph\ C4}$), 139.2 ($C_{tolyl\ C4}$), 144.3 ($C_{SO2\text{-}Ph\ C1}$), 144.5 ($C_{pyrazol\ C5}$), 152.9 ($C_{pyrazol\ C3}$); MS (ASAP$^+$): *m/z* (%) = 357 (100) $[M+H]^+$, 356 (28) $[M]^+$, 326 (28) $[M\text{-}CH_2OH+H]^+$; elemental analysis calc. for $C_{19}H_{20}N_2O_3S$ (356.44): C: 64.0, H: 5.7, N: 7.9, S: 9.0%, found C: 63.7, H: 5.7, N: 7.7, S: 8.7%; t_R (HPLC, λref 360 nm): 4.59 min (95.2%, Iso 3, *system 1*)

2-{1-[4-(Methylsulfonyl)phenyl]-5-phenyl-1*H*-pyrazol-3-yl}ethan-1-ol (3c): Starting from **2c** (201 mg, 0.54 mmol, 1.0 equiv) and lithium aluminium hydrid (31 mg, 0.82 mmol, 1.51 equiv) following general procedure B resulted in a crude product which was adsorbed on silica gel and purified by column chromatography (chloroform/methanol, 100/1 → 98/2). The title compound **3c** was isolated as colorless solid (151 mg, 81%): mp: 108.4-110.0 °C (Optimelt); R_f (chloroform/methanol, 95/5) = 0.41; ^{1}H NMR (400 MHz, CD_3OD): δ 2.94 (t, ^3J 6.8, 2H, $\underline{CH_2}CH_2OH$), 3.14 (s, 3H, SO_2CH_3), 3.91 (t, ^3J 6.8, 2H, $CH_2\underline{CH_2}OH$), 6.54 (s, 1H, $H_{pyrazol\ H4}$), 7.24 – 7.30 (m, 2H, $H_{phenyl\ H2/H6}$), 7.35 – 7.41 (m, 3H, $H_{phenyl\ H3/H4/H5}$), 7.51 (d, ^3J 8.8, 2H, $H_{SO2\text{-}Ph\ H2/H6}$), 7.94 (d, ^3J 8.8, 2H, $H_{SO2\text{-}Ph\ H3/H5}$); ^{13}C NMR (101 MHz, CD_3OD): δ 32.5 ($\underline{C}H_2CH_2OH$), 44.3 (SO_2CH_3), 62.3 ($CH_2\underline{C}H_2OH$), 109.8 ($CH_{pyrazol\ C4}$), 126.6 ($2CH_{SO2\text{-}Ph\ C2/C6}$), 129.5 ($2CH_{SO2\text{-}Ph\ C3/C5}$), 129.9 ($2CH_{phenyl\ C2/C6}$), 130.0 ($2CH_{phenyl\ C3/C5}$ + $CH_{phenyl\ C4}$)*, 131.5 ($C_{phenyl\ C1}$), 140.5 ($C_{SO2\text{-}Ph\ C4}$), 145.3 ($C_{SO2\text{-}Ph\ C1}$), 146.0 ($C_{pyrazol\ C5}$), 153.9 ($C_{pyrazol\ C3}$), *two carbon species with identical chemical shift; MS (ASAP$^+$): m/z (%) = 343 (100) $[M+H]^+$, 312 (38) $[M\text{-}CH_2OH+H]^+$; elemental analysis calc. for $C_{18}H_{18}N_2O_3S$ (342.41): C: 63.1, H: 5.3, N: 8.2, S: 9.4%, Found C: 62.0, H: 5.4, N: 8.0, S: 9.0%; t_R (HPLC, λref 360 nm): 3.51 min (99.9%, Iso 2, *system 1*); HRMS (ESI/micrOTOF) m/z: $[M + Na]^+$ calc. for $C_{18}H_{18}N_2O_3SNa$ 365.0930; Found 365.0939.

2-{1-[4-(Methylsulfonyl)phenyl]-3-(*p*-tolyl)-1*H*-pyrazol-5-yl}ethan-1-ol (3d): Starting from **2d** (188 mg, 0.49 mmol, 1.0 equiv) and lithium aluminium hydrid (28 mg, 0.74 mmol, 1.51 equiv) following general procedure B resulted in a crude product which was adsorbed on silica gel and purified by column chromatography (chloroform/methanol, 100/1 → 98/2). The title compound **3d** was isolated as pale yellow solid (107 mg, 61%): mp: 138-142 °C; R_f (chloroform/methanol, 95/5) = 0.53; ^{1}H NMR (600 MHz, $CDCl_3$): δ 2.38 (s, 3H, $CH_{3\ tolyl}$), 3.02 (t, ^3J 6.4, 2H, $\underline{CH_2}CH_2OH$), 3.09 (s, 3H, SO_2CH_3), 3.94 (t, ^3J 6.3, 2H, $CH_2\underline{CH_2}OH$), 6.66 (s, 1H, $H_{pyrazol\ H4}$), 7.23 (d, ^3J 7.8, 2H, $H_{tolyl\ H3/H5}$), 7.74 (d, ^3J 7.7, 2H, $H_{tolyl\ H2/H6}$),

7.78 (d, ^3J 8.2, 2H, $H_{SO2\text{-}Ph\ H2/H6}$), 8.05 (d, ^3J 8.1, 2H, $H_{SO2\text{-}Ph\ H3/H5}$); ^{13}C NMR (151 MHz, $CDCl_3$): δ 21.5 ($CH_{3\ tolyl}$), 29.9 ($\underline{C}H_2CH_2OH$), 44.7 (SO_2CH_3), 61.5 ($CH_2\underline{C}H_2OH$), 104.8 ($CH_{pyrazol\ C4}$), 125.7 ($2CH_{SO2\text{-}Ph\ C2/C6}$), 125.9 ($2CH_{tolyl\ C2/C6}$), 128.8 ($2CH_{SO2\text{-}Ph\ C3/C5}$), 129.6 ($2CH_{tolyl\ C3/C5}$), 129.7 ($C_{tolyl\ C1}$), 138.5 ($C_{tolyl\ C4}$), 139.3 ($C_{SO2\text{-}Ph\ C4}$), 142.3 ($C_{pyrazol\ C5}$), 144.2 ($C_{SO2\text{-}Ph\ C1}$), 153.1 ($C_{pyrazol\ C3}$); MS (ASAP$^+$): *m/z* (%) = 356 (89) $[M]^+$, 326 (59) $[M\text{-}CH_2OH+H]^+$, 278 (100) $[M\text{-}SO_2CH_3+H]^+$; elemental analysis calc. for $C_{19}H_{20}N_2O_3S$ (356.44): C: 64.0, H: 5.7, N: 7.9, S: 9.0%, Found: C: 63.6, H: 5.6, N: 7.6, S: 8.7%; t_R (HPLC, λ_{ref} 360 nm): 9.01 min (96.9%, Iso 4, *system 1*).

General Procedure C

4-(Dimethylamino)pyridine (16 mg, 0.13 mmol, 0.3 equiv) and pyridine (113.4 µl, 1.40 mmol, 3.2 equiv) was added to a solution of the respective alcohol **3a-d**/$[D_2]$**3a** (0.44 mmol, 1.0 equiv) in DCM (11 mL) at -10 °C and stirred for 10 min at this temperature. Afterwards, a cooled (-10 °C) solution of *p*-toluenesulfonic anhydride (286 mg, 0.88 mmol, 2.0 equiv) in DCM (30 mL) was slowly added, the mixture was allowed to warm up to room temperature, and stirred for 1-2 days as given below. If stated below, further *p*-toluenesulfonic anhydride (286 mg, 0.88 mmol, 2.0 equiv) was added after 21 h reaction time. The solvent was evaporated under reduced pressure and the crude product was adsorbed on silica gel followed by chromatographic purification as given below to obtain the tosyl-substituted compounds **4a-d**/$[D_2]$**4a**.

{1-[4-(Methylsulfonyl)phenyl]-5-(*p*-tolyl)-1*H*-pyrazol-3-yl}methyl 4-methylbenzenesulfonate (4a): Starting from **3a** (408 mg, 1.19 mmol, 1.0 equiv), 4-(dimethylamino)pyridine (46 mg, 0.36 mmol, 0.3 equiv), and pyridine (300 µL, 3.6 mmol, 3.0 equiv) following general procedure C applying a total reaction time of 2 days and column chromatographic purification (petroleum ether / EtOAc, 7/3 → 3/2 → 1/1), the title compound **4a** was isolated as a beige solid (359 mg, 61%): mp: 62-64 °C; R_f (petroleum ether / EtOAc, 3/7) = 0.52; ^{1}H NMR (400 MHz, $CDCl_3$): δ 2.37 (s, 3H, $CH_{3\ tolyl}$), 2.43 (s, 3H, $CH_{3\ tosyl}$), 3.05 (s, 3H, SO_2CH_3), 5.17 (s, 2H, CH_2), 6.47 (s, 1H, $H_{pyrazol\ H4}$), 7.04 (d, ^3J 8.2, 2H, $H_{tolyl\ H2/H6}$), 7.15 (d, ^3J 7.9, 2H, $H_{tolyl\ H3/H5}$), 7.33 (d, ^3J 7.9, 2H, $H_{tosyl\ H3/H5}$), 7.42 (d, ^3J 9.0, 2H, $H_{SO2\text{-}Ph\ H2/H6}$), 7.83 (d, ^3J 8.1, 2H, $H_{tosyl\ H2/H6}$), 7.88 (d, ^3J 9.0, 2H, $H_{SO2\text{-}Ph\ H3/H5}$); ^{13}C NMR (101 MHz, $CDCl_3$): δ 21.4 ($CH_{3\ tolyl}$), 21.8 ($CH_{3\ tosyl}$), 44.7 (SO_2CH_3), 65.6 (CH_2), 109.1 ($CH_{pyrazol\ C4}$), 125.2 ($2CH_{SO2\text{-}Ph\ C2/C6}$), 126.6 ($C_{tolyl\ C1}$), 128.3 ($2CH_{tosyl\ C2/C6}$), 128.5 ($2CH_{SO2\text{-}Ph\ C3/C5}$), 128.7 ($2CH_{tolyl\ C2/C6}$), 129.8 ($2CH_{tolyl\ C3/C5}$), 129.9 ($2CH_{tosyl\ C3/C5}$), 133.4 ($C_{tosyl\ C1}$), 139.1 ($C_{SO2\text{-}Ph\ C4}$), 139.5 ($C_{tolyl\ C4}$), 144.0 ($C_{SO2\text{-}Ph\ C1}$), 145.0 ($C_{pyrazol\ C5}$), 145.2 ($C_{tosyl\ C4}$), 147.5 ($C_{pyrazol\ C3}$); MS (ASAP$^+$): *m/z* (%) = 416 (27) $[M]^+$, 341 (12) $[M\text{-}C_6H_4SO_2CH_3]^+$, 326 (10) $[M\text{-}SO_3C_6H_4CH_3+H]^+$, 172 (100) $[HO_3SC_6H_4CH_3]^+$, 91 (54) $[C_6H_4CH_3]^+$; HRMS (ESI/micrOTOF) m/z: $[M + Na]^+$ calc. for $C_{25}H_{24}N_2O_5S_2Na$ 519.1019; Found 519.1015.

{1-[4-(Methylsulfonyl)phenyl]-5-(*p*-tolyl)-1*H*-pyrazol-3-yl}methyl-*d*$_2$ 4-methylbenzenesulfonate ([D$_2$]4a): Starting from [D$_2$]**3a** (212 mg, 0.62 mmol, 1.0 equiv), 4-(dimethylamino)pyridine (42.4 mg, 0.35 mmol, 0.56 equiv), and pyridine (140.0 µL, 1.73 mmol, 2.79 equiv) following general procedure C applying a total reaction time of 1 day and column chromatographic purification (petroleum ether / EtOAc, 7/3 → 1/1), the title compound [D$_2$]**4a** was isolated as a beige solid (148 mg, 48%): mp: 58-60 °C; *Rf* (petroleum ether / EtOAc, 3/7) = 0.53; ^{1}H NMR (400 MHz, CDCl$_3$): δ 2.37 (s, 3H, $CH_{3\ tolyl}$), 2.43 (s, 3H, $CH_{3\ tosyl}$), 3.05 (s, 3H, SO_2CH_3), 6.48 (s, 1H, $CH_{pyrazol\ H4}$), 7.04 (d, ^3J 7.8, 2H, $H_{tolyl\ H2/H6}$), 7.15 (d, ^3J 7.8, 2H, $H_{tolyl\ H3/H5}$), 7.33 (d, ^3J 8.0, 2H, $H_{tosyl\ H3/H5}$), 7.42 (d, ^3J 8.7, 2H, $H_{SO2\text{-}Ph\ H2/H6}$), 7.83 (d, ^3J 8.0, 2H, $H_{tosyl\ H2/H6}$), 7.88 (d, ^3J 8.3, 2H, $H_{SO2\text{-}Ph\ H3/H5}$); ^{13}C NMR (101 MHz, CDCl$_3$): δ 21.5 ($CH_{3\ tolyl}$), 21.8 ($CH_{3\ tosyl}$), 44.7 (SO_2CH_3), 109.1 ($CH_{pyrazol\ C4}$), 125.2 ($2CH_{SO2\text{-}Ph\ C2/C6}$), 126.6 ($C_{tolyl\ C1}$), 128.3 ($2CH_{tosyl\ C3/C5}$), 128.5 ($2CH_{SO2\text{-}Ph\ C3/C5}$), 128.8 ($2CH_{tolyl\ C2/C6}$), 129.8 ($2CH_{tolyl\ C3/C5}$), 129.9 ($2CH_{tosyl\ C3/C5}$), 133.4 ($C_{tosyl\ C1}$), 139.1 ($C_{SO2\text{-}Ph\ C4}$), 139.5 ($C_{tolyl\ C4}$), 144.0 ($C_{SO2\text{-}Ph\ C1}$), 145.0 ($C_{pyrazol\ C5}$), 145.2 ($C_{tosyl\ C4}$), 147.4 ($C_{pyrazol\ C3}$), signal of CD_2 carbon is not detectable within the spectra due to broadening by coupling with deuterium; MS (ASAP$^+$): *m/z* (%) = 499 (5) [*M*+H]$^+$, 327 (100) [*M*-$SO_3C_6H_4CH_3$]$^+$; t_R (HPLC, λref 360 nm): 7.57 min (97.8%, Iso 2, *system 1*); HRMS (ESI/micrOTOF) m/z: [*M* + Na]$^+$ calc. for $C_{25}H_{22}D_2N_2O_5S_2Na$ 521.1144; Found 521.1149.

2-{1-[4-(Methylsulfonyl)phenyl]-5-(*p*-tolyl)-1*H*-pyrazol-3-yl}ethyl 4-methylbenzenesulfonate (4b): Starting from **3b** (170 mg, 0.48 mmol, 1.0 equiv), 4-(dimethylamino)pyridine (18 mg, 0.15 mmol, 0.3 equiv), and pyridine (123.5 µL, 1.54 mmol, 3.2 equiv) following general procedure C applying a total reaction time of 2 days as well as addition of further *p*-toluenesulfonic anhydride (2 x 321 mg, 1.97 mmol, 4.1 equiv) and column chromatographic purification (DCVC, petroleum ether / EtOAc, 1/1 → 1/2), the title compound **4b** was obtained as colorless solid (185 mg, 76%): mp: 188-190 °C; R_f (petroleum ether / EtOAc, 1/2) = 0.39; ^{1}H NMR (400 MHz, CDCl$_3$): δ 2.38 (s, 3H, $CH_{3\ tolyl}$), 2.41 (s, 3H, $CH_{3\ tosyl}$), 3.04 (s, 3H, SO_2CH_3), 3.08 (t, ^3J 6.9, 2H, $\underline{CH_2}CH_2OR$), 4.38 (t, ^3J 6.8, 2H, $CH_2\underline{CH_2}OR$), 6.31 (s, 1H, $H_{pyrazol\ H4}$), 7.07 (d, ^3J 8.1, 2H, $H_{tolyl\ H2/H6}$), 7.16 (d, ^3J 8.0, 2H, $H_{tolyl\ H3/H5}$), 7.30 (d, ^3J 8.1, 2H, $H_{tosyl\ H3/H5}$), 7.43 (d, ^3J 8.7, 2H, $H_{SO2\text{-}Ph\ H2/H6}$), 7.79 (d, ^3J 8.3, 2 H, $H_{tosyl\ H2/H6}$), 7.86 (d, ^3J 8.7, 2 H, $H_{SO2\text{-}Ph\ H3/H5}$); ^{13}C NMR (101 MHz, CDCl$_3$): δ 21.4 ($CH_{3\ tolyl}$), 21.8 ($CH_{3\ tosyl}$), 28.3 ($\underline{C}H_2CH_2OR$), 44.7 (SO_2CH_3), 69.2 ($CH_2\underline{C}H_2OR$), 108.9 ($CH_{pyrazol\ C4}$), 125.0 ($2CH_{SO2\text{-}Ph\ C2/C6}$), 127.0 ($C_{tolyl\ C1}$), 128.1 ($2CH_{tosyl\ C2/C6}$), 128.4 ($2CH_{SO2\text{-}Ph\ C3/C5}$), 128.7 ($2CH_{tolyl\ C2/C6}$), 129.7 ($2CH_{tolyl\ C3/C5}$), 130.0 ($2CH_{tosyl\ C3/C5}$), 133.2 ($C_{tosyl\ C1}$), 138.5 ($C_{SO2\text{-}Ph\ C4}$), 139.2 ($C_{tolyl\ C4}$), 144.3 ($C_{SO2\text{-}Ph\ C1}$), 144.6 ($C_{pyrazol\ C5}$), 144.9 ($C_{tosyl\ C4}$), 149.9 ($C_{pyrazol\ C3}$); MS (ASAP$^+$): *m/z* (%) = 510 (58) [*M*]$^+$, 338 (100) [*M*-TosOH]$^+$, 259 (57) [*M*-TosOH-SO_2CH_3]$^+$, 193 (43), 172 (75) [TosOH]$^+$; elemental analysis calc. for $C_{26}H_{26}N_2O_5S_2$ (510.63): C: 61.2, H: 5.1, N: 5.5, S: 12.6%, Found: C: 61.5 H: 5.4 N: 5.2 S: 12.2%; t_R (HPLC, λ_{ref} 360 nm): 15.42 min (100%, Iso 3, *system 1*).

General procedure D

(Diethylamino)sulfur trifluoride (79 µl, 0.60 mmol, 1.5 equiv) was added to a solution of the respective alcohol **3a-d**/[D$_2$]**3a** (0.40 mmol, 1.0 equiv) in DCM (22 mL) at -10 °C. The reaction mixture was allowed to warm up to room temperature and stirred for 2 days. If stated below, further (diethylamino)sulfur trifluoride (52.6 µL, 0.40 mmol, 1.0 equiv) was added after a reaction time of 24 h. The reaction was quenched with water (4 mL), the organic phase was separated, and the aqueous phase was extracted with DCM (3 x 5 mL). The combined organic phases were dried over sodium sulfate, filtered, and the solvent was removed under reduced pressure. The crude product was adsorbed on silica gel and purified by column chromatography as given below to obtain the fluoro-substituted compounds **5a-d**/[D$_2$]**5a**.

3-(Fluoromethyl)-1-[4-(methylsulfonyl)phenyl]-5-(*p*-tolyl)-1*H*-pyrazole (5a): Starting from **3a** (172 mg, 0.50 mmol, 1.0 equiv) and (diethylamino)sulfur trifluoride (90 µL, 0.68 mmol, 1.36 equiv) in DCM (10 mL) following general procedure D with a reaction time of 22 h followed by column chromatographic purification (petroleum ether / EtOAc, 1/1), the title compound **5a** was obtained as a beige solid (146 mg, 85%): mp: 168-170 °C; R_f (petroleum ether / EtOAc, 1/1) = 0.34; ^{1}H NMR (400 MHz, d_6-DMSO): δ 2.32 (s, 3H), 3.26 (s, 3 H), 5.46 (d, $^2J_{H,F}$ 48.1, 2 H), 6.82 (d, ^4J 1.4, 1 H), 7.18 (d, ^3J 8.3, 2 H, H$_{tolyl}$), 7.23 (d, ^3J 8.0, 2 H, H$_{tolyl}$), 7.53 (d, ^3J 8.8, 2 H, H$_{SO2\text{-}Ph\ H2/H6}$), 7.96 (d, ^3J 8.8, 2 H, H$_{SO2\text{-}Ph\ H3/H5}$); ^{19}F NMR (376 MHz, d_6-DMSO): δ -207.4 (t, $^2J_{C,F}$ 48); MS (ASAP$^+$): *m/z* (%) = 344 (100) [*M*]$^+$, 296 (63) [*M*-CH$_2$F-CH$_3$]$^+$, 265 (58) [*M*-SO$_2$CH$_3$]$^+$, 245 (35) [*M*-HF-SO$_2$CH$_3$]$^+$; t$_R$ (HPLC, λ_{ref} 360 nm): 8.89 min (100%, Iso 3, *system 1*); HRMS (ESI/micrOTOF) m/z: [*M* + Na]$^+$ calc. for $C_{18}H_{17}FN_2O_2SNa$ 367.0887; Found 367.0885.

3-(Fluoromethyl-*d*$_2$)-1-[4-(methylsulfonyl)phenyl]-5-(*p*-tolyl)-1*H*-pyrazole ([D$_2$]5a): Starting from **3a** (169 mg, 0.49 mmol, 1.0 equiv) and (diethylamino)sulfur trifluoride (100 µL, 0.74 mmol, 1.51 equiv) in DCM (10 mL) following general procedure D with a reaction time of 18 h followed by column chromatographic purification (petroleum ether / EtOAc, 1/1), the title compound [^{18}F]**5a** was obtained as a beige solid (80 mg, 47%): mp: 166.6-168.8 °C (Optimelt); R_f (petroleum ether / EtOAc, 1/1) = 0.34, (petroleum ether / EtOAc, 1/2) = 0.68; ^{1}H NMR (400 MHz, CDCl$_3$): δ 2.38 (s, 3H, CH$_{3\ tolyl}$), 3.06 (s, 3H, SO$_2$CH$_3$), 6.62 (d, $^4J_{H,F}$ 1.3, 1H, H$_{pyrazol\ H4}$), 7.12 (d, ^3J 8.2, 2H, H$_{tolyl\ H2/H6}$), 7.17 (d, ^3J 8.0, 2H, H$_{tolyl\ H3/H5}$), 7.51 (d, ^3J 8.7, 2H, H$_{SO2\text{-}Ph\ H2/H6}$), 7.90 (d, ^3J 8.7, 2H, H$_{SO2\text{-}Ph\ H3/H5}$); ^{13}C NMR (101 MHz, CDCl$_3$): δ 21.5 (CH$_{3\ tolyl}$), 44.7 (SO$_2$CH$_3$), 108.8 (d, $^3J_{4,F}$ 2, CH$_{pyrazol\ C4}$), 125.3 (2CH$_{SO2\text{-}Ph\ C2/C6}$), 126.8 (C$_{tolyl\ C1}$), 128.5 (2CH$_{SO2\text{-}Ph\ C3/C5}$), 128.8 (2CH$_{tolyl\ C2/C6}$), 129.8 (2CH$_{tolyl\ C3/C5}$), 139.0 (C$_{SO2\text{-}Ph\ C4}$), 139.5 (C$_{tolyl\ C4}$), 144.2 (C$_{SO2\text{-}Ph\ C1}$), 145.2 (d, $^4J_{C,F}$ 2, C$_{pyrazol\ C5}$), 149.7 (d, $^2J_{C,F}$ 22, C$_{pyrazol\ C3}$), signal of CD$_2$F is not detectable within the spectra due to broadening by coupling with deuterium and fluorine; ^{19}F NMR (376 MHz, CDCl$_3$): δ -215.48-215.65 (m) MS (ASAP$^+$): *m/z* (%) = 346 (100) [*M*]$^+$, 298 (44) [*M*-CD$_2$F-CH$_3$+H]$^+$, 267 (35) [*M*-SO$_2$CH$_3$]$^+$; t$_R$ (HPLC, λ_{ref}

360 nm): 5.82 min (99.0%, Iso 2, *system 1*); HRMS (ESI/micrOTOF) m/z: [*M* + Na]$^+$ calc. for $C_{18}H_{15}D_2FN_2O_2SNa$ 369.1012; Found 369.1039.

3-(2-Fluoroethyl)-1-[4-(methylsulfonyl)phenyl]-5-(*p*-tolyl)-1*H*-pyrazole (5b): Starting from **3b** (143 mg, 0.40 mmol, 1.0 equiv) and (diethylamino)sulfur trifluoride (79 µl, 0.60 mmol, 1.5 equiv and 52,6 µl, 0.40 mmol, 1.0 equiv after 23 h) in DCM (22 mL) following general procedure D with a reaction time of 2 days and column chromatographic purification (petroleum ether / EtOAc, 1/1), the title compound **5b** was obtained as a beige solid (129 mg, 90%): mp: 132-134 °C; R_f (petroleum ether / EtOAc, 1/2) = 0.43; ^{1}H NMR (400 MHz, $CDCl_3$): δ 2.38 (s, 3H, $CH_{3\ tolyl}$), 3.05 (s, 3H, SO_2CH_3), 3.14 (dt, $^3J_{H,F}$ 24.4, ^3J 6.3, 2H, $\underline{CH_2}CH_2F$), 4.79 (dt, $^3J_{H,F}$ 46.9, ^3J 6.3, 2H, $CH_2\underline{CH_2}F$), 6.42 (s, 1H, $H_{pyrazol\ H4}$), 7.11 (d, ^3J 8.1, 2H, $H_{tolyl\ H2/H6}$), 7.16 (d, ^3J 8.2, 2H, $H_{tolyl\ H3/H5}$), 7.49 (d, ^3J 8.5, 2H, $H_{SO2\text{-}Ph\ H2/H6}$), 7.87 (d, ^3J 8.5, 2H, $H_{SO2\text{-}Ph\ H3/H5}$); ^{13}C NMR (151 MHz, $CDCl_3$, measured at 600 MHz NMR device): δ 21.4 ($CH_{3\ tolyl}$), 29.9 (d, $^2J_{C,F}$ 21, $\underline{C}H_2CH_2F$), 44.7 (SO_2CH_3), 82.9 (d, $^1J_{C,F}$ 168, $CH_2\underline{C}H_2F$), 109.0 ($CH_{pyrazol\ H4}$), 125.0 ($2CH_{SO2\text{-}Ph\ C2/C6}$), 127.2 ($C_{tolyl\ C1}$), 128.4 ($2CH_{SO2\text{-}Ph\ C3/C5}$), 128.8 ($2CH_{tolyl\ C2/C6}$), 129.7 ($2CH_{tolyl\ C3/C5}$), 138.4 ($C_{SO2\text{-}Ph\ C4}$), 139.2 ($C_{tolyl\ C4}$), 144.5 ($C_{SO2\text{-}Ph\ C1}$), 144.7 ($C_{tolyl\ C1}$), 150.8 (d, $^3J_{C,F}$ 6, $C_{pyrazol\ C3}$); ^{19}F NMR (376 MHz, $CDCl_3$): δ -216.7; MS (ASAP$^+$): *m/z* (%) = 358 (100) [*M*]$^+$, 338 (62) [*M*-HF]$^+$, 325 (39) [*M*-CH_2F]$^+$; t_R (HPLC, λ_{ref} 360 nm): 8.88 min (99.1%, Iso 3, *system 1*); HRMS (ESI/micrOTOF) m/z: [*M* + Na]$^+$ calc. for $C_{19}H_{19}FN_2O_2SNa$ 381.1043; Found 381.1061.

3-(2-Fluoroethyl)-1-[4-(methylsulfonyl)phenyl]-5-phenyl-1*H*-pyrazole (5c): Starting from **3c** (148 mg, 0.43 mmol, 1.0 equiv) and (diethylamino)sulfur trifluoride (170 µl, 1.30 mmol, 3.0 equiv) in DCM (24 mL) following general procedure D with a reaction time of 18 h and column chromatographic purification (petroleum ether / EtOAc, 1/1 → 3/7), the title compound **5c** was obtained as a colorless solid (83 mg, 56%): mp: 99.5-101.5 °C; R_f (petroleum ether / EtOAc, 1/4) = 0.47; ^{1}H NMR (400 MHz, $CDCl_3$): δ 3.05 (s, 3H, SO_2CH_3), 3.15 (dt, $^3J_{H,F}$ 24.5, ^3J 6.3, 2H, $\underline{CH_2}CH_2F$), 4.79 (dt, $^2J_{H,F}$ 46.9, ^3J 6.3, 2H, $CH_2\underline{CH_2}F$), 6.46 (s, 1H, $H_{pyrazol\ H4}$), 7.20 – 7.27 (m, 2H, $H_{phenyl\ C2/C6}$), 7.32 – 7.40 (m, 3H, $H_{phenyl\ H3/H4/H5}$), 7.49 (d, ^3J 8.8, 2H, $H_{SO2\text{-}Ph\ H2/H6}$), 7.88 (d, ^3J 8.8, 2H, $H_{SO2\text{-}Ph\ H3/H5}$); ^{13}C NMR (101 MHz, $CDCl_3$): δ 29.9 (d, $^2J_{C,F}$ 21, $\underline{CH_2}CH_2F$), 44.7 (SO_2CH_3), 82.8 (d, $^1J_{C,F}$ 168, $CH_2\underline{CH_2}F$), 109.3 ($CH_{pyrazol\ C4}$), 125.0 ($2CH_{SO2\text{-}Ph\ C2/C6}$), 128.5 ($2CH_{SO2\text{-}Ph\ C3/C5}$), 128.9 ($2CH_{phenyl\ C2/C6}$) , 129.0 ($2CH_{phenyl\ C3/C5}$) , 129.1 ($CH_{phenyl\ C4}$) , 130.2 ($C_{phenyl\ C1}$), 138.5 ($C_{SO2\text{-}Ph\ C4}$), 144.4 ($C_{SO2\text{-}Ph\ C1}$), 144.5 ($C_{pyrazol\ C5}$), 150.9 (d, $^3J_{C,F}$ 6, $C_{pyrazol\ C3}$); ^{19}F NMR (376 MHz, $CDCl_3$): δ -216.7; MS (ASAP$^+$): *m/z* (%) = 345 (100) [*M*+H]$^+$, 344 (99) [*M*]$^+$; t_R (HPLC, λ_{ref} 360 nm): 10.78 min (100%, Iso 4, *system 1*); HRMS (ESI/micrOTOF) m/z: [M + Na]$^+$ calc. for $C_{18}H_{17}FN_2O_2SNa$ 367.0887; Found 367.0895. Crystals suitable for X-ray analysis were obtained by diffusion of petroleum ether into a solution of **5c** in chloroform followed by slow evaporation of the solvent.

5-(2-Fluoroethyl)-1-[4-(methylsulfonyl)phenyl]-3-(*p*-tolyl)-1*H*-pyrazole (5d): Starting from **3d** (89 mg, 0.25 mmol, 1.0 equiv), (diethylamino)sulfur trifluoride (49.1 µl, 0.38 mmol, 1.5 equiv and 32.8 µl, 0.25 mmol, 1.0 equiv after 25 h) in DCM (13.7 mL) following general procedure D with a reaction time of 2 days and column chromatographic purification (petroleum ether / EtOAc, 1/1), the title compound **5d** was obtained as a colorless solid (69 mg, 77%): mp: 127.0-128.3 °C (Optimelt); R_f (petroleum ether / EtOAc, 1/2) = 0.59; ^{1}H NMR (600 MHz, $CDCl_3$): δ 2.39 (s, 3H, $CH_{3\ tolyl}$), 3.10 (s, 3H, SO_2CH_3), 3.18 (dt, $^3J_{H,F}$ 23.6, ^3J 6.0, 2H, $\underline{CH_2}CH_2F$), 4.73 (dt, $^2J_{H,F}$ 46.7, ^3J 6.0, 2H, $CH_2\underline{CH_2}F$), 6.72 (s, 1H, $H_{pyrazol\ H4}$), 7.24 (d, ^3J 7.8, 2H, $H_{tolyl\ H3/H5}$), 7.71 – 7.80 (m, 4H, $H_{SO2\text{-}Ph\ H2/H6\ +\ tolyl\ H2/H6}$), 8.08 (d, ^3J 8.2, 2H, $H_{SO2\text{-}Ph\ H3/H5}$); ^{13}C NMR (151 MHz, $CDCl_3$): δ 21.5 ($CH_{3\ tolyl}$), 28.1 (d, $^2J_{C,F}$ 22, $\underline{C}H_2CH_2F$), 44.8 (SO_2CH_3), 81.9 (d, $^1J_{C,F}$ 171, $CH_2\underline{C}H_2F$), 105.0 ($CH_{pyrazol\ C4}$), 125.8 ($2CH_{SO2\text{-}Ph\ C2/C6}$), 125.9 ($2CH_{tolyl\ C2/C6}$), 128.9 ($2CH_{SO2\text{-}Ph\ C3/C5}$), 129.6 ($2CH_{tolyl\ C3/C5}$), 129.7 ($C_{tolyl\ C1}$), 138.5 ($C_{tolyl\ C4}$), 139.5 ($C_{SO2\text{-}Ph\ C4}$), 140.7 (d, $^3J_{C,F}$ 7, $C_{pyrazol\ C5}$), 144.1 ($C_{SO2\text{-}Ph\ C1}$), 153.3 ($C_{pyrazol\ C3}$); ^{19}F NMR (564 MHz, $CDCl_3$): δ -219.1 (tt, $^2J_{H,F}$ 47, $^3J_{H,F}$ 24); MS (ASAP$^+$): *m/z* (%) = 358 (100) $[M]^+$; elemental analysis calc. for $C_{19}H_{19}FN_2O_2S$ (358.43): C: 63.7, H: 5.3, N: 7.8, S: 8.9%, Found: C: 63.95 H: 5.4 N: 7.6 S: 8.7%; t_R (HPLC, λ_{ref} 360 nm): 10.38 min (96.9%, Iso 3, *system 1*)

7.3. Automated radiosyntheses

The tosyl-substituted precursor **4a**/[D_2]**4a**/**4b** was reacted in an automated synthesizer module with [^{18}F]fluoride to the radiotracer [^{18}F]**5a**/[D_2,^{18}F]**5a**/[^{18}F]**5b**. For this purpose, [^{18}F]fluoride was trapped on an ion exchange resin (Waters, Sep-Pak Accell Plus QMA Carbonate Plus Light Cartridge, 46 mg sorbent per cartridge; conditioned sequentially with 5 mL H_2O, 5 mL 1M $NaHCO_3$ and 5 mL H_2O) and eluted with 1.5 mL of a weak alkaline kryptofix solution (26.6 mM K_{222}, 3.4 mM K_2CO_3, 15/85 (*v/v*) H_2O/MeCN) into the reactor. After azeotropic drying with 3 mL MeCN, the [^{18}F]KF/K_{222} complex was reacted with 5 mg precursor (**4a** (10 µmol)/[D_2]**4a** (10 µmol)/**4b** (9.8 µmol)) dissolved in 1.0 mL MeCN for 15 min at 80 °C. The mixture was cooled to 50 °C, diluted with 1.0 mL eluent (MeCN/H_2O + 0.1% TFA, 40/60), and filtered through a PTFE filter (d=15 mm; 20 µm). The crude product was purified by semi-preparative HPLC (*System 4 and 5*) where the radiotracers eluted at the following time intervals after injection: [^{18}F]**5a** and [D_2,^{18}F]**5a** at t_R = 47-50 min, and [^{18}F]**5b** at t_R = 51-53 min. The product containing fraction was collected, diluted in 30 mL water, and separated from the HPLC eluent by solid phase extraction using a RP18 cartridge (Chromafix C_{18} ec (s), Macherey-Nagel Part Nr. 731804). Finally, the RP18 cartridge was washed with water (4 mL) and the radiotracer [^{18}F]**5a**/[D_2,^{18}F]**5a**/[^{18}F]**5b** was eluted with 1 mL EtOH and concentrated to a volume of 0.2 mL under a stream of nitrogen. This procedure gave the radiotracers suitable for further *in vitro* and *in vivo* studies in a radiochemical yield (RCY), radiochemical purity (RCP), chemical purity (CP), molar activity (A_m), and synthesis time (t_s) as follows: [^{18}F]**5a**: RCY 35 ± 9% (n = 8), RCP > 99%, CP > 94%, A_m 7 – 54 GBq/µmol, and t_s = 116 min;

$[D_2,^{18}F]$**5a**: RCY 33 ± 6% (n = 4), RCP > 99%, CP > 92%, A_m 13 – 44 GBq/µmol, and t_s = 129 min; $[^{18}F]$**5b**: RCY 38 ± 8% (n = 6), RCP > 99%, CP > 94%, A_m 4 – 40 GBq/µmol, and t_s = 121 min. A representative experiment starting from 3.48 GBq $[^{18}F]$fluoride resulted in the formation of 532 MBq of $[^{18}F]$**5a**.

7.4. In vitro studies

7.4.1 COX inhibition assay

The COX inhibition potency against ovine COX-1 and human COX-2 was determined using the fluorescence-based COX assay 'COX Fluorescent Inhibitor Screening Assay Kit' (catalog number 700100; Cayman Chemical, Ann Arbor, MI) according to the manufacturer's instructions as previously reported by us.[7] In brief, as a measure of COX mediated conversion of arachidonic acid to PG H_2 in the presence of different inhibitor concentrations this assay utilizes the peroxidase component of COX and analyzes the formation of fluorescent resorufin. All compounds were assayed in a concentration range of 10 nM to 100 µM, and every inhibitor concentration was assayed in duplicate. Celecoxib was used as internal control. IC_{50} values were estimated using a nonlinear logistic regression fitting procedure (sigmoidal dose−response model) with Prism Software.

7.4.2 LogD$_{7.4HPLC}$ determination

The distribution coefficient was determined by application of an HPLC method originally described by Donovan and Pescatore.[8] The following HPLC system was used: Agilent 1100 HPLC (binary pump G1312A, autosampler G1313A, column oven G1316A, degasser G1322A, UV detector G1314A, γ detector Gabi Star (Raytest); column ODP-50 4B (Shodex Asahipak 50 x 4.6 mm); eluent: MeOH/ PBS (10 mM, pH 7.4), gradient $t_{0\ min}$ 70/30 – $t_{10\ min}$ 100/0 – $t_{18\ min}$ 70/30, flow rate = 0.6 mL/min with UV detection at 254 nm as recently described by us.[9] Toluene (t_R 4.16 min) and triphenylene (t_R 12.89 min) served as references to calculate log$D_{7.4HPLC}$ as given in formula 4 of reference[8]. Retention time of $[^{18}F]$**5a** (t_R 4.12 min), $[D_2,^{18}F]$**5a** (t_R 4.12 min), and $[^{18}F]$**5b** (t_R 3.85 min) corresponds to a log$D_{7.4HPLC}$ of 2.72, 2.72, and 2.65, respectively.

7.4.3 Stability towards liver microsomes of mice

Microsome experiments with $[^{18}F]$**5a**, $[D_2,^{18}F]$**5a**, and $[^{18}F]$**5b** in the presence of NADPH (oxidizing conditions) were performed using 'Mouse (CD-1) Microsomes' (Gibco^{TM}, Cat. No. MSMCPL) according to the procedure recently described.[10] Incubations had a final volume of 250 µL. The radiotracer dissolved in ethanol (8 µL; 0.6-0.8 MBq/µL) was diluted with PBS (92 µL) followed by the addition of DMSO (4.6 µL; no-carrier-added mixture) or the respective reference compound (4.6 µL of 2.7 mM

stock in DMSO, carrier-added mixture, 50 µM final). PBS (107.9 µL) and mouse liver microsomes (12.5 µL of 20 mg/mL stock; 1 mg/mL final) were mixed in a 1.5 mL Eppendorf tube and stored for 15 min on ice. After that time, the mixture was preincubated at 37 °C for 3 min. Subsequently, NADPH (25 µL of a freshly prepared 20 mM solution in PBS, 2 mM final) was added and the mixture was again incubated at 37 °C for 3 min. Then, the radiotracer solution (104.6 µL) was added and the mixture was incubated again at 37 °C. After distinct time points (10, 30, 60, and 120 min), an aliquot (40 µL) was withdrawn and added to CH_3CN (160 µL). The mixture was vortexed for 30 s, stored on ice for 4 min, and centrifuged (5 min at 14,000 rpm). Aliquots for analytical radio-HPLC (100 µL) and radio-TLC (2 µL) were withdrawn from the supernatant. Radio-TLC was performed on RP-18W/UV_{254} aluminum plates (Macherey Nagel, pre-coated TLC sheets ALUGRAM RP-18W/UV_{254}, 818146) using a solvent mixture of 45% CH_3CN/water (containing 0.1% TFA) as eluent. Testosteron (40 µM final) was used as positive control for oxidation. Complete conversion of testosteron was confirmed by UPLC-DAD (*system 2*) after 60 min. Carrier-added samples were stored at -20°C and analyzed after radioactive decay by UPLC-MS/MS for structural elucidation of the metabolites (*system 3*, ESI+ in MS, MS/MS, and MS/MS survey mode with the following MS parameters: capillary voltage 2.80 kV, cone voltage between 20 and 30 V, source temperature 150 °C, desolvation temperature 450 °C, collision gas (Ar) flow 0.15 mL/min, MS mode collision energy 12.00, MS/MS mode collision energy between 25 and 45).

7.4.4 Cell uptake studies in vitro

Binding and uptake of [^{18}F]**5a**, [D_2,^{18}F]**5a**, and [^{18}F]**5b** was investigated using the two human cell lines Mel-Juso (human malignant melanoma line, DSMZ ACC-74; COX-2-negative) and A2058 (human malignant melanoma line, ATCC CRL-11147; COX-2-positive), significantly differing in their COX-2 expression levels and their COX-1/COX-2-expression pattern. Cells were cultivated in Dulbecco's modified Eagles' medium supplemented with 10% heat-inactivated fetal calf serum (FCS), penicillin (100 U/mL), and streptomycin (100 µg/mL) at 37 °C and 5% CO_2 in a humidified incubator. Radiotracer uptake studies were performed in confluent monolayer cultures as described elsewhere with some modifications.[9,11] In brief, radiotracer was added to the cells at an activity concentration of 0.7 MBq/mL (0.5 mL per well) and cellular binding and uptake was investigated after 5, 10, 30, 60, and 120 min at 37 °C. For blocking experiments, cells were preincubated for 30 min with 100 µM celecoxib. Uptake data are expressed as percent injected dose per mg protein (% ID/mg protein).

7.5 In vivo studies

All animal experiments were carried out according to the guidelines of the German Regulations for Animal Welfare. The protocols were approved by the local Ethical Committee for Animal Experiments (AZ 24-9168.21-4/2004-1).

7.5.1 Metabolic stability in vivo

For *in vivo* stability, radiotracer [^{18}F]**5a**, [D_2,^{18}F]**5a**, or [^{18}F]**5b** was injected i.v. into male Wistar rats (n=2, body weight 203 ± 31 g, injected dose 70 ± 10 MBq) under desflurane anesthesia (10% desflurane in 30% oxygen/air). Using a catheter, blood samples from femoral artery were taken at 1, 3, 5, 10, 20, 30, and 60 min p.i.[9,11] Resulting loss of volume was compensated by i.v. injection of E153. Plasma was separated by centrifugation (3 min; 13,000 × g) followed by precipitation of plasma proteins with ice cold Supersol (EtOH 20% (v/v), Triton X-100 0.5% (v/v), EDTA 5 mM, o-Phenanthrolin 0.5 mM, Saponin 0.1% (w/v)). Clear supernatant separated by a second centrifugation step (3 min; 13,000 × g) was analyzed by radio-HPLC (Hewlett Packard Series 1100 equipped with a γ-detector (Raytest Ramona), Zorbax 300SB-C_{18} column, 250 × 9.4 mm, 4 µm; eluent: (A): 0.1% TFA in MeCN, (B): 0.1% TFA in H_2O; flow rate 3 mL/min, gradient: $t_{0\ min}$ 5/95 – $t_{5\ min}$ 5/95 - $t_{15\ min}$ 95/5 – $t_{20\ min}$ 95/5 - $t_{25\ min}$ 5/95 – $t_{30\ min}$ 5/95) and by radio-TLC (RP18 aluminum foil, 0.1% TFA in acetonitrile/ 0.1% TFA in water (80 /20)). For total protein precipitation, plasma samples were diluted with twice the volume of 15% trichloroacetic acid (TCA) in water instead of Supersol. Clear supernatant was separated by centrifugation (3 min; 13,000 × g) and analyzed as described above.

7.5.2 Biodistribution in healthy rats

To investigate biodistribution of [^{18}F]**5a**, [D_2,^{18}F]**5a**, and [^{18}F]**5b**, healthy rats (body weight 150 ± 20 g) were injected i.v. with about 1.5 MBq of the radiotracer in 0.4 ml 0.9% NaCl with a maximum of 10% ethanol (v/v). Animals were sacrificed at 5 and 60 min p.i. Organs and tissues of interest were excised, weighed, and radioactivity was determined using the Wizard™3'' gamma counter. Activity in selected organs and tissues was expressed as standardized uptake value (SUV; SUV = activity concentration in tissue [Bq/g] × body weight [g] / injected dose [Bq]) or % injected dose (% ID). Values are given as mean ± SD (n = 8 for each time point and tracer).

7.5.3 Small animal PET studies in healthy rats

Pharmacokinetic of [^{18}F]**5a**, [D_2,^{18}F]**5a**, and [^{18}F]**5b** was further assessed by dynamic small animal PET imaging in healthy rats. General anesthesia of rats was induced and maintained by inhalation of 10% desflurane in 30% oxygen/air (v/v). *In vivo* PET imaging using a dedicated small animal PET/CT system (nanoScan PET/CT, Mediso, Budapest, Hungary) was performed as described elsewhere.[12] In brief, PET

acquisition was started 20 s before bolus injection of [^{18}F]**5a**, [D_2,^{18}F]**5a**, or [^{18}F]**5b** (13 ± 0.9 MBq in 0.4 mL 0.9% NaCl) through a needle catheter into a lateral tail vein of the rat and emission data were recorded continuously for 60 min (dynamic PET scan). Afterwards, a static whole body PET scan was performed for at least 30 min. In addition, CT imaging was used for both anatomic information and attenuation correction. Acquired emission data from dynamic PET scan were sorted into 32 time frames and reconstructed using Tera-Tomo™ 3D PET reconstruction algorithm (Mediso). PET data from static scan were reconstructed using the same algorithm but without time framing. Data were calculated as standard uptake value (SUV, SUV = activity concentration in tissue [Bq/g] × body weight [g] / injected dose [Bq]), defined as the tracer concentration at a certain time point normalized to injected dose per unit body weight.

8. Copies of ^{1}H NMR and ^{13}C NMR spectra of new compounds

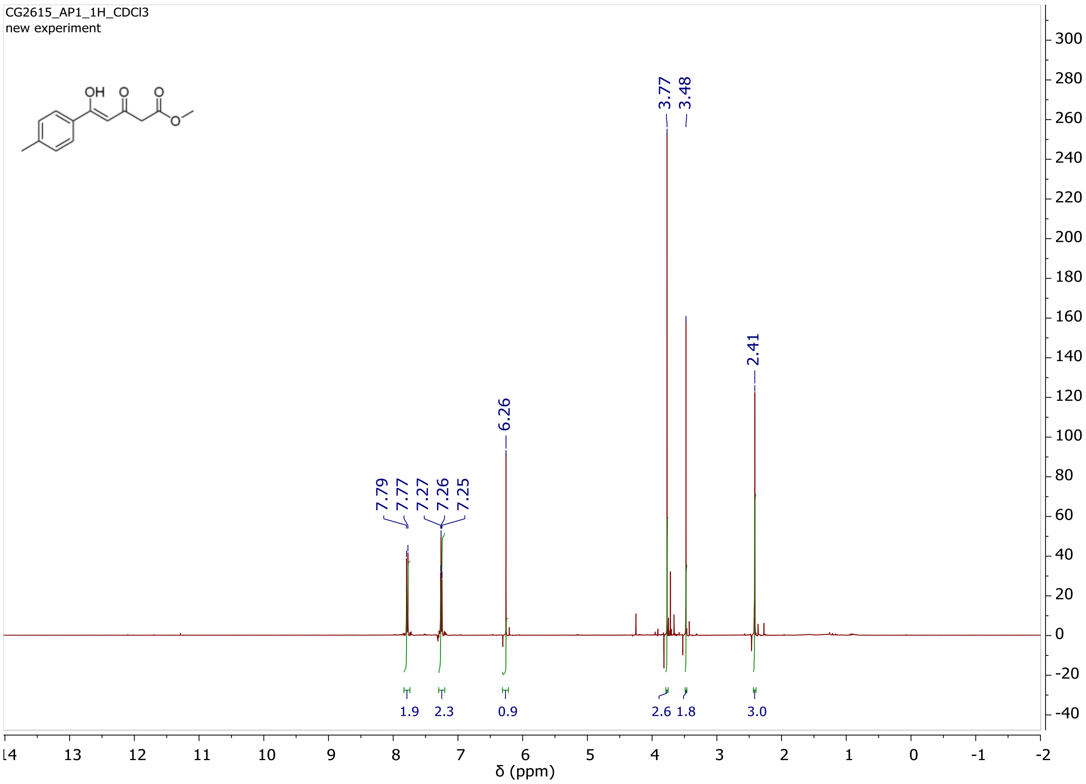

Figure S25. ^{1}H NMR spectrum of compound **1c** in $CDCl_3$

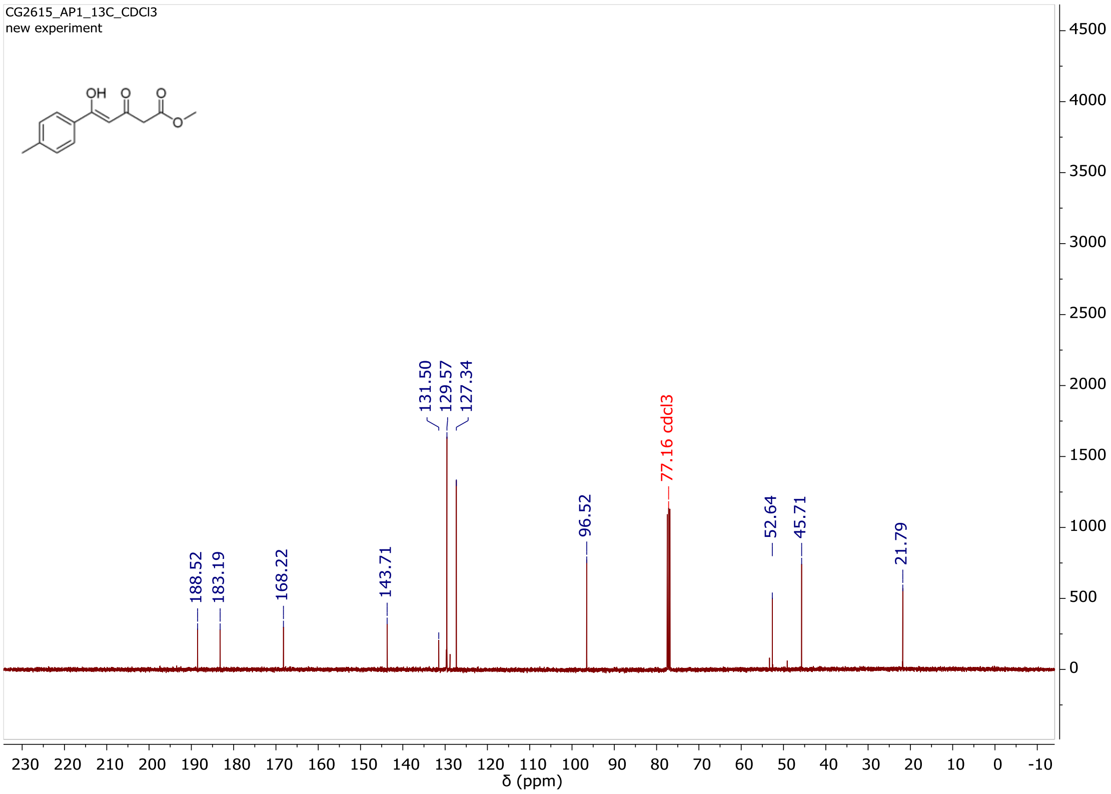

Figure S26. ^{13}C NMR spectrum of compound **1c** in $CDCl_3$

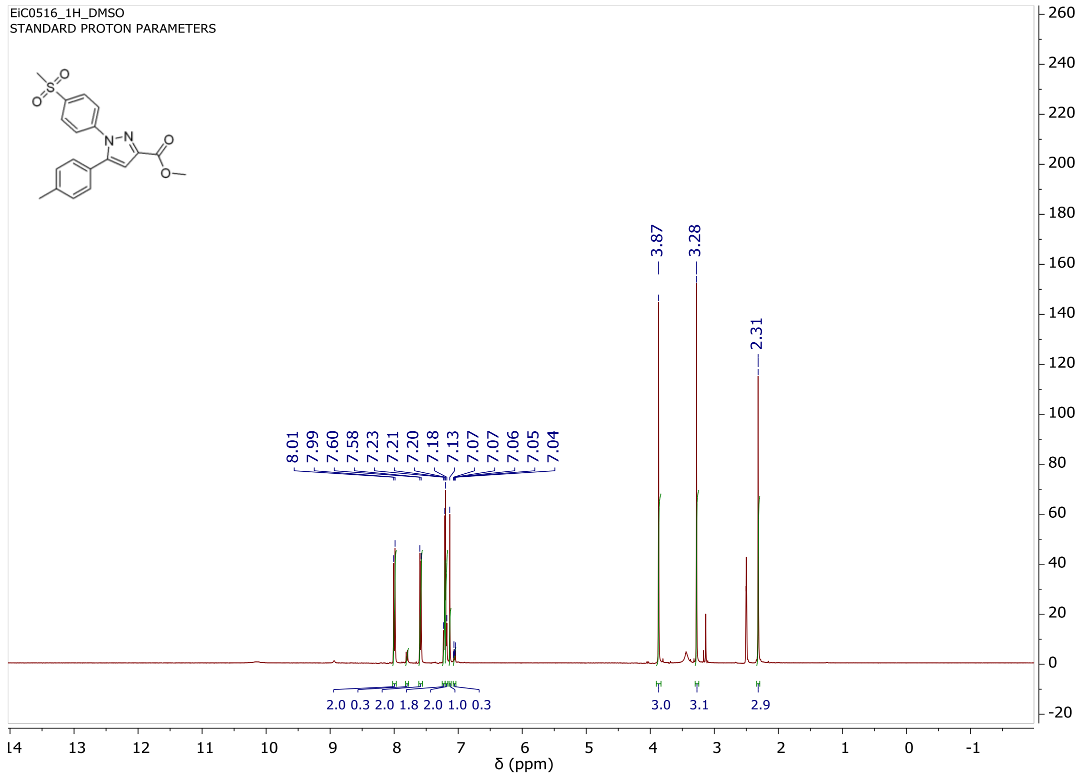

Figure S27. ^{1}H NMR spectrum of compound **2a** in DMSO-d_6

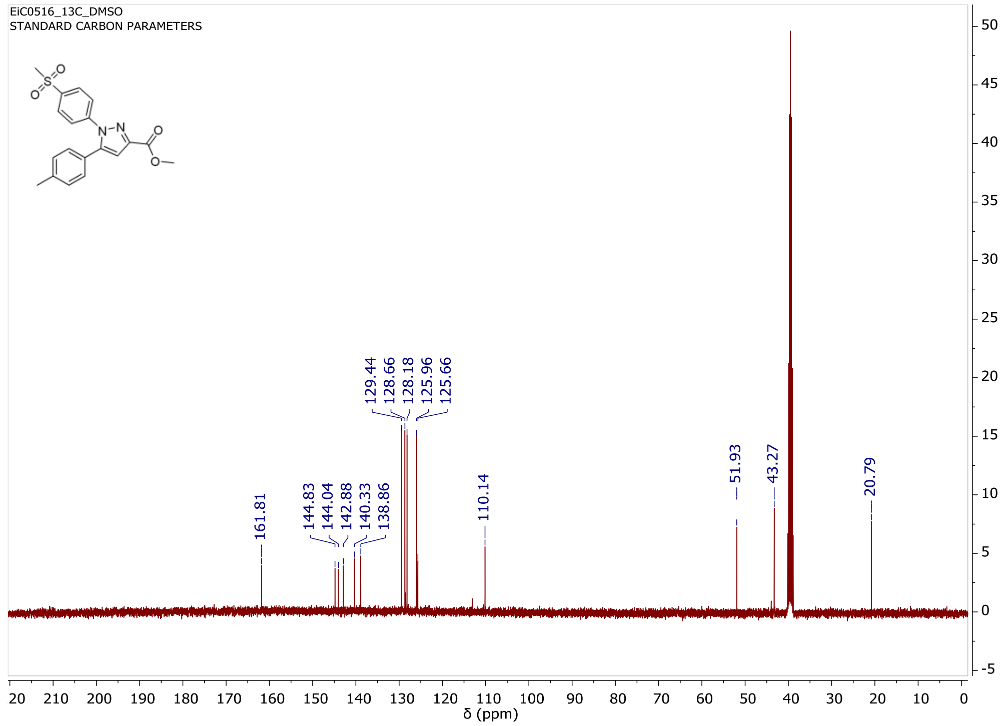

Figure S28. ^{13}C NMR spectrum of compound **2a** in DMSO-d_6

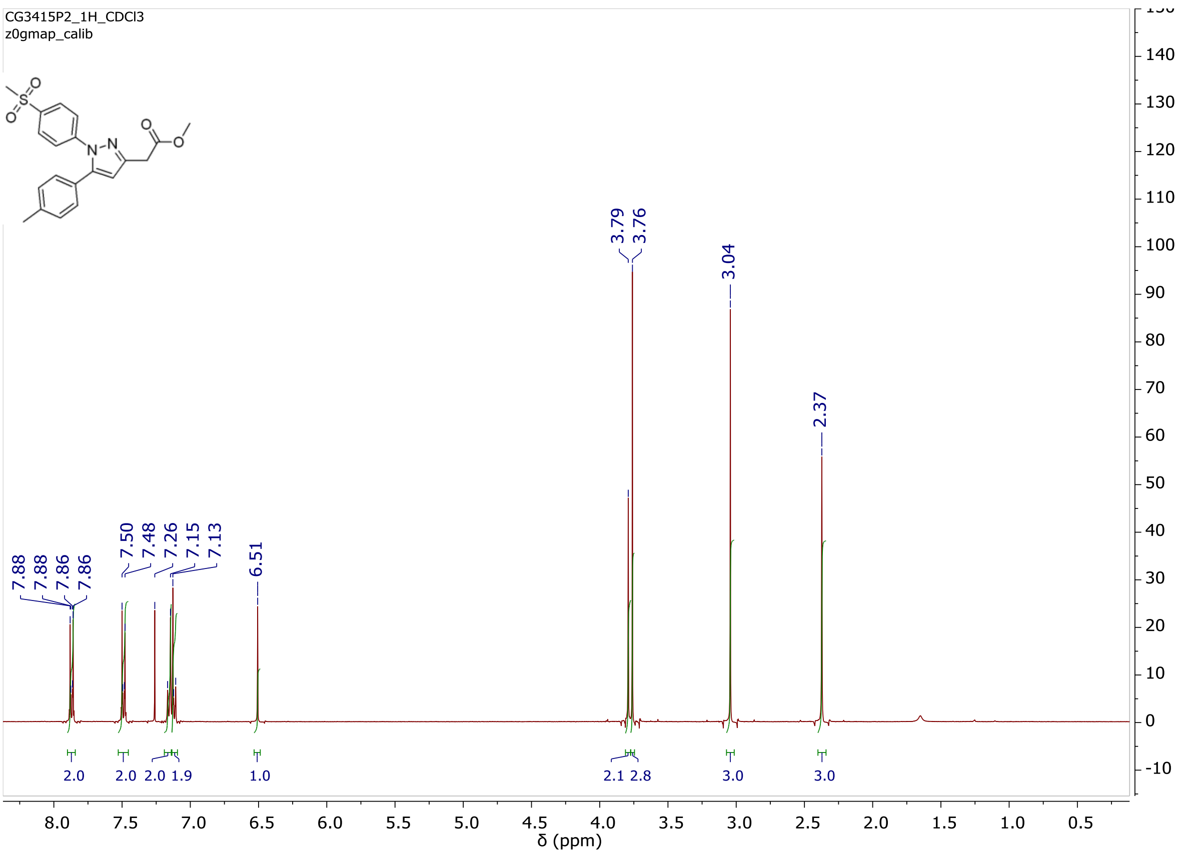

Figure S29. ^{1}H NMR spectrum of compound **2b** in $CDCl_3$

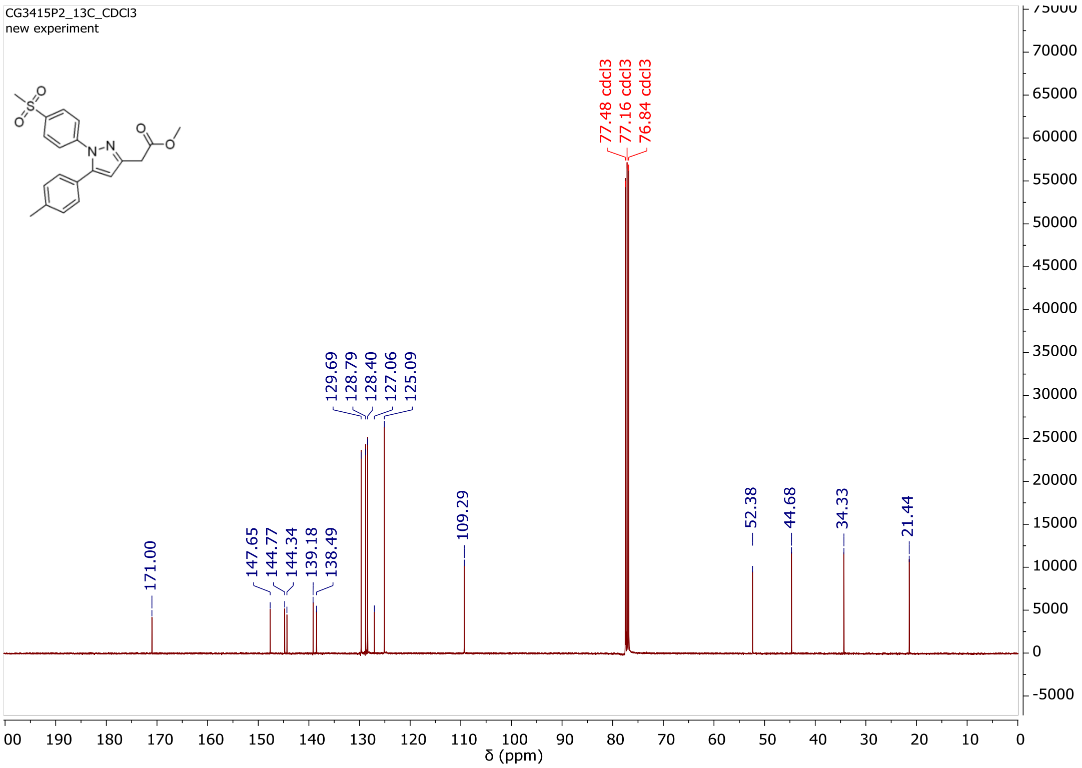

Figure S30. ^{13}C NMR spectrum of compound **2b** in $CDCl_3$

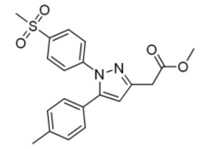

	f1 (ppm)	f2 (ppm)
1	129.7	7.14
2	128.8	7.13
3	128.4	7.87
4	125.1	7.48
5	109.3	6.50
6	52.4	3.76
7	44.7	3.04
8	34.4	3.78
9	21.4	2.37

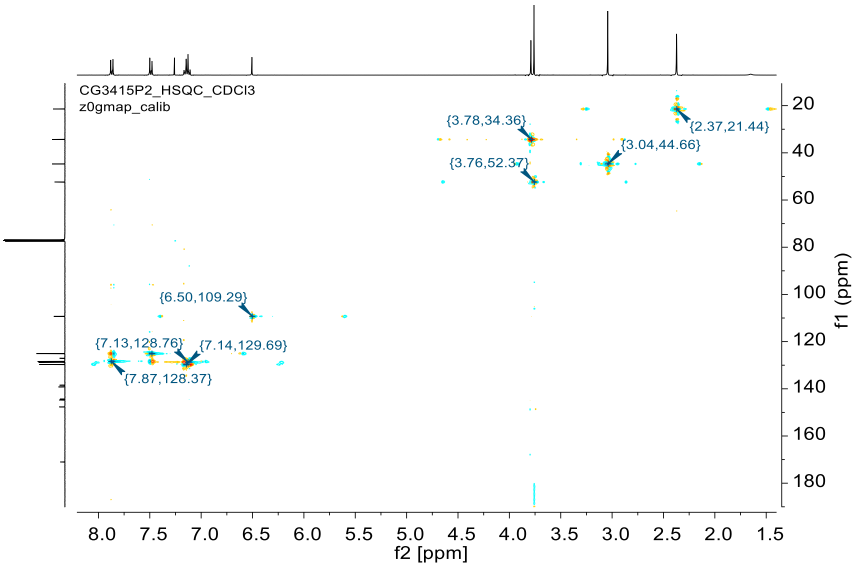

Figure S31. HSQC spectrum of compound **2b** in $CDCl_3$

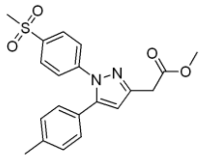

	f1 (ppm)	f2 (ppm)
1	171.0	3.75
2	171.0	3.79
3	147.7	6.50
4	147.7	3.79
5	144.8	6.51
6	144.8	7.12
7	144.4	7.87
8	144.3	7.48
9	139.2	2.37
10	139.2	7.12
11	138.5	7.48
12	129.7	2.37
13	129.7	7.13
14	128.8	7.12
15	128.4	7.87
16	127.1	7.12
17	127.1	7.16
18	125.1	7.87
19	125.1	7.48
20	109.3	3.79
21	21.4	7.12
22	21.4	7.16

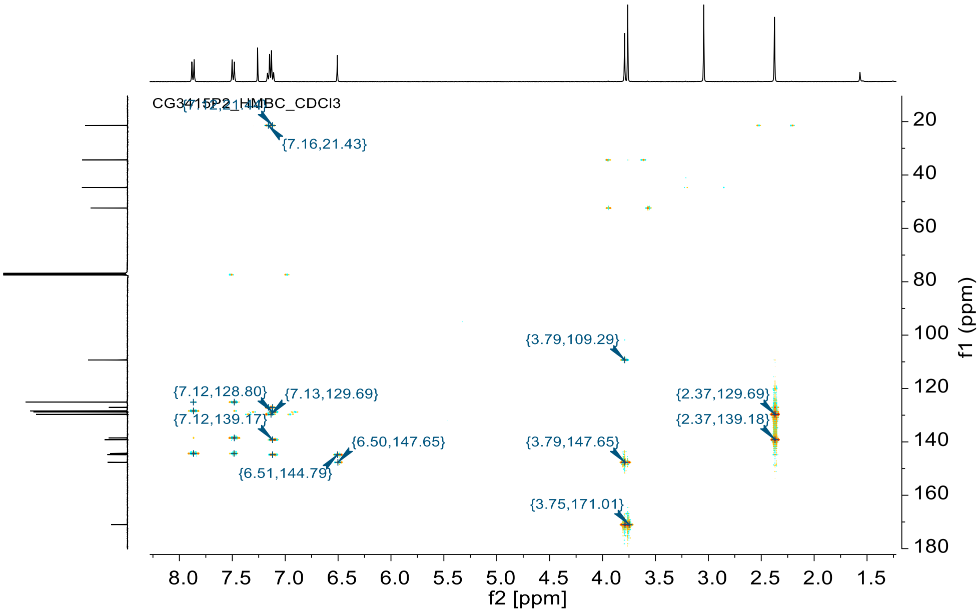

Figure S32. HMBC spectrum of compound **2b** in $CDCl_3$

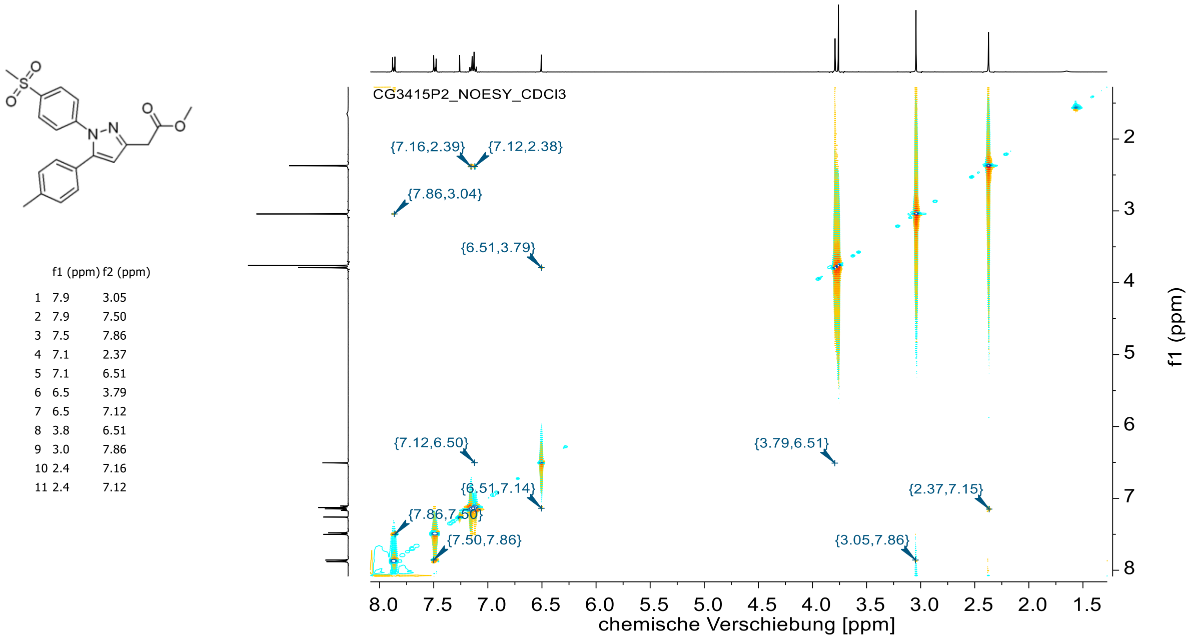

Figure S33. NOESY spectrum of compound **2b** in $CDCl_3$

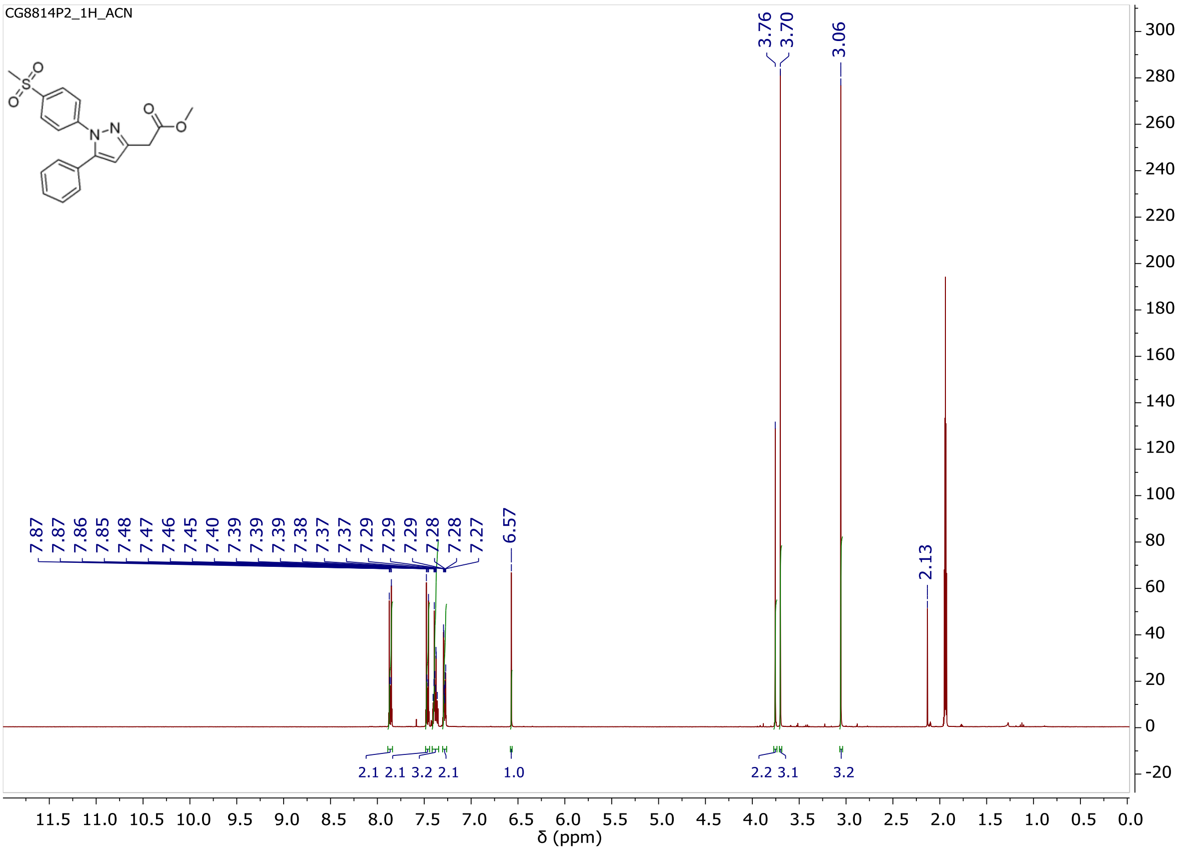

Figure S34. ^{1}H NMR spectrum of compound **2c** in CD_3CN

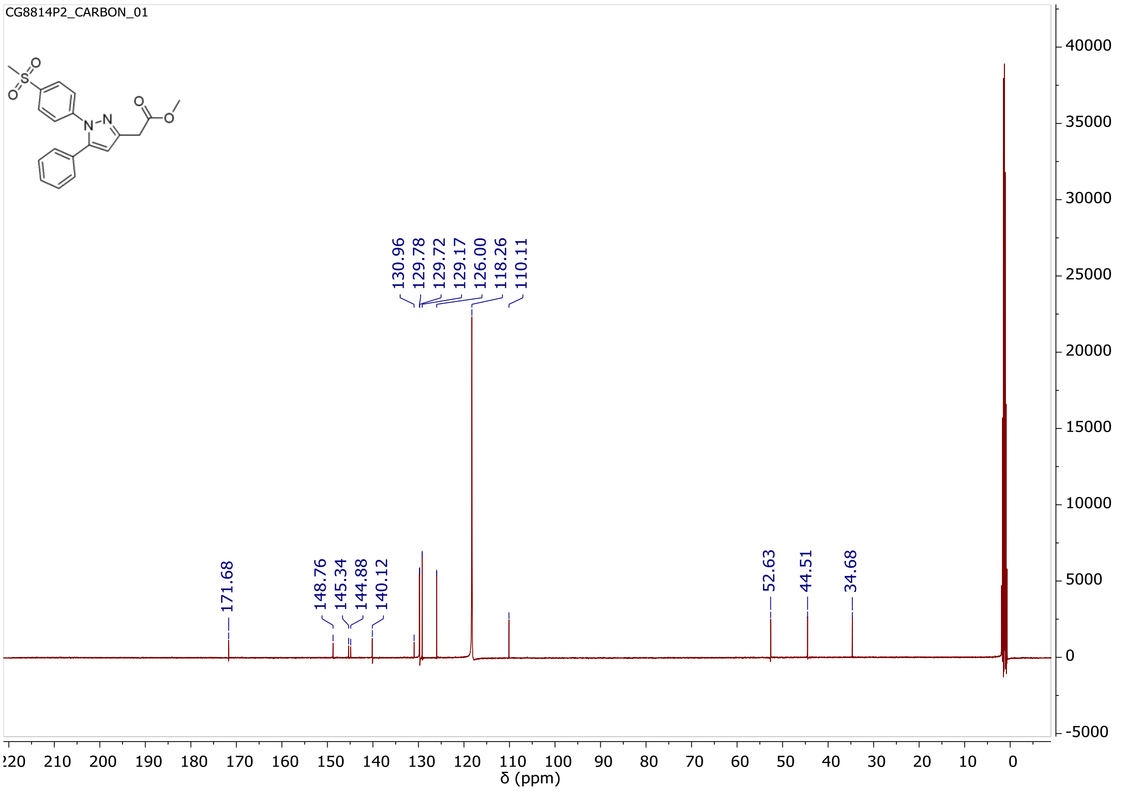

Figure S35. ^{13}C NMR spectrum of compound **2c** in CD_3CN

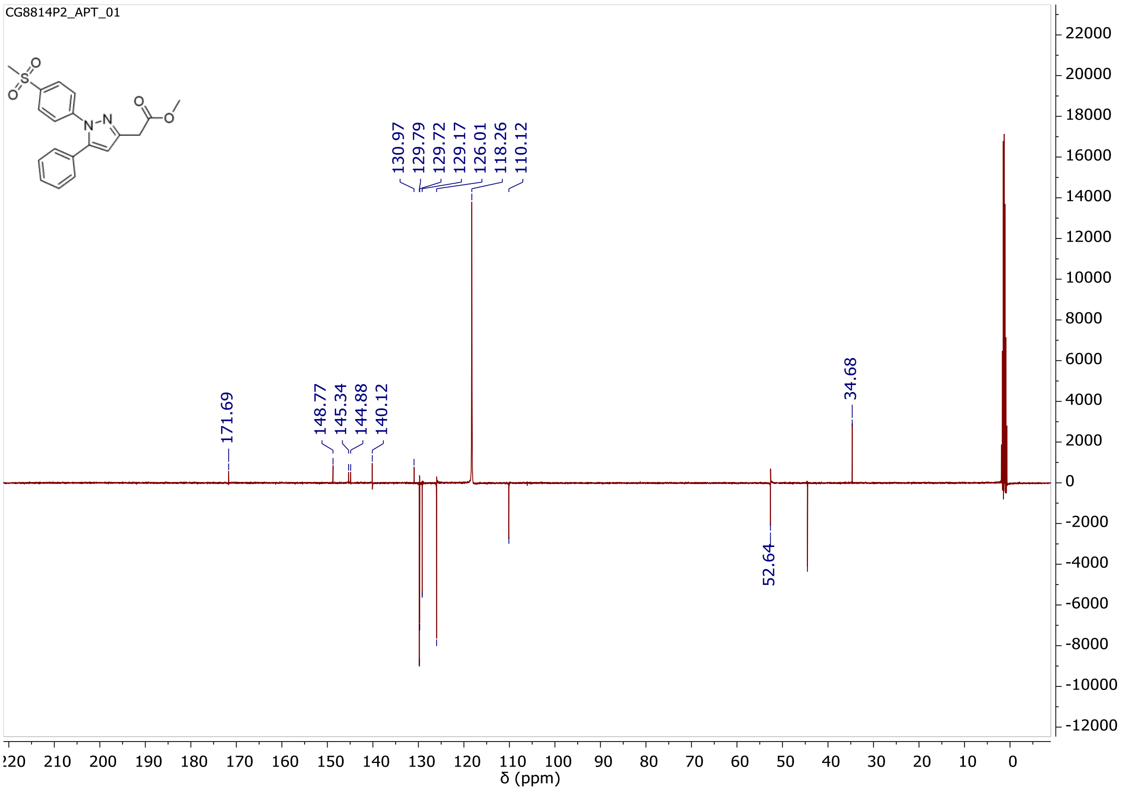

Figure S36. APT spectrum of compound **2c** in CD_3CN

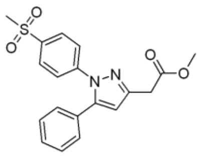

	f1 (ppm)	f2 (ppm)
1	129.2	7.87
2	126.0	7.48
3	129.7	7.39
4	129.8	7.30
5	110.1	6.58
6	34.7	3.76
7	52.6	3.71
8	44.5	3.06

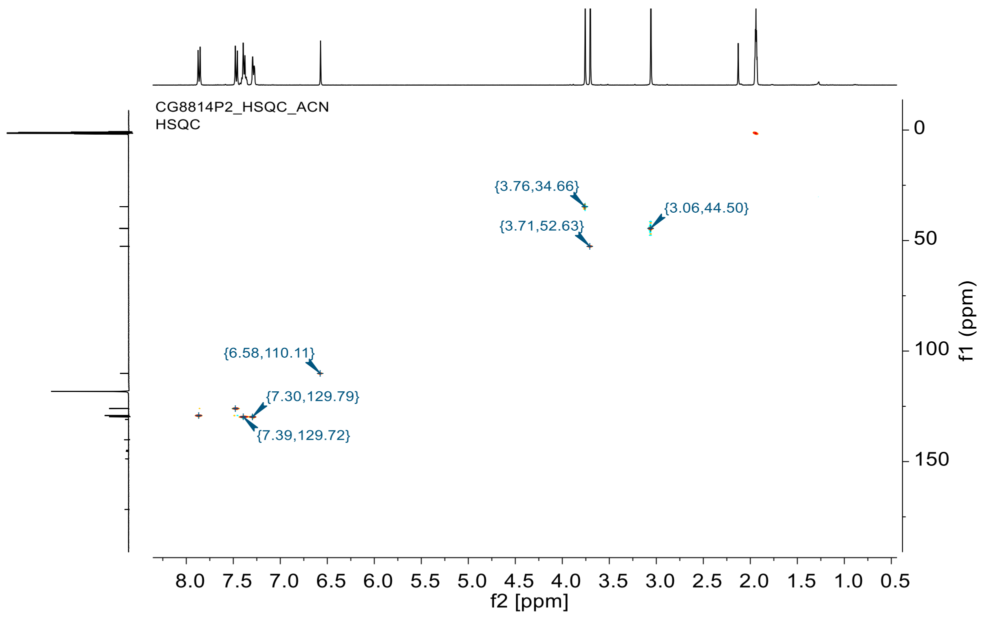

Figure S37. HSQC spectrum of compound **2c** in CD_3CN

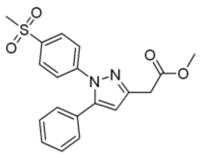

	f1 (ppm)	f2 (ppm)
1	129.2	7.87
2	144.9	7.87
3	144.9	7.47
4	140.1	7.47
5	126.0	7.47
6	129.6	7.40
7	131.0	7.39
8	130.9	7.30
9	145.3	7.30
10	129.8	7.28
11	131.0	6.57
12	148.8	6.57
13	145.3	6.57
14	148.8	3.77
15	110.1	3.77
16	171.7	3.73

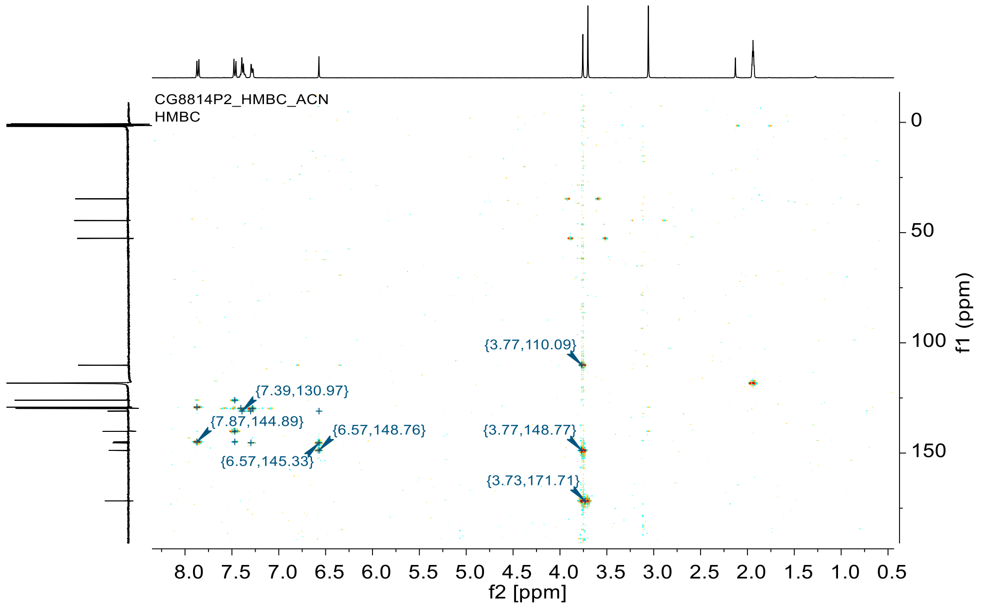

Figure S38. HMBC spectrum of compound **2c** in CD_3CN

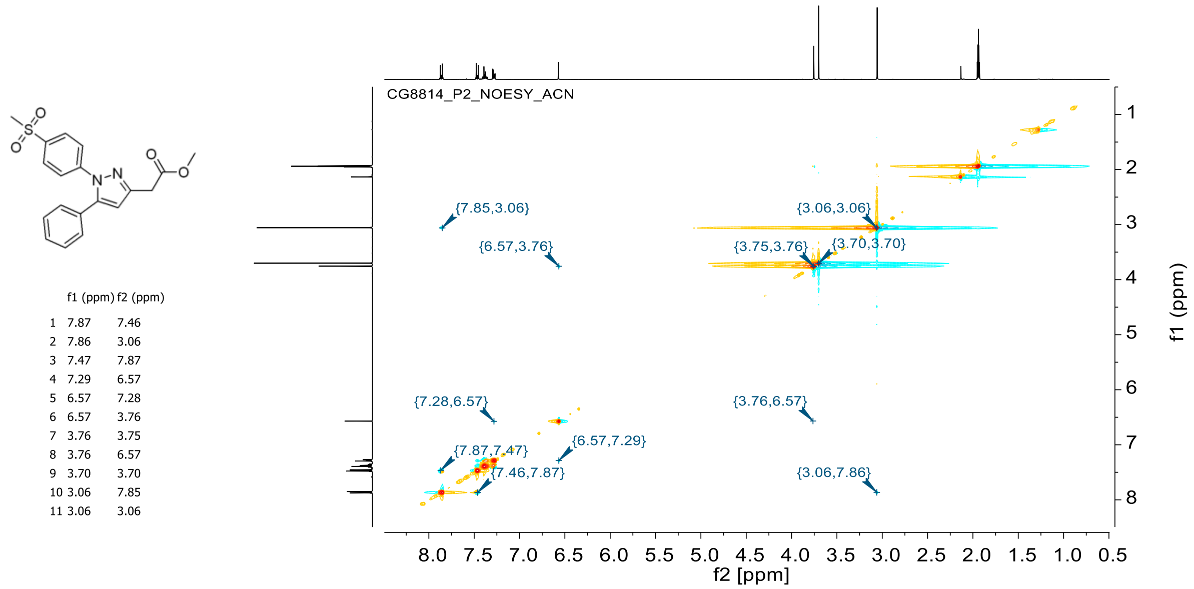

Figure S39. NOESY spectrum of compound **2c** in CD_3CN

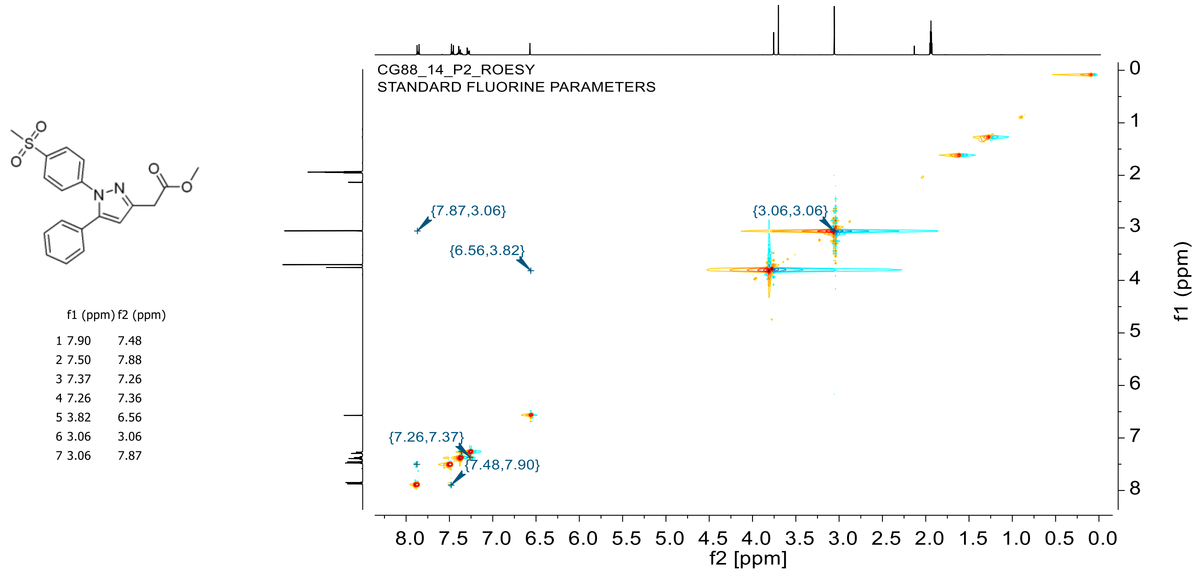

Figure S40. ROESY spectrum of compound **2c** in CD_3CN

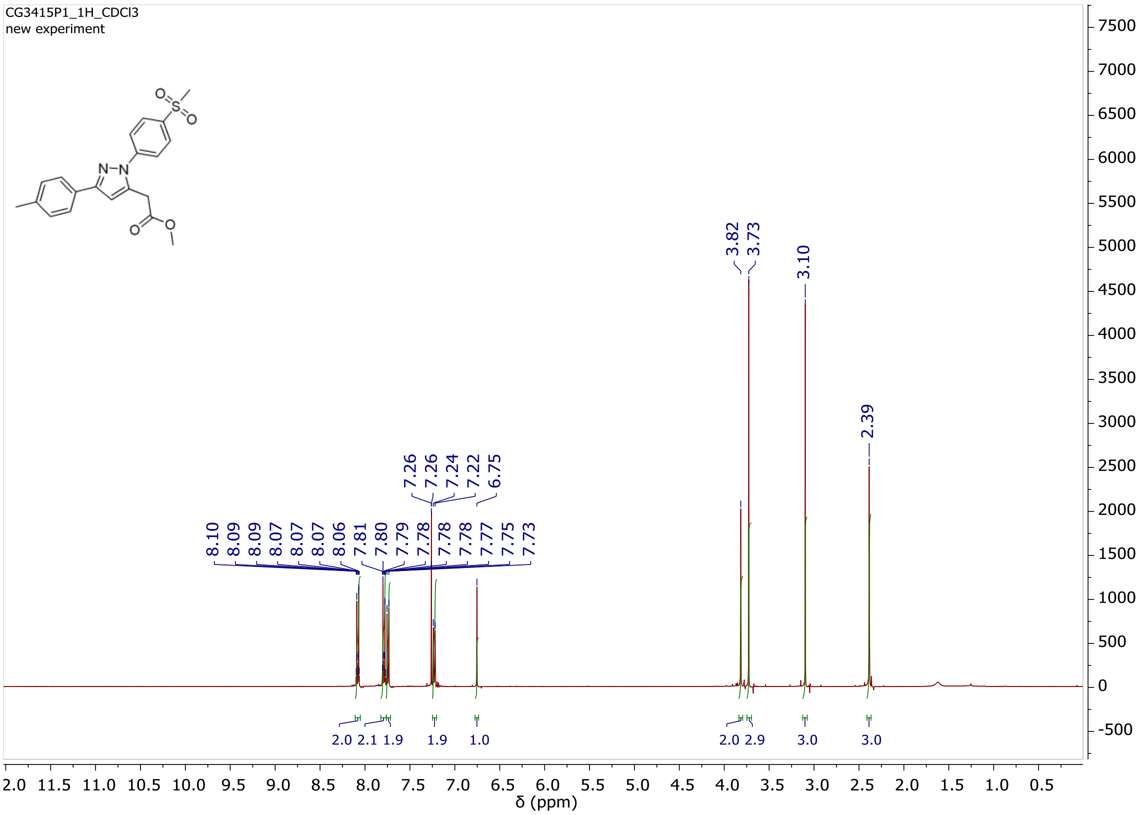

Figure S41. ^{1}H NMR spectrum of compound **2d** in $CDCl_3$

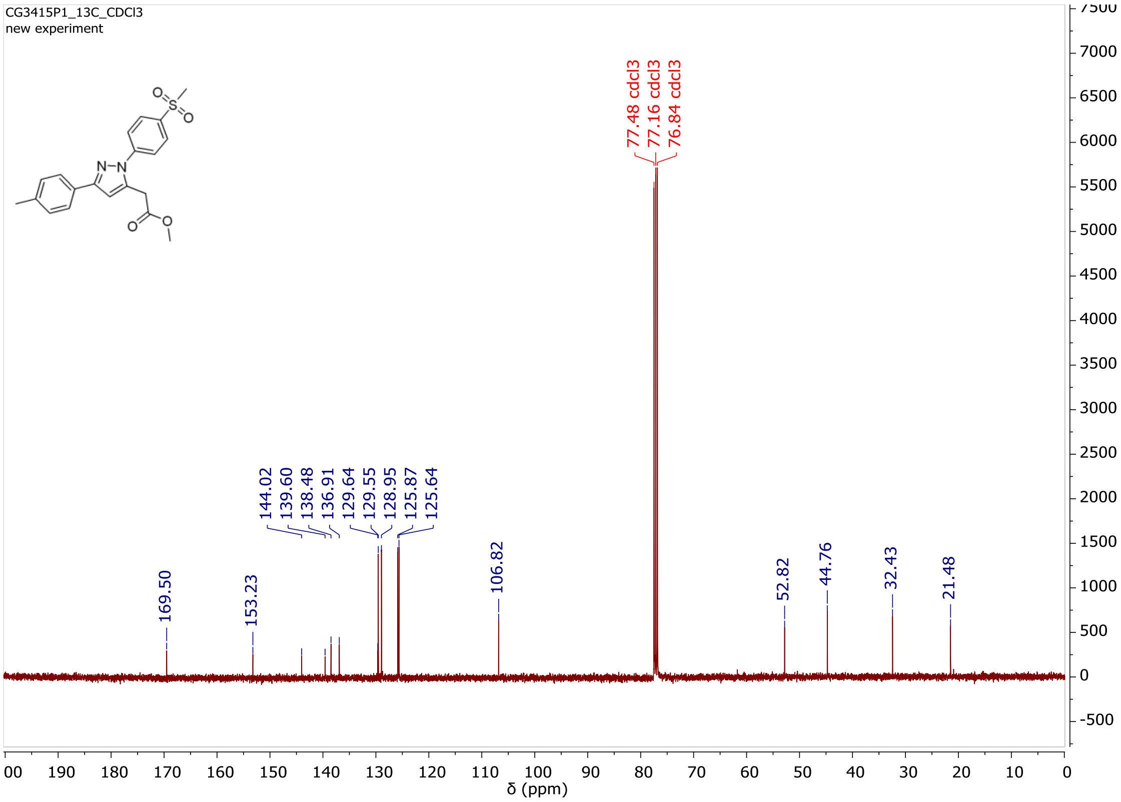

Figure S42. ^{13}C NMR spectrum of compound **2d** in $CDCl_3$

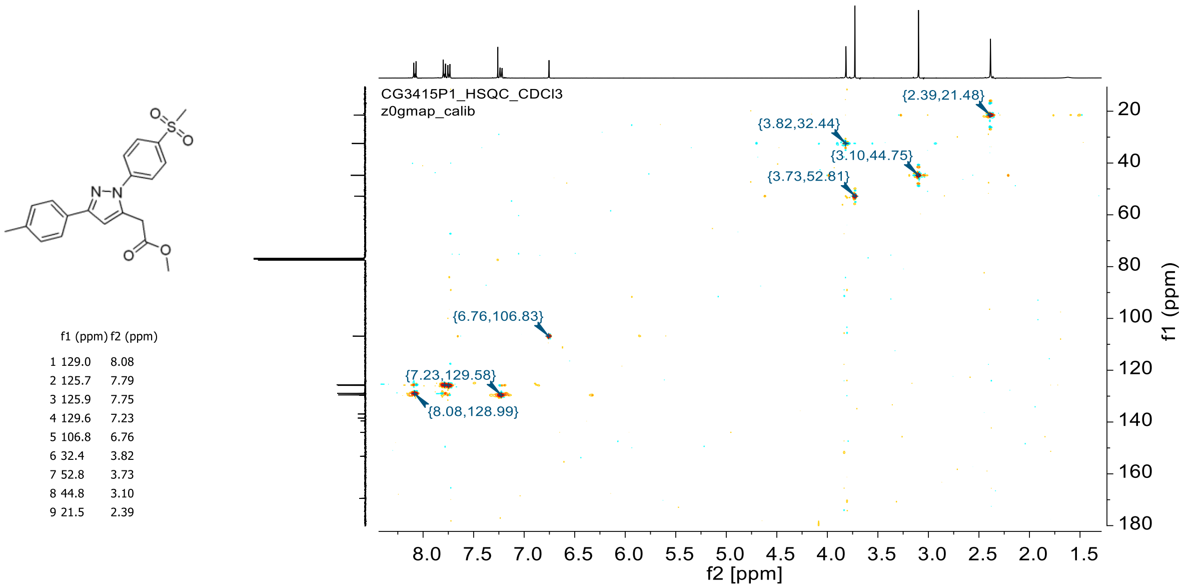

f1 (ppm)	f2 (ppm)
1 129.0	8.08
2 125.7	7.79
3 125.9	7.75
4 129.6	7.23
5 106.8	6.76
6 32.4	3.82
7 52.8	3.73
8 44.8	3.10
9 21.5	2.39

Figure S43. HSQC spectrum of compound **2d** in $CDCl_3$

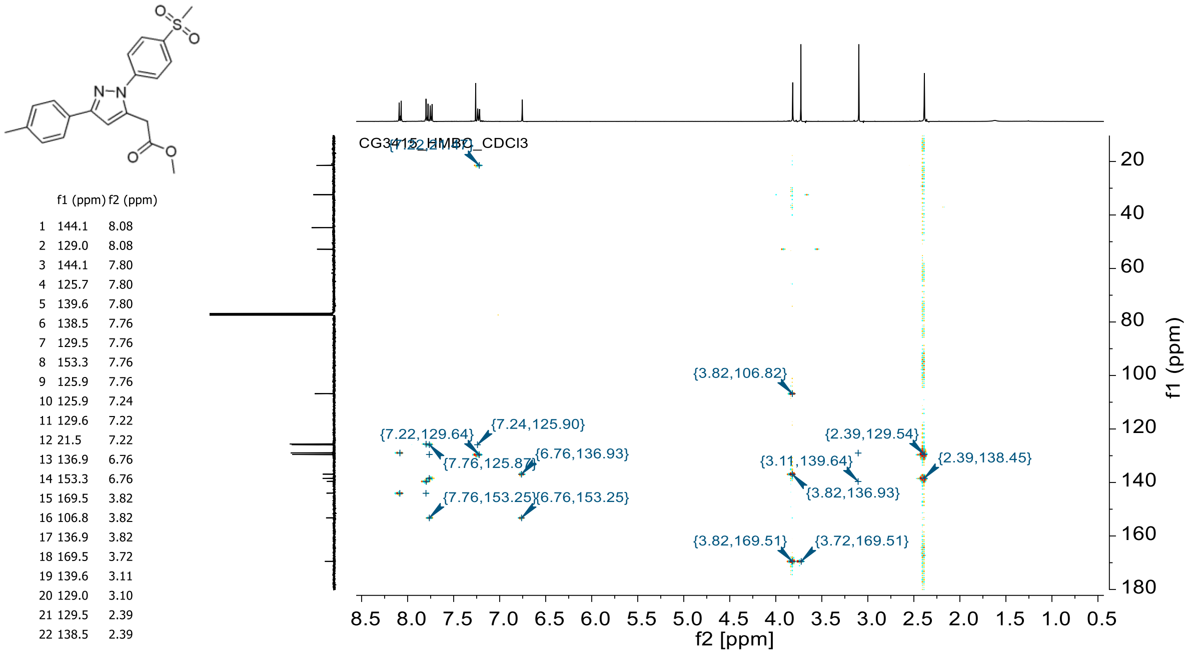

	f1 (ppm)	f2 (ppm)
1	144.1	8.08
2	129.0	8.08
3	144.1	7.80
4	125.7	7.80
5	139.6	7.80
6	138.5	7.76
7	129.5	7.76
8	153.3	7.76
9	125.9	7.76
10	125.9	7.24
11	129.6	7.22
12	21.5	7.22
13	136.9	6.76
14	153.3	6.76
15	169.5	3.82
16	106.8	3.82
17	136.9	3.82
18	169.5	3.72
19	139.6	3.11
20	129.0	3.10
21	129.5	2.39
22	138.5	2.39

Figure S44. HMBC spectrum of compound **2d** in $CDCl_3$

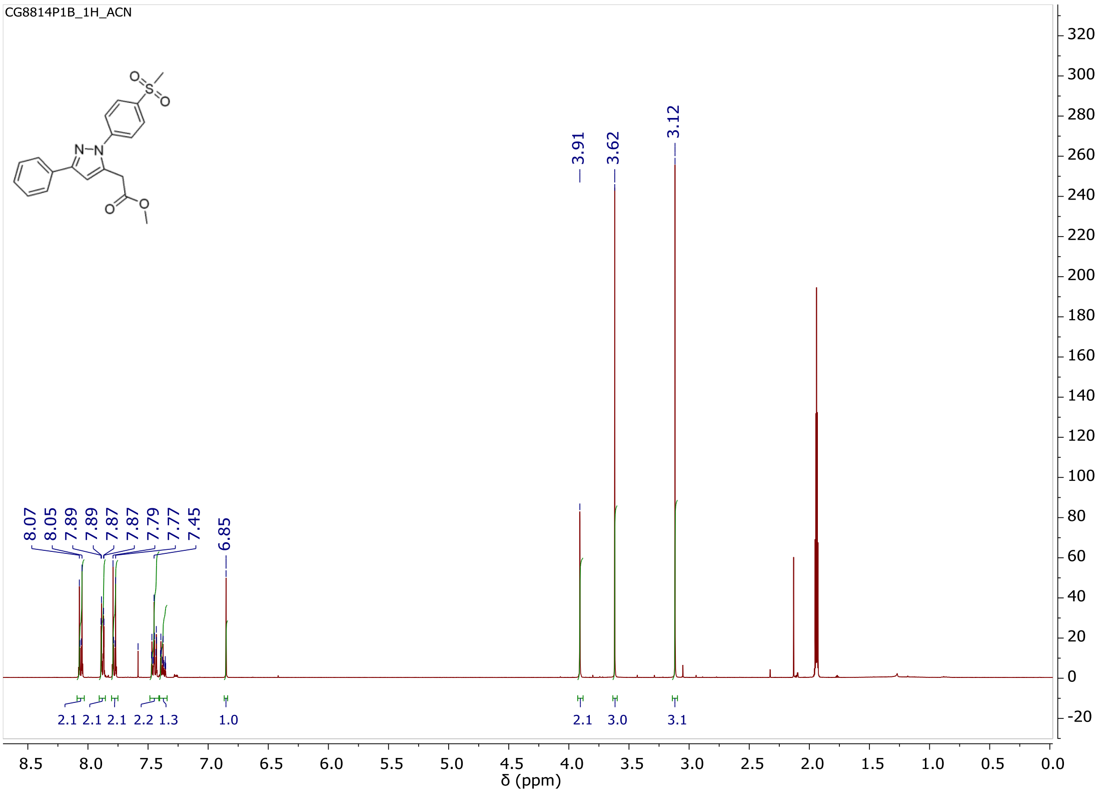

Figure S45. ^{1}H NMR spectrum of compound **2e** in CD_3CN

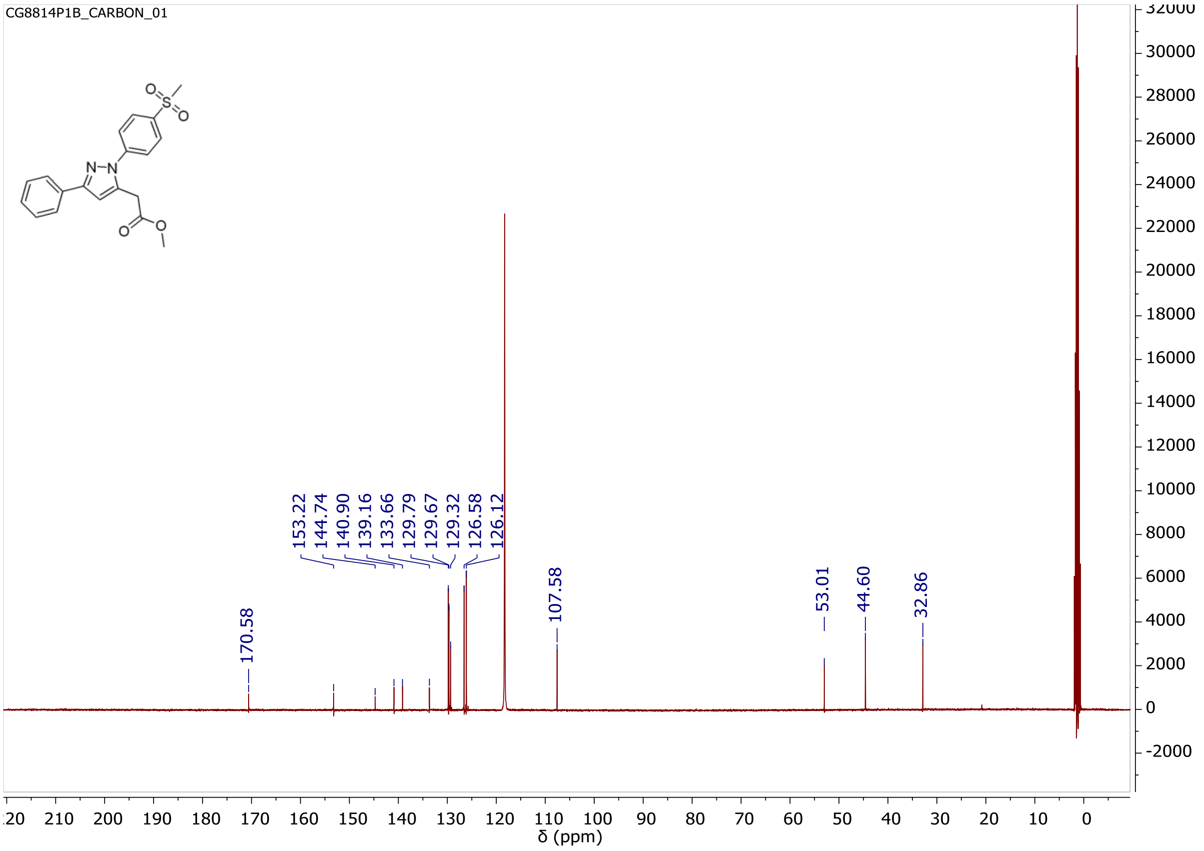

Figure S46. ^{13}C NMR spectrum of compound **2e** in CD_3CN

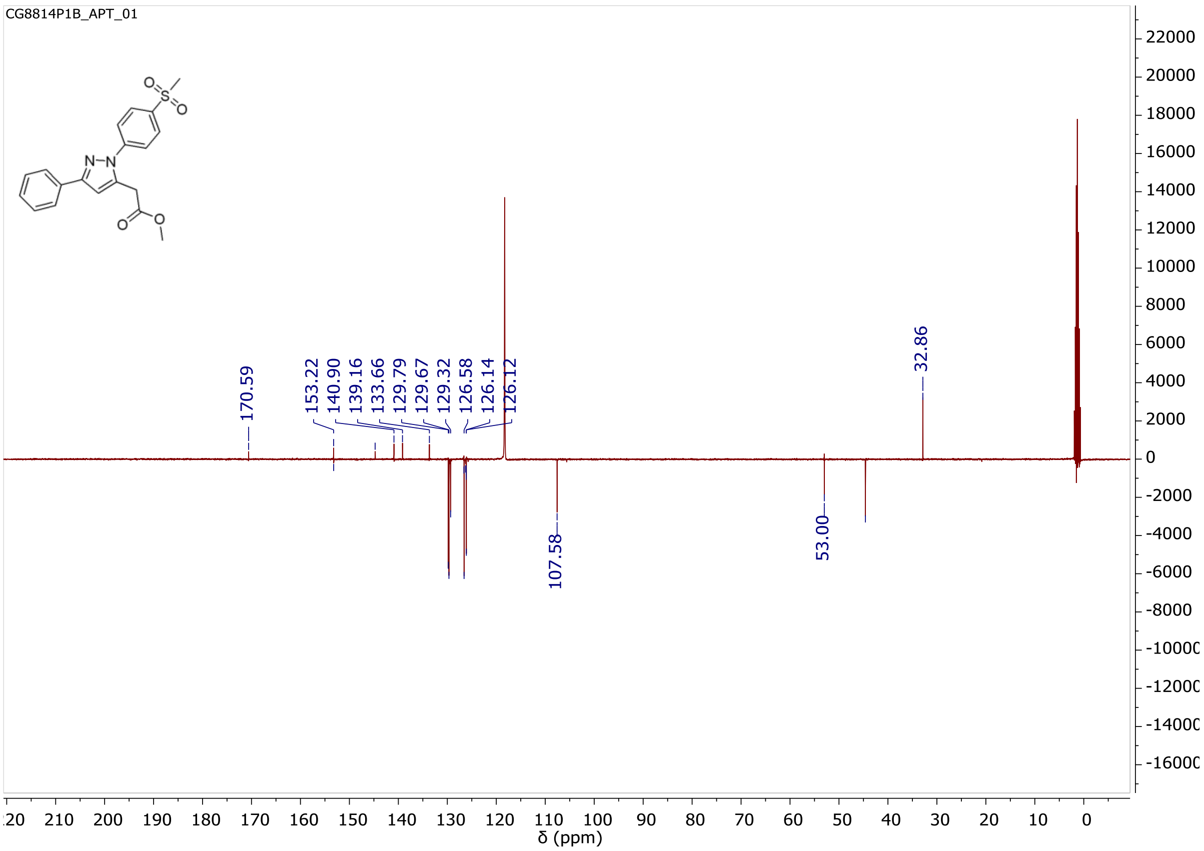

Figure S47. APT spectrum of compound **2e** in CD_3CN

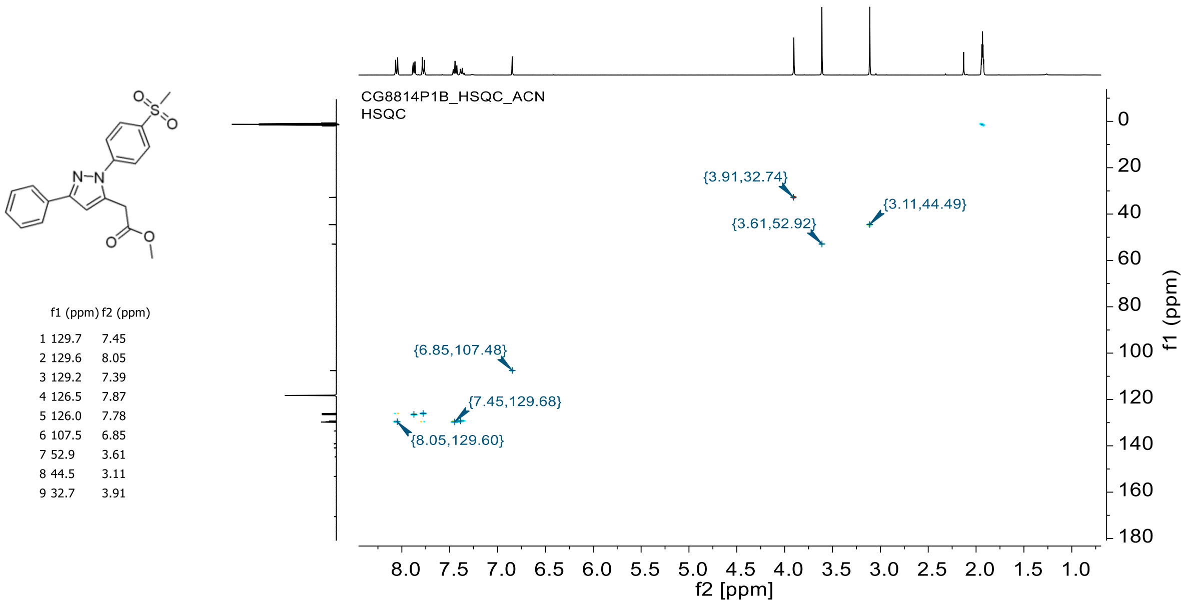

Figure S48. HSQC spectrum of compound **2e** in CD_3CN

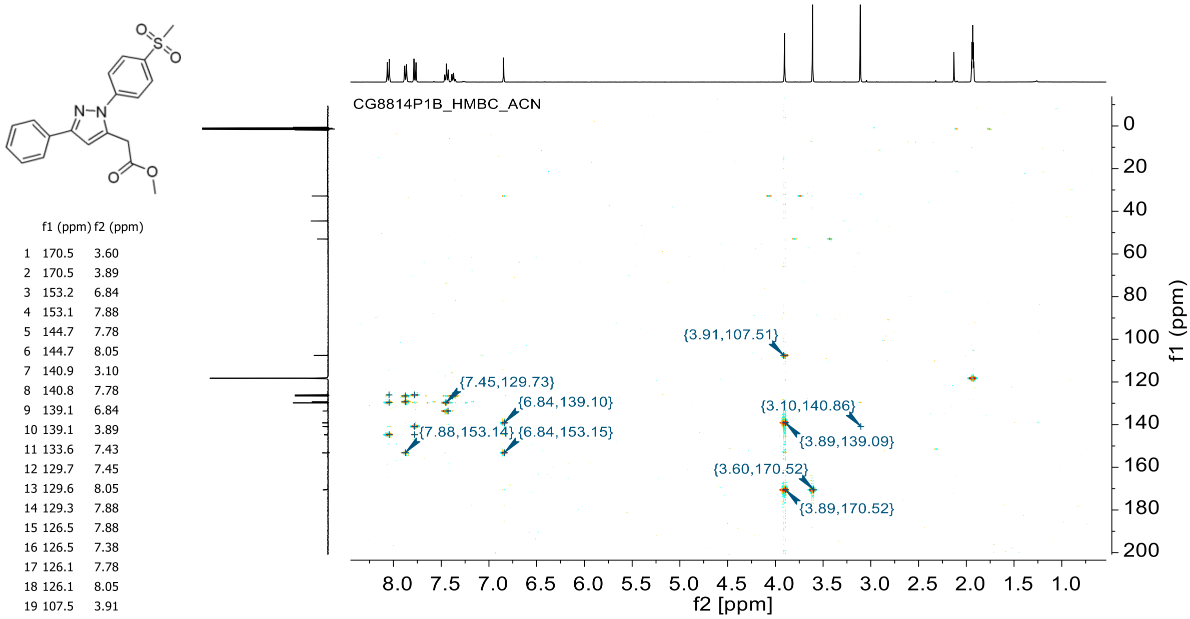

Figure S49. HMBC spectrum of compound **2e** in CD_3CN

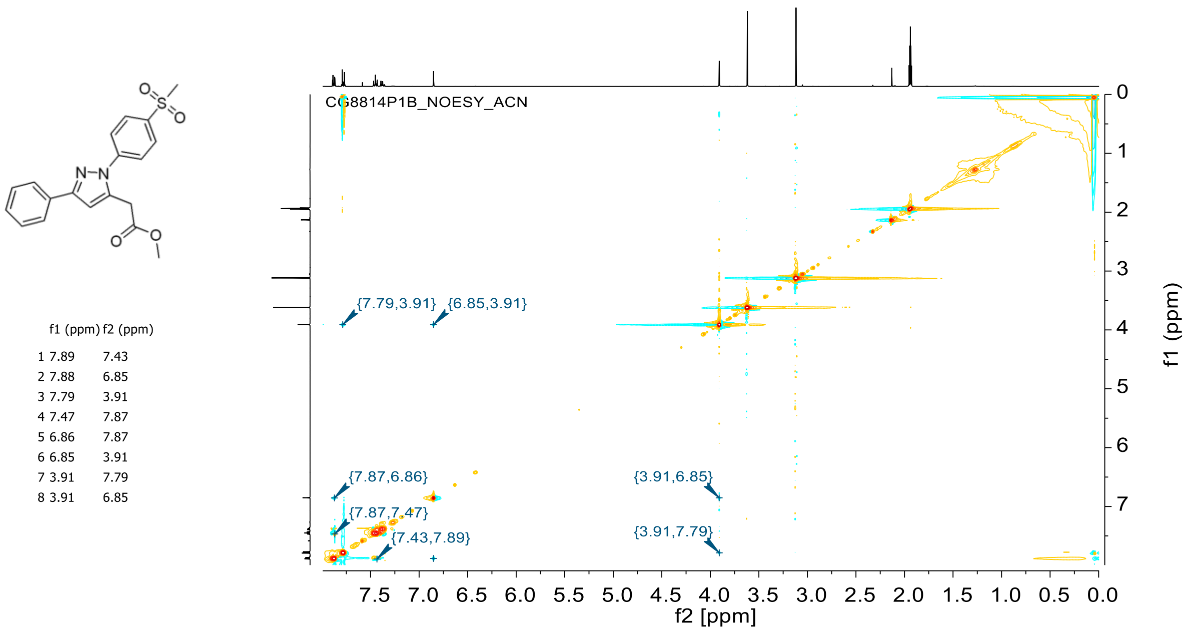

Figure S50. NOESY spectrum of compound **2e** in CD_3CN

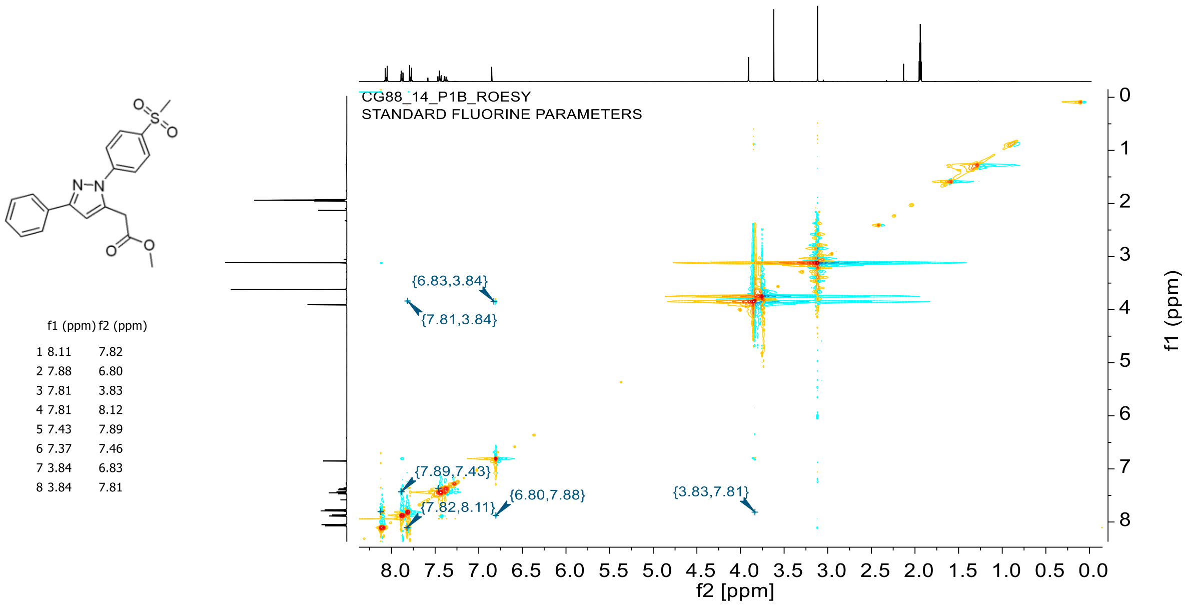

Figure S51. ROESY spectrum of compound **2e** in CD_3CN

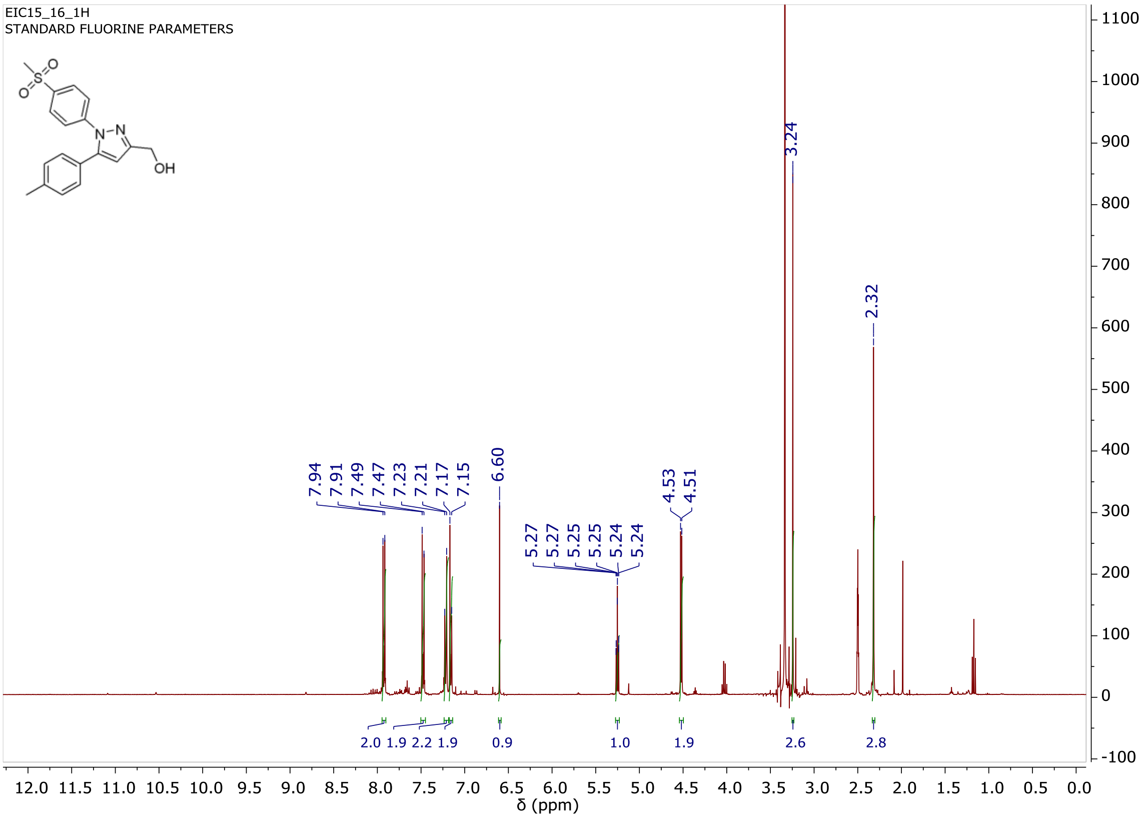

Figure S52. ^{1}H NMR spectrum of compound **3a** in DMSO-d_6

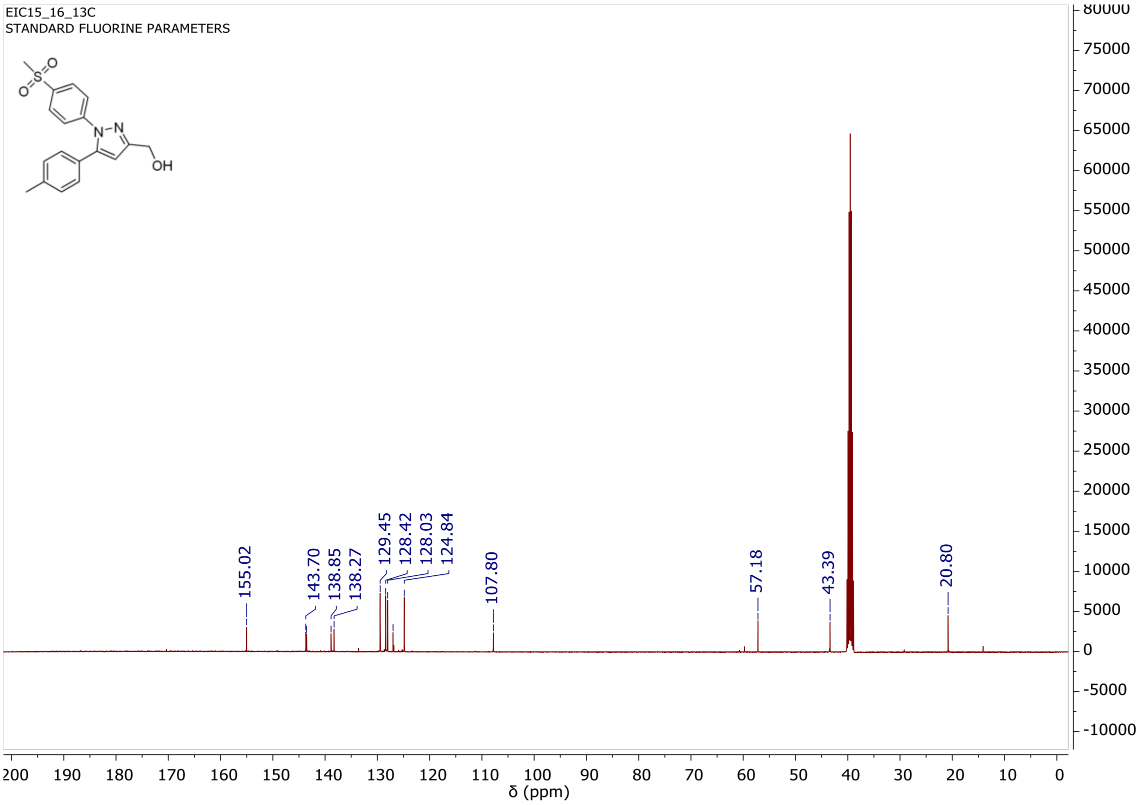

Figure S53. ^{13}C NMR spectrum of compound **3a** in DMSO-*d*$_6$

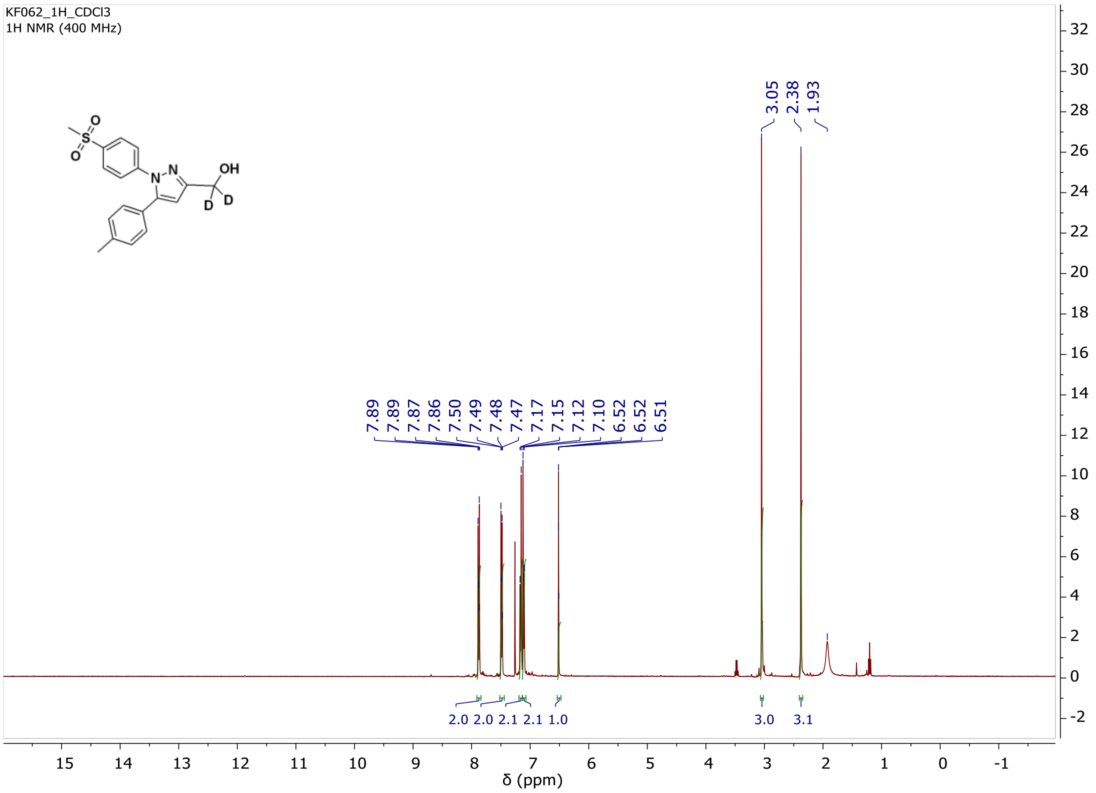

Figure S54. ^{1}H NMR spectrum of compound [D$_2$]**3a** in $CDCl_3$

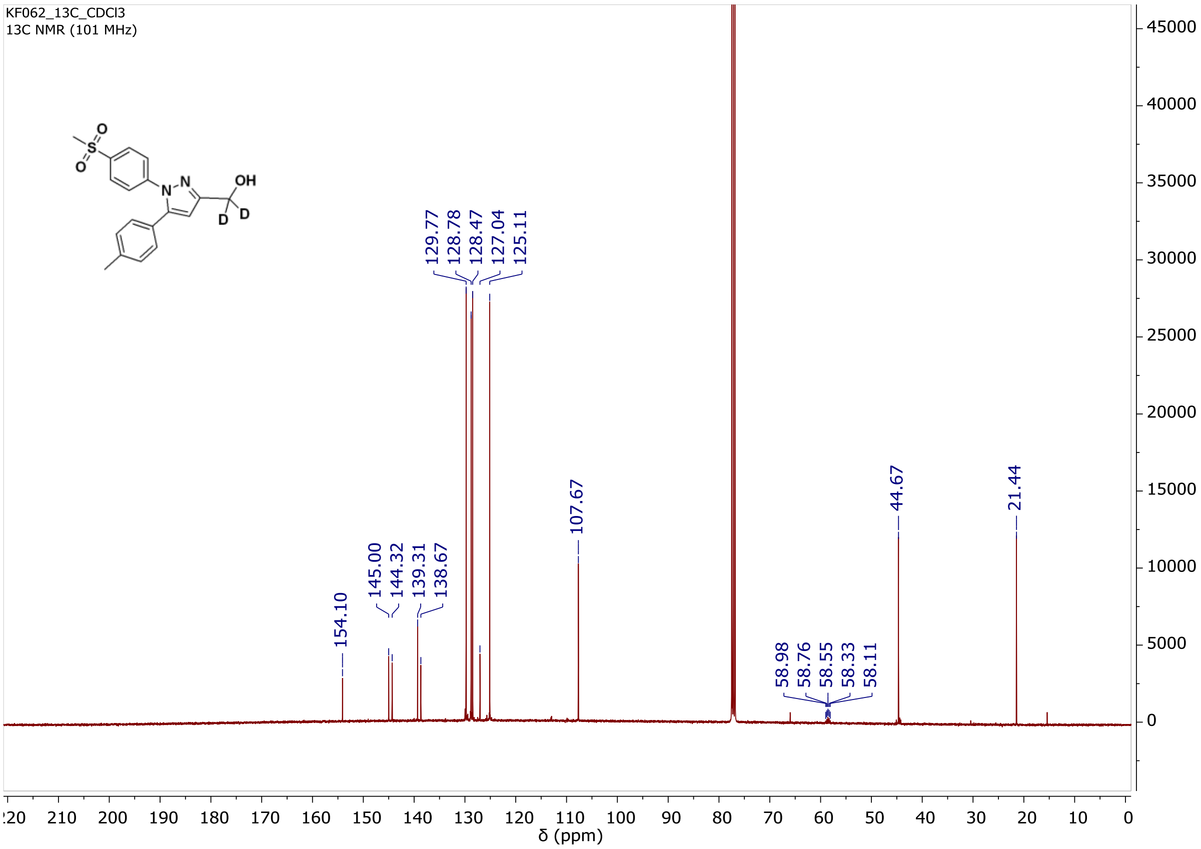

Figure S55. ^{13}C NMR spectrum of compound [D_2]**3a** in $CDCl_3$

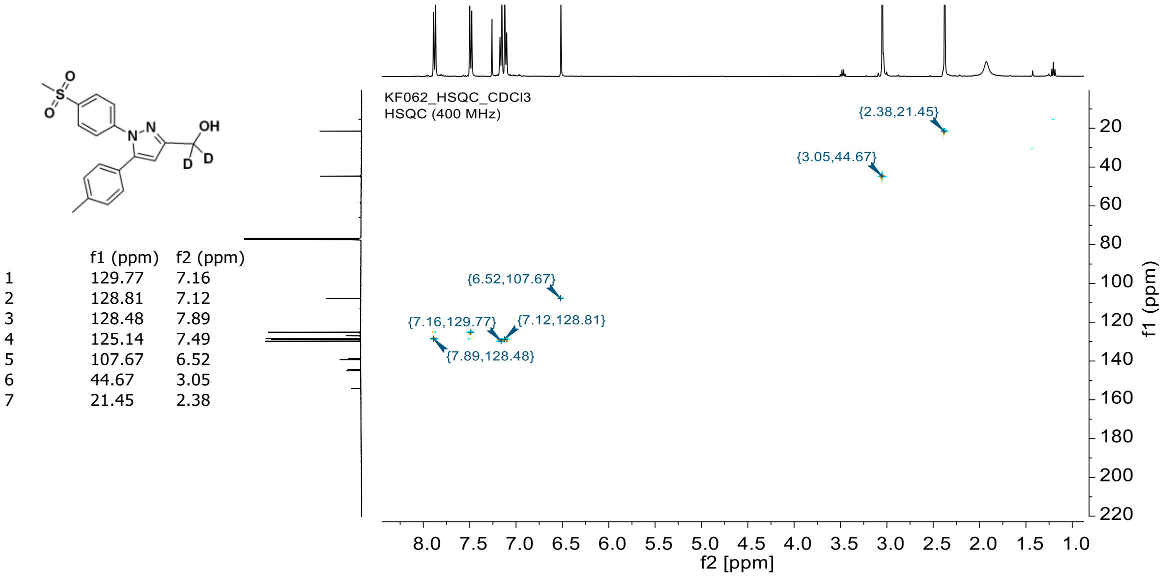

Figure S56. HSQC spectrum of compound [D_2]**3a** in $CDCl_3$

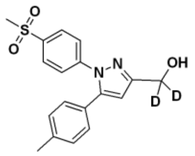

	f1 (ppm)	f2 (ppm)
1	154.08	6.52
2	145.00	6.52
3	145.00	7.11
4	144.32	7.89
5	144.31	7.48
6	139.33	7.11
7	139.33	2.38
8	138.68	3.06
9	138.68	7.49
10	129.77	7.16
11	129.76	2.38
12	128.80	7.11
13	128.49	3.05
14	128.47	7.88
15	127.03	7.17
16	125.12	7.50
17	125.09	7.88
18	21.46	7.17

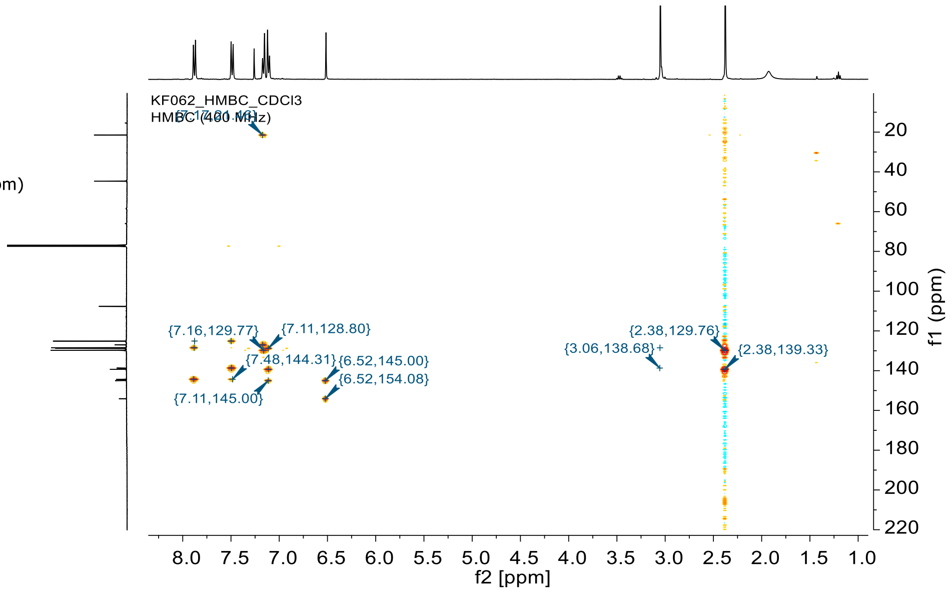

Figure S57. HMBC spectrum of compound [D_2]**3a** in $CDCl_3$

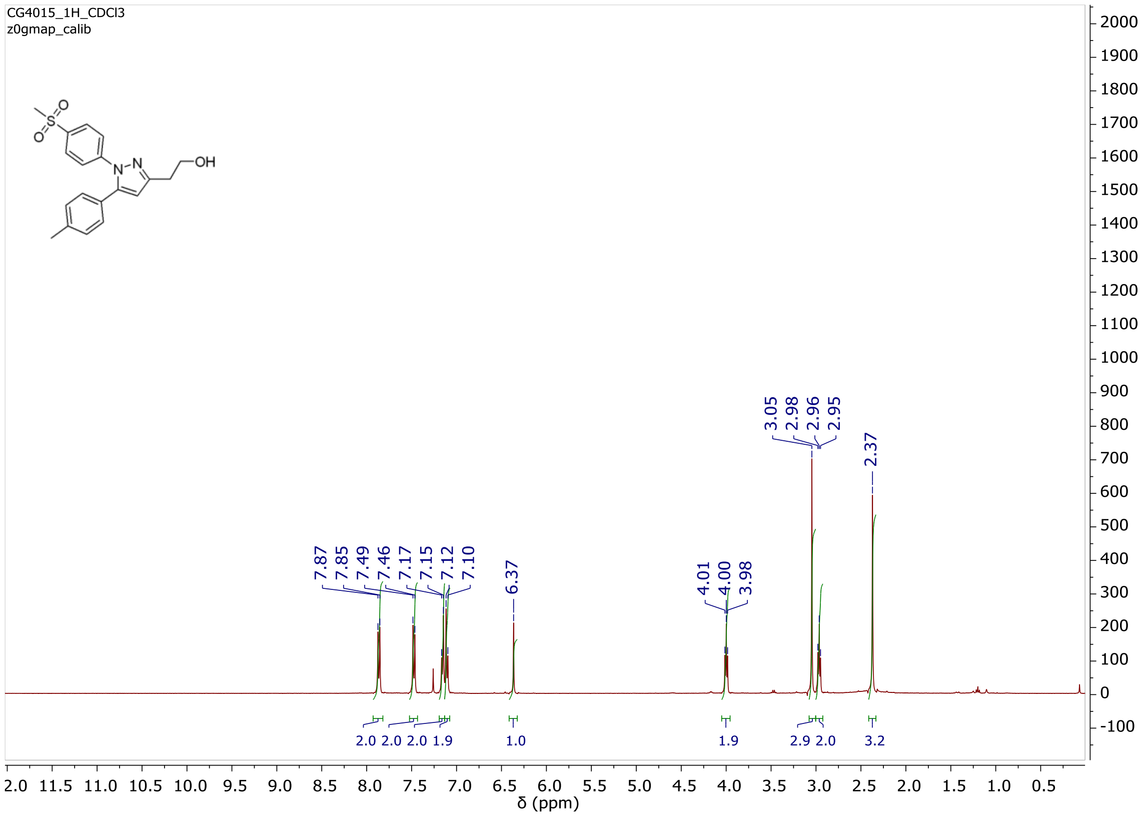

Figure S58. ^{1}H NMR spectrum of compound **3b** in $CDCl_3$

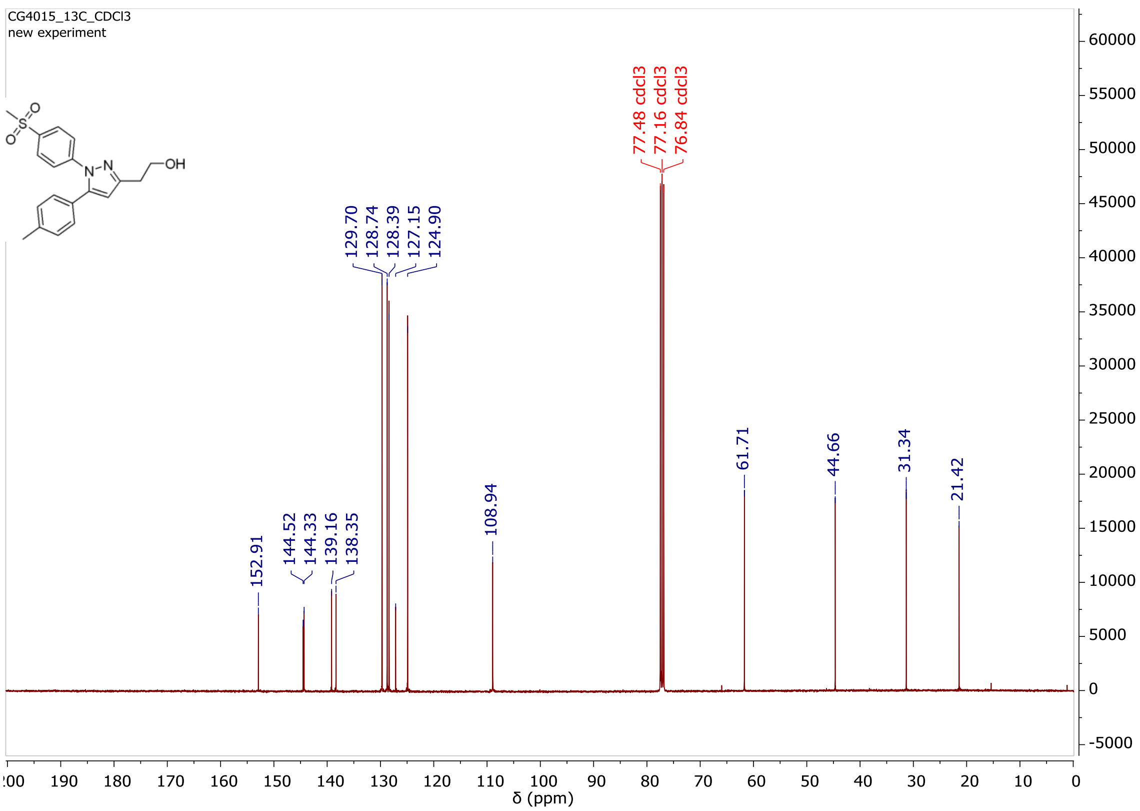

Figure S59. ^{13}C NMR spectrum of compound **3b** in $CDCl_3$

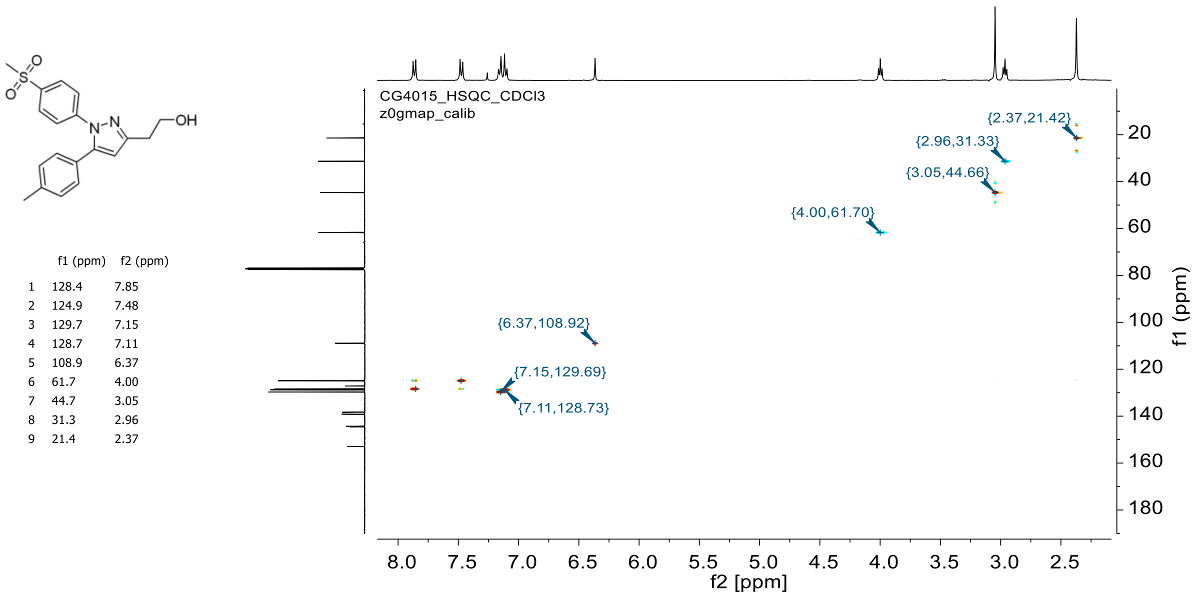

Figure S60. HSQC spectrum of compound **3b** in $CDCl_3$

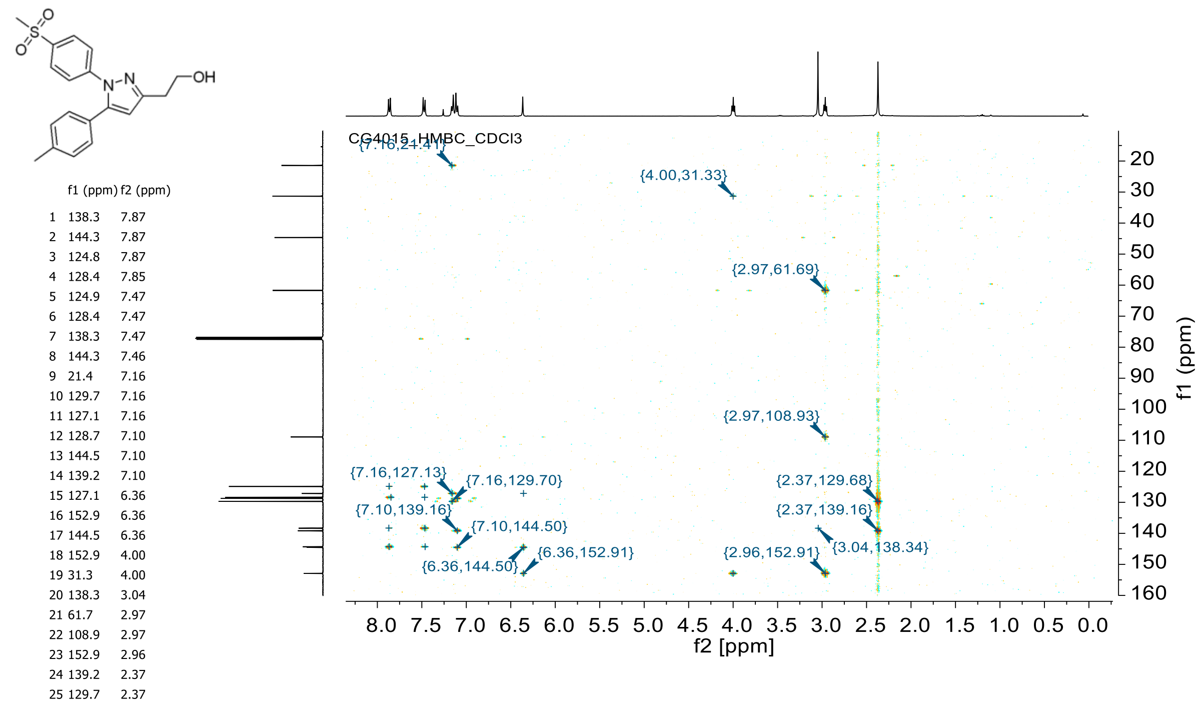

Figure S61. HMBC spectrum of compound **3b** in $CDCl_3$

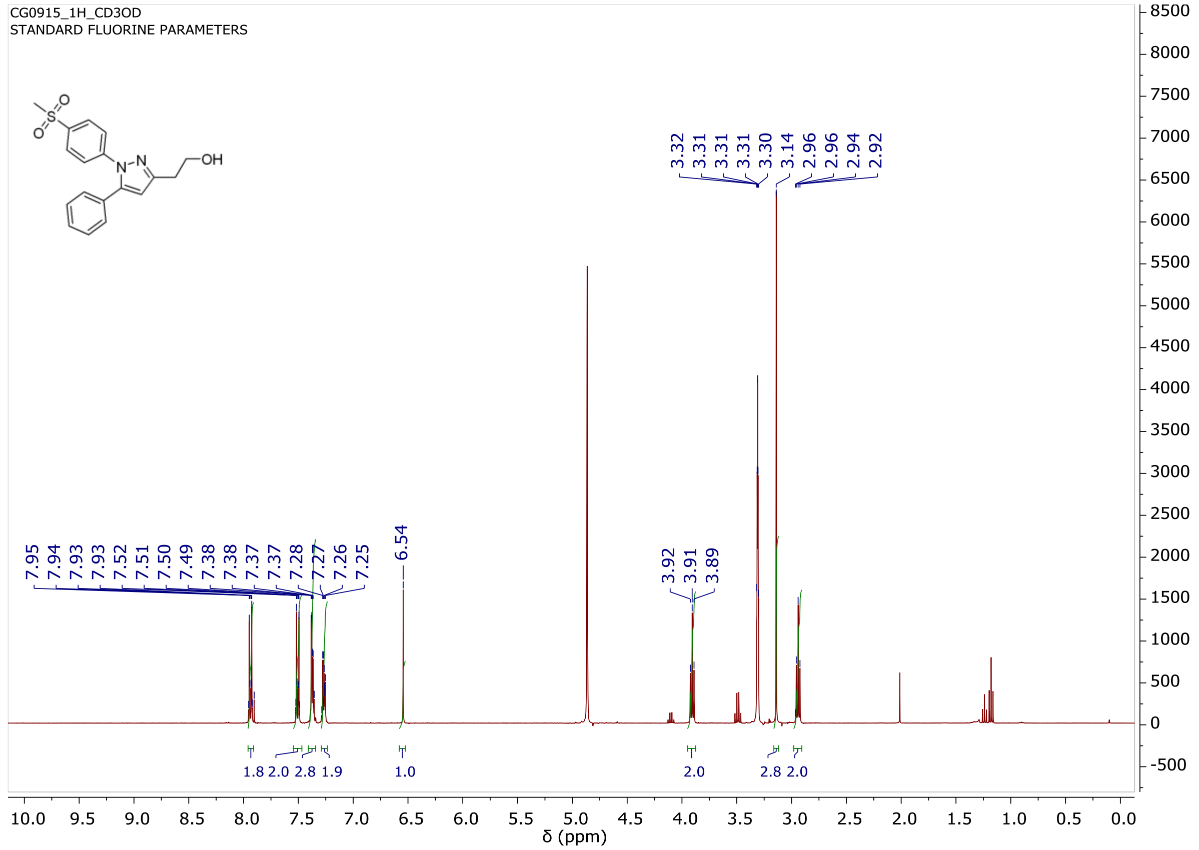

Figure S62. ^{1}H NMR spectrum of compound **3c** in CD_3OD

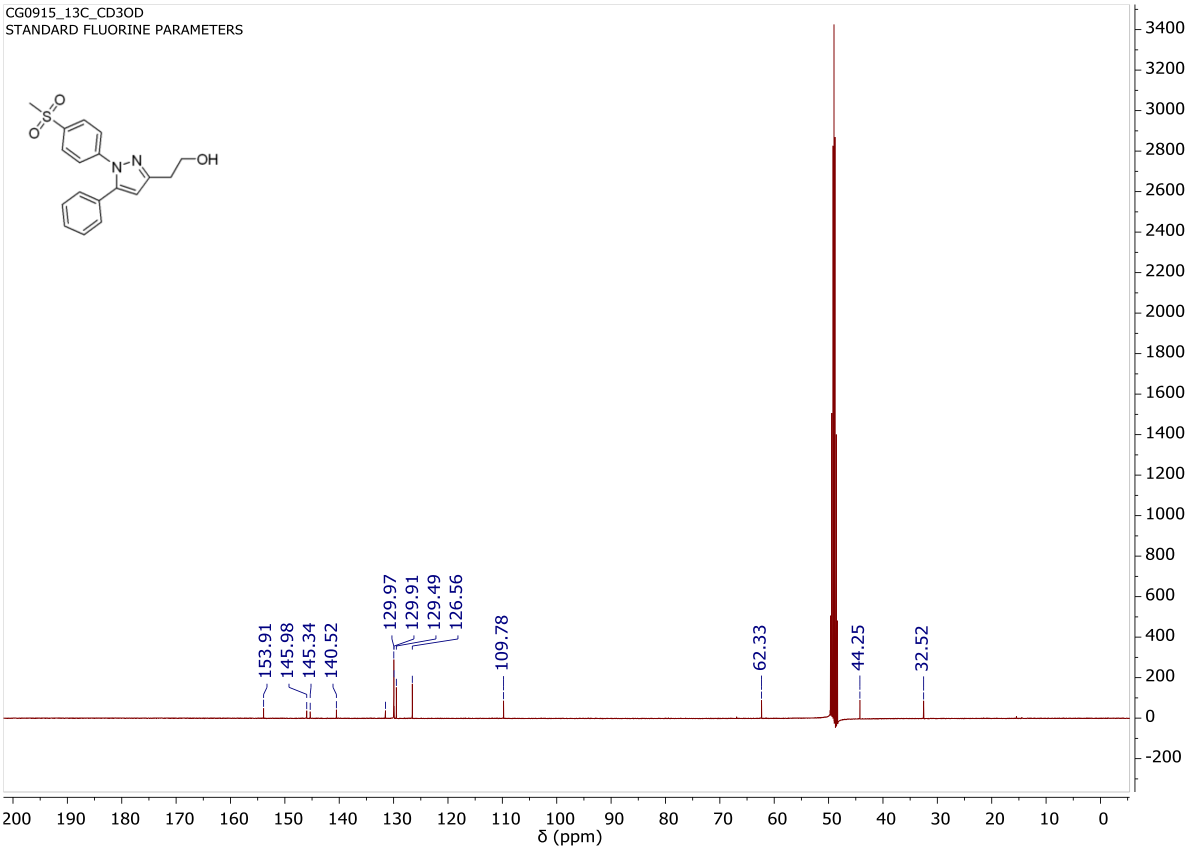

Figure S63. ^{13}C NMR spectrum of compound **3c** in CD_3OD

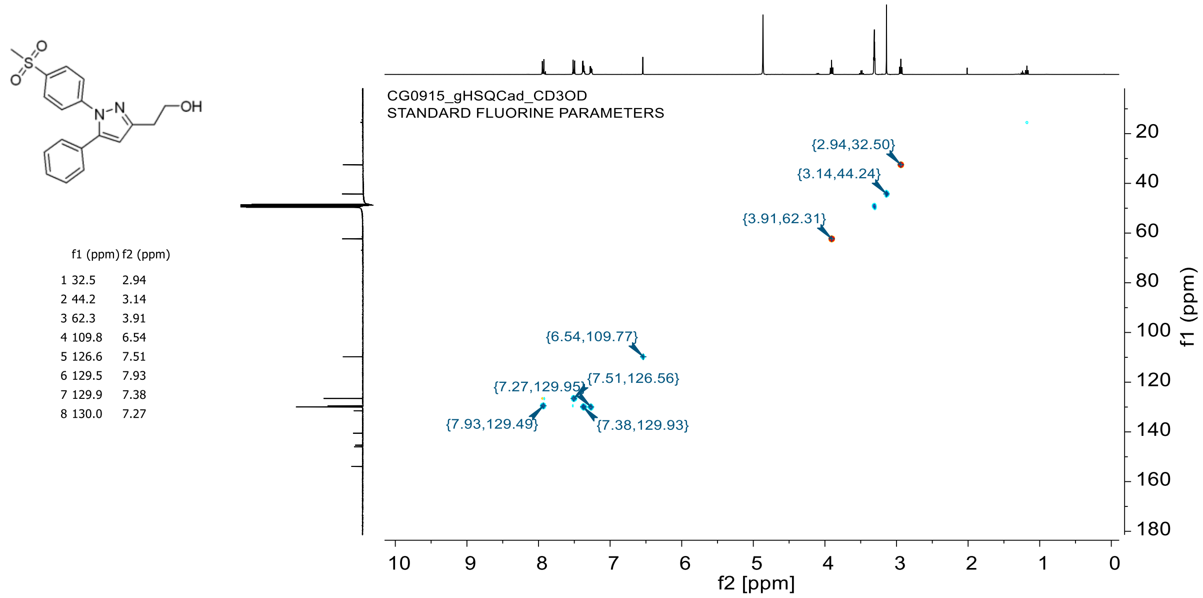

Figure S64. HSQC spectrum of compound **3c** in CD_3OD

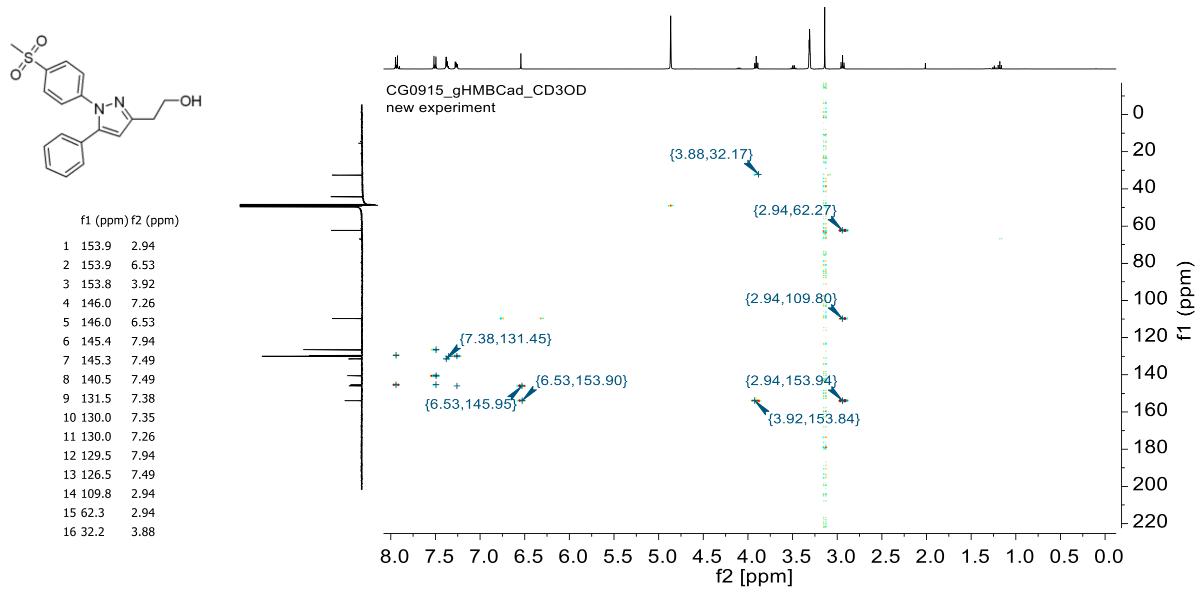

Figure S65. HMBC spectrum of compound **3c** in CD_3OD

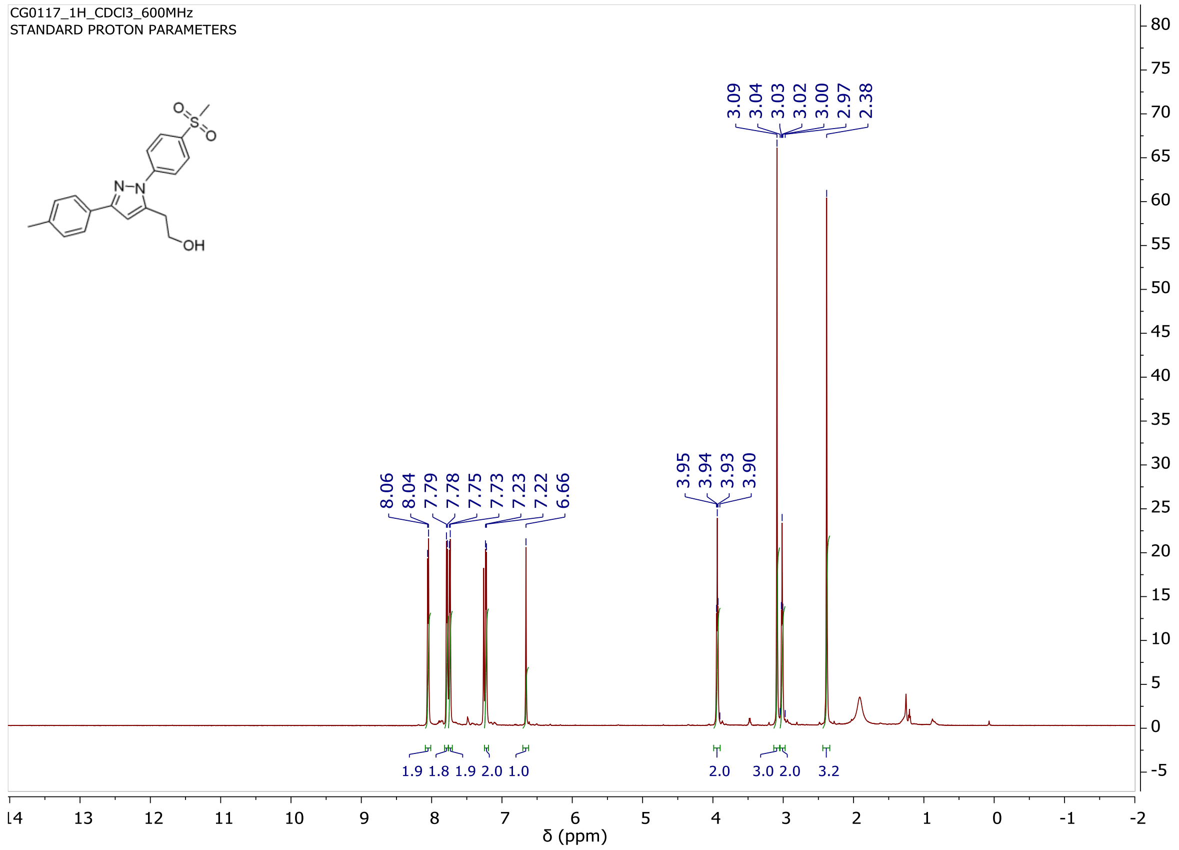

Figure S66. ^{1}H NMR spectrum of compound **3d** in $CDCl_3$

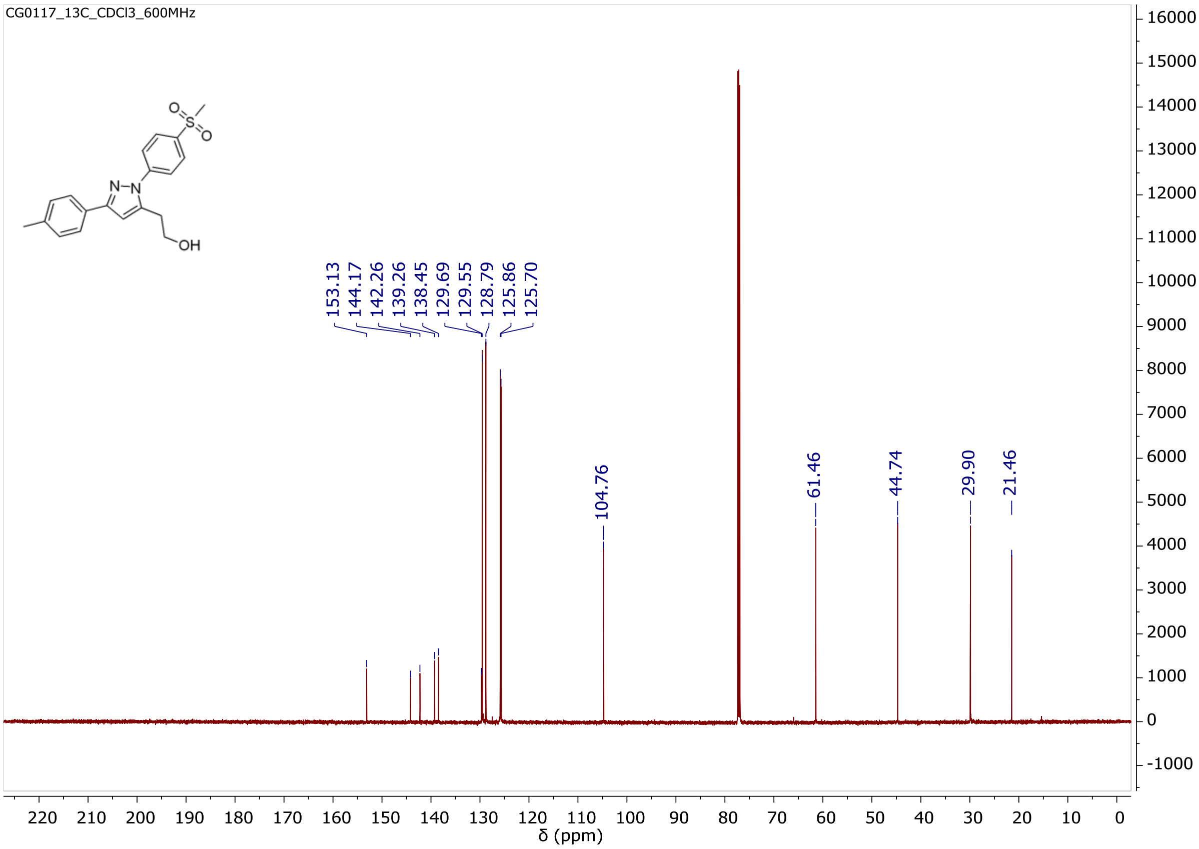

Figure S67. ^{13}C NMR spectrum of compound **3d** in $CDCl_3$

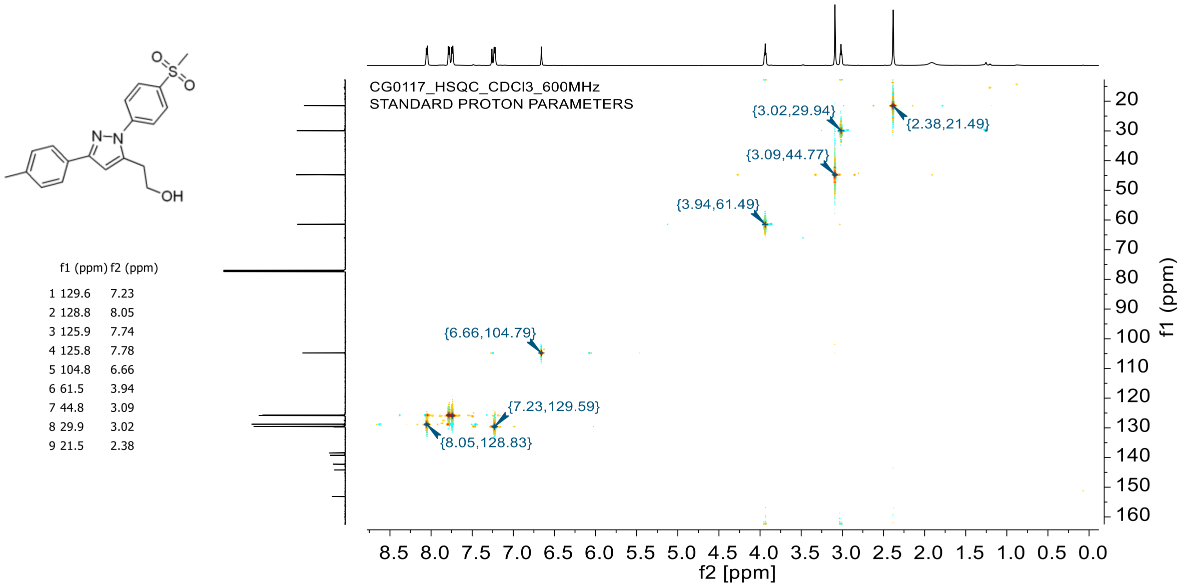

Figure S68. HSQC spectrum of compound **3d** in $CDCl_3$

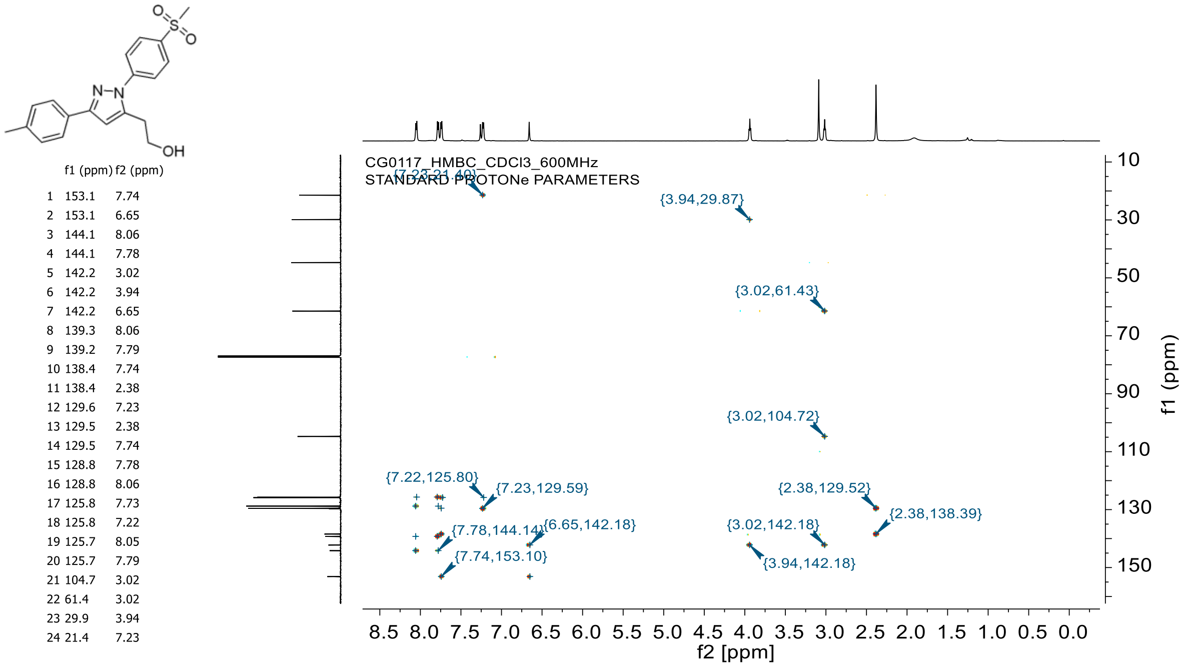

	f1 (ppm)	f2 (ppm)
1	153.1	7.74
2	153.1	6.65
3	144.1	8.06
4	144.1	7.78
5	142.2	3.02
6	142.2	3.94
7	142.2	6.65
8	139.3	8.06
9	139.2	7.79
10	138.4	7.74
11	138.4	2.38
12	129.6	7.23
13	129.5	2.38
14	129.5	7.74
15	128.8	7.78
16	128.8	8.06
17	125.8	7.73
18	125.8	7.22
19	125.7	8.05
20	125.7	7.79
21	104.7	3.02
22	61.4	3.02
23	29.9	3.94
24	21.4	7.23

Figure S69. HMBC spectrum of compound **3d** in $CDCl_3$

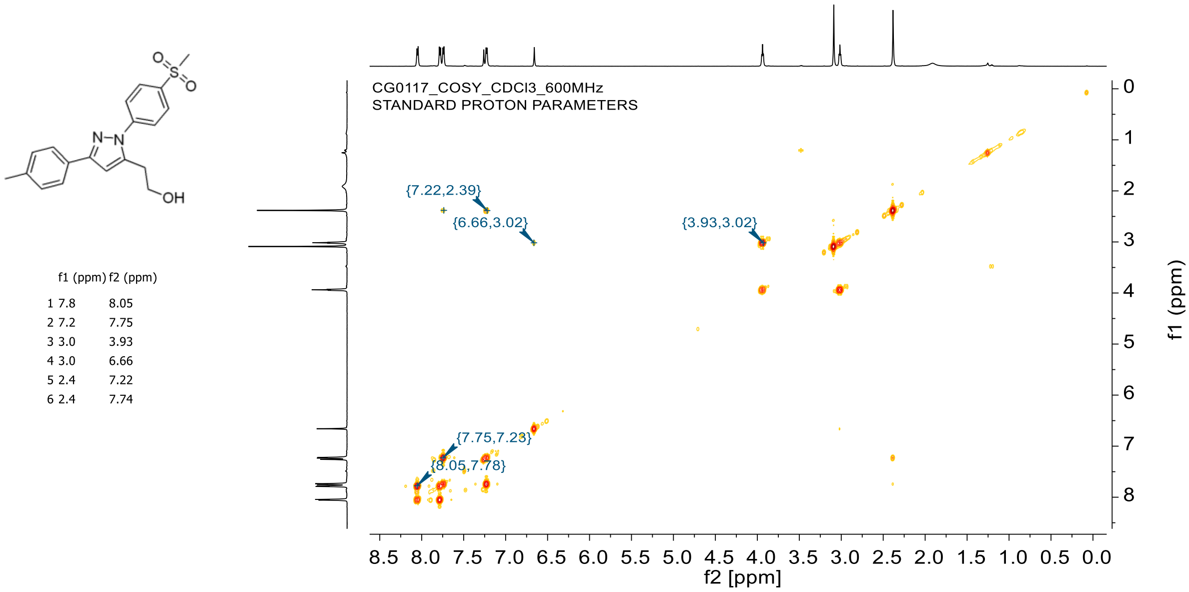

	f1 (ppm)	f2 (ppm)
1	7.8	8.05
2	7.2	7.75
3	3.0	3.93
4	3.0	6.66
5	2.4	7.22
6	2.4	7.74

Figure S70. COSY spectrum of compound **3d** in $CDCl_3$

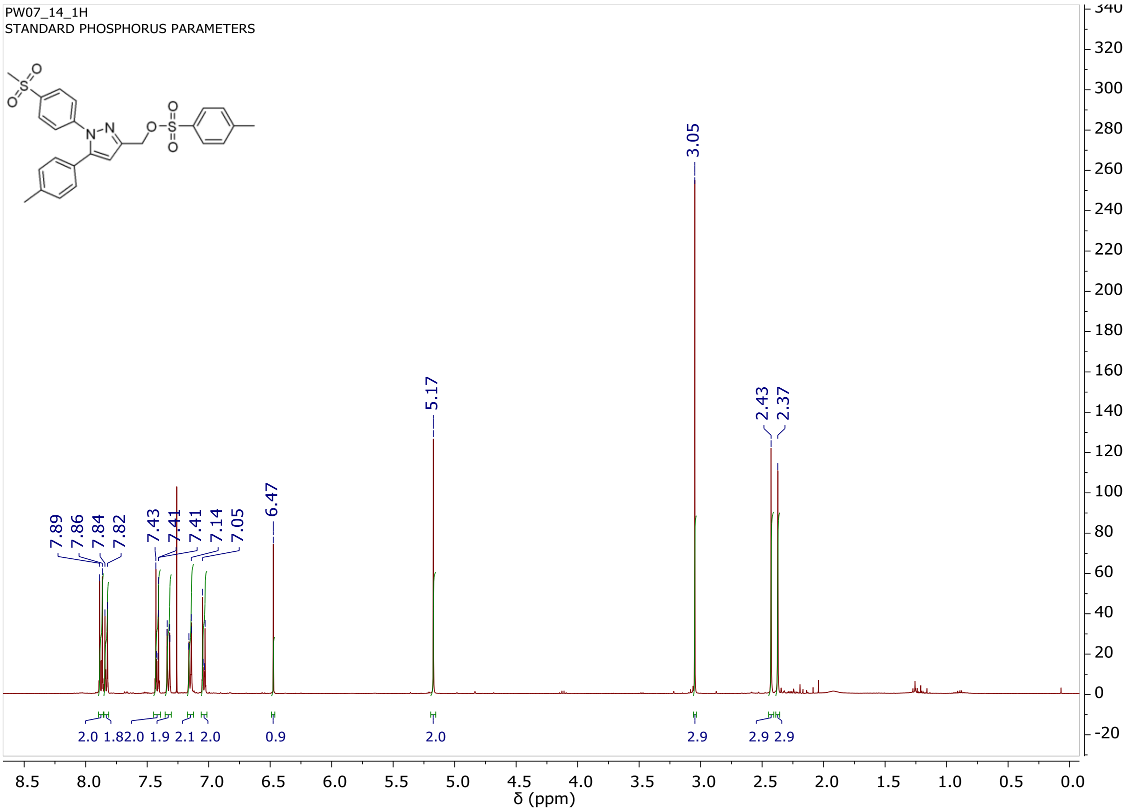

Figure S71. ^{1}H NMR spectrum of compound **4a** in $CDCl_3$

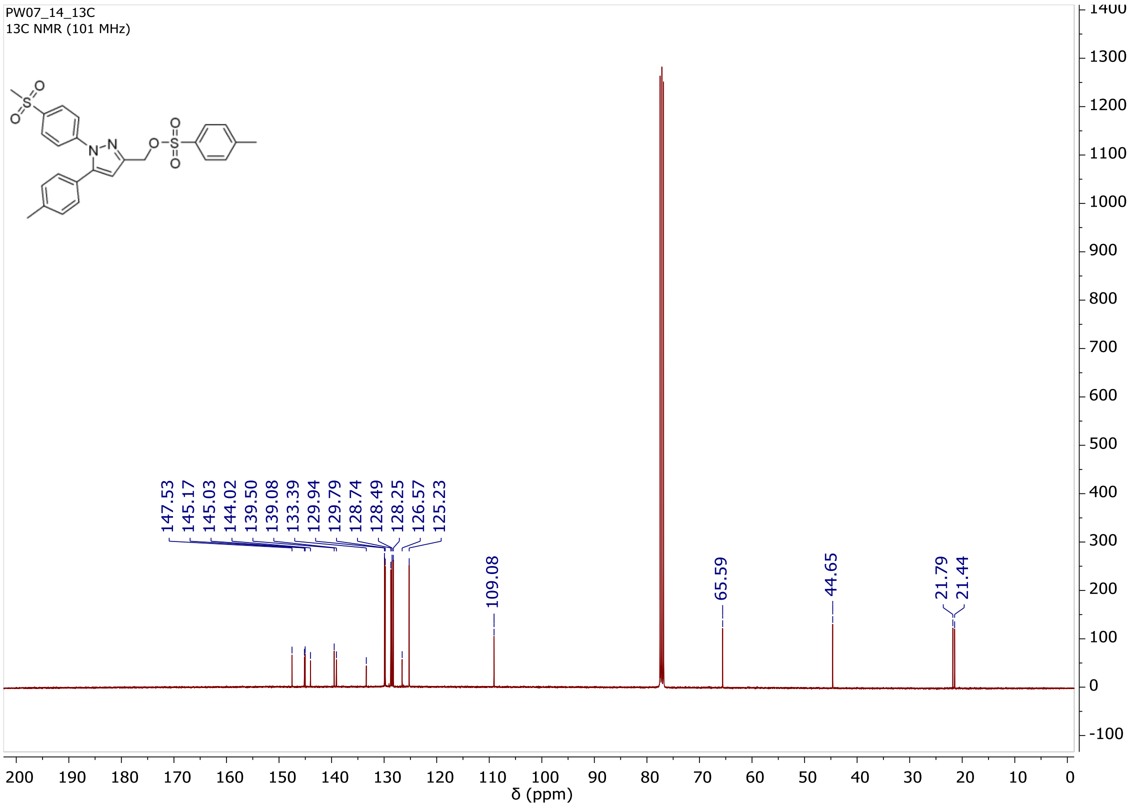

Figure S72. ^{13}C NMR spectrum of compound **4a** in $CDCl_3$

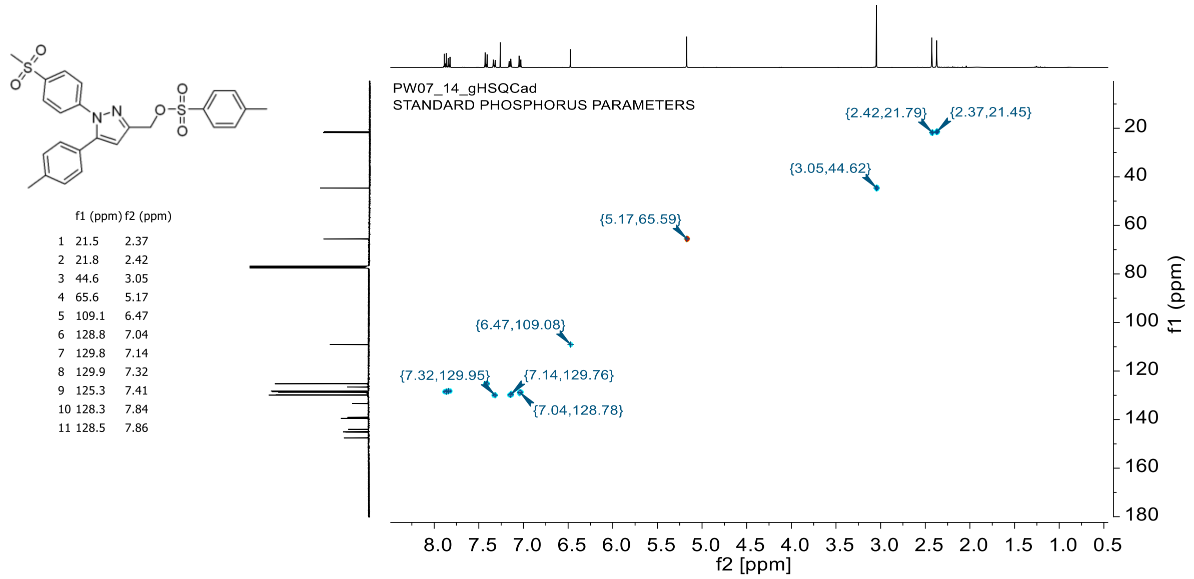

Figure S73. HSQC spectrum of compound **4a** in $CDCl_3$

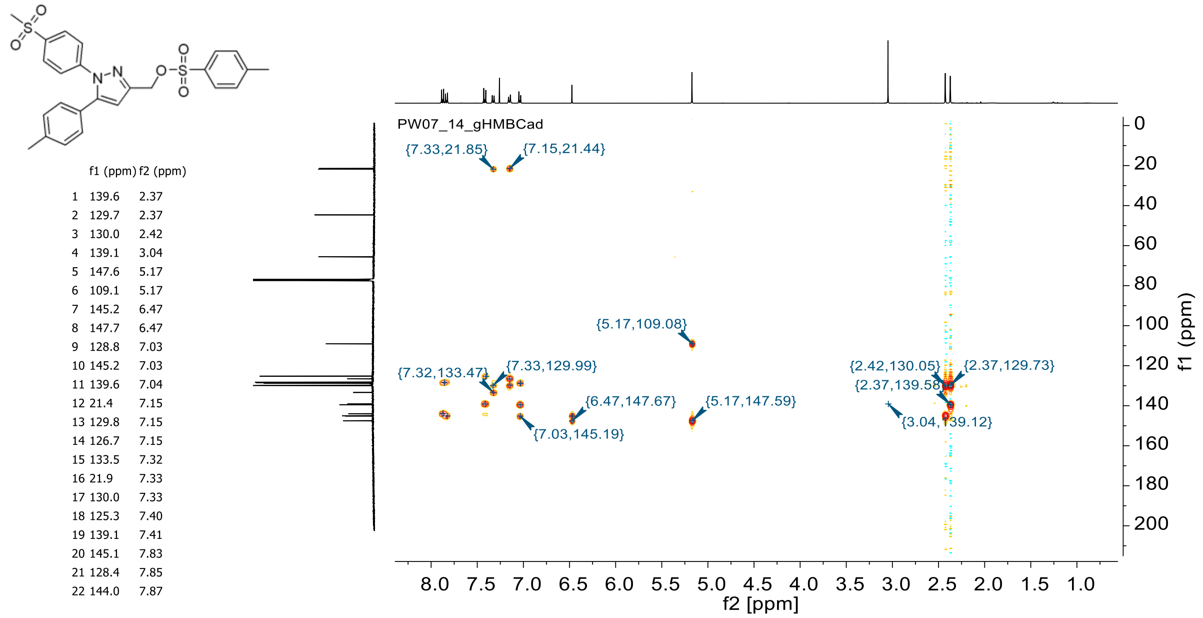

Figure S74. HMBC spectrum of compound **4a** in $CDCl_3$

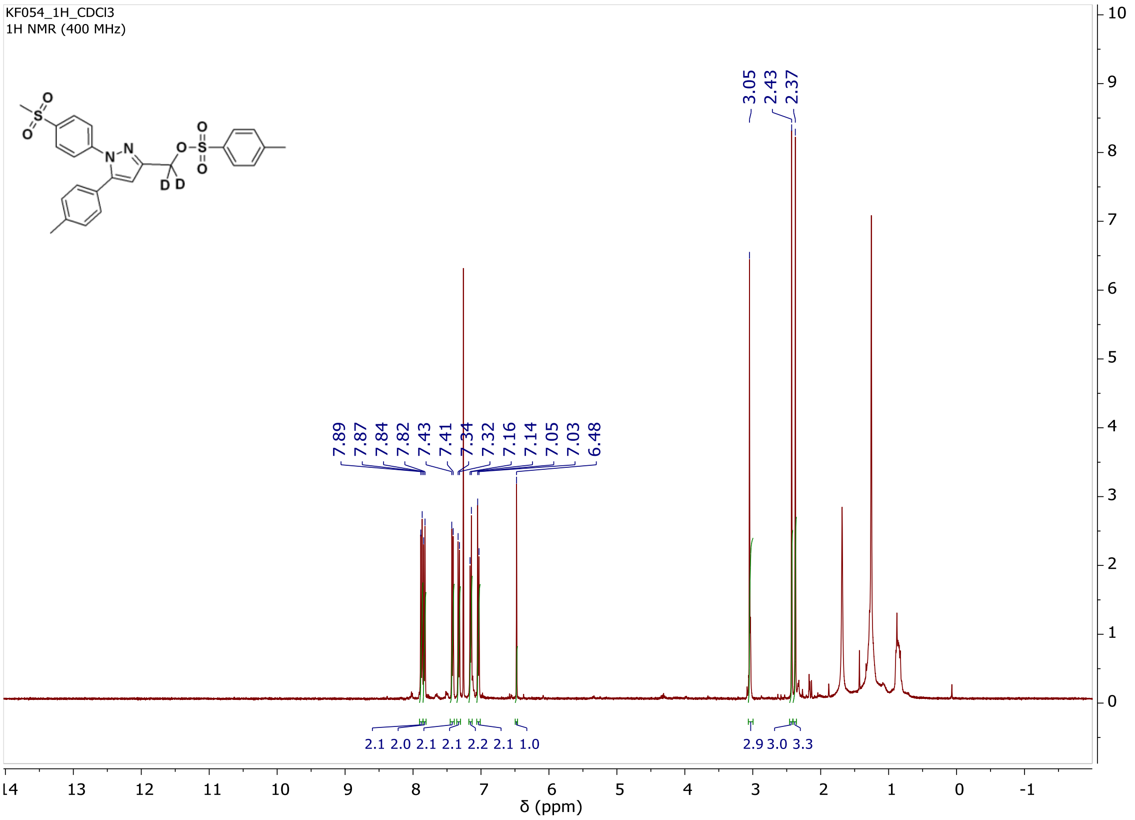

Figure S75. ^{1}H NMR spectrum of compound [D$_2$]**4a** in $CDCl_3$

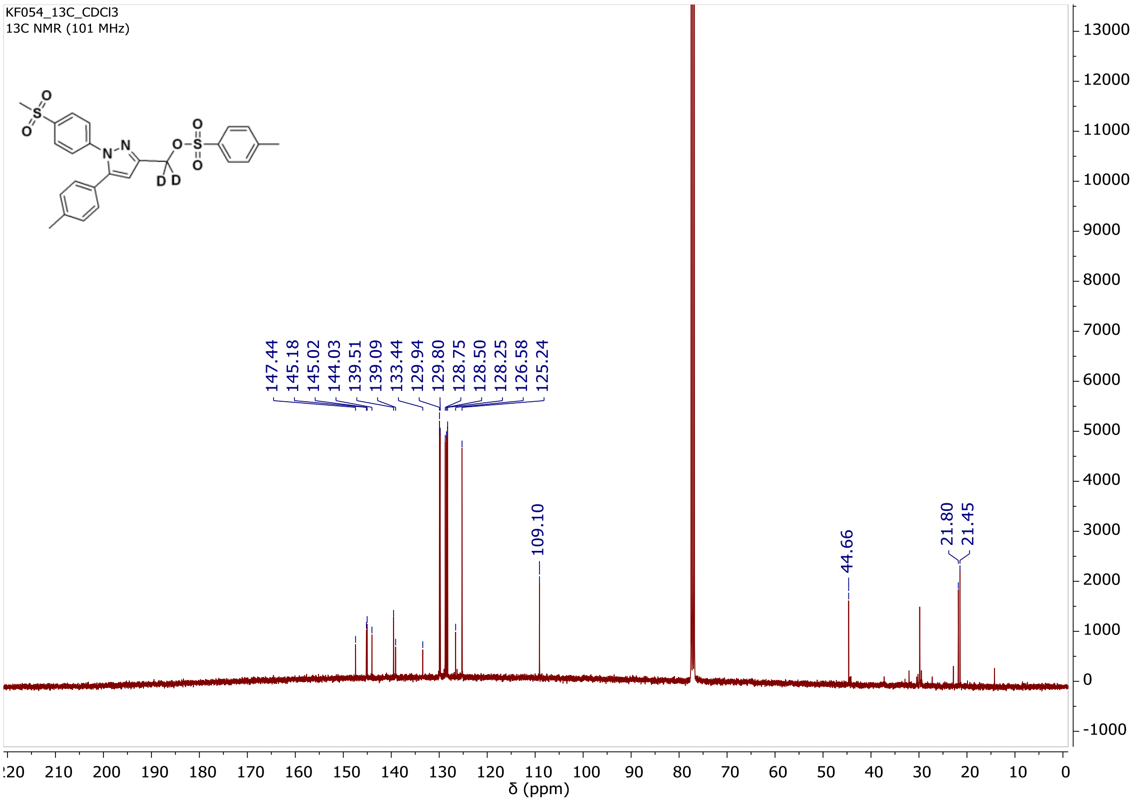

Figure S76. ^{13}C NMR spectrum of compound [D$_2$]**4a** in $CDCl_3$

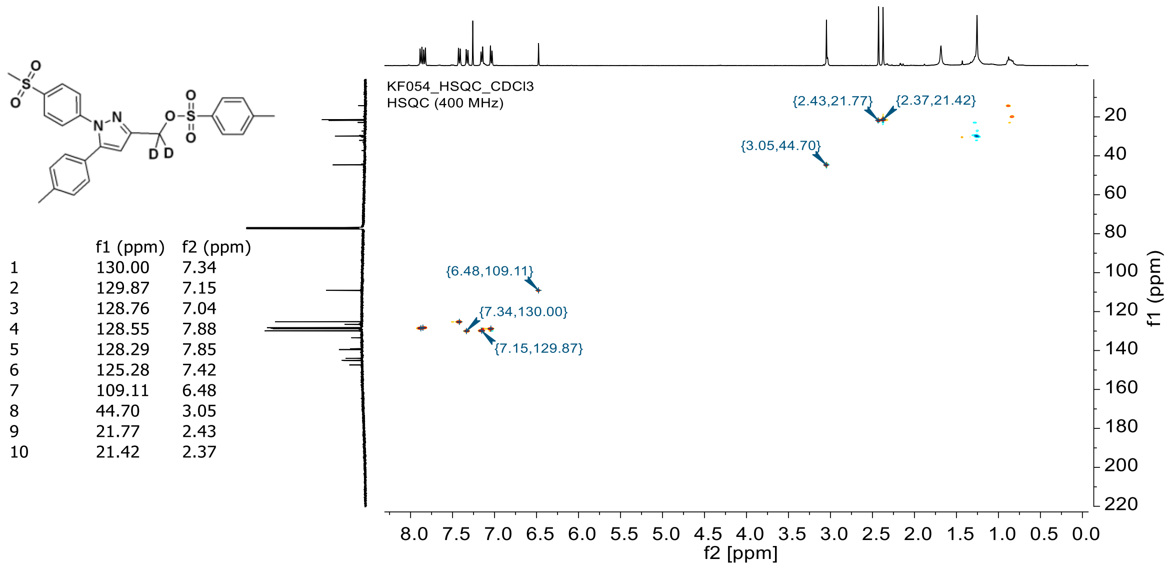

	f1 (ppm)	f2 (ppm)
1	130.00	7.34
2	129.87	7.15
3	128.76	7.04
4	128.55	7.88
5	128.29	7.85
6	125.28	7.42
7	109.11	6.48
8	44.70	3.05
9	21.77	2.43
10	21.42	2.37

Figure S77. HSQC spectrum of compound [D_2]**4a** in $CDCl_3$

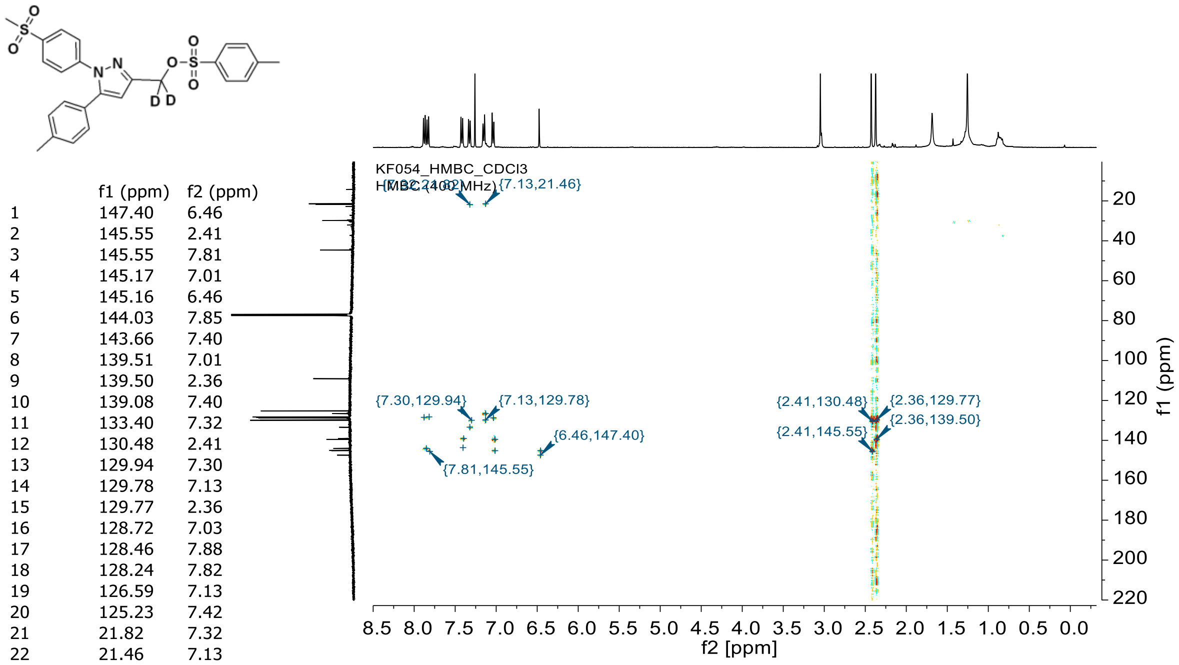

	f1 (ppm)	f2 (ppm)
1	147.40	6.46
2	145.55	2.41
3	145.55	7.81
4	145.17	7.01
5	145.16	6.46
6	144.03	7.85
7	143.66	7.40
8	139.51	7.01
9	139.50	2.36
10	139.08	7.40
11	133.40	7.32
12	130.48	2.41
13	129.94	7.30
14	129.78	7.13
15	129.77	2.36
16	128.72	7.03
17	128.46	7.88
18	128.24	7.82
19	126.59	7.13
20	125.23	7.42
21	21.82	7.32
22	21.46	7.13

Figure S78. HMBC spectrum of compound [D_2]**4a** in $CDCl_3$

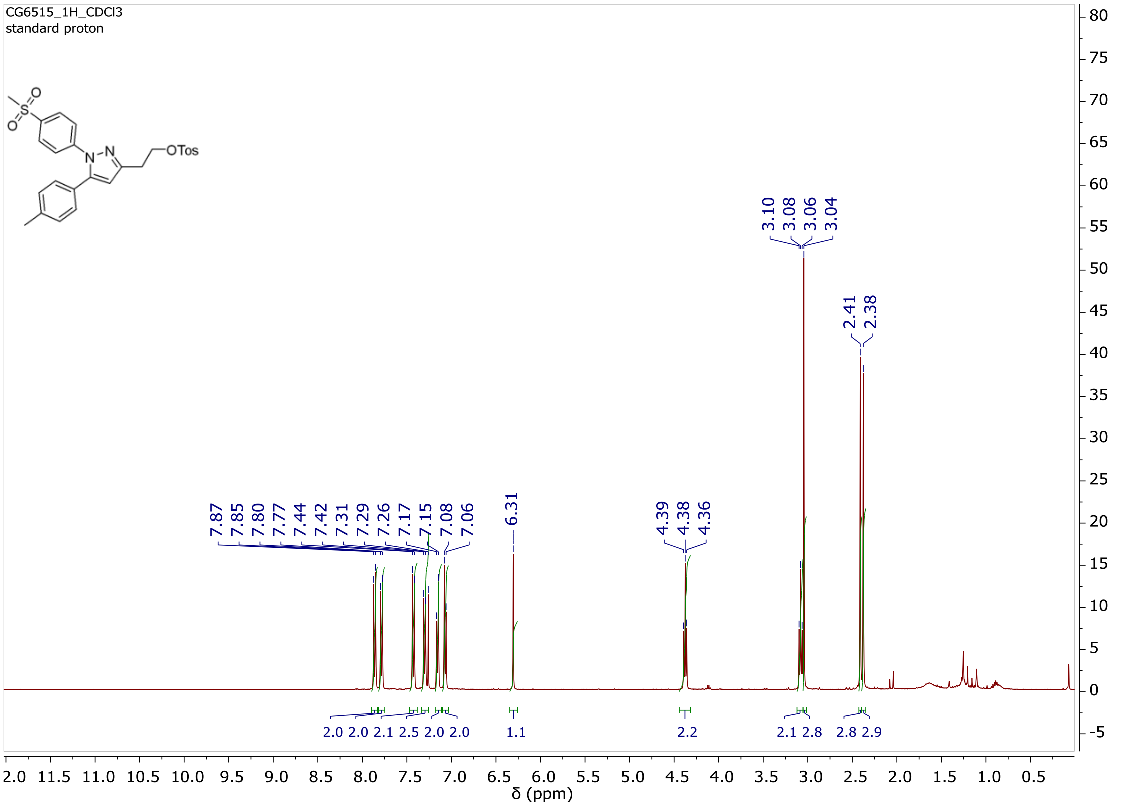

Figure S79. ^{1}H NMR spectrum of compound **4b** in $CDCl_3$

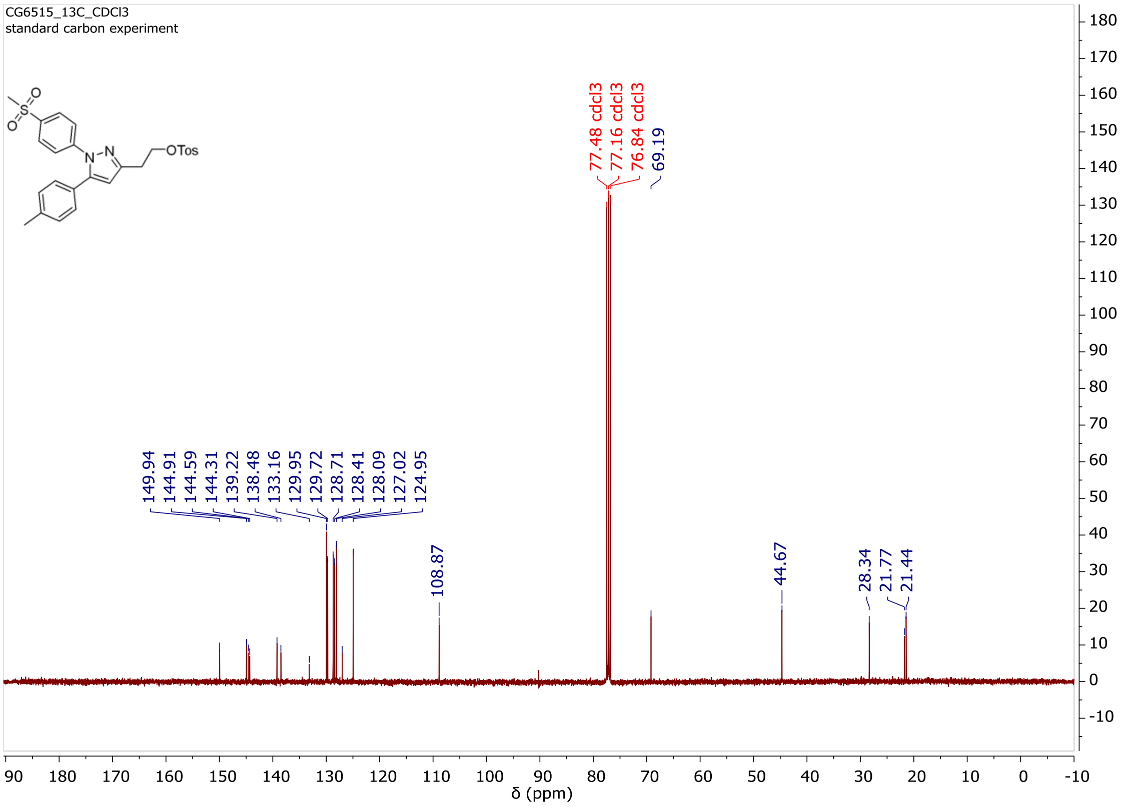

Figure S80. ^{13}C NMR spectrum of compound **4b** in $CDCl_3$

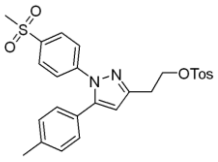

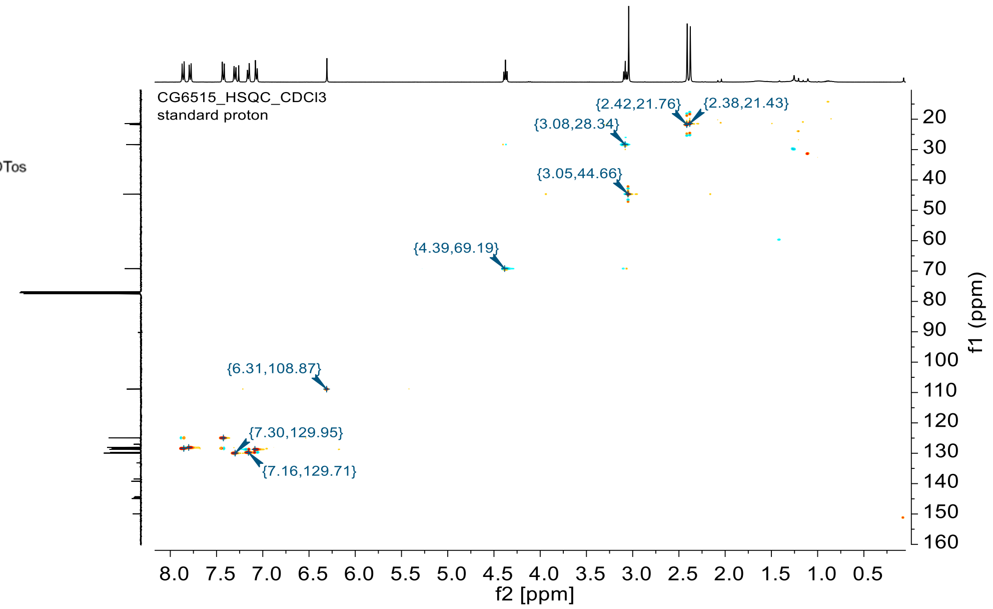

	f1 (ppm)	f2 (ppm)
1	130.0	7.30
2	129.7	7.16
3	128.7	7.09
4	128.4	7.85
5	128.1	7.80
6	125.0	7.43
7	108.9	6.31
8	69.2	4.39
9	44.7	3.05
10	28.3	3.08
11	21.8	2.42
12	21.4	2.38

Figure S81. HSQC spectrum of compound **4b** in $CDCl_3$

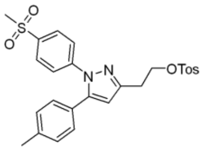

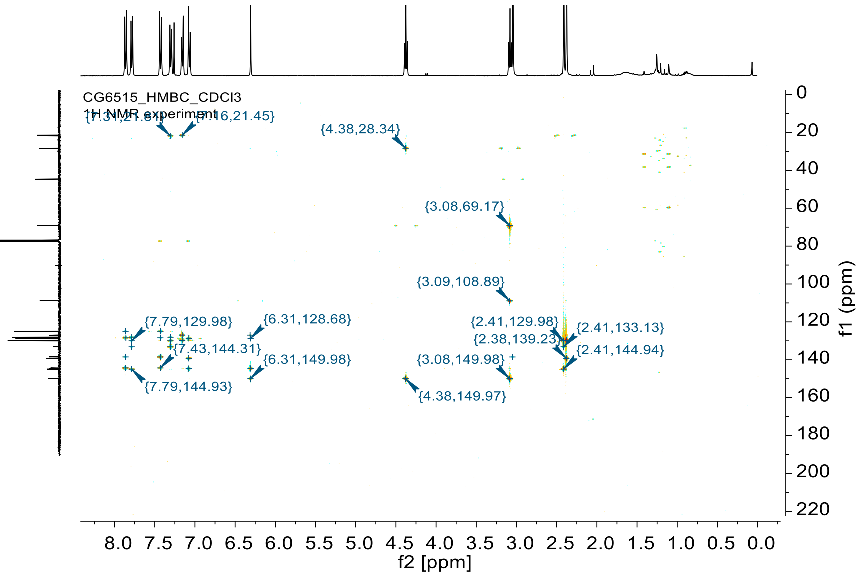

	f1 (ppm)	f2 (ppm)		f1 (ppm)	f2 (ppm)
1	150.0	6.31	20	130.0	7.31
2	150.0	3.08	21	129.8	7.16
3	150.0	4.38	22	129.8	2.38
4	144.9	2.41	23	128.7	7.08
5	144.9	7.79	24	128.7	6.31
6	144.6	7.08	25	128.4	7.86
7	144.6	6.31	26	128.4	7.43
8	144.3	7.43	27	128.1	7.30
9	144.3	7.86	28	128.1	7.79
10	139.2	2.38	29	127.0	6.31
11	139.2	7.08	30	127.0	7.16
12	138.5	3.05	31	125.0	7.86
13	138.5	7.43	32	124.9	7.43
14	138.5	7.86	33	108.9	3.09
15	133.2	7.31	34	69.2	3.08
16	133.2	7.79	35	28.3	4.38
17	133.1	2.41	36	21.8	7.31
18	130.0	7.79	37	21.4	7.16
19	130.0	2.41			

Figure S82. HMBC spectrum of compound **4b** in $CDCl_3$

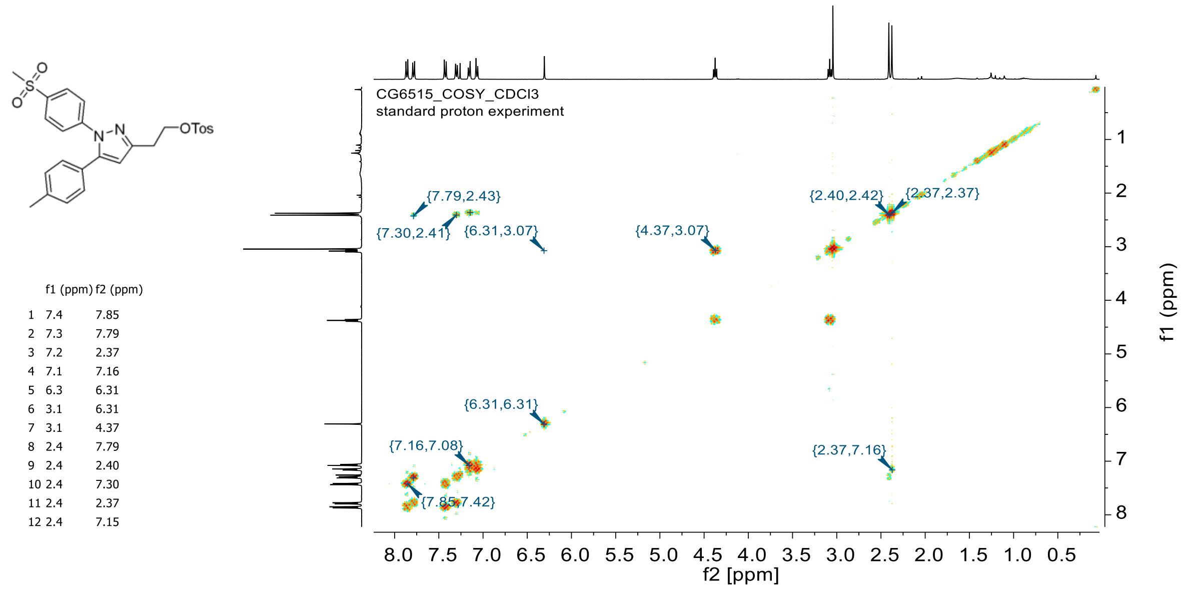

Figure S83. COSY spectrum of compound **4b** in $CDCl_3$

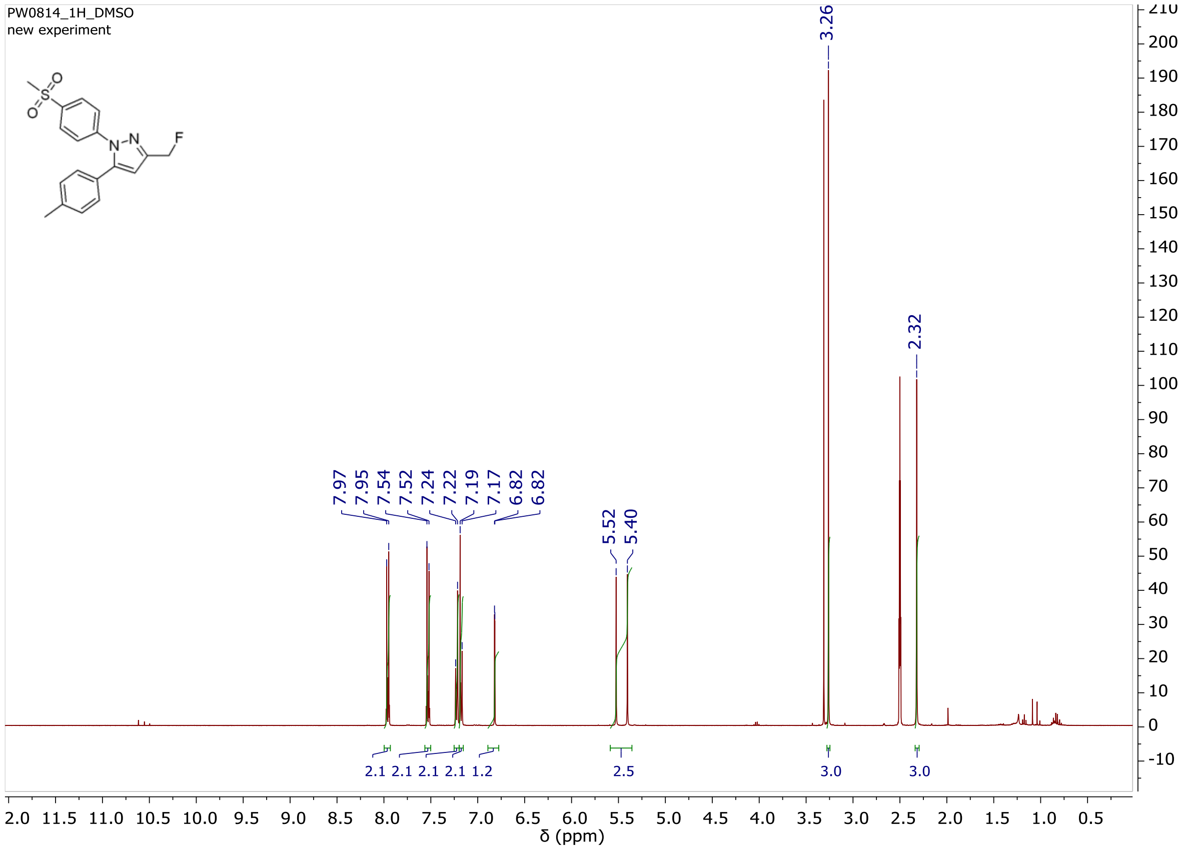

Figure S84. ^{1}H NMR spectrum of compound **5a** in DMSO-d_6

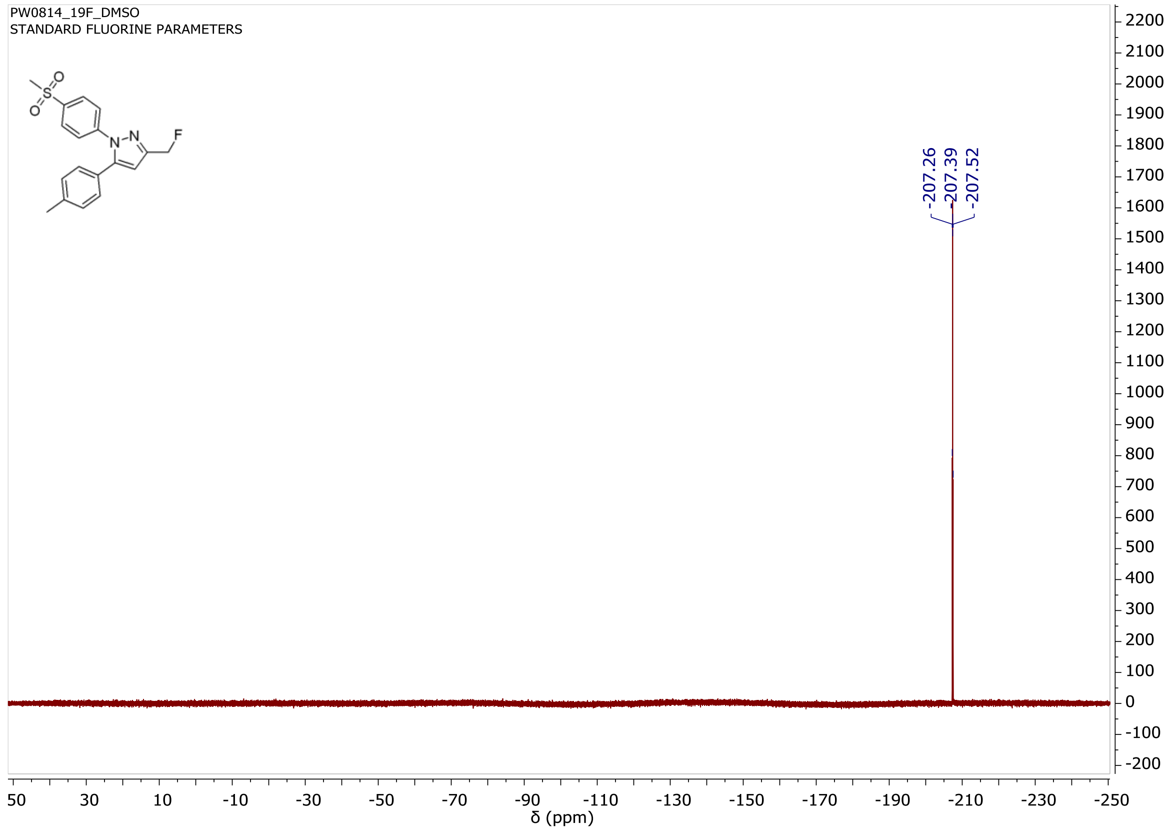

Figure S85. ^{19}F NMR spectrum of compound **5a** in DMSO-d_6

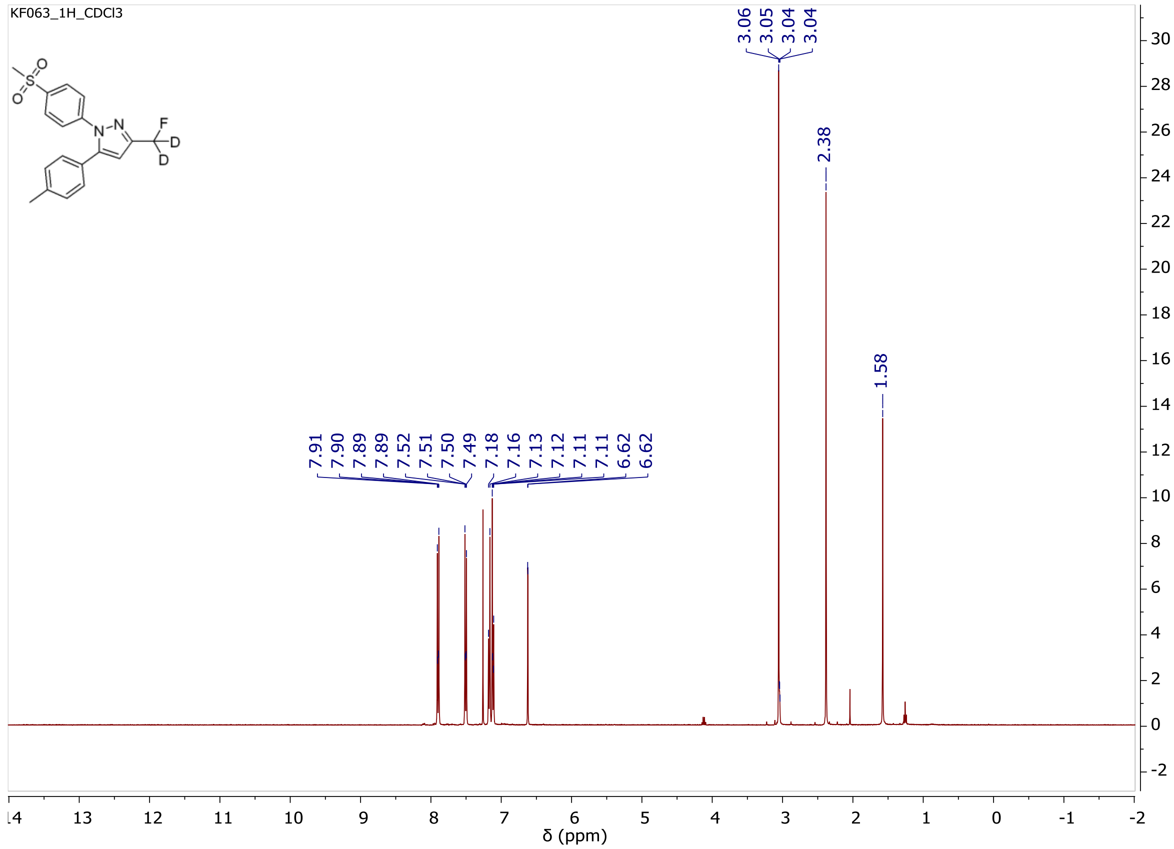

Figure S86. ^{1}H NMR spectrum of compound **[D$_2$]5a** in $CDCl_3$

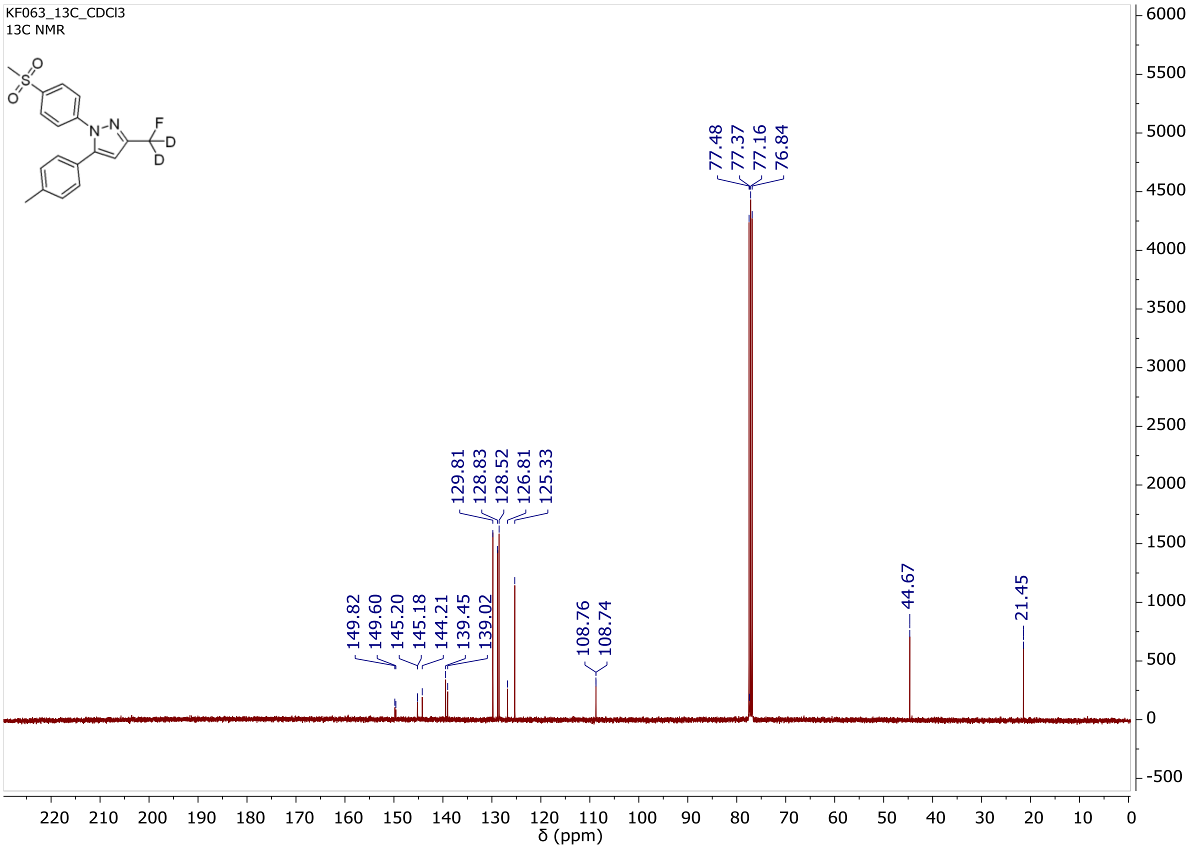

Figure S87. ^{13}C NMR spectrum of compound **[D$_2$]5a** in $CDCl_3$

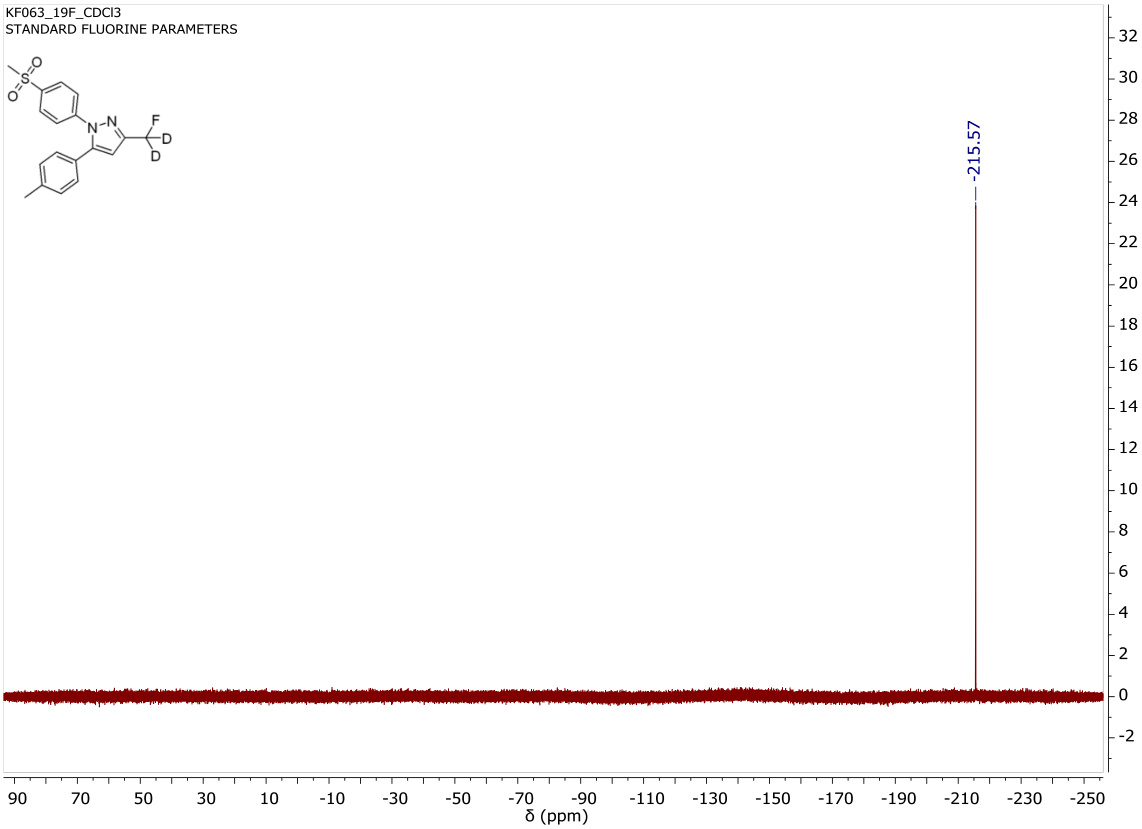

Figure S88. ^{19}F NMR spectrum of compound **[D$_2$]5a** in $CDCl_3$

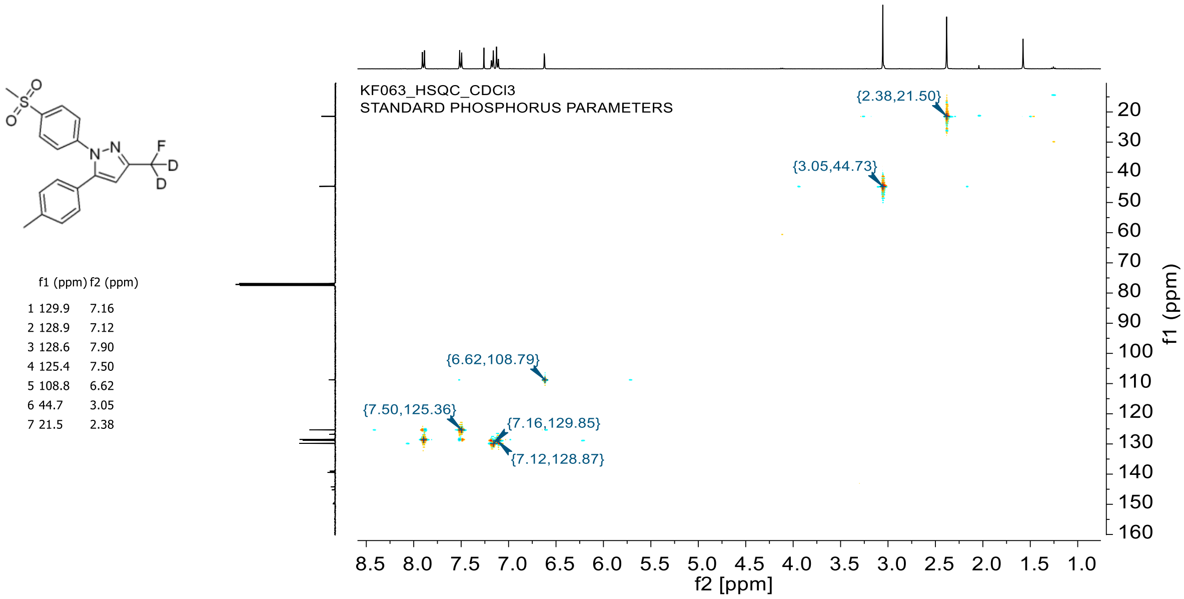

Figure S89. HSQC spectrum of compound **[D$_2$]5a** in $CDCl_3$

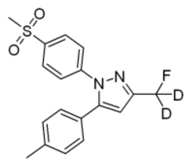

	f1 (ppm)	f2 (ppm)
1	149.7	6.64
2	145.3	2.39
3	145.2	7.11
4	145.2	6.64
5	144.3	7.92
6	144.3	7.52
7	139.5	7.11
8	139.5	2.39
9	139.1	3.07
10	139.1	7.52
11	139.0	7.88
12	129.9	2.39
13	129.9	7.17
14	128.9	7.13
15	128.6	7.90
16	128.6	7.52
17	126.8	7.17
18	126.8	6.62
19	126.8	2.39
20	125.4	7.90
21	125.4	7.52
22	21.5	7.17

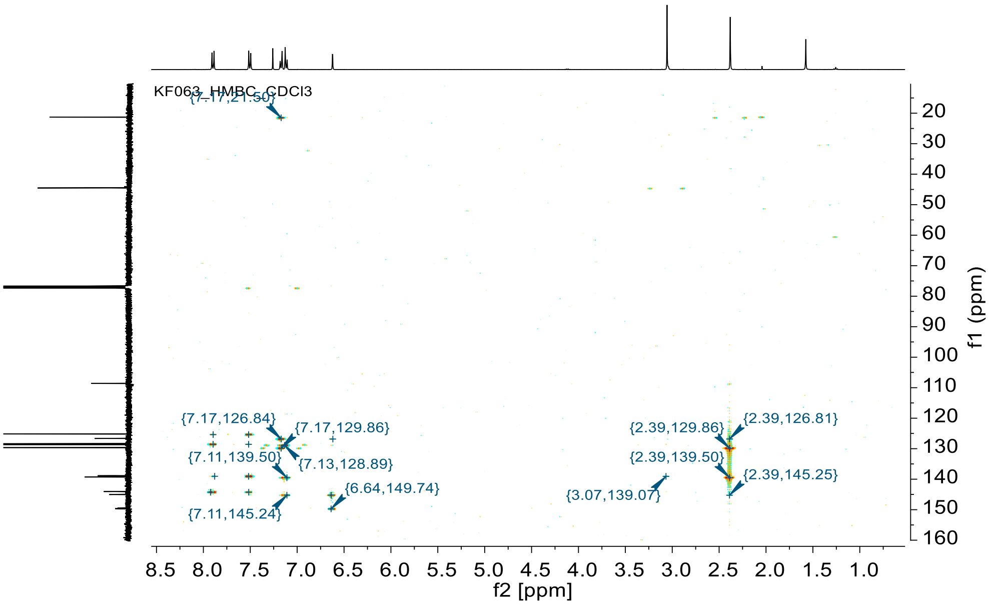

Figure S90. HMBC spectrum of compound **[D$_2$]5a** in $CDCl_3$

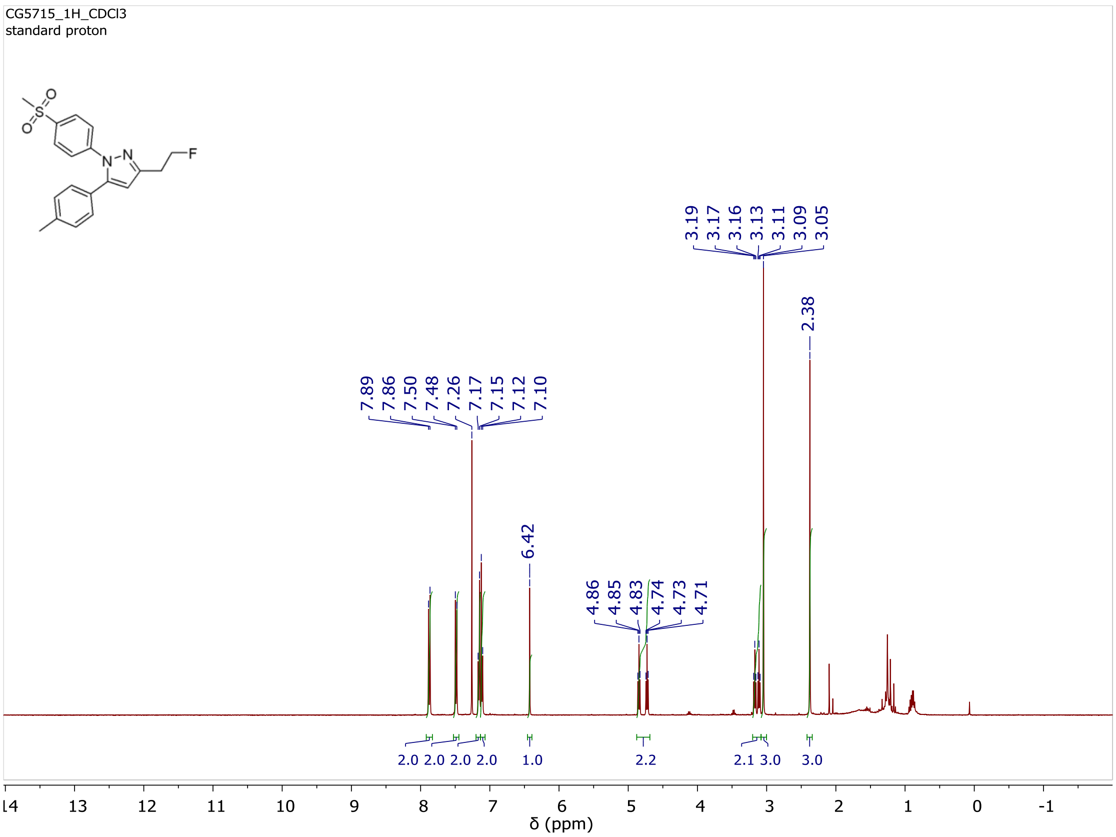

Figure S91. ^{1}H NMR spectrum of compound **5b** in $CDCl_3$

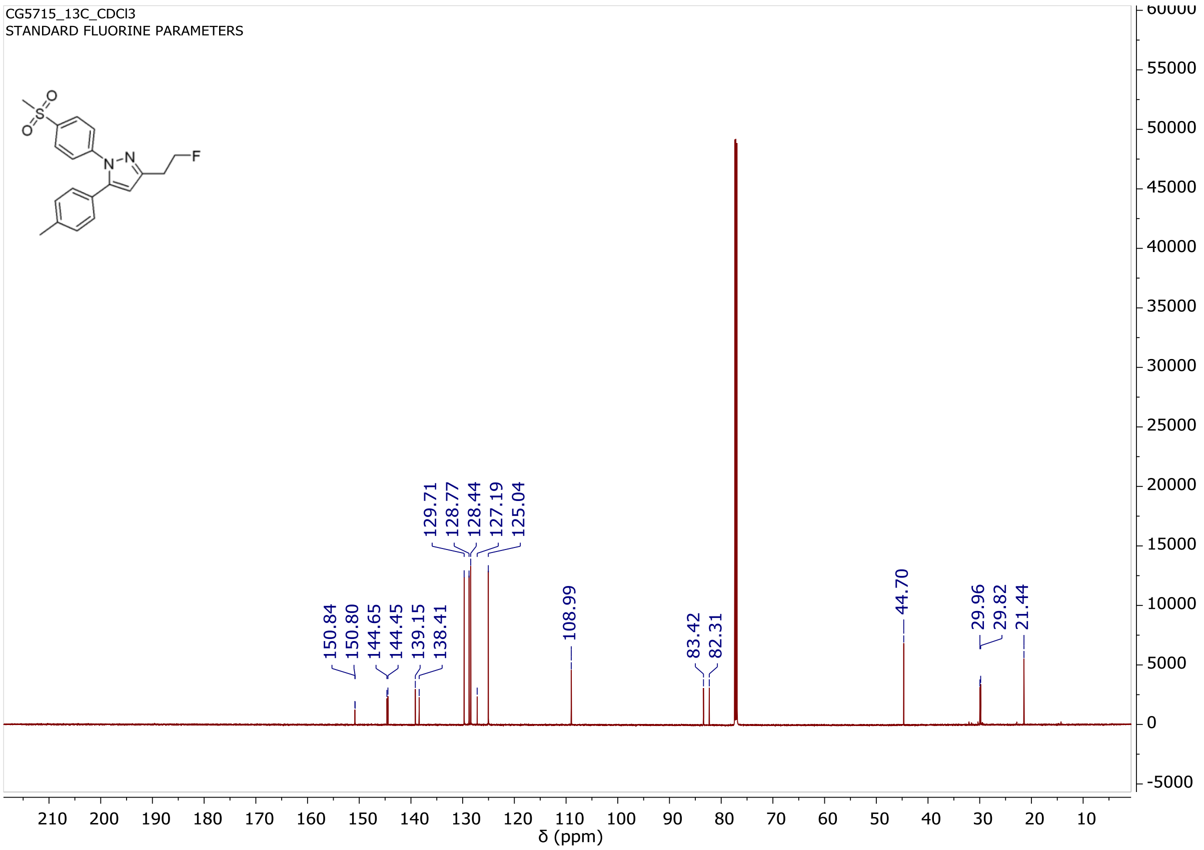

Figure S92. ^{13}C NMR spectrum of compound **5b** in $CDCl_3$

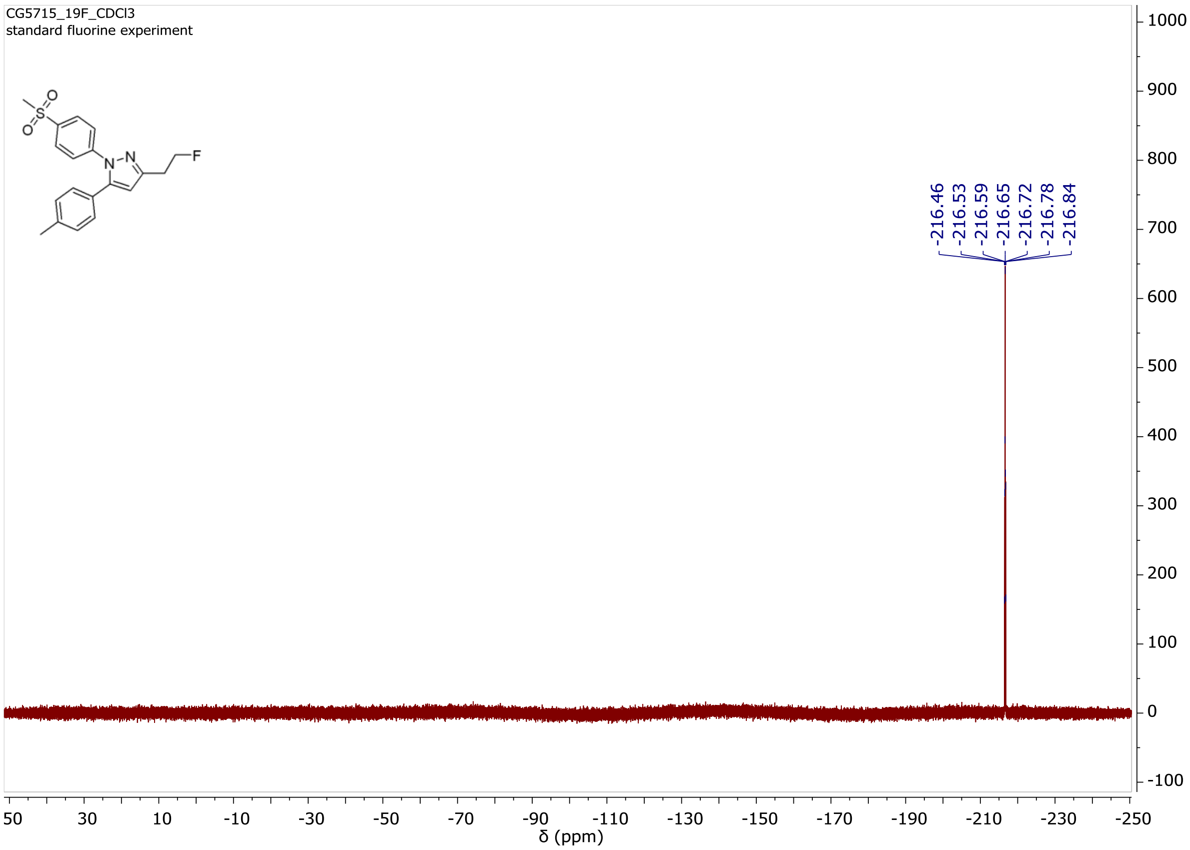

Figure S93. ^{19}F NMR spectrum of compound **5b** in $CDCl_3$

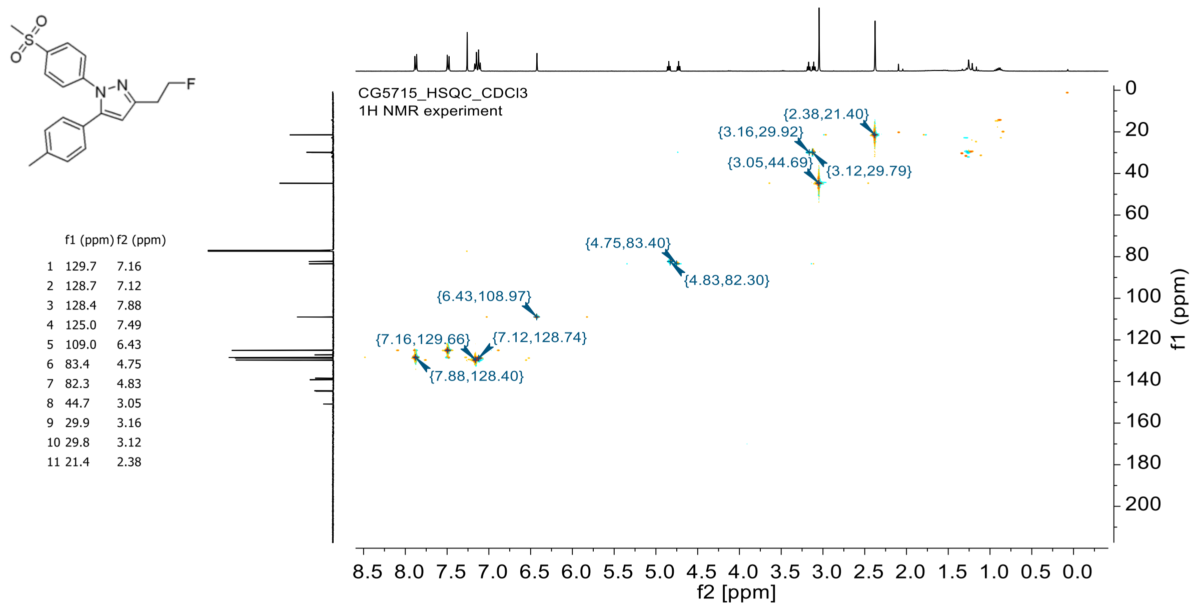

Figure S94. HSQC spectrum of compound **5b** in $CDCl_3$

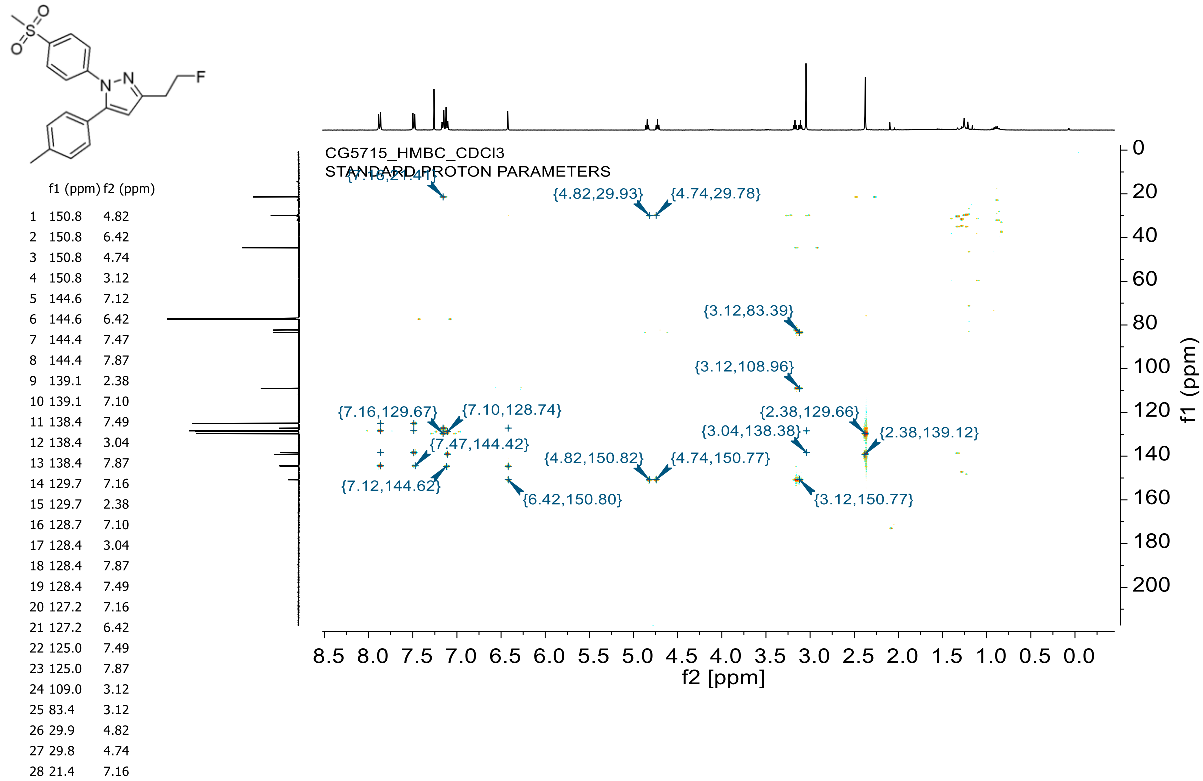

Figure S95. HMBC spectrum of compound **5b** in $CDCl_3$

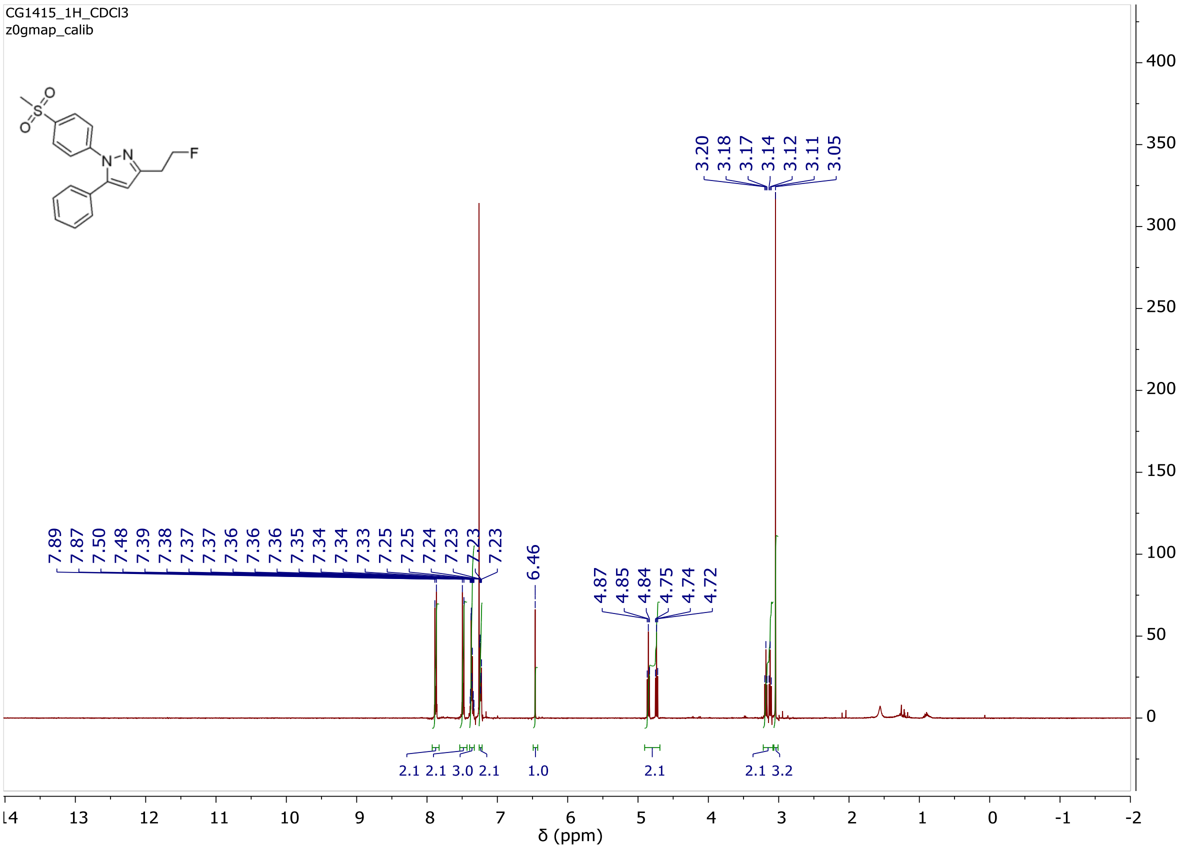

Figure S96. ^{1}H NMR spectrum of compound **5c** in $CDCl_3$

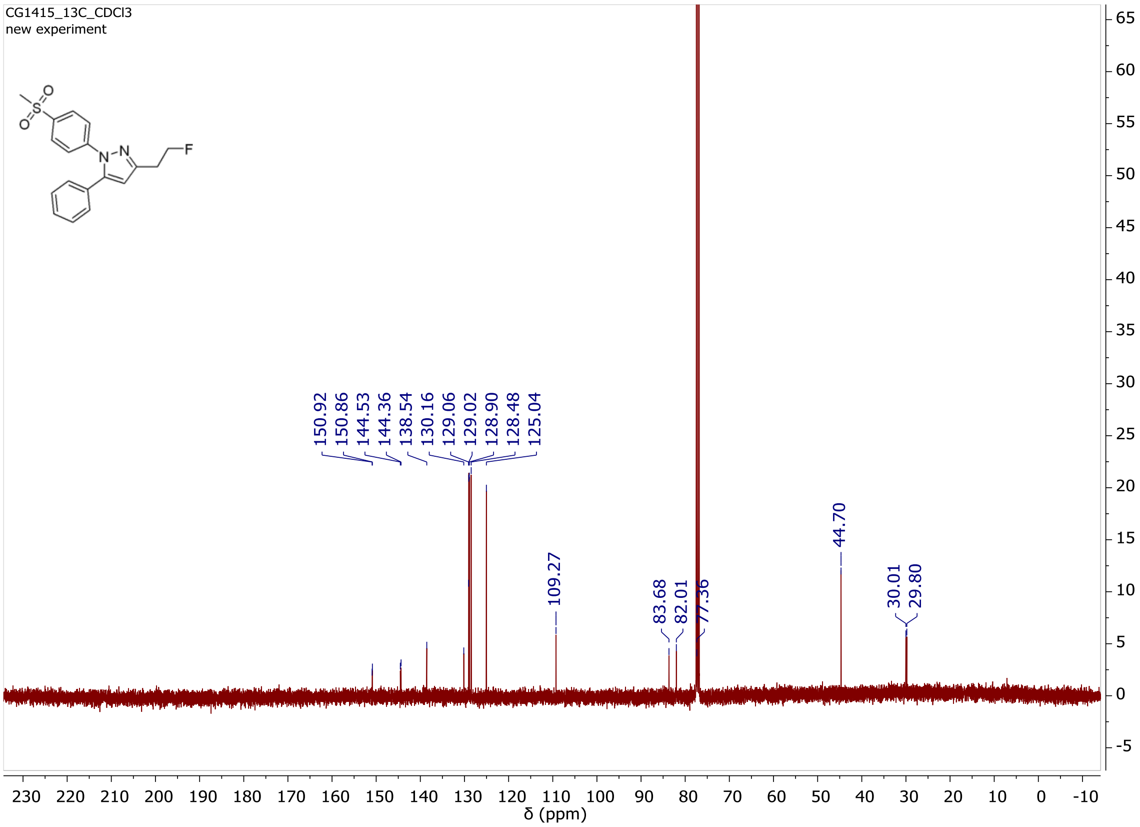

Figure S97. ^{13}C NMR spectrum of compound **5c** in $CDCl_3$

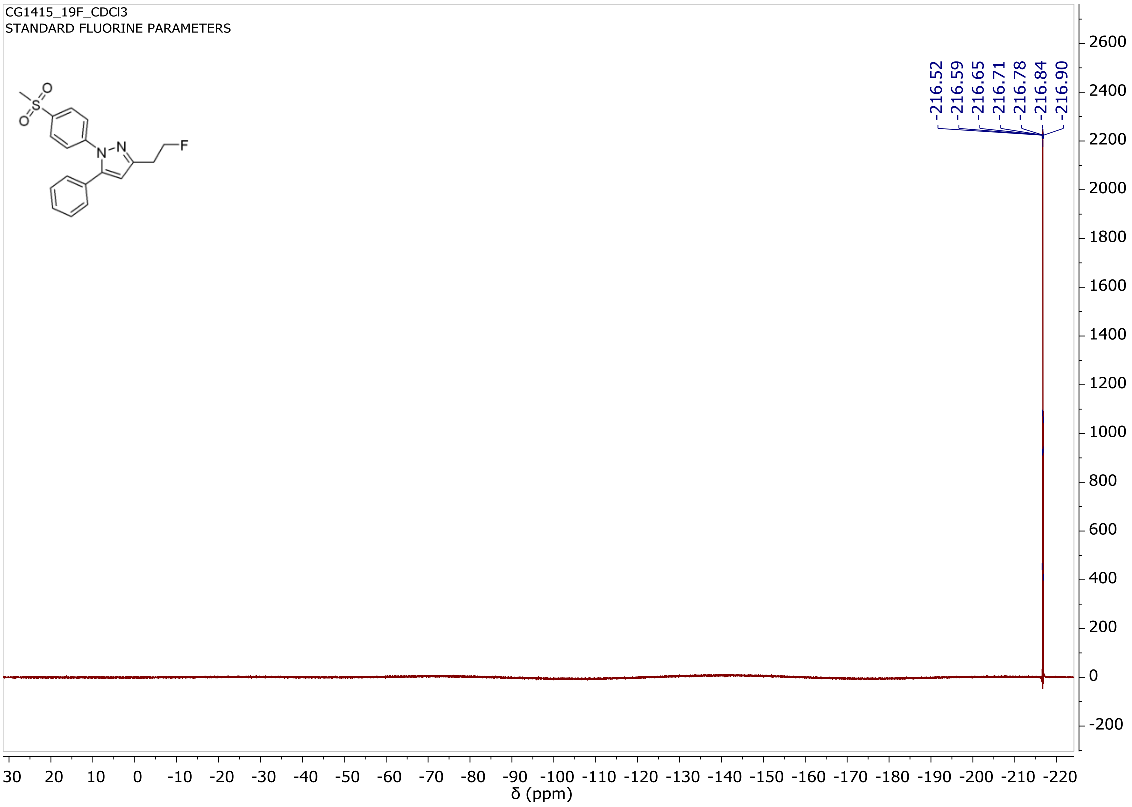

Figure S98. ^{19}F NMR spectrum of compound **5c** in $CDCl_3$

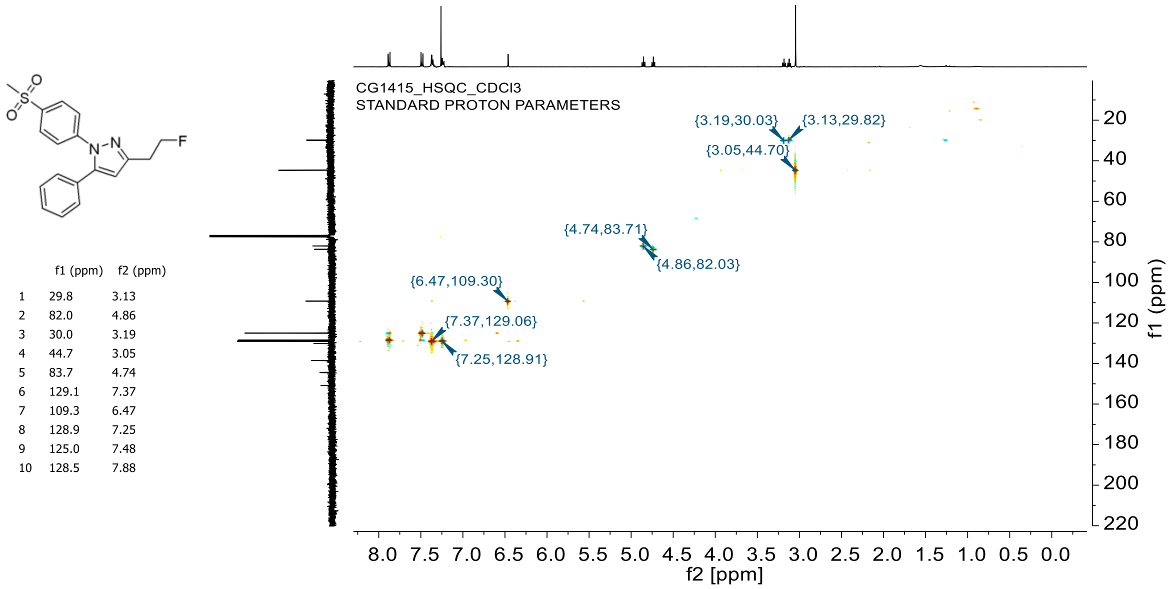

Figure S99. HSQC spectrum of compound **5c** in $CDCl_3$

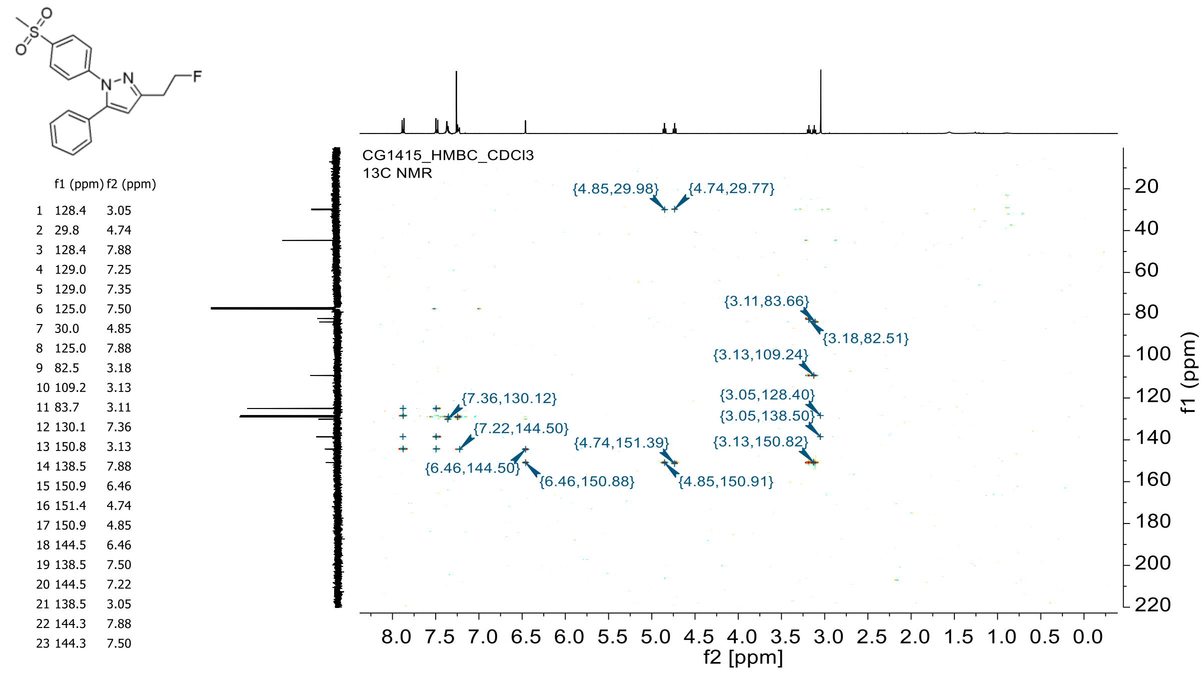

Figure S100. HMBC spectrum of compound **5c** in $CDCl_3$

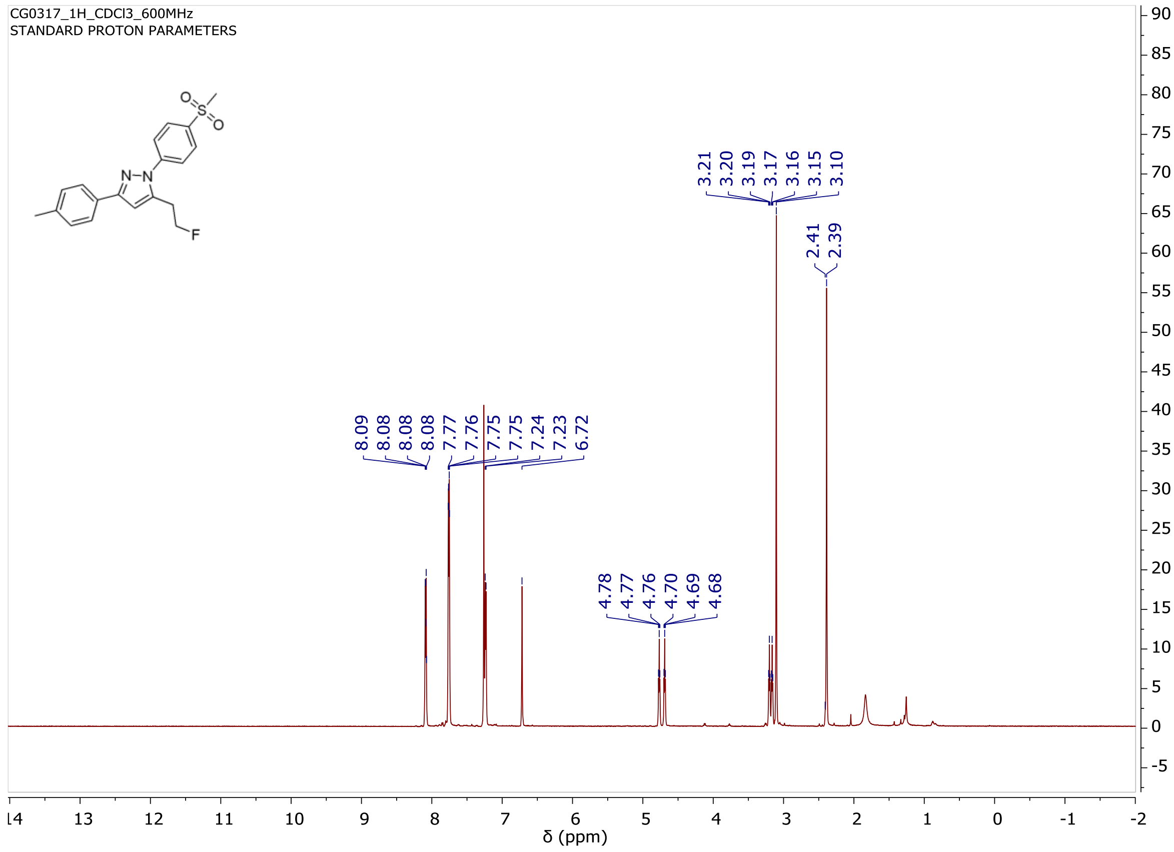

Figure S101. ^{1}H NMR spectrum of compound **5d** in $CDCl_3$

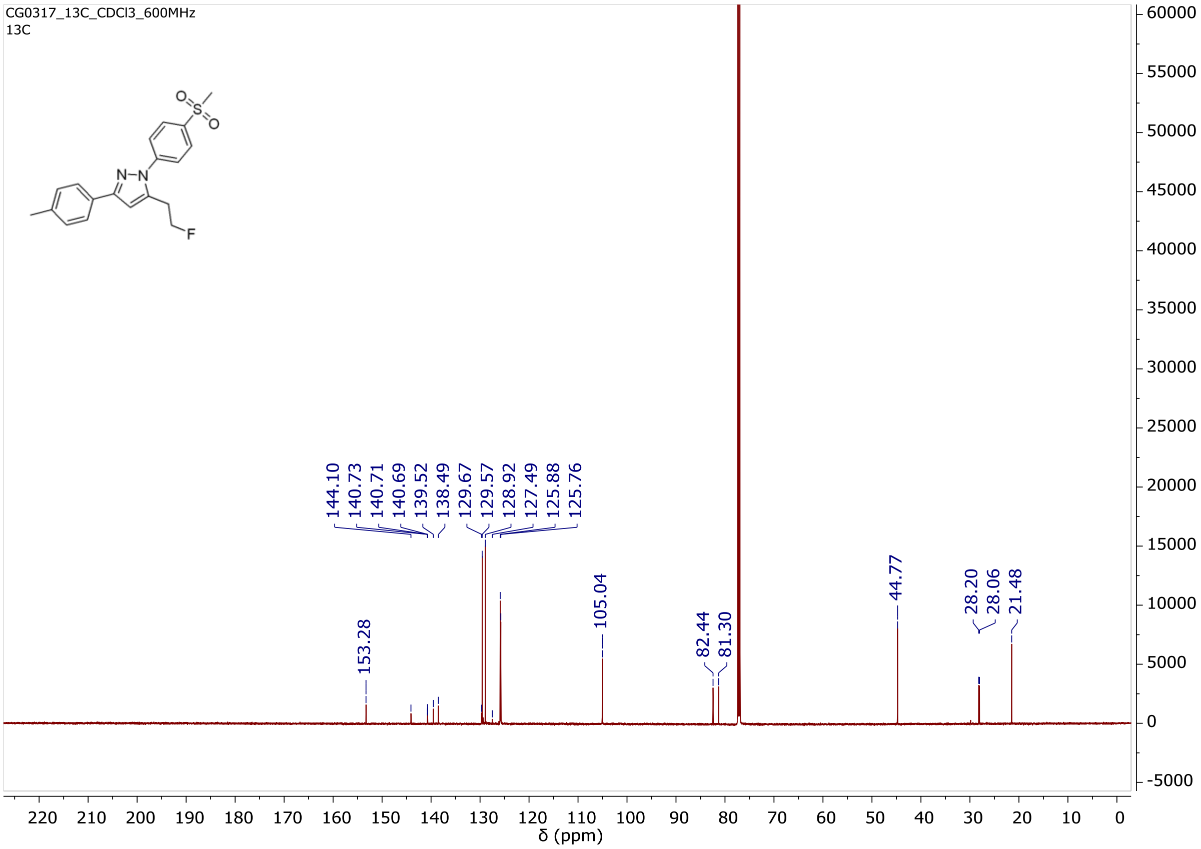

Figure S102. ^{13}C NMR spectrum of compound **5d** in $CDCl_3$

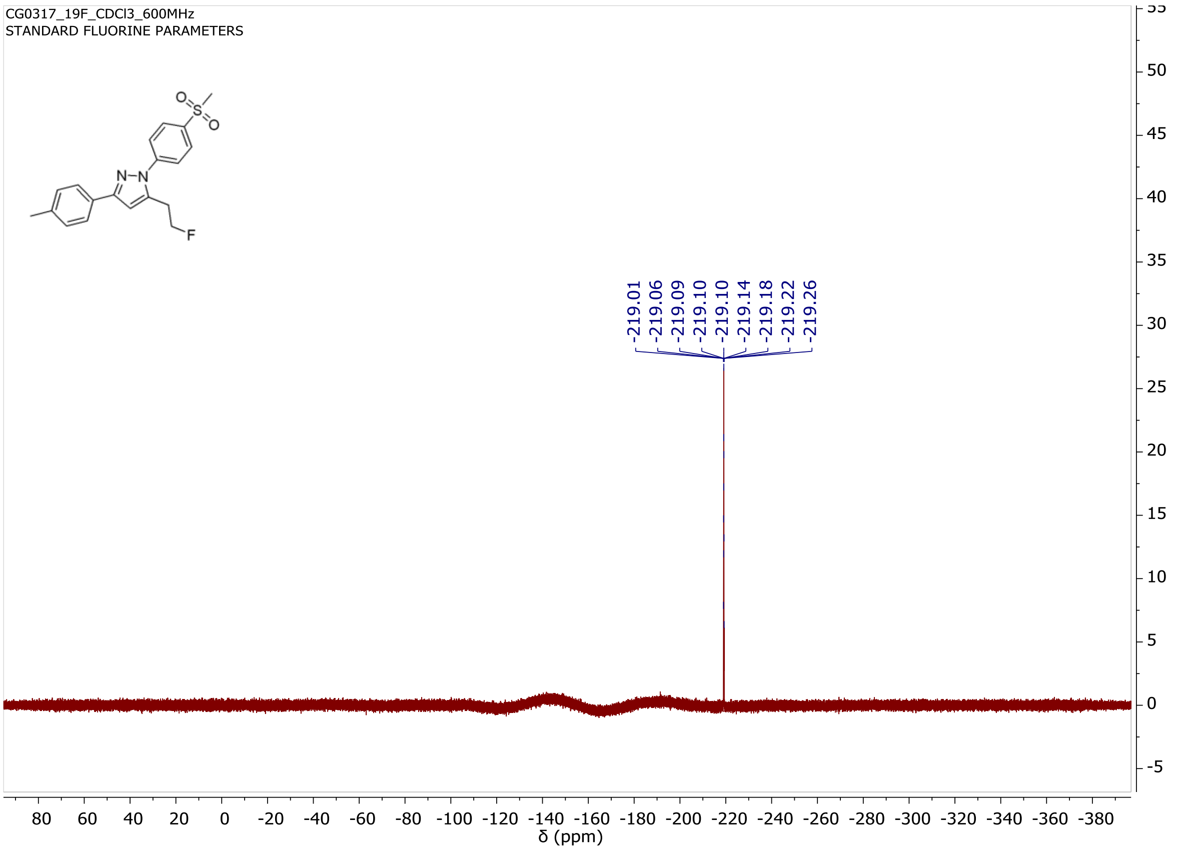

Figure S103. ^{19}F NMR spectrum of compound **5d** in $CDCl_3$

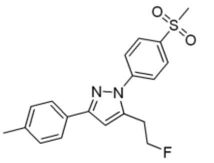

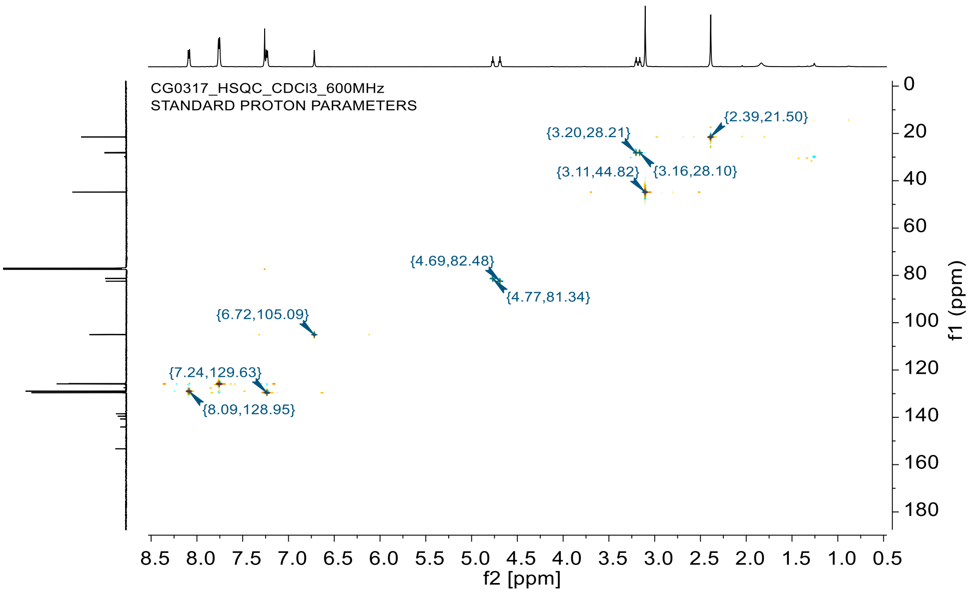

	f1 (ppm)	f2 (ppm)
1	21.5	2.39
2	44.8	3.11
3	28.1	3.16
4	28.2	3.20
5	82.5	4.69
6	81.3	4.77
7	105.1	6.72
8	129.6	7.24
9	125.9	7.76
10	129.0	8.09

Figure S104. HSQC spectrum of compound **5d** in $CDCl_3$

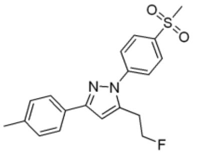

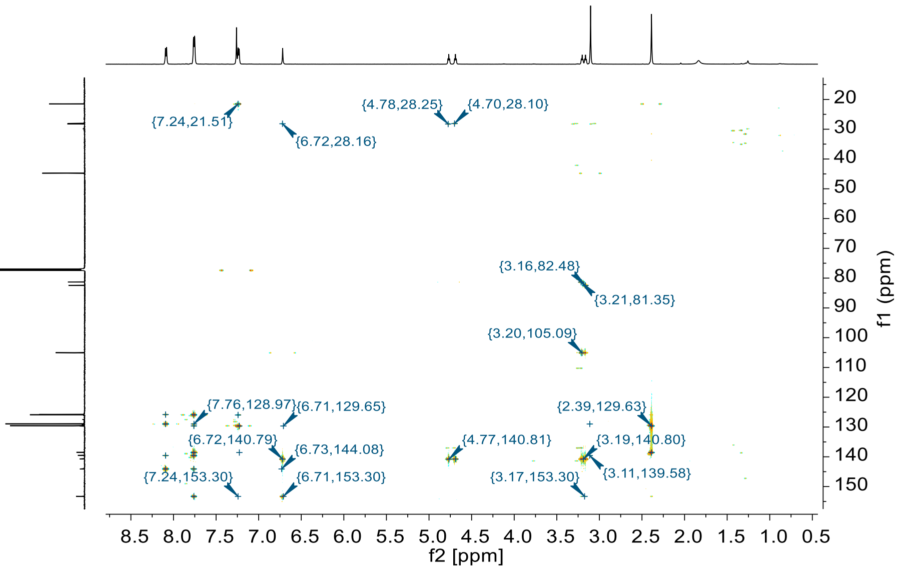

	f1 (ppm)	f2 (ppm)		f1 (ppm)	f2 (ppm)
1	129.0	8.09	18	144.1	6.73
2	129.0	7.76	19	144.1	7.76
3	21.5	7.24	20	138.6	2.39
4	129.0	3.11	21	144.1	8.09
5	129.6	7.76	22	153.3	3.17
6	129.6	7.23	23	153.3	7.24
7	129.6	2.39	24	153.3	6.71
8	126.0	7.24	25	153.3	7.76
9	28.1	4.70	26	140.8	4.77
10	28.2	6.72	27	138.6	7.76
11	125.9	7.76	28	138.6	7.23
12	28.2	4.78	29	140.8	3.19
13	81.4	3.21	30	139.6	8.09
14	125.8	8.09	31	139.6	7.76
15	82.5	3.16	32	140.8	6.72
16	105.1	3.20	33	139.6	3.11
17	129.6	6.71	34	140.8	4.69

Figure S105. HMBC spectrum of compound **5d** in $CDCl_3$

9. References

1. M. J. Uddin, B. C. Crews, K. Ghebreselasie, I. Huda, P. J. Kingsley, M. S. Ansari, M. N. Tantawy, J. J. Reese and L. J. Marnett, *Cancer Prev. Res.*, 2011, **4**, 1536-1545.
2. V. Weber, P. Coudert, E. Duroux, F. Leal, J. Couquelet and M. Madesclaire, *Arzneimittelforschung*, 2001, **51**, 877-884.
3. D. S. Pedersen and C. Rosenbohm, *Synthesis*, 2001, **2001**, 2431-2434.
4. G. M. Sheldrick, *SHELXS/L-14, Programs for the Refinement of Crystal Structures*, University of Göttingen, Göttingen (Germany), 2014.
5. G. M. Sheldrick, *Acta Crystallographica Section C: Structural Chemistry*, 2015, **71**, 3-8.
6. T. Rahn, F. Bendrath, M. Hein, W. Baumann, H. Jiao, A. Börner, A. Villinger and P. Langer, *Org. Biomol. Chem.*, 2011, **9**, 5172-5184.
7. M. Laube, C. Gassner, S. K. Sharma, R. Gunther, A. Pigorsch, J. Konig, M. Kockerling, F. Wuest, J. Pietzsch and T. Kniess, *J. Org. Chem.*, 2015, **80**, 5611-5624.
8. S. F. Donovan and M. C. Pescatore, *J. Chromatogr. A*, 2002, **952**, 47-61.
9. C. Gassner, C. Neuber, M. Laube, R. Bergmann, T. Kniess and J. Pietzsch, *ChemistrySelect*, 2016, **1**, 5812-5820.
10. R. Wodtke, C. Hauser, G. Ruiz-Gomez, E. Jackel, D. Bauer, M. Lohse, A. Wong, J. Pufe, F. A. Ludwig, S. Fischer, S. Hauser, D. Greif, M. T. Pisabarro, J. Pietzsch, M. Pietsch and R. Loser, *J. Med. Chem.*, 2018, **61**, 4528-4560.
11. T. Kniess, M. Laube, R. Bergmann, F. Sehn, F. Graf, J. Steinbach, F. Wuest and J. Pietzsch, *Bioorg. Med. Chem.*, 2012, **20**, 3410-3421.
12. C. Neuber, B. Belter, S. Meister, F. Hofheinz, R. Bergmann, H. J. Pietzsch and J. Pietzsch, *Molecules*, 2018, **23**, 444.
